# Building Metabolically Stable and Potent Anti-HIV Thioether-Lipid Analogues of Tenofovir Exalidex: A thorough Pharmacological Analysis

**DOI:** 10.1021/acs.jmedchem.4c01510

**Published:** 2024-10-16

**Authors:** Michael P. D’Erasmo, Savita K. Sharma, Nicole Pribut, Adriaan Basson, Madhuri Dasari, Perry Bartsch, Sabrina E. Iskandar, Kyle E. Giesler, Samantha Burton, Cindy A. Derdeyn, Dennis C. Liotta, Eric J. Miller

**Affiliations:** † Department of Chemistry, Emory University College of Arts & Sciences, Atlanta, Georgia 30322, United States; ‡ HIV Pathogenesis Research Unit, Department of Molecular Medicine and Haematology, University of the Witwatersrand, Johannesburg 2000, Gauteng, South Africa; § Department of Laboratory Medicine & Pathology, University of Washington School of Medicine, Seattle, Washington 98195, United States; ∥ Department of Pharmacology & Chemical Biology, Emory University School of Medicine, Atlanta, Georgia 30322, United States

## Abstract

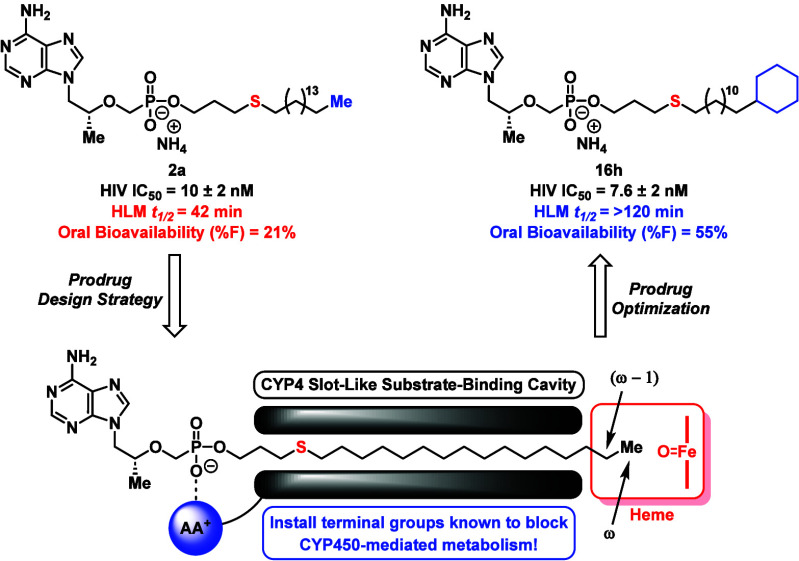

Inherently limited by poor bioavailability, antiviral agent tenofovir (TFV) is administered to people living with HIV in prodrug form. However, current prodrugs are prematurely metabolized, compromising access to HIV-infected cells and inducing toxicity. Inspired by lipid conjugate TFV exalidex (TXL), we recently disclosed TXL analogs with potent activity and robust hepatic stability in vitro, as well as attractive oral PK profiles in vivo. In parallel, we discovered the equipotent and equally stable hexadecylthiopropyl (HTP) derivative of TXL (**2a**). Reported herein are the synthetic and bioanalytic efforts that led to potent, safe, and hepatically stable HTP derivatives. While HTP analog **16h** showed the most attractive PK profile in mice (55% F) discrepancies in translating in vitro cell-based results to in vivo PK data, for certain prodrugs, indicated that further in vitro/in vivo optimization is required for continued advancement of this program.

## Introduction

**Figure 1:**
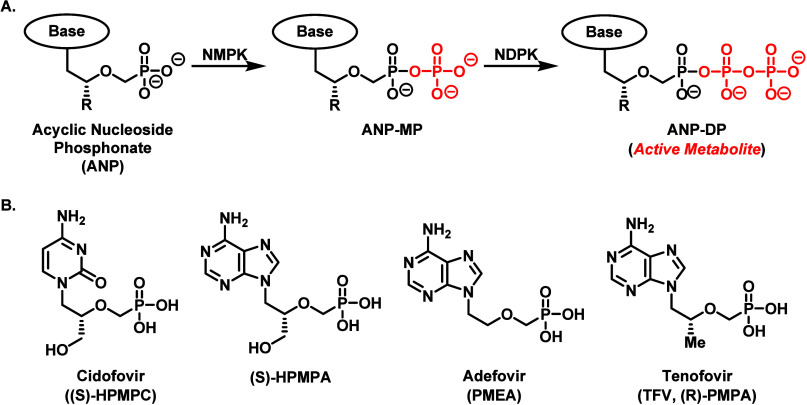
Conversion of ANPs to their active metabolite via enzyme-catalyzed phosphorylation by nucleoside kinases (A) and representative structures for clinically relevant ANPs (B). NMPK, nucleoside monophosphate kinase; NDPK, nucleoside diphosphate kinase.

Very little perusing of scientific literature is required to determine how clinically impactful nucleoside analogs have been with regard to antiviral treatment.^1−6^ Among the commercially successful nucleoside therapies, acyclic nucleoside phosphonates (ANPs, Figure 1) have achieved considerable acclaim due to their broad-spectrum antiviral activity, low drug resistance, controllable toxicological profile, and availability in highly potent oral prodrug formats.^3,7,8^ Mechanistically speaking, the therapeutic function of ANPs requires transformation into an active metabolite via two phosphorylation events catalyzed by cytosolic enzymes, such as nucleotide kinase and nucleoside diphosphate kinase (Figure 1A). The activated species then serves as an inhibitor/substrate for either pathogenic RNA and/or DNA polymerases. The primary advantages of ANPs over their cyclic nucleoside and nucleotide brethren stems from the ingenious insertion of a phosphonate moiety, which is not only metabolically stable to premature hydrolytic processing by physiologically abundant phosphatase enzymes, but also enables ANP drugs to bypass the first phosphorylation step toward activation, which is the bottleneck for many synthetic nucleoside therapeutics.^7−9^ However, the phosphonate component, which is negatively charged at physiological pH, limits the permeability of ANP drugs into virally infected cells and impedes oral bioavailability. Fortunately, various prodrug strategies have been successfully applied to ANPs to overcome these limitations.^10−15^

Tenofovir (TFV), a crucial ANP drug in the antiviral toolbox of medicinal chemists and medical practitioners, has a rich history of prodrug development.^3,12−16^ Therapeutically, TFV is a 2′,3′-dideoxyadenine analog that elicits broad-spectrum antiviral activity against human immunodeficiency virus (HIV),^16−18^ hepatitis B virus (HBV),^19,20^ and herpes simplex virus type-2 (HSV-2).^21^ Nevertheless, the uncapped phosphonate functionality of TFV imparts undesirable pharmacokinetic (PK) properties and poor oral bioavailability in clinical models, necessitating the creation of TFV prodrugs. As a result, the two most prevalent scaffolds that emerged in the race to improve TFV’s therapeutic potential and oral absorption are the phosphonate diester prodrug, tenofovir disoproxil fumarate (TDF),^11,17,22^ and the phosphonamidate prodrug, tenofovir alafenamide (TAF).^23^ Both prodrugs can be found in an assortment of commercially approved combination antiviral therapies used today as the backbones of anti-HIV therapeutics.^2,15^

**Figure 2:**
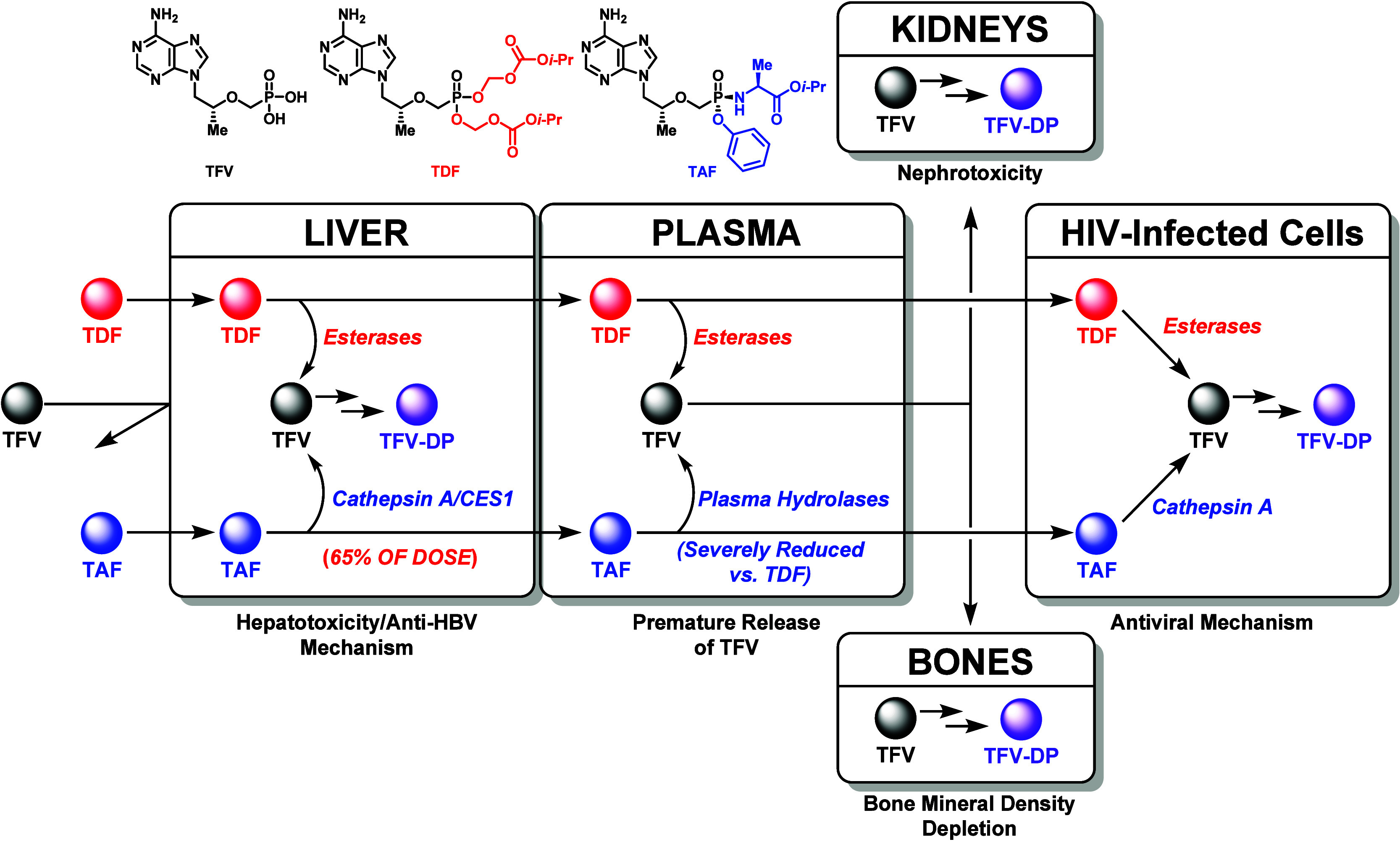
Mechanism of action and metabolic profile of TFV prodrugs in vivo.

The intrigue with TDF and TAF goes far beyond the simple masking of anionic character imposed by the TFV phosphonate moiety and generating a more lipophilic compound with amplified intestinal permeability. These prodrugs depend on unique enzyme-mediated cleavage mechanisms to physiologically distribute parent TFV, each with advantages and disadvantages (Figure 2).^15^ For example, TDF strategically relies upon esterases to release a TFV payload into virally infected cells. Through the installation of isopropyloxycarbonyloxymethyl (POC) esters on TFV, TDF provides a significantly higher oral bioavailability (i.e., 25–40% depending on food intake) versus the parent drug.^10,11^ However, due to the ubiquitous nature of esterase enzymes, TDF undergoes premature cleavage in the liver and plasma and elicits adverse side-effects over long-term use. Some TDF-induced toxic events, include: (1) liver function alteration through the accumulation of TFV-diphosphate (TFV-DP) in hepatocytes,^22^ (2) nephrotoxicity promoted by TFV renal tubular efflux,^24,25^ and (3) bone mineral density depletion driven by osteoclast dysfunction from TFV aggregation.^26^ Contrary to TDF, TAF possesses a phosphoramidate-based masking group (ProTide technology), which depends upon intracellular cleavage mechanisms for TFV release.^27^ Specifically, TAF is a substrate for the lysosomal serine protease, cathepsin A,^28^ and the serine hydrolase, carboxylesterase I (CES1), located in the cytoplasm and endoplasmic reticulum.^29^ The ProTide prodrug approach not only provides increased intracellular levels of TFV in target cells but imparts higher metabolic stability in plasma. As a result, TAF confers significantly reduced nephrotoxicity and TFV plasma exposure in comparison to TDF.^15,30^ Nevertheless, the distribution profile of TAF is severely limited to the liver (i.e., 65% of oral dose undergoes hepatic processing in dogs) since considerable levels of cathepsin A and carboxylesterase I are found in hepatocytes.^27−29,31,32^ Though TAF was repurposed as an HBV treatment in 2016,^33^ ProTide-based prodrugs do not mechanistically provide the ideal distribution profile required to tackle a more organ diffuse viral target, such as HIV. Furthermore, these agents necessitate strict adherence to once daily oral treatment schedules to prevent viral rebound, the onset of drug resistance, and progression to AIDS. And this essential strict adherence is directly compromised by deleterious drug-induced side effects like renal toxicity and bone mineral density depletion.

**Figure 3:**
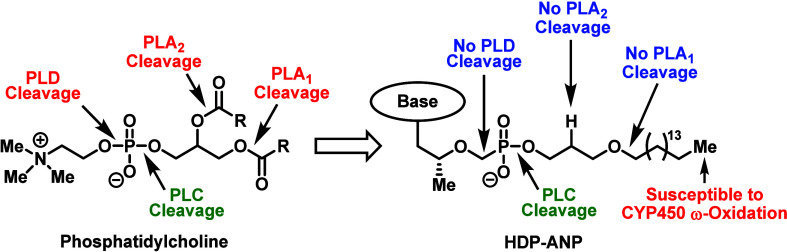
Hostetler and co-worker’s lipid-based strategy to enhance the oral absorption and reduce the toxicity of nucleoside drugs.

In order to mitigate the long-term side-effects associated with POC-based prodrugs, such as TDF, Hostetler and co-workers developed a lipid-based strategy inspired by lysoglycerophospholipids (Figure 3).^13^ After years of modifications, the Hostetler group introduced the key hexadecyloxypropyl (HDP) moiety, which was installed on ANP cidofovir to create brincidofovir (CMX-001)^34^ and on TFV to generate TFV exalidex (TXL, CMX-157).^35^ Mechanistically, the prodrugs are predominantly cleaved intracellularly by membrane-associated enzyme, especially phospholipase C (PLC) and sphingomyelinase.^13^ By targeting these intracellular catabolic processes, the prodrugs exhibited favorable PK properties and safety profiles in relation to TDF (i.e., increased plasma stability and abated nephrotoxicity). However, just like TAF, the HDP scaffold undergoes significant metabolism in the liver since the terminal methyl group found on the lipid component is susceptible to cytochrome P450 (CYP450)-mediated ω-oxidation.^13,36−38^

**Figure 4:**
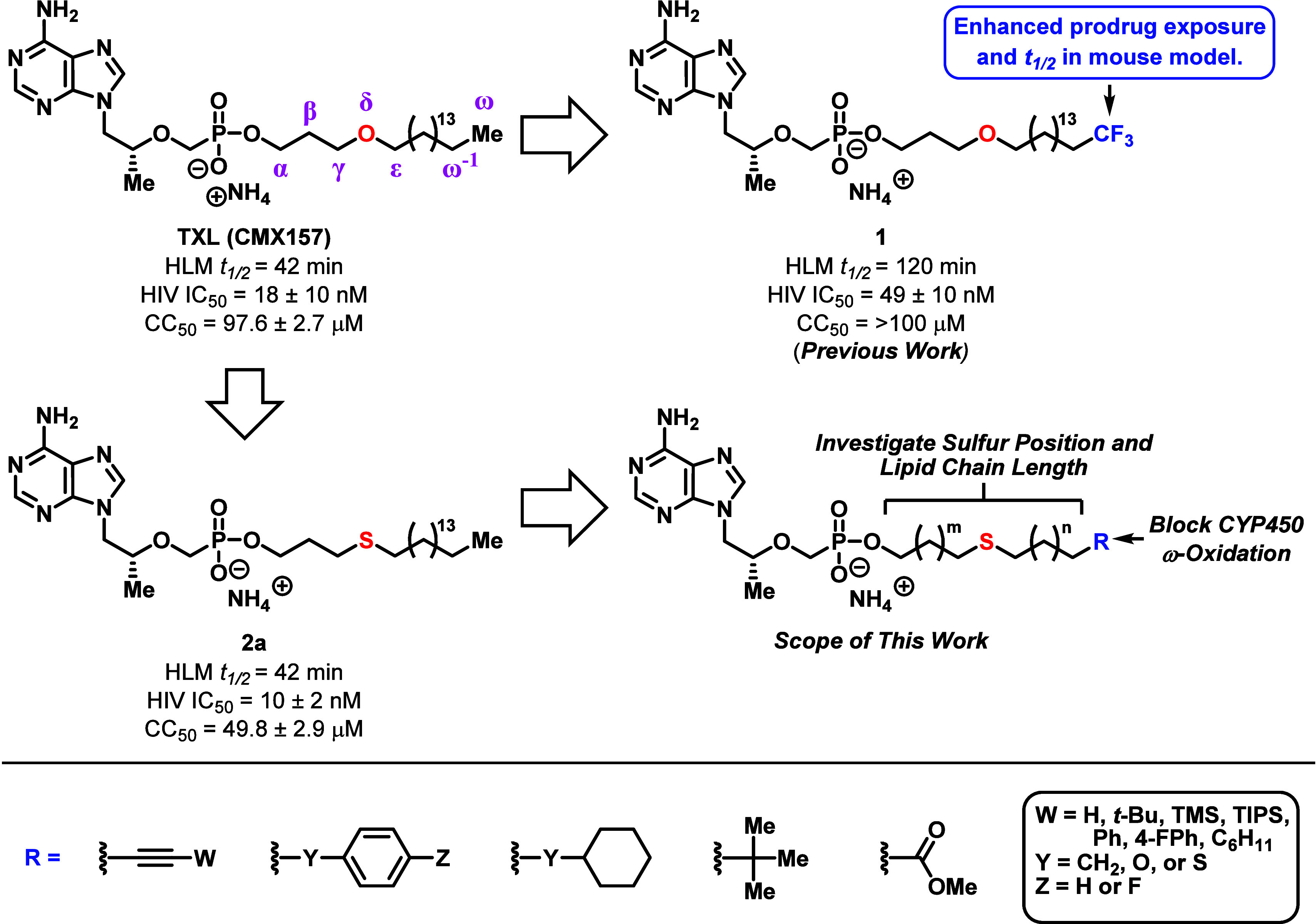
Summary of the pharmacological properties and drug design parameters for our HDP- and HTP-derived prodrugs. The scope of this study entails the optimization of HTP-based scaffolds through systematic investigation of sulfur position, lipid chain length, and newly integrated terminal motifs designed to resist CYP450-mediated ω-oxidation.

Despite the limitation with HDP-derived prodrugs, our lab seized upon an opportunity to improve the pharmacological properties of TXL and developed novel analogs capable of resisting ω-oxidation in vitro and in vivo.^39,40^ More specifically, by functionalizing the lipid terminus with chemical moieties designed to resist CYP450-mediated oxidation, we discovered the ω-CF_3_ prodrug **1**, which displayed similar in vitro antiviral activity and cytotoxicity, improved human liver microsome (HLM) stability, and enhanced exposure levels and prodrug *t*_1/2_ in mice (Figure 4) compared to TXL. During these studies, we also discovered that the HDP component could be replaced with a hexadecylthiopropyl (HTP) group, resulting in compound **2a** that showed comparable HIV activity (IC_50_ = 10 nM) and HLM metabolic half-life (*t*_1/2_ = 42 min) to TXL. Though the cytotoxic potential of **2a** (CC_50_ = 50 μM) was 2-fold greater than TXL (CC_50_ = 98 μM), the molecule still exhibited an impressive therapeutic index of approximately 5000. Considering that substituting the HDP oxygen atom with a sulfur atom presents certain chemical and biological advantages (e.g., ease of nucleophilic reactions under mild basic conditions and potentially higher cellular permeability due to increased lipophilicity),^41,42^ we saw **2a** as a great starting point for developing potent TXL analogs with elevated biological distribution and robust drug safety profiles. Additionally, since CYP450-mediated ω-oxidation of a lipid substrate is mechanistically controlled by a variety of factors, including steric effects, electronegativity, and bond dissociation energies,^38,43−47^ we used the HTP platform to further explore the prodrug terminal motif. Herein, we outline the synthesis and systematic pharmacological optimization of these HTP-derived TFV prodrugs, with detailed investigations of sulfur position, lipid chain length, and newly installed CYP450-resistant terminal motifs. Several of these compounds displayed HLM stability that far exceeded TXL, while maintaining potent anti-HIV activity and safe cellular cytotoxicity levels in vitro. Additionally, one prodrug demonstrated 2-fold greater oral bioavailability than TXL in vivo.

## Results and Discussion

### Organic Synthesis

**Scheme 1:**
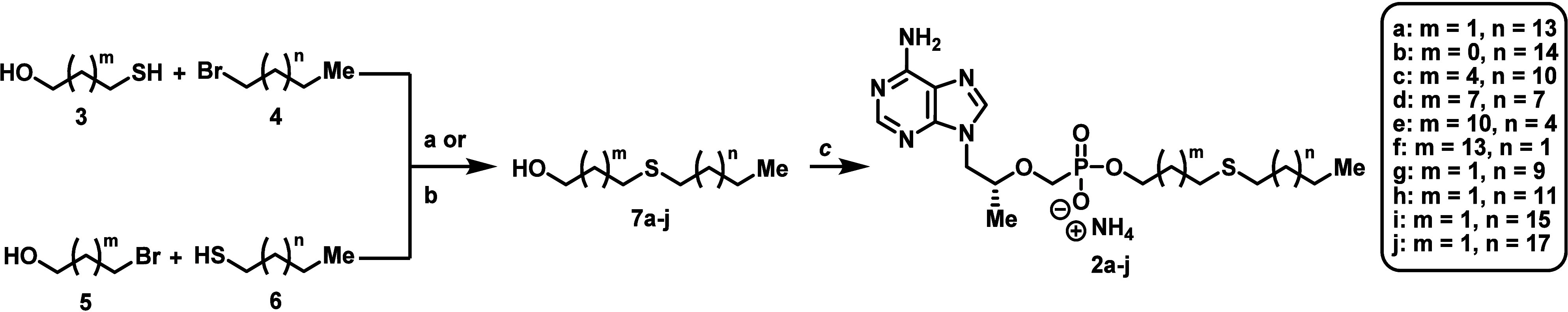
Synthesis of HTP-Derived TFV Prodrugs with Modified Sulfur Atom Position and Chain Length Reagents and conditions: (a) CsCO_3_, DMF, rt, 16 h, 92%; (b) DBU, DMF, rt to 60–65 °C, 1.5–4 h, 76 → 95%; (c) TFV, DCC, Et_3_N, DMAP, DMF or NMP, 100 or 105 °C, 18–24 h, 30–69%.

Lipid analogs with modifications to sulfur position and chain length were synthesized utilizing the same two-step reaction sequence (Scheme 1). First, mercaptoalcohols (**3**) or alkyl thiols (**6**) were efficiently alkylated with the corresponding alkyl bromides (**4**) or bromo alcohols (**5**) respectively in the presence of 1,8-diazabicyclo[5.4.0]undec-7-ene (DBU) or cesium carbonate (Cs_2_CO_3_). Next, the resulting thioether-containing lipid alcohols **7a**–**j** were conjugated to TFV using *N,N*′-dicyclohexylcarbodiimide (DCC) in combination with triethylamine (Et_3_N) and 4-(dimethylamino)pyridine (DMAP) to produce the final compounds **2a**–**j** in low to moderate yields (30–69%). As shown in Scheme 1, these thioether-containing TXL derivatives, along with the remaining analogs described in our report, were obtained as ammonium salts due to the required use of ammonium hydroxide (NH_4_OH) as an additive for column chromatography.

**Scheme 2:**
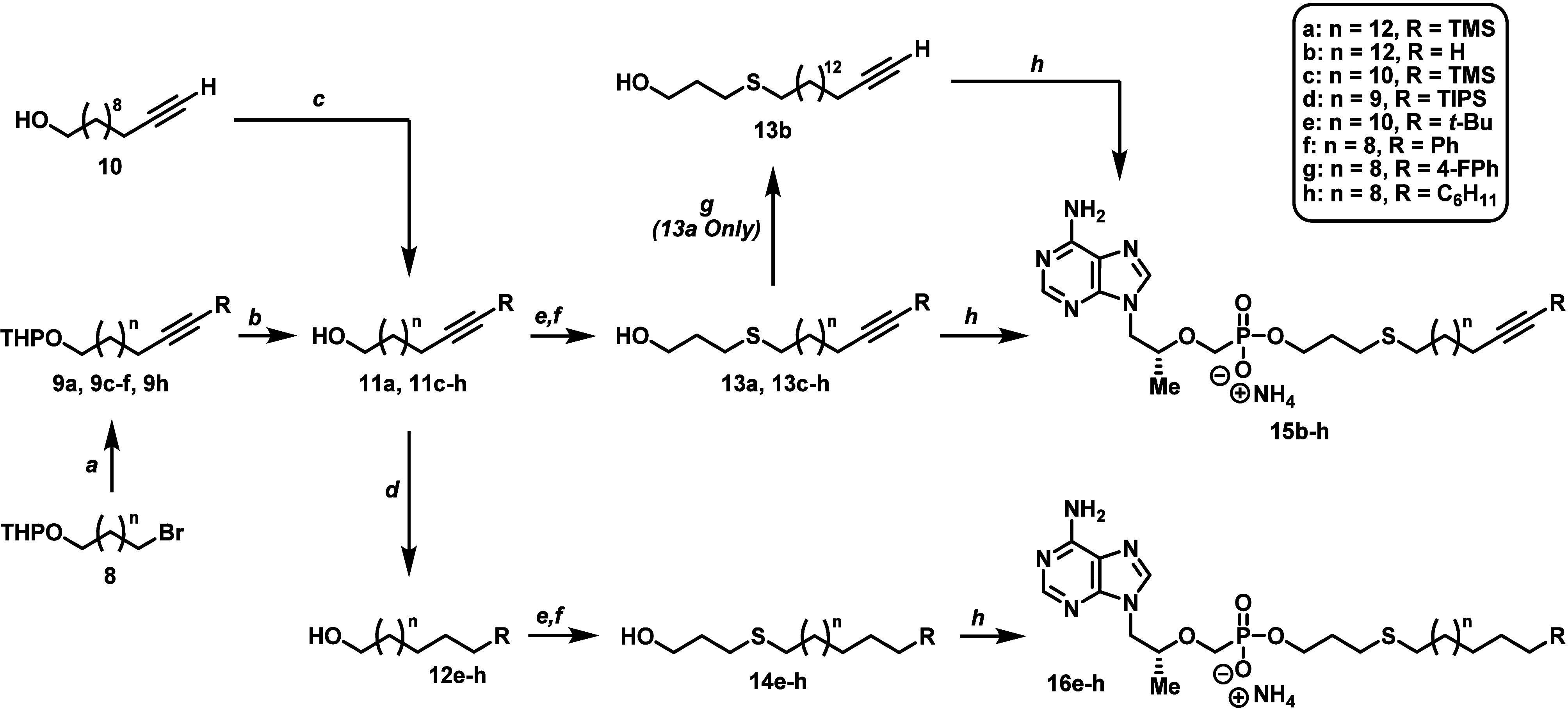
Synthesis of ω-Functionalized HTP-Derived TFV Prodrugs Featuring Alkynyl- and Alkyl-Based Terminal Motifs Reagents and conditions: (a) substituted acetylene, *n*-BuLi, HMPA, THF, −78 to −40 °C, 1 h then −40 °C to rt, 5–24 h, 84–94%; (b) *p*-TsOH·H_2_O, MeOH, rt, overnight, 69–91%; (c) 1-fluoro-4-iodobenzene, Pd(PPh_3_)_2_Cl_2_, CuI, Et_3_N, THF, rt to 70 °C, overnight, 62%; (d) 10% Pd/C, H_2_, MeOH or EtOAc, rt, 3–16 h, 55–83%; has MsCl, Et_3_N, DCM, 0 °C to rt, 45 min; (f) 3-mercaptopropanol, DBU, DMF, rt to 60–65 °C, 1.5–5 h, 68–92% over two steps; (g) TBAF, THF, rt, 45 min, 87%; (h) TFV, DCC, Et_3_N, DMAP, NMP, 100 °C, 18–24 h, 35–61%.

With regards to our target ω-functionalized lipid prodrugs (**15b**–**h** and **16e**–**h**), the majority were constructed by starting with tetrahydropyranyl (THP)-protected bromo alcohols of varying chain length (**8**, Scheme 2). (Alkynyloxy)tetrahydropyran series **9** was then generated in high yield (84–94%) through a sequential treatment of commercially available asymmetrically functionalized acetylenes with *n*-butyllithium (*n*-BuLi) followed by nucleophilic substitution on bromide compounds **8**. Next, alkynols **11a**, **11c**–**f**, and **11h** were acquired by exposing the corresponding THP-protected alkynes (**9**) to standard deprotection conditions with a catalytic amount of *p*-toluenesulfonic acid (p-TsOH) in MeOH. Alternatively, alkynol **11g** was directly synthesized using conventional Sonogashira coupling between 11-dodecyn-1-ol (**10**) and 1-fluoro-4-iodobenzene with a moderate 62% yield. Specific alkynols (**11e**–**h**) were then reduced with palladium on carbon (Pd/C) in either MeOH or EtOAc to generate lipid alcohols **12e**–**h**. Even with solvents and catalysts known to slow hydrogenations, the silyl groups of compounds **11a**, **11c**, and **11d** did not survive alkyne reduction toward the corresponding saturated lipids (results not shown). The collection of alkynols (**11a** and **11c**–**h**) and alcohols (**12e**–**h**) were subsequently converted to thioether-containing lipid alcohols **13a**, **13c**–**h**, and **14e**–**h** through an efficient two-step reaction sequence involving alcohol mesylation followed by nucleophilic displacement with 3-mercaptopropanol in the presence of DBU (68–92% yield over two steps). Trimethylsilyl (TMS) acetylene **13a** was further processed to terminal alkyne **13b** by simple tetrabutylammonium fluoride (TBAF)-mediated silyl deprotection. Lastly, the newly synthesized thioether lipids were coupled to TFV using the DCC procedure outlined above to produce final compounds **15b**–**h** and **16e**–**h**.

**Scheme 3:**
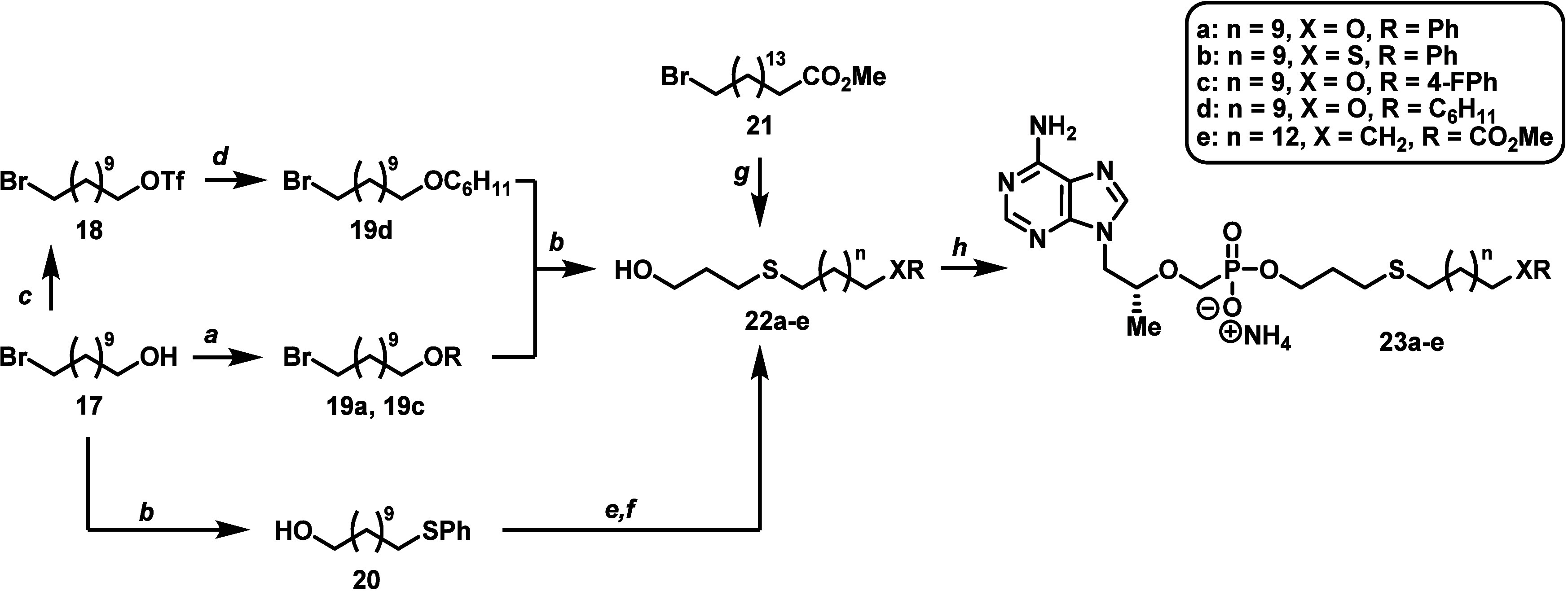
Synthesis of ω-Functionalized HTP-Derived TFV Prodrugs with Alternative Phenyl and Ester Terminal Motifs Reagents and conditions: (a) phenol, DIAD, PPh_3_, THF, 0 °C to rt, overnight, 70–77%; (b) thiol, CsCO_3_, DMF, rt, 1–16 h, 84–95%; (c) triflic anhydride, 2,6-lutidine, DCM, −78 °C, 30 min, 61%; (d) cyclohexanol, *n*-BuLi, HMPA, THF, −78 to 0 °C, 1 h then −78 to 0 °C, 5 h, 74%; (e) MsCl, Et_3_N, DCM, 0 °C to rt, 45 min; (f) 3-mercaptopropanol, DBU, DMF, rt to 60 °C, 3 h, 68% over two steps; (g) 3-mercaptopropanol, DBU, DMF, rt to 60 °C, 2 h, 88% over two steps; (h) TFV, DCC, Et_3_N, DMAP, DMF or NMP, 100 or 105 °C, 18–24 h, 32–54%.

The synthesis for the remaining batch of designed thioether-containing TXL derivatives (**23a**–**e**, Scheme 3) started with 11-bromo-1-undecanol (**17**) or methyl 16-bromohexadecanoate (**21**). Phenyl ether derivatives **19a** and **19c** were first constructed using a standard Mitsunobu reaction between **17** and the corresponding phenols in the presence of diisopropyl azodicarboxylate (DIAD) and triphenylphosphine (PPh_3_). On the other hand, cyclohexyl ether **19d** was more difficult to produce, requiring a sequential alcohol triflation of **17**(48) followed by a nucleophilic substitution with lithium cyclohexanolate under cold conditions. Next, the thioether-containing lipid alcohols **22a**, **22c**, and **22d** were efficiently synthesized (i.e., 84–95% yield) by a simple nucleophilic substitution between bromide series **19** and 3-mercaptopropanol with Cs_2_CO_3_ as base. This reaction was also used to generate thiobenzene **20**, which was carried forward to lipid **22b** according to the two-step mesylation/nucleophilic displacement outlined in Scheme 2. Additionally, ester moiety **22e** was obtained in a similar manner to **22a**, **22c**, and **22d** by starting with bromide **21** and using DBU as the base for 3-mercaptopropanol deprotonation. Ultimately, lipid alcohols **22a**–**e** were coupled to TFV to yield the final set of prodrugs analyzed for this study (**23a**–**e**).

### In Vitro Antiviral Potency and Metabolic Stability Evaluation for HTP-Derived Prodrugs

All newly synthesized HTP-derived TFV prodrugs (**2a**–**j**, **15b**–**h**, **16e**–**h**, and **23a-e)** underwent a preliminary pharmacological screen in vitro to ascertain HIV activity (IC_50_), cellular cytotoxicity (CC_50_), and HLM *t*_1/2_. The initial biological screen was conducted using assays and procedures our lab has previously reported (see Supporting Information).^39,40^ In brief, the metabolic *t*_1/2_ for each final compound was deduced in HLM (1 mg/mL) with the assistance of an LC-MS/MS to evaluate the rate of degradation. Second, HIV IC_50_ and cellular CC_50_ were determined with a pseudoviral, whole (HEK293T) cell, nonreplicative HIV assay, using the HIV-1 subtype C strain MJ4 (refer to Experimental Methods). To account for the unique amphiphilic properties of TXL and the novel HTP-based prodrugs, all compounds were formulated in a human serum albumin (HSA) solution (5:1 drug:protein). Consequently, the HIV potency for our designed prodrugs depended on several factors, including: (1) association/dissociation with HSA, (2) cell permeability, and (3) PLC kinetics for lipid cleavage.^39^ As the parent compound (TFV) is identical in all cases, activity differences between prodrugs hinged on disruptions or amplifications to these processes.

Considering that sulfur is chemically and biologically distinct from oxygen (i.e., lower electronegativity, larger atomic size, susceptibility to oxidation, etc.),^41,42^ a systematic structural evaluation of the novel HTP-based TFV prodrugs was necessary. Initially, we synthesized a series of lipid analogs with alterations in sulfur atom position and chain length to analyze how these changes could impact the HIV activity, metabolic stability, and cellular cytotoxicity for this compound class (Table 1). The results suggest that the more sterically or electronically accessible the sulfur atom is toward CYP450-mediatiated oxidation, the lower the metabolic stability of our prodrugs.^38^ Compound **2a** displays an identical HLM stability profile to TXL (*t*_1/2_ = 42 min), which is notable due to the thioether’s position, which may provide steric or electronic protection against CYP450 enzymes. We were interested in defining which lipid positions the sulfur atom could occupy without negatively influencing the prodrug’s pharmacological properties. Additionally, since thioethers are generally more lipophilic than their ether counterparts, the lipid chain length threshold for HIV potency and cytotoxicity, which depend on compound solubility, cell permeability, and rate of PLC cleavage, could be severely influenced.^13,34,35,39,40^ Thus, we first aimed to identify the optimal thioether placement and linear atom count for our lipid prodrugs.

**Table 1:** In Vitro HIV (MJ4) Activity and HLM Stability Profiles of HTP-Derived TFV Prodrugs with Varied Sulfur Position and Chain Length

compound	lipid motif	linear atom #	HLM *t*_1/2_ (min)^a^ [% remaining]^b^	HIV (MJ4) IC_50_ (μM)^a^	CC_50_ (μM)^a^	therapeutic index^c^
**TXL**	C_3_H_6_–O-C_16_H_33_	20	41.6 ± 9.0^d^	0.018 ± 0.010^d^	97.6 ± 2.7	5420
**2a**	C_3_H_6_–S-C_16_H_33_	20	41.7 ± 2.3	0.010 ± 0.002^d^	49.8 ± 2.9	4980
**2b**	C_2_H_4_–S-C_17_H_35_	20	66.1 ± 2.6	0.004 ± 0.003	28.6 ± 3.7	7150
**2c**	C_6_H_12_–S-C_13_H_27_	20	66.2 ± 3.0	0.073 ± 0.011	>100	>1370
**2d**	C_9_H_18_–S-C_10_H_21_	20	13.3 ± 0.5	0.058 ± 0.009	>100	>1720
**2e**	C_12_H_24_–S-C_7_H_15_	20	20.6 ± 0.1	0.338 ± 0.004	>100	>296
**2f**	C_15_H_6_–S-C_4_H_9_	20	19.7 ± 0.003	0.302 ± 0.062	>100	>331
**2g**	C_3_H_6_–S-C_12_H_25_	16	11.3 ± 0.2	0.583 ± 0.188	>100	>532
**2h**	C_3_H_6_–S-C_14_H_29_	18	15.6 ± 0.1	0.007 ± 0.006	>100	>16,700
**2i**	C_3_H_6_–S-C_18_H_37_	22	>120 [>95%]	n.d.	n.d.	n.d.
**2j**	C_3_H_6_–S-C_20_H_41_	24	>120 [>95%]	n.d.	n.d.	n.d.

a *n* = 2.

b Relative percent remaining of prodrug at 120 min. Linear atom # does not count H or F.

c Therapeutic index = CC_50_/HIV (MJ4) IC_50_.

d *n* = 4.

As illustrated with compounds **2a**–**f** in Table 1, thioether placement on the lipid prodrugs clearly affected all studied in vitro parameters. Situating the thioether closer to the phosphonate functionality, as in compound **2b**, produced slight improvements in both HIV activity (IC_50_ = 4 nM) and HLM stability (*t*_1/2_ = 66 min), but resulted in an estimated 2-fold and 4-fold increase in cytotoxicity (CC_50_ = 29 μM) over **2a** and TXL, respectively. Despite the elevation in cellular toxicity, **2b** maintained a therapeutic index of over 7000 and presented another starting scaffold for prodrug modification. On the other hand, the data for compounds **2c**–**f** indicated that installing the thioether further down the lipid chain severely reduced HIV activity (IC_50_ = 60–340 nM) and/or HLM tolerance (*t*_1/2_ = 13–21 min). While compound **2c** possessed a metabolic stability comparable to **2b** (*t*_1/2_ = 66 min), the prodrug showed an 18-fold decrease in HIV potency (IC_50_ = 73 nM), providing a threshold for thioether placement on our HTP-derived analogs. On the other hand, compounds **2d**–**f** demonstrated drastic declines in both antiviral activity and HLM *t*_1/2_, despite possessing better cytotoxicity profiles over **2a** and **2b** (CC_50_ > 100 μM). Ultimately, the results observed with **2d**–**f** support our initial hypothesis that the HLM stability for HTP-derived prodrugs depends on the accessibility of the sulfur atom toward CYP450-mediated oxidation. Furthermore, in terms of the intracellular availability of **2c**–**f** to inhibit HIV activity, further study is required to pinpoint how the thioether position mechanistically disrupts HSA binding, cell permeability, and/or PLC cleavage. When taking Table 1 into account, **2a** and **2b** revealed that the γ and δ positions on the lipid chain (see Figure 4) are pharmacologically optimal for the thioether. Though **2b** is a potential starting point for future drug development, we decided to move forward with prodrug **2a** for structural consistency with TXL and our previously reported analogs.^39,40^

As anticipated, compounds **2a** and **2g**–**j**, with lipid moieties ranging from 16 to 24 atoms, demonstrated the significant impact chain length has on both HIV activity and HLM stability, following the pattern observed for alkoxyalkyl-based TFV prodrugs reported previously (Table 1).^13,34,35,39,40^ For example, while compound **2h** (18 atoms) maintained high antiviral potency (IC_50_ = 7 nM) in relation to **2a** (20 atoms), the shorter lipid accelerated HLM metabolism (*t*_1/2_ = 15.6 min). When the prodrug was further truncated, as in **2g** (16 atoms), dramatic loses in both HIV activity (IC_50_ = 583 nM) and HLM stability (*t*_1/2_ = 11.3 min) were observed. Conversely, extension of the lipid moiety beyond 20 atoms (**2i** and **2j**) imparted considerable resistance to CYP450-mediated oxidation (*t*_1/2_ > 120 min with 95% prodrug remaining). Unfortunately, **2i** and **2j** could not be tested for antiviral potency and cell cytotoxicity because of poor solubility in the assay medium, despite formulation with HSA. Nevertheless, due to the promising metabolic profile of **2i** and **2j**, our lab is continuing to analyze TXL analogs with chain lengths beyond 20 atoms and will disclose a report in the future. However, for the sake of this study, **2a** remained as the most pharmacologically attractive starting point for ω-functionalization. Hence, with one exception (refer to **23e**), we focused on prodrugs containing lipid moieties with a linear atom count of 20 atoms.

**Figure 5:**
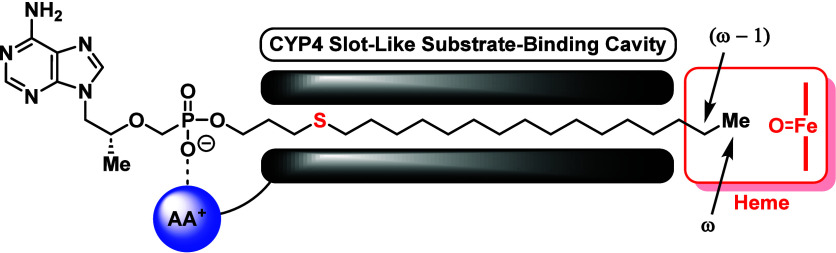
Proposed binding mode for substrates of CYP-4 enzymes, using compound **2a** as an example. AA^+^ = Positively Charged Amino Acid.

Upon discovering the most pharmacologically attractive starting point for our thioether scaffolds, we moved toward installing ω-functionalized lipids on TFV to block CYP450-mediated oxidation at the terminal position. Apart from chain length^45^ and bond-dissociation energy considerations,^39,43^ previous structure–activity relationship (SAR) studies on fatty acid hydroxylases (i.e., CYP450 isoform family 4 enzymes, CYP-4) have obstructed ω-oxidation of an unbranched lipid substrate through the strategic installation of steric bulk (cycloalkanes or aromatic rings)^44^ or large sized atoms (bromo or iodo)^47^ at the terminus. Generally, the observed steric control occurs due to the structural characteristics of the CYP-4 catalytic site, which possesses a narrow, sterically restricted channel near the heme moiety to allow for regioselective oxidation of the ω position on lipids (Figure 5).^49−53^ However, depending on the functional group properties at the ω-position, CYP-4 enzymes will favorably engage in ω^–1^ hydroxylation.^38^ Consequently, despite blocking the ω-position with chemical moieties known to resist CYP450 metabolism, prodrug decomposition could potentially occur at the ω^–1^-site. Therefore, motivated by these studies, we performed a systematic pharmacological analysis on a variety of alkynyl, phenyl, silyl, and bulky alkyl terminal groups to assess how to fully limit CYP450-mediated ω-oxidation without attenuating HIV potency and impacting drug safety (Tables 2 and 3).

**Table 2:** In Vitro HIV (MJ4) Activity and HLM Stability Profiles of ω-Functionalized HTP-Derived TFV Prodrugs Featuring Acetylenic Terminal Groups

compound	lipid motif	linear atom #	HLM *t*_1/2_ (min)^a^ [% remaining]^b^	HIV (MJ4) IC_50_ (μM)^a^	CC_50_ (μM)^a^	therapeutic index^c^
**TXL**	C_3_H_6_–O-C_16_H_33_	20	41.6 ± 9.0^d^	0.018 ± 0.010^d^	97.6 ± 2.7	5420
**2a**	C_3_H_6_–S-C_16_H_33_	20	41.7 ± 2.3	0.010 ± 0.002^d^	49.8 ± 2.9	4980
**15b**	C_3_H_6_–S-C_14_H_28_–C≡CH	20	95.8 ± 1.8	0.028 ± 0.0002	>100	>3570
**15c**	C_3_H_6_–S-C_12_H_24_–C≡CTMS	20	>120 [82%]	0.024 ± 0.0001	>100	>4170
**15d**	C_3_H_6_–S-C_11_H_22_–C≡CTIPS	20	>120 [>95%]	0.265 ± 0.069^d^	59.3 ± 2.4	224
**15e**	C_3_H_6_–S-C_12_H_24_–C≡C*t*-Bu	20	>120 [71%]	0.194 ± 0.044^c^	>100	>516
**15f**	C_3_H_6_–S-C_10_H_20_-C≡CPh	20	71.3 ± 1.1	0.008 ± 0.0004	>100	>12,500
**15g**	C_3_H_6_–S-C_10_H_20_-C≡C4-FPh	20	>120 [67%]	0.010 ± 0.002	>100	>10,000
**15h**	C_3_H_6_–S-C_10_H_20_-C≡CC_6_H_11_	20	>120 [70%]	0.013 ± 0.002	>100	>7580

a *n* = 2.

b Relative percent remaining of prodrug at 120 min. Linear atom # does not count H or F.

c Therapeutic index = CC_50_/HIV (MJ4) IC_50_.

d *n* = 4.

**Table 3:** In Vitro HIV (MJ4) Activity and HLM Stability Profiles of ω-Functionalized HTP-Derived TFV Prodrugs Featuring Alkyl- or Heteroatom-Linked Terminal Groups

compound	lipid motif	linear atom #	HLM *t*_*1/2*_ (min)^a^ [% remaining]^b^	HIV (MJ4) IC_50_ (μM)^a^	CC_50_ (μM)^a^	therapeutic index^c^
**TXL**	C_3_H_6_–O-C_16_H_33_	20	41.6 ± 9.0^d^	0.018 ± 0.010^d^	97.6 ± 2.7	5420
**2a**	C_3_H_6_–S-C_16_H_33_	20	41.7 ± 2.3	0.010 ± 0.002^d^	49.8 ± 2.9	4980
**16e**	C_3_H_6_–S-C_14_H_28_-*t*-Bu	20	>120 [>95%]	0.174 ± 0.045^d^	24.0 ± 1.4	138
**16f**	C_3_H_6_–S-C_12_H_24_–Ph	20	68.4 ± 0.1	0.008 ± 0.001	>100	>12,500
**23a**	C_3_H_6_–S-C_11_H_22_–OPh	20	8.4 ± 0.1	0.009 ± 0.004	>100	>11,100
**23b**	C_3_H_6_–S-C_11_H_22_–SPh	20	7.9 ± 0.2	0.050 ± 0.008	>100	>2000
**16g**	C_3_H_6_–S-C_12_H_24_-4-FPh	20	>120 [69%]	0.012 ± 0.002	>100	>8065
**23c**	C_3_H_6_–S-C_11_H_22_–O4-FPh	20	49.7 ± 0.03	0.118 ± 0.014^e^	>100	>594
**16h**	C_3_H_6_–S-C_12_H_24_–C_6_H_11_	20	>120 [85%]	0.008 ± 0.002	47.5 ± 8.7	6250
**23d**	C_3_H_6_–S-C_11_H_22_–OC_6_H_11_	20	47.9 ± 0.4	0.184 ± 0.023^d^	>100	>542
**23e**	C_3_H_6_–S-C_15_H_30_-CO_2_Me	22	5.5 ± 0.3	1.420 ± 0.675	>100	>70

a *n* = 2.

b Relative percent remaining of prodrug at 120 min. Linear atom # does not count H or F.

c Therapeutic index = CC_50_/HIV (MJ4) IC_50_.

d *n* = 4.

e *n* = 3.

Our previous report indicated that installation of substituted acetylenic functionality at the lipid terminus generally produced pharmacologically beneficial results in vitro and in vivo,^39^ and thus we initially focused on applying the same concept to the HTP-derived prodrugs (Table 2). Acetylene **15b** and TMS acetylene **15c** exhibited improvements in HLM stability (*t*_1/2_ > 95 min) and cell cytotoxicity (CC_50_ > 100 μM) while maintaining relative antiviral potency (IC_50_ = 24–28 nM) compared with TXL and **2a**. Contrarily, despite demonstrating significant resistance to metabolic oxidation (*t*_1/2_ > 120 min with >95% prodrug remaining), triisopropylsilyl (TIPS) acetylene **15d** displayed very poor HIV activity with an IC_50_ value of 265 nM. More surprisingly, we observed similar results from *tert*-butyl (*t*-Bu) acetylene **15e** (IC_50_ = 196 nM, *t*_1/2_ > 120 min with 71% prodrug remaining). While the chemical distinction between TIPS and TMS acetylenic substitution at the terminus was obvious, we were intrigued by the drastic impact to antiviral activity seen with the *t*-Bu group. Therefore, we conducted further studies on **15e** in HepaRG cells (data presented below) to better characterize this SAR. Moving forward, we were pleased to see that phenylacetylene **15f** showed HIV potency proportional to **2a** (IC_50_ = 8 nM) with a slight boost in HLM stability (*t*_1/2_ = 71 min) and appreciable reduction in cytotoxicity (CC_50_ > 100 μM). By capping a potential metabolic hotspot on the phenyl ring of **15f** with a fluorine atom, we generated prodrug **15g**, which not only performed well against HIV (IC_50_ = 10 nM) but also exhibited a desirable metabolic stability (*t*_1/2_ > 120 min with 67% prodrug remaining) and cytotoxicity (CC_50_ > 100 μM) profile relative to TXL and **2a**. Moreover, upon strategically replacing the phenyl ring with its fully saturated counterpart, we created the cyclohexyl-derived compound **15h** that was just as potent, resistant to metabolic oxidation, and safe to cells as **15g** (IC_50_ = 13 nM, *t*_1/2_ > 120 min with 67% prodrug remaining, CC_50_ > 100 μM). Ultimately, based on the data in Table 2, we thought compounds **15c**, **15g**, and **15h** met our initial screening criteria for PK analysis. However, during the discovery process, we observed qualitatively by eye (i.e., white-to-yellow solid transition) and by LC–MS (i.e., rising impurity, *m*/*z* suggests amination) that these acetylenic prodrugs can undergo spontaneous decomposition while being stored at −20 °C (refer to Figure S52 for example). Considering that our TFV prodrugs were synthesized as ammonium salts, it is possible that acetylenic degradation occurs via a slow hydroamination of the terminal triple bond. However, additional experiments are required to fully characterize this process, and salt screens must be conducted to assess the relationship between counterion properties and prodrug stability. Because the rest of the compounds reported herein were quite bench-stable, and since we do not seek to further develop these acetylenic prodrugs, no further data on these apparently unstable analogs were collected. In contrast, we sought to progress forward by avoiding the triple bond entirely.

As shown with compounds **16e**–**16h** in Table 3, our acetylene reduction strategy produced similar trends across all in vitro parameters studied. For example, with exception to an appreciable boost in cytotoxicity (CC_50_ = 24 μM), *t*-Bu analog **16e** demonstrated very poor antiviral activity (IC_50_ = 177 nM) with high metabolic stability (*t*_1/2_ > 120 min with 95% prodrug remaining) akin to **15e**. Additionally, phenyl compound **16f** exhibited a comparable profile to **15f**, with proportional HIV potency to **2a** (IC_50_ = 8 nM), a slight increase in HLM stability (*t*_1/2_ = 68 min) and low cytotoxic potential (CC_50_ > 100 μM). By addressing the metabolic hotspots on the phenyl ring terminus of **16f**, we generated our lead compounds 4-fluorophenyl **16g** and cyclohexyl **16h**, which were both effective against HIV activity (IC_50_ = 8–12 nM) and resistant to CYP450-mediated oxidation (*t*_1/2_ > 120 min) in HLM. Though the cytotoxic potential of **16h** was greater than other prodrugs in the series (CC_50_ = 48 μM), the compound possessed an attractive therapeutic index of 6250.

In addition to the ω-functionalized lipids described above, we also sought to install a fatty acid ester (**23e**) to mimic more naturally occurring substrates. Regrettably, this strategy negatively impacted all studied parameters presumably due to the metabolic instability and increased polarity imparted by the ester moiety. Next, in efforts to simplify prodrug synthesis and take advantage of the promising pharmacological behavior of the phenyl and cyclohexyl terminal group insertions found in **16f**–**h**, we constructed ethers **23a**, **23c**, and **23d** and thioether **23b**. While **23a** showed antiviral activity (IC_50_ = 9 nM) and cellular cytotoxicity (CC_50_ > 100 μM) on par with **15f** and **16f**, the compound’s HLM stability was severely impacted by the phenoxy terminal group (*t*_1/2_ = 8 min). In principle, the electronic properties of the phenoxy terminus of **23a** could enable the prodrug to bind more favorably to the heme complexes of CYP450 enzymes than phenylacetylene **15f** and phenyl **16f** to accelerate aromatic ring oxidation, which could explain the 8-fold shorter *t*_1/2_ in HLM versus its hydrocarbon counterparts. Unsurprisingly, thioether **23b** also exhibited very low metabolic stability, presumably on account of the exposed sulfur atom in proximity to the lipid terminus (refer to Table 1). Interestingly, relative to the acetylenic- and alkyl-based prodrugs described above, we observed minimal improvements in HLM stability (*t*_1/2_ = 48–50 min) when mitigating potential metabolic hotspots found in compounds **23a** and **23b** with 4-fluorophenyl (**23c**) and cyclohexyl (**23d**) substitutions. While speculative, this could suggest that dealkylation/dearylation could be a major route of metabolism for this prodrug class. Furthermore, we were intrigued by the drastic decrease in antiviral activity observed with compounds **23c** and **23d**, which gave IC_50_ values of 118 and 184 nM, respectively. While the electronic effects induced by the 4-fluorophenyl ether of **23c** could potentially disrupt HSA binding, cell permeability, and/or PLC kinetics versus **15g** and **16g**, the results for **23d** are not as easy to rationalize. One thought is that the cyclohexyl ether terminus of **23d** could interfere with the lipid membrane mechanics required for cell permeability^13^ and/or PLC cleavage. However, further studies are certainly required to pinpoint which step or steps of the TFV prodrug processing pathway (i.e., prodrug cellular uptake and/or cleavage) are most perturbed by these structural modifications.

### In Vivo Pharmacokinetics for Compounds **2a**, **16g**, and **16h**

Considering the data presented in Tables 2 and 3, our final candidates for in vivo studies (C57BL/6 mice) were **16g** and **16h** with **2a** as a control compound against TXL. Prior to submission, these prodrugs were evaluated in mouse liver microsomes (MLMs) and human and mouse plasma in vitro (Table 4) to assess whether the thioether linker would impart a distinct metabolic profile from our previously reported TXL analogs in mice.^39^ Just as with TXL, **2a** demonstrates a much higher metabolic stability in MLM (*t*_1/2_ > 120 min), most likely due to species differences in prodrug metabolizing CYP450 enzymes required for ω-oxidation. Additionally, **2a** was completely stable in human and mouse plasma (*t*_1/2_ > 240 min with 95% prodrug remaining at 240 min). Furthermore, similar trends were observed across human and mouse matrices for **16g** and **16h**, with exception to **16h** showing minimal decomposition in mouse plasma over the studied time course (71% prodrug remaining after 240 min). Since rodent plasma presents distinct esterase and hydrolase activity profiles compared to human plasma, **16h** decomposition within the mouse matrix may have emerged from an enzyme isoform not found in humans.^54^ Nevertheless, we moved forward with our PK analysis of these three prodrugs.

**Table 4:** In Vitro Stability Profiles for **2a**, **16g**, and **16h** in Human and Mouse Liver Microsomes and Plasma

compound	lipid motif	HLM *t*_1/2_ (min)^a^ [% remaining]^b^	MLM *t*_1/2_ (min)^a^ [% remaining]^b^	human plasma *t*_1/2_ (min)^a^ [% remaining]^c^	mouse plasma *t*_1/2_ (min)^a^ [% remaining]^c^
**2a**	C_3_H_6_–S-C_16_H_33_	41.7 ± 2.3	>120 [>95%]	>240 [>95%]	>240 [>95%]
**16g**	C_3_H_6_–S-C_12_H_24_-4-FPh	>120 [69%]	>120 [88%]	>240 [>95%]	>240 [>95%]
**16h**	C_3_H_6_–S-C_12_H_24_–C_6_H_11_	>120 [85%]	>120 [>95%]	>240 [>95%]	>240 [71%]^d^

a *n* = 2.

b Relative percent remaining of prodrug at 120 min.

c Relative percent remaining of prodrug at 240 min.

d Low LC–MS/MS signal intensity seen throughout assay time course.

To compare the in vivo PK profiles to TXL and our previously reported drug lead **1** (Tables S19 and S26),^39^ one group of male C57BL/6 mice were administered a single oral dose (p.o., 10 mg/kg) of **2a**, **16g**, or **16h** (refer to Experimental Methods). Additionally, a separate group of mice were given a single intravenous dose (i.v., 3 mg/kg) of each prodrug to obtain plasma clearance (*Cl*), volume of distribution (*V*_d_), and oral bioavailability data. Blood was sampled at 8 time points over 8 h (i.v.) or 24 h (p.o.) postdose, and plasma was isolated via centrifugation. Tissues (liver, kidney, and brain) were also harvested and homogenized at 3 time points over 24 h after oral dose. The concentrations of prodrug and TFV metabolite in each of these samples was then quantified using LC–MS/MS methodology.

**Figure 6:**
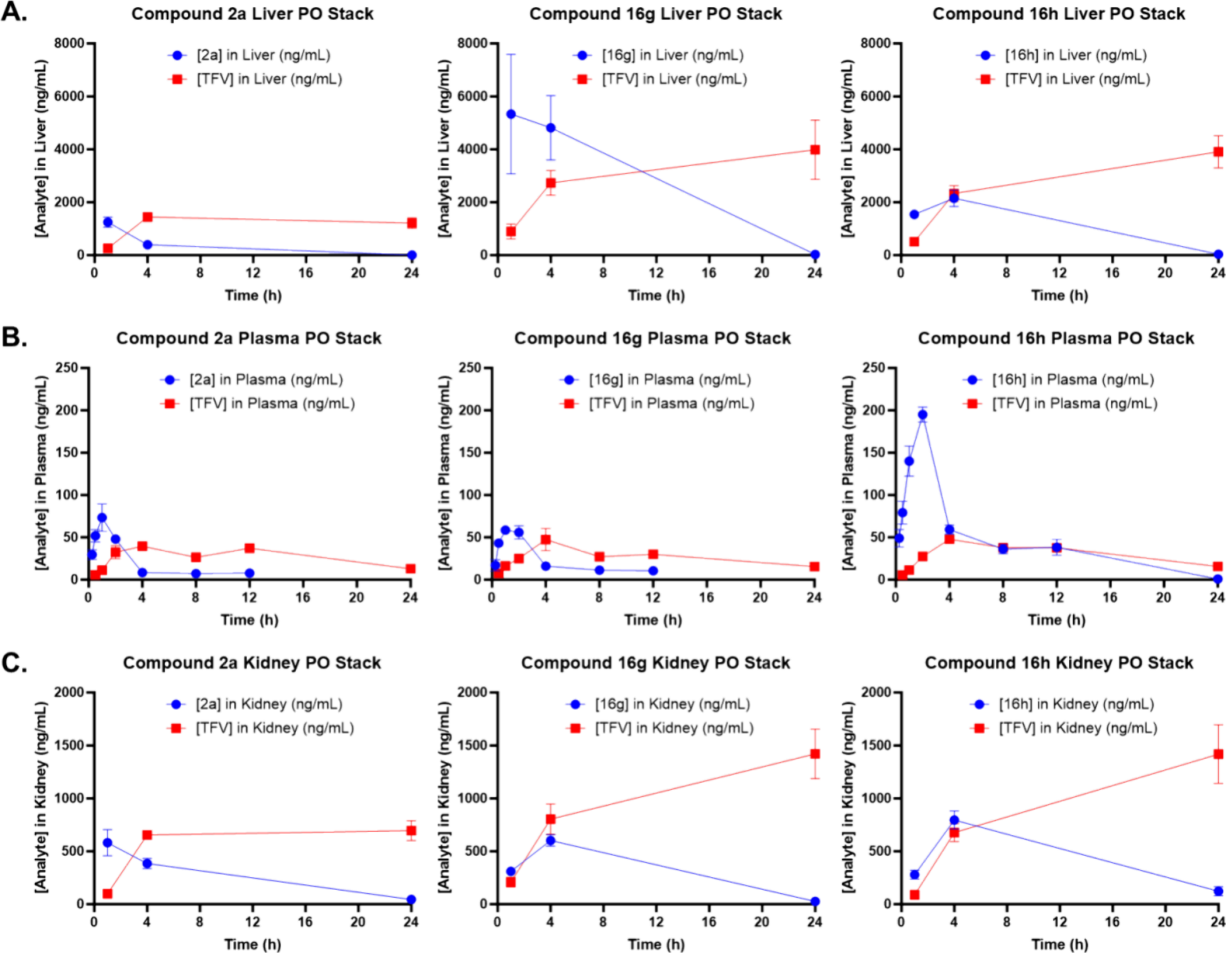
Mouse plasma and liver pharmacokinetic profiles of HTP-derived analogs of TXL. Male C57BL/6 mice (*n* = 3 per time point) were administered a single oral dose (10 mg/kg) of TFV prodrug using 90:10 olive oil:EtOH as a vehicle. Plasma, liver, and kidney levels of prodrug and TFV were quantified using LC–MS/MS. Liver (A), plasma (B), and kidney (C) concentrations of TFV prodrug and TFV metabolite are plotted together. Data represent the mean concentration at each time point ± SEM. The figures are generated with GraphPad Prism v9.

After oral dosing, compound **16g** achieved the highest hepatic exposure levels, as indicated by *C*_max_ and AUC_0–24h_, followed by **16h**, which demonstrated greater than a 4-fold elevated AUC_0–24h_ relative to **2a** (Figure 6A, Table 5). However, while TXL exhibited a comparable mouse liver profile to **2a**, analog **1** (Figure 4) achieved higher *C*_max_ (8710 ng/mL) and AUC_0–24h_ (109,000 h·ng/mL) values than each of the HTP-derived lipid prodrugs.^39^ Regrettably, as signified by TFV AUC_0–24h_, compounds **16g** and **16h** delivered significantly higher levels (greater than 2-fold) of TFV metabolite to the liver relative to **2a**, TXL, and **1** (TFV AUC_0–24h_ = 23000–29000 h·ng/mL). Additionally, while **16g** and **16h** achieved liver prodrug to TFV AUC_0–24h_ ratios that were 4- and 2-fold higher than TXL respectively, analog **1** solely displayed a ratio greater than 1 (Table S26). Considering that **16g** and **16h** were optimized against CYP450-mediated ω-oxidation alongside **1**, one potential explanation for the unexpected increase in hepatic TFV levels is that these two prodrugs could be effectively metabolized by phase I and/or II enzymes absent in mouse and human liver microsome matrices. Alternatively, seeing as all prodrugs studied released some degree of TFV in the liver, metabolite exposure may significantly depend on the compound’s ability to permeate species-specific hepatocytes and engage in PLC cleavage.^55−57^ Evidence for the latter was observed during in vitro cellular uptake and stability experiments conducted with HepaRG cells, which simulate primary human hepatocytes (results outlined below).

**Table 5:** Mouse Liver Pharmacokinetic Properties of Top HTP-Derived TFV Prodrugs After Oral Dose (10 mg/kg)

PK parameter (p.o.)	**2a** (CH_3_)	**16g** (4-FPh)	**16h** (C_6_H_11_)
*T*_max_ (h)	1.00	1.00	4.00
*C*_max_ (ng/mL)	1250	5330	2150
AUC_0–24h_ (h·ng/mL)	6450	63,600	27,300
TFVT_max_ (h)	4.00	24.0	24.0
TFVC_max_ (ng/mL)	1440	3980	3900
TFV AUC_0–24h_ (h·ng/mL)	29,100	72,500	66,600
Prodrug AUC_0–24h_/TFV AUC_0–24h_	0.222	0.877	0.411

Interestingly, following i.v. (Figures S43, S46, and S49, Table 6) and p.o. (Figure 6B, Table 7) administration, compound **16h** achieved the best plasma exposure levels among the thioether-lipid derivatives, as illustrated by the reported *C*_max_ and AUC_0–24h_. In fact, after oral dosing, prodrug **16h** demonstrated a plasma *C*_max_ (195 ng/mL) that surpassed all tested lipid moieties (Table S19) and a greater than 3-fold improvement in AUC_0–24h_ (1070 h·ng/mL) over TXL, **2a**, and **16g**. Moreover, the plasma AUC_0–24h_ value of **16h** was attractively comparable to that of our previous drug lead **1** (1280 h·ng/mL) and resulted in an impressive oral bioavailability (%*F*) of 54.9%. Surprisingly, compound **16g** showed a similar oral bioavailability to **2a** (overall %*F* of 20%), despite **16g** exhibiting significantly higher hepatic levels than **2a** and **16h**. Additionally notable was that the plasma exposure profile of TXL outperformed **16g** in oral experiments, raising questions about the PLC and membrane diffusion kinetics of **16g** in mouse hepatocytes. Next, prodrug **16h** displayed the lowest *Cl* (5.15 L/h/kg) and *V*_d_ (22.8 L/kg) of the HTP series in correlation with the significantly higher plasma exposure levels observed after oral administration. Analog **16h** also exhibited a plasma *t*_1/2_ (3.07 h) comparable to TXL (2.92 h) and **2a** (3.49 h), presumably due to compound’s propensity to engage with the liver and kidneys (refer to Table 8). Although **16g** exhibited the longest p.o. plasma *t*_1/2_ (4.19 h) among the three congeners, surpassing TXL but being 2-fold shorter than that of compound **1** (8.66 h), relatively poor linear regression (0.65 ≤ *R*^2^ ≤ 0.77) of first-order kinetic plots suggest that **2a** and **16g** undergo nonlinear pharmacokinetic elimination just like TXL (refer to Supporting Information). As *t*_1/2_ and *V*_d_ values are calculated from the slopes (*k*) of these linear fits, these parameters should be considered approximations as opposed to absolute values. In contrast, **16h** and **1** appear to nicely undergo first-order pharmacokinetic elimination (0.94 ≤ *R*^2^ ≤ 0.96), thereby placing higher confidence on *t*_1/2_ and *V*_d_ values for these compounds. Nevertheless, further studies are necessary to elucidate which prodrug properties are responsible for providing the distinct plasma PK profile observed with **16h** relative to **2a** and **16g**. Based on our initial in vitro stability evaluation of **16h** in mouse plasma, the prodrug’s poor matrix solubility or increased protein binding compared to the other HTP derivatives may play a role.

**Table 6:** Mouse Plasma Pharmacokinetic Properties of Top HTP-Derived TFV Prodrugs After Intravenous Dose (3 mg/kg)

PK parameter (p.o.)	**2a** (CH_3_)	**16g** (4-FPh)	**16h** (C_6_H_11_)
*T*_max_ (h)	0.10	0.10	0.10
*C*_max_ (ng/mL)	2060	863	2380
AUC_0–8h_ (h·ng/mL)	340	373	583
*k*	0.467	0.406	0.574
*R*^2^	0.85	0.93	0.91
*t*_1/2_ (h)	1.49	1.71	1.21
*Cl*(L/h/kg)	8.82	8.05	5.15
*V*_d_(L/kg)	18.9	19.9	8.97

**Table 7:** Mouse Plasma Pharmacokinetic Properties of Top HTP-Derived TFV Prodrugs After Oral Dose (10 mg/kg)

PK parameter (p.o.)	**2a** (CH_3_)	**16g** (4-FPh)	**16h** (C_6_H_11_)
*T*_max_ (h)	1.00	1.00	2.00
*C*_max_ (ng/mL)	73.3	58.6	195
AUC_0–24h_ (h·ng/mL)	219	260	1070
%*F*	19.3	20.9	**54.9**
*k*	0.199	0.165	0.226
*R*^2^	0.65	0.77	0.94
*t*_1/2_ (h)	3.49	4.19	3.07
*Cl*(L/h/kg)	8.82	8.05	5.15
*V*_d_(L/kg)	44.4	48.7	22.8
TFVT_max_ (h)	4.00	4.00	4.00
TFVC_max_ (ng/mL)	39.4	47.4	47.8
TFV AUC_0–24h_ (h·ng/mL)	657	635	740
Prodrug AUC_0–24h_/TFV AUC_0–24h_	0.333	0.409	1.44

**Table 8:** Mouse Kidney and Brain Pharmacokinetic Properties of Top HTP-Derived TFV Prodrugs After Oral Dose (10 mg/kg)

tissue	PK parameter (p.o.)	**2a** (CH_3_)	**16g** (4-FPh)	**16h** (C_6_H_11_)
kidney	*T*_max_ (h)	1.00	4.00	4.00
	*C*_max_ (ng/mL)	581	602	795
	AUC_0–24h_ (h·ng/mL)	5740	7650	10,800
	TFVT_max_ (h)	4.00	24.0	24.0
	TFVC_max_ (ng/mL)	695	1420	1420
	TFV AUC_0–24h_ (h·ng/mL)	14,600	23,800	22,100
	Prodrug AUC_0–24h_/TFV AUC_0–24h_	0.393	0.322	0.488
brain	*T*_max_ (h)	1.00	4.00	1.00
	*C*_max_ (ng/mL)	43.8	5.74	16.2
	AUC_0–24h_ (h·ng/mL)	69.0	13.5	36.8
	TFVT_max_ (h)			
	TFVC_max_ (ng/mL)			
	TFV AUC_0–24h_ (h·ng/mL)			

Furthermore, despite **16h** delivering the highest plasma TFV *C*_max_ and AUC_0–24h_ after oral dose, the ratio of **16h** AUC_0–24h_ to TFV AUC_0–24h_ was most attractive compared to **2a** and **16g**, both of which exhibited ratios below 1 (Table 7). However, TXL and **1** delivered lower plasma TFV *C*_max_ and AUC_0–24h_ values and demonstrated higher AUC_0–24h_ ratios than each of the HTP-derived lipid prodrugs evaluated herein. Once more, considering that **2a**, **16g**, and **16h** displayed high in vitro mouse plasma stability (*t*_1/2_ > 4 h), the mechanism by which each prodrug released such a high degree of TFV in vivo (5-fold greater AUC_0–24h_ versus TXL) remains unclear. Additionally, while the hepatic TFV levels generated from **16g** and **16h** may have led to increased plasma exposure in relation to TXL and **1**, the same does not apply to **2a**, which possessed a similar hepatic profile to TXL.

Moreover, we ventured to further elucidate the distribution profile for the 3 HTP-derived lipid prodrugs by specifically analyzing the kidney and brain, which are relevant due to TFV-mediated nephrotoxicity and HIV reservoirs in the CNS (Figure 6C, Table 8). After oral administration, compound **16h** achieved the highest renal exposure levels, followed by **16g** and **2a**. Interestingly, while **16g** demonstrated an 8-fold higher AUC_0–24h_ in the liver versus kidney, **16h** and **2a** displayed significantly lower liver to kidney AUC_0–24h_ ratios. Considering that glomerular filtration in the kidneys is negatively impacted by protein binding, lipophilicity, and molecular size and HDP prodrugs historically exhibit very low renal uptake,^13^ the prodrug exposure levels for **16h** and **2a** were unexpected. Additionally, based on the liver and plasma PK results outlined above, prodrugs **16g** and **16h** unsurprisingly delivered the highest TFV *C*_max_ and AUC_0–24h_, whereas **2a** yielded the lowest renal exposure levels of TFV. Regrettably, all compounds displayed prodrug to TFV AUC_0–24h_ ratios below 1, posing nephrotoxic implications over long-term use. Finally, in contrast to exposure levels in liver and kidney, the 3 HTP-derived lipid prodrugs achieved very low prodrug *C*_max_ and AUC_0–24h_ values in brain tissue, and none of them delivered detectable quantities of TFV, at least not at the time points evaluated. It is worth noting however that we did not measure levels of TFV monophosphate or diphosphate, and therefore, additional experiments are required to draw strong conclusions about CNS penetration. Overall, **2a** achieved the highest prodrug exposure levels, followed by **16h** then **16g**. Based on these results, we are currently conducting experiments in brain homogenate to evaluate whether HDP and HTP prodrug strategies have utility for targeting latent HIV in the CNS.

### In Vitro Cellular Uptake and Stability Evaluation For Select HTP-Derived Prodrugs In HepaRG Cells

**Figure 7:**
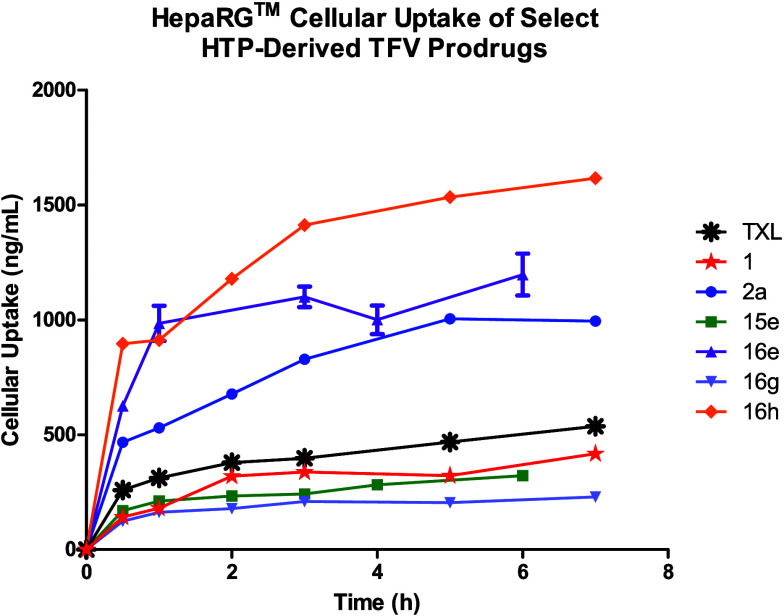
HepaRG cellular uptake profiles of select HTP-derived analogs of TXL. Data represent the mean concentration at each time point ± SD. The figure was generated with GraphPad Prism v9.

Between the in vitro HLM (Tables 1–4) and in vivo mouse liver PK (Tables 5 and S26) data sets presented above, we discovered certain discrepancies in the pharmacological profiles of our HTP-derived TFV prodrugs that warranted further investigation. For example, compound **16g** unexpectedly demonstrated a 2-fold higher in vivo hepatic AUC_0–24h_ than **16h**, despite the 4-fluorophenyl lipid moiety potentially having an order of magnitude lower lipophilicity versus its cyclohexyl counterpart according to predictions (Table S41). Furthermore, while HDP and trifluoromethyl lipid derivatives are anticipated to possess similar clogP values, analog **1** displayed a nearly 22-fold increase in hepatic AUC_0–24_ in vivo over TXL. Lastly, contrary to our optimization efforts in HLM in vitro, compounds **16g** and **16h** exhibited 3-fold greater TFV AUC_0–24h_ levels compared to TXL and prodrug **1**. Such inconsistencies pose challenges for the translation of in vitro data obtained in human matrices toward mouse PK models. Hence, we utilized an in vitro HepaRG cell-based assay to evaluate the pharmacological behavior of our best prodrugs **16g** and **16h**, along with TXL, **1**, and **2a** as control compounds, in a more metabolically complete, human-related system. Additionally, we spiked the cells with prodrugs **15e** and **16e** to provide insight into the relationship between cellular drug delivery and sterics at the lipid terminus. Plated HepaRG cells were employed for this purpose since they retain many of the characteristics found in primary human hepatocytes without compromising CYP450 activity, including non-CYP450 and phase II enzymes, active transport mechanisms, tight cellular junctions, and fatty acid catabolic pathways.^58−60^ Naturally, due the metabolic complexity of HepaRG cells, we specifically analyzed prodrug cellular uptake and stability, as represented by *C*_max_ and intrinsic clearance (*Cl*_int,*u*_) accordingly (Figure 7 and Table 9). Furthermore, the in vitro parameters described in Table 9 were obtained using identical compound formulation (5:1 drug:HSA) and LC-MS/MS method development as outlined above (refer to Experimental Methods).

**Table 9:** In Vitro HepaRG Uptake and Stability Profiles For Select Lipid-Based TFV Prodrugs

compound	lipid motif	HepaRG *C*_max_ (ng/mL)^a^^,^^b^	HepaRG *t*_1/2_ (min)^a^	HepaRG *Cl*_int*,u*_ (μL/min/million cells)^c^
**TXL**	C_3_H_6_–O-C_16_H_33_	397.1 ± 1.4	44.3 ± 0.5	28.5
**1**	C_3_H_6_–O-C_15_H_31_–CF_3_	337.4 ± 1.0	42.9 ± 0.5	29.4
**2a**	C_3_H_6_–S-C_16_H_33_	828.0 ± 0.2	89.4 ± 0.4	14.1
**15e**	C_3_H_6_–S-C_12_H_24_–C≡C*t*-Bu	242.0 ± 5.5	66.6 ± 5.0	18.9
**16e**	C_3_H_6_–S-C_14_H_28_-*t*-Bu	1100.0 ± 63.6	123.1 ± 3.0	10.2
**16g**	C_3_H_6_–S-C_12_H_24_-4-FPh	209.8 ± 0.2	46.7 ± 0.3	27.0
**16h**	C_3_H_6_–S-C_12_H_24_–C_6_H_11_	1412.4 ± 1.5	71.0 ± 0.4	17.8

a *n* = 2.

b *C*_max_ value that was recorded after 3 h incubation.

c *Cl*_int,*u*_ value was calculated from cellular *t*_1/2_ (refer to Experimental Methods for equation). Linear atom # does not count H or F.

Contrary to the mouse hepatocyte data described above,^39^ TXL and compound **1** demonstrated comparable HepaRG uptake with *C*_max_ values of 397 ng/mL and 337 ng/mL after 3 h incubation, respectively. Moreover, despite displaying a substantially longer *t*_*1/2*_ in HLM and a 25-fold higher prodrug to TFV AUC_0–24h_ ratio in mouse liver, prodrug **1** showed a nearly identical rate of cellular clearance to TXL (*t*_1/2_ = 43–44 min, *Cl*_int,*u*_ = ∼ 29 μL/min/million cells). These results suggest drastic differences in PLC cleavage and/or membrane diffusion kinetics across cell lines. Next, prodrug **2a** exhibited greater than 2-fold higher cellular uptake (*C*_max_ = 828 ng/mL) relative to TXL and **1**, indicating that HepaRG permeability improves with increasing lipophilicity. Further evidence for this trend was observed with cyclohexyl derivative **16h** demonstrating an impressive HepaRG *C*_max_ value of 1410 ng/mL, followed by *t*-Bu moiety **16e** as the next best permeating prodrug (*C*_max_ = 1100 ng/mL). Consequently, due to the heightened cellular prodrug levels observed with **2a**, **16e**, and **16h**, these compounds displayed lower *Cl*_int*,u*_ values (10–18 μL/min/million cells) than TXL and **1**. However, despite delivering the largest drug payload into HepaRG cells, compound **16h** exhibited a faster rate of clearance than both **2a** and **16e**, potentially suggesting that the cyclohexyl lipid moiety is a more favorable PLC substrate under these biological conditions. On the other hand, prodrug **16e** markedly showed the slowest rate of cellular clearance (*t*_1/2_ = 123 min, *Cl*_int,*u*_ = 10.2 μL/min/million cells), indicating that the *t*-Bu lipid derivative undergoes inefficient cell processing and may resist cleavage to TFV. Interestingly, in direct contrast to mouse liver PK experiments, compound **16g** displayed the lowest cellular uptake with a *C*_max_ value of 210 ng/mL, a nearly 4-fold and 7-fold reduction compared to **2a** and **16h**, respectively. Hence, due to the decreased cellular prodrug levels, analog **16g** demonstrated a clearance rate analogous to TXL and **1** (*t*_1/2_ = 47 min, *Cl*_int,*u*_ = 27 μL/min/million cells). Lastly, *t*-Bu acetylene **15e** showed a greater than 4-fold decrease in cell uptake (*C*_max_ = 242 ng/mL) versus counterpart **16e**, hinting at a relationship between structural conformation at the lipid terminus and HepaRG membrane diffusion efficiency that requires further study. Additionally notable is that although **15e** exhibited nearly a 6-fold lower cellular uptake efficiency compared to **16h**, these compounds displayed nearly equivalent rates of cellular clearance (*t*_1/2_ = 67–71 min, *Cl*_int,*u*_ = 18–19 μL/min/million cells). Akin to **16e**, this result suggests that our *t*-Bu terminally capped lipids are poorly processed by cells.

Overall, we discovered key data set differences while evaluating lipid-based TFV prodrugs with in vitro HepaRG assays and in vivo mouse liver PK experiments. Particularly, drastic variances in the pharmacological parameters of TXL, **1**, **2a**, **16g**, and **16h** raise questions about how well data obtained from in vitro human-related systems translates to in vivo mouse models. We are currently analyzing the behavior of these prodrug constructs in a series of species-specific hepatocytes and PLC isozymes that will be reported in the future. Nonetheless, on a preliminary level, the compilation of in vitro and in vivo results presented here demonstrates the significance of fatty acid catabolic pathways and membrane diffusion kinetics in each cell line have on prodrug delivery, cleavage, and distribution for HIV treatment.

## Conclusions

Herein, we describe the strategies, synthesis, and pharmacological assessment of an HTP-derived prodrug series of TFV (Figure 4). In consideration of the promising in vitro characteristics of **2a**, we performed systematic thioether position, chain length, and lipid terminus optimizations to identify the most therapeutically effective and metabolically stable TFV prodrugs. As demonstrated in Table 1, analogs bearing thioether functionality beyond the δ position (**2c**–**2f**) or lipid chain lengths below 20 atoms (**2g** and **2h**) exhibited considerable reductions in HLM stability and/or anti-HIV activity in vitro. Interestingly, prodrugs carrying lipids over 20 atoms (**2i** and **2j**) showed significantly enhanced HLM resistance versus TXL and **2a** but could not be properly assessed for antiviral potency due to poor solubility in assay media. Accordingly, we opted to employ a terminal group SAR study using the general lipid characteristics of **2a** (i.e., 20 atom lipids with sulfur in δ position), coupled with functional groups designed to block CYP450-mediated ω-oxidation (Tables 2 and 3). While our investigation resulted in the synthesis of several prodrugs with substantially longer *t*_1/2_ in HLM than TXL and **2a,** dramatic variability in anti-HIV activity was observed among structurally similar congeners (e.g., **16e** vs **16h**). Ultimately, compounds **16g** and **16h** passed our initial in vitro screening requirements, and hence were subjected to in vivo mouse PK experiments, alongside **2a** as a control (Tables 5–8). Regrettably, while **16h** arguably demonstrated the best PK profile in vivo with an oral bioavailability of 55% after a 10 mg/kg dose, we observed significant levels of TFV in mouse liver, plasma, and kidney for all HTP-based compounds studied. Additionally, the HTP-derived TFV prodrugs were unable to surpass our previously discovered drug lead **1** in terms of mouse plasma *t*_1/2_ and overall prodrug to TFV AUC_0–24h_ ratio. Motivated by discrepancies found between in vitro HLM and in vivo mouse liver PK experiments, we analyzed select lipid-based TFV prodrugs in HepaRG uptake and stability assays (Table 9) to simulate an advanced human-related metabolic system. After discovering significant deviation between the in vitro and in vivo uptake and clearance profiles of our prodrugs, we determined that careful animal model selection will be necessitated for future studies. Nevertheless, the preliminary results presented in this report demonstrate that our lead **16h** is significantly absorbed and prematurely processed by the liver, which is somewhat inconsistent with our optimization efforts in HLM. Thus, further prodrug optimization using more physiologically relevant systems is required to achieve more attractive distribution and stability in vivo. Toward this aim, our lab is currently experimenting with longer chain length lipids featuring elements of unsaturation that will be reported soon.

## Experimental Methods

### Chemistry

Automated flash column chromatography was performed using a Teledyne ISCO CombiFlash Companion system with RediSep Rf normal-phase silica gel-packed columns or RediSep Rf Gold reverse-phase C18 columns (Teledyne Isco). Melting/decomposition ranges were determined on a REACH Devices RD-MP digital melting point apparatus. Analytical thin-layer chromatography (TLC) was carried out on commercially available (Sigma) aluminum-supported (thickness: 200 μm) or glass (2.5 × 7.5 cm) silica gel plates with fluorescent indicator (F-254). Visualization of compounds on TLC plates was achieved using UV light (254 nm) and/or using ethanolic phosphomolybdic acid (PMA) or aqueous potassium permanganate (KMnO_4_) solutions. TLC retention factors (*R*_f_) were determined on glass (2.5 × 7.5 cm) silica gel plates with fluorescent indicator (F-254) and calculated as the average of three replicate experiments. NMR spectra (^1^H, ^13^C, and ^31^P) were acquired using a Bruker Ascend 600 MHz spectrometer, a Varian INOVA 600 MHz spectrometer, a Varian INOVA 500 MHz spectrometer, a Bruker NEO 400 MHz spectrometer, a Varian INOVA 400 MHz spectrometer, or a Varian VNMR 400 MHz spectrometer (Emory University NMR Center, directed by Dr. Shaoxiong Wu). NMR samples were prepared in deuterated chloroform (CDCl_3_) or deuterated methanol (CD_3_OD) using tetramethylsilane (TMS) or residual solvent peaks (CDCl_3_: ^1^H = 7.26 ppm, ^13^C = 77.16 ppm; CD_3_OD: ^1^H = 3.31 ppm, ^13^C = 49.00 ppm; TMS: ^1^H = 0.00 ppm) as internal references. Alternatively, the residual chloroform peak in ^1^H NMR was used as an absolute reference for ^31^P NMR, unless otherwise specified. MestreNova software was used to process all NMR spectra. NMR data includes chemical shifts (δ) reported in ppm, multiplicities indicated as s (singlet), d (doublet), t (triplet), q (quartet), dd (doublet of doublets), dt (doublet of triplets), td (triplet of doublets), m (multiplet), br (broad), or app (apparent), and coupling constants (*J*) reported in Hz. High resolution mass spectrometry (HRMS) was performed by the Emory University Mass Spectrometry Center, directed by Dr. Fred Strobel. Liquid chromatography–mass spectrometry (LC–MS) was performed on an Agilent 1200 HPLC equipped with a 6120 Quadrupole mass spectrometer (ESI) eluting with mixtures of HPLC grade MeOH and H_2_O or MeCN and H_2_O (all spiked with 0.1% HCO_2_H) through an analytical, reverse-phase Agilent InfinityLab Poroshell 120 EC-C8 (2.1 mm × 50 mm, 2.7 μm) column. LC-MS samples were prepared with HPLC grade MeOH at a concentration of 1 mg/mL. Final compound purity was assessed using LC-MS, and purity of all final compounds reported herein were determined to be ≥95% pure. The chemical synthesis and characterization for all compounds can be found in the Supporting Information.

### Cellular Toxicity and Antiviral Activity Assays

Assessment of cytotoxicity and antiviral activity of all final compounds in vitro was conducted according to previously reported procedures.^39^ In summary, human embryonic kidney (HEK293T) cells maintained in high glucose (25 mM), Dulbecco’s modified Eagle’s medium (DMEM) supplemented with fetal bovine serum (10%), sodium pyruvate (1 mM), l-glutamine (2 mM), HEPES (25 mM), and gentamicin (50 μg/mL) were used for determinations. The cells were maintained in an incubator at 37 °C under 5% CO_2_ in a humidified atmosphere. For both cytotoxicity and antiviral screening, all compounds were evaluated in duplicate in at least two independent experiments. All final compounds were formulated with human serum albumin (HSA, 5:1 compound to HSA molar ratio) and subsequently diluted with complete DMEM to 200 μM. Fifty microliters of test compound serial dilutions (3-fold) were prepared in 96-well culture plates, after which 50 μL of HEK293T cells (2 × 10^4^ cells/well) was added. Final test compound concentrations for toxicity assessments ranged from 100 μM to 1.7 nM. A growth medium control without test compound was included as an indicator of 100% cell viability (no cytotoxicity). Ninety-six-well plates were incubated for 48 h at 37 °C under 5% CO_2_ in a humidified atmosphere. Cytotoxicity was then assessed by quantifying cell viability using the CellTiter 96 Aqueous One Solution Cell Proliferation Assay (Promega, Madison, WI) or resazurin sodium salt (cat# R7017, Merck, Darmstadt, Germany).^61^ The concentration of test compound that kills 50% of cultured cells (CC_50_) was calculated using Microsoft Excel (Redmond, WA). Antiviral activity was then assessed using a single-cycle, nonreplicating, envelope deleted HIV pseudoviral in vitro assay.^62,63^ The assay relies on the introduction of the firefly luciferase gene into HEK293T cells through infection with HIV-like viral particles, containing Reverse Transcriptase (RT) and Integrase (IN) from the HIV-1 subtype C strain MJ4.^64^ The expression of the luciferase in the cells is directly proportional to the level of infection by the HIV-like viral particles and can be used to assess the inhibition of HIV-1 RT and IN. To evaluate anti-HIV activity, 3-fold serial dilutions of all final compounds were prepared in 50 μL over the noncytotoxic concentration range (as determined from the cytotoxicity screens described above) in 96-well culture plates. HEK293T cells (2 × 10^4^ cells/well) and HIV-like viral particles, standardized to produce a luminescence signal of 1 × 10^6^ relative light units (RLUs) in the growth medium only control, were combined, and 50 μL was added to the wells containing serially diluted test compounds. A growth medium control without test compound was included as a reference for 100% viral activity (no inhibition). Ninety-six-well plates were incubated for 48 h at 37 °C under 5% CO_2_ in a humidified atmosphere. The expression of luciferase was subsequently quantified by adding 100 μL of the Bright-Glo Luciferase Assay substrate (Promega, Madison, WI) to each well of the 96-well plates. After incubation at rt for 3 min, luminescence was quantified on the GloMax Explorer Multimode Microplate Reader (Promega, Madison, WI). The concentration of each compound required to inhibit viral activity by 50% (IC_50_) was calculated using Microsoft Excel (Redmond, WA).

### Metabolic Stability Assays

#### Liver Microsome Stability

Human liver microsomes (HLMs, 20 mg/mL) and CD-1 mouse liver microsomes (MLMs, 20 mg/mL) were purchased from Xenotech. NADPH was purchased from Sigma-Aldrich and prepared in 10 mM stock solutions of distilled H_2_O (Invitrogen UltraPure). Verapamil and diphenhydramine were both purchased from Sigma-Aldrich and served as positive controls for HLM and MLM stability, respectively. Test compounds and positive controls were initially dissolved in MeOH to make 10 mM stock solutions. Sample solutions were then further diluted in 70/30 MeOH/H_2_O to 500 μM. Next, the reactions were prepared by mixing human or mouse liver microsomes (55 μL) with potassium phosphate buffer (100 mM, 928 μL) in 1.5 mL Eppendorf tubes. The test compounds (6.6 μL of 500 μM solution) were subsequently added to the suspensions, and the reaction mixtures were incubated at 37 °C for 5 min. Afterward, the liver microsome reactions were initiated with 110 μL of 10 mM NADPH and further incubated at 37 °C for the designated time course of the study. This procedure provided experiments with a final volume of 1100 μL (<0.2% organic solvent content), a concentration for HLMs and MLMs of 1 mg/mL, and a final test compound concentration of 3 μM. Aliquots (100 μL) were removed from each reaction mixture in duplicate at 0, 15, 30, 60, and 120 min time intervals and quenched with 100 μL of cold internal standard solution (ISTD, 2 μM 7-ethoxy-*d*_*5*_-coumarin in MeOH). Quenched aliquots were then centrifuged at 12,500 g for 5–10 min, and the resulting supernatant solutions were withdrawn and placed in LC–MS vials to be analyzed by LC–MS/MS (Agilent G6460C QQQ MS coupled with an Infinity II 1260 HPLC). Each test compound was run in tandem with positive and negative control experiments for quality assurance. Positive control reactions were conducted at a final volume of 550 μL for a single run of each time point. Lastly, the negative control experiment was conducted with test compounds and liver microsomes in the absence of NADPH (150 μL) and analyzed at the 120 min time point.

#### Plasma Stability

Human plasma (lithium heparin (LiHep) mixed, gender pooled, 0.2 μm filtered) and mouse plasma (BALB/C, LiHep mixed, male pooled, 0.2 μm filtered) were purchased from BioIVT. Procaine (Sigma-Aldrich) served as a positive control for both human and mouse plasma experiments. Test compounds and positive controls were initially dissolved in MeOH to make 10 mM stock solutions. The solutions of test and control compounds were then further diluted in 70/30 MeOH/H_2_O to 500 μM. Next, the human or mouse plasma (994 μL) was aliquoted into 1.5 mL Eppendorf tubes with duplicates (reactions A and B) being prepared for each compound. The plasma was then incubated at 37 °C for 10 min. Afterward, the reaction was initiated by the addition of test compound (6 μL of 500 μM solution) and further incubated at 37 °C for the designated time course of the study. This procedure provided duplicate experiments with a final volume of 1000 μL (<0.2% organic solvent content) and a final test compound concentration of 3 μM. Aliquots (100 μL) were removed from each reaction mixture at 0, 30, 60, 120, and 240 min time intervals and quenched with 150 μL of cold ISTD solution (2 μM 7-ethoxy-*d*_*5*_-coumarin in MeOH). Quenched aliquots were then centrifuged at 15,000*g* for 30–45 min, and the resulting supernatant solutions (∼70 μL) were withdrawn and placed in LC–MS vials to be analyzed by LC–MS/MS (Agilent G6460C QQQ MS coupled with an Infinity II 1260 HPLC). Each test compound was run in tandem with positive and negative control experiments for quality assurance. The positive control reaction was conducted at a final volume of 1000 μL for a single run of each time point. Finally, the negative control experiment was conducted with test compounds in Dulbecco’s phosphate buffered saline without calcium and magnesium (DPBS, Fisher Scientific, 143 μL) and analyzed at the 240 min time point.

#### Data Analysis

For both LM and plasma stability assays, each data point was analyzed in duplicate using in-between blank washes to avoid carry over and to equilibrate the column for the subsequent runs. Averages of these duplicate for individual compounds at each time point were then normalized to the data at 0 min, representing 100% test compound remaining or 0% metabolism. Half-lives (*t*_1/2_) were calculated by plotting ln of % test compound remaining versus time and performing linear regression to determine slope. Slope = −*k* and *t*_1/2_ = 0.693/*k* for first-order kinetics (Figures S1–S34).

### HepaRG Cell Uptake and Metabolic Stability

The HepaRG cell line (HPRGC10, Thermo Fisher Scientific) was used to evaluate the cellular permeability and metabolic stability for select final compounds in vitro. The cells were initially thawed with working media composed of HepaRG Thaw, Plate, & General Purpose Medium Supplement (HPRG670), Williams Medium E (100 mL), and GlutaMax (1 mL), all purchased from Thermo Fisher Scientific. Next, the cells were seeded in sterile collagen-coated 24-well plates (0.55 × 10^6^ cells/well) and maintained in an incubator at 37 °C under 5% CO_2_ in a humidified atmosphere for 24 h. After the thawing medium was aspirated from each well, the cells were cultured for an additional 6 days with working media consisting of HepaRG Maintenance/Metabolism Medium Supplement (HPRG720), Williams Medium E (500 mL), and GlutaMax (1 mL), all purchased from Thermo Fisher Scientific. Upon completing the 7-day culture period, HepaRG cells were plated in enough wells to allow for duplicate analysis of prodrugs in two independent cellular uptake or metabolic stability experiments. All studied compounds were formulated with human serum albumin (HSA, 5:1 compound to HSA molar ratio) and subsequently diluted to a 20 μM final concentration with assay medium composed of serum-free INVITROGRO HI Medium and TORPEDO Antibiotic Mix purchased from BioIVT. For cellular uptake determinations, the maintenance/metabolism medium was first aspirated from each well, and then the plated cells were spiked with 1.0 mL of test compound stock solution. Subsequently, the plate was incubated at 37 °C under 5% CO_2_ environment over the desired time course (seven data points/compound). Blank wells not treated with prodrug were included in assay runs as controls and initial time points (0 min). At each time point, the test compound solution was removed from the appropriate wells, and the plated cells were washed with 1.0 mL of DPBS twice. The HepaRG cells were then extracted with 500 μL of cold ISTD solution (1 μM 7-ethoxy-*d*_*5*_-coumarin in 70/30 MeOH/H_2_O), and the quenched aliquots were centrifuged at 15,000 g for 10 min at 4 °C. The resulting supernatant solutions were withdrawn and placed in LC–MS vials to be analyzed by LC–MS/MS (Agilent G6460C QQQ MS coupled with an Infinity II 1260 HPLC). After obtaining prodrug *C*_max_ and *T*_max_ values from the cellular uptake assay, HepaRG metabolic stability was then assessed. Following the initial 7-day culture procedure, the maintenance/metabolism medium was aspirated from each well, and the plated cells were spiked with 1.0 mL of test compound stock solution. Next, the plate was incubated at 37 °C under 5% CO_2_ atmosphere until the calculated *T*_max_ was reached (3 h). The test compound solution was then removed from each well and replaced with untreated dilution medium (i.e., serum-free INVITROGRO HI Medium and TORPEDO Antibiotic Mix), initializing the stability determination. The HepaRG plate was once more incubated at 37 °C under 5% CO_2_ atmosphere over the desired time course (nine data points/compound), and sample preparation and analysis were handled in identical fashion to cellular uptake experiments.

#### Data Analysis

Concentrations of lipid-based TFV prodrugs were determined using a fit-for-purpose LC-MS/MS method (see Tables S2–S8). Analyses of these data were conducted at Emory University according to the following specifications. *C*_max_ represents the mean observed maximum concentration achieved by prodrug in HepaRG cells. Similarly, *T*_max_ represents the average time at which *C*_max_ was observed. Metabolic half-lives (*t*_1/2_) were then calculated using methodology from LM and plasma stability assays described above (Figures S36–S42). The unscaled intrinsic clearance was determined using the equation: *Cl*_int,*u*_ = (0.693/*t*_1/2_) × (1000/0.55) μL/min/million cells.

### Mouse Pharmacokinetic Experiments (Conducted by Sai Life Sciences)

#### Test System

Healthy male C57BL/6 mice (8–12 weeks old) weighing 20–30 g were procured from Global, India. Three mice were housed in each cage. Temperature and humidity were maintained at 22 ± 3 °C and 30–70%, respectively, and illumination was controlled to provide for 12 h light and 12 h dark cycles. Temperature and humidity were recorded by an autocontrolled data logger system. All animals were provided a laboratory rodent diet (Envigo Research private Ltd., Hyderabad). Reverse osmosis H_2_O treated with ultraviolet light was provided ad libitum.

#### Study Design

Male mice were divided into two groups (*n* = 18 total, *n* = 9 per treatment group, *n* = 3 per time point, sparse sampling). One group was intravenously administered a solution of **2a**, **16g**, and **16h** in 10% PEG-300, 10% Solutol HS-15, and 80% saline at 3 mg/kg with a dosing volume of 5 mL/kg. The second group was administered a solution of **2a**, **16g**, and **16h** in 10% EtOH and 90% olive oil at 10 mg/kg via oral gavage with a dosing volume of 10 mL/kg.

#### Sample Collection

Blood samples (approximately 60 μL) were collected from retro-orbital plexus under light isoflurane anesthesia such that the samples were obtained predose, as well as at eight time points post dose: 0.1, 0.25, 0.5, 1, 2, 4, 6, and 8 (i.v.) and 0.25, 0.5, 1, 2, 4, 8, 12, and 24 h (p.o.). Immediately after blood collection, plasma samples were harvested by centrifugation at 4000 rpm and 4 °C for 10 min and stored at −70 °C until LC–MS/MS analysis. Following blood collection from p.o. group, liver, kidney, and brain samples were collected (*n* = 3 per treatment group per time point) at 1, 4, and 24 h post dose. After isolation, tissue samples were rinsed three times with ice-cold saline and dried with blotting paper. Next, the tissue samples were homogenized using ice-cold PBS (pH = 7.4), and the resulting homogenates were stored below −70 °C until LC-MS/MS analysis. The total homogenate volume was 3 times the brain weight and 5 times the liver and kidney weight.

#### Data Analysis

Concentrations of **2a**, **16g**, and **16h** were determined using a fit-for-purpose LC–MS/MS method by Sai Life Sciences (see Tables S9–S40). Analyses of these data were conducted at Emory University according to the following specifications. *C*_max_ represents the mean observed maximum concentration achieved by prodrug (**2a**, **16g**, and **16h**) and common metabolite TFV in plasma or tissue. Similarly, *T*_max_ represents the average time at which *C*_max_ was observed. Areas under the concentration–time curves (AUC_0–8h_ and AUC_0–24h_) were calculated via the linear trapezoidal rule^65^ using GraphPad Prism v9. Furthermore, terminal elimination rate constants k were determined using linear regression (slope = −*k*) of the terminal portion of semilog-transformed concentration–time curves (Figures S44, S45, S47, S48, S50, and S51). Half-lives (*t*_1/2_) were then estimated using the first-order kinetics equation: *t*_1/2_ = 0.693/*k*. After i.v. or p.o. dosing, plasma clearance was calculated using the equations: *Cl*_*i.v*._ = *V*_d_ × *k* = Dose_i.v._/AUC_0–8h_ and *Cl*_*p.o*._ = *V*_*d*_ × k = Dose/AUC_0–24h_. The oral bioavailability for each prodrug was calculated using the equation: %F = [(AUC_0–24h_ × Dose(i.v.))/(AUC_0–8h_ × Dose(p.o.))] × 100.

#### Ethical Declaration

These studies were performed with approval from the Institutional Animal Ethics Committee (IAEC) in accordance with the requirements of and the guidelines provided by The Committee for the Purpose of Control and Supervision of Experiments on Animals (CPCSEA, India), as published in The Gazette of India, December 15, 1998.

## Supporting Information Available

Protocols for and data from in vitro bioassays (metabolic stability and cellular uptake and stability assays), mouse PK experiments, synthetic procedures for all intermediates and final compounds, ^1^H and ^13^C NMRs for thioether-containing lipids and prodrugs, and LC–MS traces of all compounds tested in vivo (PDF)

## Abbreviations

ANP: acyclic nucleoside phosphonate
AUC: area under the curve
cART: combination antiretroviral therapies
CES1: carboxyesterase I
*Cl*: clearance
CYP450: cytochrome P450
DBU: 1,8-diazabicyclo[5.4.0]undec-7-ene
DCC: *N,N*′-dicyclohexylcarbodiimide
DCM: dichloromethane
DIAD: diisopropyl azodicarboxylate
DMAP: 4-(dimethylamino)pyridine
DMEM: Dulbecco’s modified Eagle’s medium
DMF: dimethylformamide
DMSO: dimethyl sulfoxide
DNA: DNA
DPBS: Dulbecco’s phosphate buffered saline
HBV: hepatitis B virus
HDP: hexadecyloxypropyl
HIV: human immunodeficiency virus
HLM: human liver microsome
HMPA: hexamethylphosphoramide
HPLC: high performance liquid chromatography
HSA: human serum albumin
HSV: herpes simplex virus
HTP: hexadecylthiopropyl
ISTD: internal standard
LC–MS: liquid chromatography–mass spectrometry
LC–MS/MS: liquid chromatography–tandem mass spectrometry
MLM: mouse liver microsome
NADPH: nicotinamide adenine dinucleotide phosphate
NMP: *N*-methylpyrrolidone
Ph: phenyl
POC: isopropyloxycarbonyloxymethyl
PK: pharmacokinetics
PLA_1_: phospholipase A_1_
PLA_2_: phospholipase A_2_
PLC: phospholipase C
PLD: phospholipase D
PMA: phosphomolybdic acid
R_f_: retention factors
SAR: structure–activity relationship
SD: standard deviation
SEM: standard error of the mean
TAF: tenofovir alafenamide
TBAF: tetrabutylammonium fluoride
TDF: tenofovir disoproxil fumarate
TFV: tenofovir
TFV-DP: tenofovir diphosphate
THF: tetrahydrofuran
THP: tetrahydropyranyl
TLC: thin-layer chromatography
TMS: trimethylsilyl
TIPS: triisopropylsilyl
TXL: tenofovir exalidex
*V*_d_: volume of distribution
