# Supplementary material for: Building Metabolically Stable and Potent Anti-HIV Thioether-Lipid Analogues of Tenofovir Exalidex: A thorough Pharmacological Analysis
Source: J Med Chem. 2024 Oct 16;67(20):18204–20. doi: 10.1021/acs.jmedchem.4c01510 (PMC11513920; doi:10.1021/acs.jmedchem.4c01510)
Supplement: Supplementary file 1 — jm4c01510_si_001.pdf [file jm4c01510_si_001.pdf]

# SUPPORTING INFORMATION

# Building Metabolically Stable and Potent Anti-HIV Thioether-Lipid Analogues of Tenofovir Exalidex: A Thorough Pharmacological Analysis

*Michael P. D'Erasmo[1], Savita K. Sharma[1], Nicole Pribut[1], Adriaan Basson[2], Madhuri Dasari[1], Perry Bartsch[1], Sabrina E. Iskandar[1], Kyle E. Giesler[1], Samantha Burton[1], Cindy A. Derdeyn[3], Dennis C. Liotta[1], Eric J. Miller*[4]*

[1]Department of Chemistry, Emory University College of Arts & Sciences, Atlanta, GA 30322, USA

[2]HIV Pathogenesis Research Unit, Department of Molecular Medicine and Haematology, University of the Witwatersrand, Johannesburg, Gauteng, South Africa

[3]Department of Laboratory Medicine & Pathology, University of Washington School of Medicine, Seattle, WA 98195, USA

[4]Department of Pharmacology & Chemical Biology, Emory University School of Medicine, Atlanta, GA 30322, USA

*Corresponding Author: Eric J. Miller, ejmill2@emory.edu

Contents

Chemical Synthesis and Characterization……………………………………………………..S3

*In Vitro* Protocols and Data Analysis…………………………………………...…….S56

Instrumentation and LC-MS/MS Method Development……………………………..S56

Metabolic Stability Assays…………………………………………………………...S59

Liver Microsome Stability…………………………………………………...S59

Plasma Stability……………………………………………………………...S74

HepaRG$^{TM}$ Cellular Uptake and Stability……………………………………………S78

Cellular Uptake………………………………………………………………S78

Cellular Stability……………………………………………………………..S81

*In Vivo* Mouse Pharmacokinetics Study (Conducted by Sai Life Sciences)……………………S88

Plasma Pharmacokinetics…………………………………………………………….S88

Liver Pharmacokinetics……………………………………………………………...S98

Kidney Pharmacokinetics…………………………………………………………..S102

Brain Pharmacokinetics……………………………………………………………S106

LC-MS Traces For HTS-Derived TFV Prodrugs……………………………………..S110

Example of Acetylenic Terminal Group Degradation Over Time (Compound **15h**)…S110

LC-MS Traces For Compounds **2a**, **16g**, and **16h** Prior to *In Vivo* Analysis…………S111

Predicted Pharmacological Properties for Key Lipid Intermediates and TFV Prodrugs……….S114

References...........................................................................................................................S115

Spectra……………………………………………………………………………………...S117

## Chemical Synthesis and Characterization

*Synthesis of Long-Chain (Alkynyloxy)tetrahydropyrans*

General Procedure for the Alkylation of Terminal Alkynes. Alkyne (2.50-3.00 eq) and anhydrous THF (0.6 M) were added to an oven-dried flask equipped with a magnetic stir bar under an atmosphere of Ar. After cooling the solution to -78 $^oC$, *n*-BuLi (2.5 M in hexanes, 2.50-3.00 eq) was added dropwise. The resulting reaction mixture was stirred vigorously at -78 $^oC$ for 15 min, then warmed to -40 $^oC$ for an additional 1 h. While maintaining the reaction mixture at -40 $^oC$, HMPA (3.00 eq) and (bromoalkoxy)tetrahydropyran[1] (**8**, 1.00 eq) dissolved in anhydrous THF were added sequentially. The following reaction mixture was stirred vigorously at -40 $^oC$ for 1 h, then slowly warmed to room temperature and stirred for an additional 5-24 h. Upon confirming complete conversion of starting material by TLC, the reaction mixture was cooled to 0 $^oC$ and carefully quenched with saturated aqueous $NH_4Cl$. Next, the aqueous layer was extracted with EtOAc (3x), and the combined organic phase was washed with brine, dried with either $Na_2SO_4$ or $MgSO_4$, and concentrated under reduced pressure. Finally, the resulting crude material was purified by column chromatography to yield long-chain (alkynyloxy)tetrahydropyrans.

***Trimethyl(16-((tetrahydro-2H-pyran-2-yl)oxy)hexadec-1-yn-1-yl)silane* (9a).** Synthesis was carried out according to *General Procedure for the Alkylation of Terminal Alkynes* using 2-(14-bromotetradecoxy)tetrahydropyran (6.25 g, 16.6 mmol, 1.00 eq), trimethylsilylacetylene (6.88 mL, 49.7 mmol, 3.00 eq), and *n*-BuLi (2.5 M in hexanes, 19.9 mL, 49.7 mmol, 3.00 eq). Purified via column chromatography eluting along a gradient of 0-1% EtOAc in hexanes to yield a colorless oil (5.72 g, 14.5 mmol, 88% yield). **TLC** (5% EtOAc in hexanes) $R_f$ = 0.27. **$^1$H NMR** (400 MHz, $CDCl_3$) δ 4.57 (dd, *J* = 4.4, 2.7 Hz, 1H), 3.92 – 3.83 (m, 1H), 3.73 (dt, *J* = 9.5, 6.9 Hz, 1H), 3.54 – 3.46 (m, 1H), 3.38 (dt, *J* = 9.5, 6.7 Hz, 1H), 2.21 (t, *J* = 7.2 Hz,

2H), 1.90 – 1.77 (m, 1H), 1.76 – 1.65 (m, 1H), 1.65 – 1.45 (m, 8H), 1.44 – 1.19 (m, 20H), 0.14 (s, 9H). **$^{13}$C NMR** (151 MHz, $CDCl_3$) δ 108.0, 99.0, 84.4, 67.9, 62.5, 31.0, 29.9, 29.81, 29.80, 29.77, 29.76, 29.75, 29.7, 29.2, 29.0, 28.8, 26.4, 25.7, 20.0, 19.9, 0.3 (*21 out of 24 carbon signals observed due to chemical shift equivalence*). **HRMS** (APCI) *m/z* calculated for $C_{24}H_{47}O_2Si^+$ $[M + H]^+$: 395.33398, found 395.33357.

***Trimethyl(14-((tetrahydro-2H-pyran-2-yl)oxy)tetradec-1-yn-1-yl)silane* (9c).** Synthesis was carried out according to *General Procedure for the Alkylation of Terminal Alkynes* using 2-(12-bromododecoxy)tetrahydropyran (5.96 g, 17.0 mmol, 1.00 eq), trimethylsilylacetylene (7.08 mL, 51.1 mmol, 3.00 eq), and *n*-BuLi (2.5 M in hexanes, 20.5 mL, 51.1 mmol, 3.00 eq). Purified via column chromatography eluting along a gradient of 0-1% EtOAc in hexanes to yield a colorless oil (5.49 g, 15.0 mmol, 88% yield). **TLC** (5% EtOAc in hexanes) $R_f$ = 0.27. **$^1$H NMR** (600 MHz, $CDCl_3$) δ 4.57 (dd, *J* = 4.5, 2.9 Hz, 1H), 3.90 – 3.84 (m, 1H), 3.72 (dt, *J* = 9.5, 6.9 Hz, 1H), 3.53 – 3.46 (m, 1H), 3.38 (dt, *J* = 9.6, 6.8 Hz, 1H), 2.20 (t, *J* = 7.2 Hz, 2H), 1.88 – 1.78 (m, 1H), 1.75 – 1.67 (m, 1H), 1.64 – 1.46 (m, 8H), 1.41 – 1.21 (m, 16H), 0.14 (s, 9H). **$^{13}$C NMR** (151 MHz, $CDCl_3$) δ 107.9, 99.0, 84.4, 67.9, 62.5, 31.0, 29.9, 29.8, 29.74, 29.72, 29.64, 29.63, 29.2, 29.0, 28.8, 26.4, 25.7, 20.0, 19.9, 0.3 (*20 out of 22 carbon signals observed due to chemical shift equivalence*). **HRMS** (APCI) *m/z* calculated for $C_{22}H_{43}O_2Si^+$ $[M + H]^+$: 367.30268, found 367.30297.

***Triisopropyl(13-((tetrahydro-2H-pyran-2-yl)oxy)tridec-1-yn-1-yl)silane* (9d).** Synthesis was carried out according to *General Procedure for the Alkylation of Terminal Alkynes* using 2-(11-bromoundecoxy)tetrahydropyran (6.30 g, 18.8 mmol, 1.00 eq), (triisopropylsilyl)-acetylene (12.5 mL, 56.4 mmol, 3.00 eq), and *n*-BuLi (2.5 M in hexanes, 22.5 mL, 56.4 mmol, 3.00 eq). Purified via column chromatography eluting along a gradient of 0-1% EtOAc in hexanes

to yield a pale yellow oil (6.86 g, 15.7 mmol, 84% yield). **TLC** (5% EtOAc in hexanes) $R_f$ = 0.33. **$^{1}$H NMR** (600 MHz, $CDCl_3$) δ 4.57 (dd, *J* = 4.6, 2.8 Hz, 1H), 3.90 – 3.83 (m, 1H), 3.73 (dt, *J* = 9.6, 6.9 Hz, 1H), 3.53 – 3.47 (m, 1H), 3.38 (dt, *J* = 9.6, 6.7 Hz, 1H), 2.24 (t, *J* = 7.0 Hz, 2H), 1.90 – 1.78 (m, 1H), 1.75 – 1.67 (m, 1H), 1.64 – 1.47 (m, 8H), 1.45 – 1.38 (m, 2H), 1.38 – 1.22 (m, 12H), 1.09 – 0.97 (m, 21H). **$^{13}$C NMR** (151 MHz, $CDCl_3$) δ 109.5, 99.0, 80.1, 67.9, 62.5, 31.0, 29.9, 29.74, 29.69, 29.66, 29.2, 29.0, 28.8, 26.4, 25.7, 20.0, 19.9, 18.8, 11.5 (*19 out of 27 carbon signals observed due to chemical shift equivalence*). **HRMS** (APCI) *m/z* calculated for $C_{27}H_{53}O_2Si^+$ [M + H]$^+$: 437.38093, found 437.38080.

***2-((15,15-Dimethylhexadec-13-yn-1-yl)oxy)tetrahydro-2H-pyran* (9e).** Synthesis was carried out according to *General Procedure for the Alkylation of Terminal Alkynes* using 2-(12-bromododecoxy)tetrahydropyran (11.9 g, 34.2 mmol, 1.00 eq), *t*-butylacetylene (12.6 mL, 103 mmol, 3.00 eq), and *n*-BuLi (2.5 M in hexanes, 41.0 mL, 103 mmol, 3.00 eq). Purified via column chromatography eluting along a gradient of 0-1% EtOAc in hexanes to yield a colorless oil (11.3 g, 32.2 mmol, 94% yield). **TLC** (5% EtOAc in hexanes) $R_f$ = 0.31. **$^{1}$H NMR** (400 MHz, $CDCl_3$) δ 4.57 (dd, *J* = 4.3, 2.7 Hz, 1H), 3.92 – 3.83 (m, 1H), 3.73 (dt, *J* = 9.6, 6.9 Hz, 1H), 3.55 – 3.45 (m, 1H), 3.38 (dt, *J* = 9.6, 6.7 Hz, 1H), 2.12 (t, *J* = 7.1 Hz, 2H), 1.90 – 1.77 (m, 1H), 1.76 – 1.65 (m, 1H), 1.64 – 1.40 (m, 8H), 1.40 – 1.22 (m, 16H), 1.19 (s, 9H). **$^{13}$C NMR** (151 MHz, $CDCl_3$) δ 99.0, 89.1, 78.7, 67.9, 62.5, 31.6, 31.0, 29.9, 29.76, 29.75, 29.74, 29.69, 29.66, 29.4, 29.3, 28.9, 27.5, 26.4, 25.7, 19.9, 18.8 (*21 out of 23 carbon signals observed due to chemical shift equivalence*). **HRMS** (APCI) *m/z* calculated for $C_{23}H_{43}O_2^+$ [M + H]$^+$: 351.32576, found 351.32522.

***2-((12-Phenyldodec-11-yn-1-yl)oxy)tetrahydro-2H-pyran* (9f).** Synthesis was carried out according to *General Procedure for the Alkylation of Terminal Alkynes* using

2-(10-bromodecoxy)tetrahydropyran (5.27 g, 16.4 mmol, 1.00 eq), phenylacetylene (5.40 mL, 49.2 mmol, 3.00 eq), and *n*-BuLi (2.5 M in hexanes, 19.7 mL, 49.2 mmol, 3.00 eq). Purified via column chromatography eluting along a gradient of 0-1% EtOAc in hexanes to yield a pale yellow oil (4.96 g, 14.5 mmol, 88% yield). **TLC** (5% EtOAc in hexanes) $R_f$ = 0.22. **$^1$H NMR** (400 MHz, $CDCl_3$) δ 7.42 – 7.36 (m, 2H), 7.31 – 7.22 (m, 3H), 4.57 (dd, *J* = 4.4, 2.7 Hz, 1H), 3.92 – 3.83 (m, 1H), 3.73 (dt, *J* = 9.6, 6.9 Hz, 1H), 3.54 – 3.45 (m, 1H), 3.38 (dt, *J* = 9.6, 6.6 Hz, 1H), 2.40 (t, *J* = 7.1 Hz, 2H), 1.90 – 1.77 (m, 1H), 1.76 – 1.65 (m, 1H), 1.64 – 1.48 (m, 8H), 1.48 – 1.40 (m, 2H), 1.39 – 1.24 (m, 10H). **$^{13}$C NMR** (151 MHz, $CDCl_3$) δ 131.7, 128.3, 127.6, 124.3, 99.0, 90.6, 80.7, 67.8, 62.5, 31.0, 29.9, 29.7, 29.6, 29.3, 29.1, 28.9, 26.4, 25.7, 19.9, 19.6 (*20 out of 23 carbon signals observed due to chemical shift equivalence*). **HRMS** (APCI) *m/z* calculated for $C_{23}H_{35}O_2^+$ $[M + H]^+$: 343.26316, found 343.26384.

***2-((12-Cyclohexyldodec-11-yn-1-yl)oxy)tetrahydro-2H-pyran* (9h).** Synthesis was carried out according to *General Procedure for the Alkylation of Terminal Alkynes* using 2-(10-bromodecoxy)tetrahydropyran (10.0 g, 31.1 mmol, 1.00 eq), cyclohexylacetylene (10.2 mL, 77.8 mmol, 2.50 eq), and *n*-BuLi (2.5 M in hexanes, 31.1 mL, 77.8 mmol, 2.50 eq). Purified via column chromatography eluting along a gradient of 0-1% EtOAc in hexanes to yield a colorless oil (10.0 g, 28.7 mmol, 92% yield). **TLC** (5% EtOAc in hexanes) $R_f$ = 0.29. **$^1$H NMR** (400 MHz, $CDCl_3$) δ 4.57 (dd, *J* = 4.4, 2.7 Hz, 1H), 3.92 – 3.83 (m, 1H), 3.72 (dt, *J* = 9.6, 6.9 Hz, 1H), 3.54 – 3.45 (m, 1H), 3.37 (dt, *J* = 9.6, 6.7 Hz, 1H), 2.36 – 2.26 (m, 1H), 2.14 (td, *J* = 7.0, 2.2 Hz, 2H), 1.90 – 1.63 (m, 6H), 1.63 – 1.41 (m, 9H), 1.41 – 1.20 (m, 17H). **$^{13}$C NMR** (151 MHz, $CDCl_3$) δ 99.0, 84.8, 80.3, 67.8, 62.5, 33.4, 31.0, 29.9, 29.7, 29.6, 29.4, 29.32, 29.28, 29.0, 26.4, 26.1, 25.7, 25.1, 19.9, 18.9 (*20 out of 23 carbon signals observed due to chemical shift equivalence*). **HRMS** (APCI) *m/z* calculated for $C_{23}H_{41}O_2^+$ $[M + H]^+$: 349.31011, found 349.30910.

*Synthesis of Long-Chain Alcohols*

General Procedure A for THP Deprotection. (Alkynyloxy)tetrahydropyran (1.00 eq) and MeOH (0.4 M) were added to an oven-dried flask equipped with a magnetic stir bar under an atmosphere of Ar. While stirring the solution vigorously at room temperature, *p*-toluenesulfonic acid monohydrate (10 mol %) was added in one portion. The reaction mixture was then stirred overnight at room temperature. After confirming complete conversion of starting material by TLC, the reaction mixture was concentrated under reduced pressure and taken up in EtOAc. Next, the organic layer was washed with water and brine, dried with $MgSO_4$, and concentrated under reduced pressure. Finally, the resulting crude material was purified by column chromatography to yield long-chain alkynols.

General Procedure B for THP Deprotection. (Alkynyloxy)tetrahydropyran (1.00 eq) and MeOH (0.3-0.4 M) were added to an oven-dried flask equipped with a magnetic stir bar under an atmosphere of Ar. While stirring the solution vigorously at room temperature, *p*-toluenesulfonic acid monohydrate (10 mol %) was added in one portion. Next, the reaction mixture was stirred overnight at room temperature. After confirming complete conversion of starting material by TLC, the reaction mixture was concentrated under reduced pressure. Lastly, the resulting crude material was immediately purified by column chromatography to yield long-chain alkynols.

***16-(Trimethylsilyl)hexadec-15-yn-1-ol*** **(11a).** Synthesis was carried out according to *General Procedure B for THP Deprotection* using compound **9a** (5.72 g, 14.5 mmol, 1.00 eq). Purified via column chromatography eluting along a gradient of 0-10% EtOAc in hexanes to yield a white crystalline solid (3.09 g, 9.94 mmol, 69% yield). MP = 32-34 °C. **TLC** (20% EtOAc in hexanes) $R_f$ = 0.32. $^{1}$**H NMR** (400 MHz, $CDCl_3$) δ 3.64 (td, *J* = 6.6, 5.5 Hz, 2H), 2.21 (t, *J* = 7.2 Hz, 2H), 1.61 – 1.46 (m, 4H), 1.41 – 1.22 (m, 20H), 1.23 (t, *J* = 5.5 Hz, 1H), 0.14 (s, 9H). $^{13}$**C NMR** (151

MHz, $CDCl_3$) δ 108.0, 84.4, 63.3, 33.0, 29.80, 29.79, 29.76, 29.74, 29.74, 29.64, 29.59, 29.2, 29.0, 28.8, 25.9, 20.0, 0.3 (*17 out of 19 carbon signals observed due to chemical shift equivalence*). **HRMS** (APCI) *m/z* calculated for $C_{19}H_{39}OSi^+$ $[M + H]^+$: 311.27647, found 311.27585.

***14-(Trimethylsilyl)tetradec-13-yn-1-ol*** **(11c).** Synthesis was carried out according to *General Procedure B for THP Deprotection* using compound **9c** (5.49 g, 15.0 mmol, 1.00 eq). Purified via column chromatography eluting along a gradient of 0-10% EtOAc in hexanes to yield a colorless oil (3.03 g, 10.7 mmol, 72% yield). **TLC** (20% EtOAc in hexanes) $R_f = 0.31$. **$^1$H NMR** (400 MHz, $CDCl_3$) δ 3.64 (td, $J$ = 6.6, 5.5 Hz, 2H), 2.21 (t, $J$ = 7.2 Hz, 2H), 1.61 – 1.46 (m, 4H), 1.41 – 1.24 (m, 16H), 1.22 (t, $J$ = 5.5 Hz, 1H), 0.14 (s, 9H). **$^{13}$C NMR** (151 MHz, $CDCl_3$) δ 108.0, 84.4, 63.3, 33.0, 29.8, 29.7, 29.62, 29.58, 29.2, 28.9, 28.8, 25.9, 20.0, 0.3 (*14 out of 17 carbon signals observed due to chemical shift equivalence*). **HRMS** (APCI) *m/z* calculated for $C_{17}H_{35}OSi^+$ $[M + H]^+$: 283.24517, found 283.24550.

***13-(Triisopropylsilyl)tridec-12-yn-1-ol*** **(11d).** Synthesis was carried out according to *General Procedure A for THP Deprotection* using compound **9d** (6.86 g, 15.7 mmol, 1.00 eq). Purified via column chromatography eluting along a gradient of 0-10% EtOAc in hexanes to yield a colorless oil (5.05 g, 14.3 mmol, 91% yield). **TLC** (20% EtOAc in hexanes) $R_f = 0.35$. **$^1$H NMR** (400 MHz, $CD_3OD$) δ 3.54 (t, $J$ = 6.7 Hz, 2H), 2.27 (t, $J$ = 6.5 Hz, 2H), 1.58 – 1.42 (m, 6H), 1.41 – 1.26 (m, 12H), 1.12 – 0.94 (m, 21H). **$^{13}$C NMR** (151 MHz, $CD_3OD$) δ 110.6, 80.8, 63.0, 33.7, 30.8, 30.7, 30.64, 30.62, 30.1, 29.8, 29.6, 27.0, 20.4, 19.1, 12.5 (*15 out of 22 carbon signals observed due to chemical shift equivalence*). **HRMS** (APCI) *m/z* calculated for $C_{22}H_{45}OSi^+$ $[M + H]^+$: 353.32342, found 353.32302.

***15,15-Dimethylhexadec-13-yn-1-ol*** **(11e).** Synthesis was carried out according to *General Procedure A for THP Deprotection* using compound **9e** (10.8 g, 30.8 mmol, 1.00 eq). Purified via

column chromatography eluting along a gradient of 0-10% EtOAc in hexanes to yield a colorless oil (5.67 g, 21.3 mmol, 69% yield). **TLC** (20% EtOAc in hexanes) $R_f$ = 0.31. **$^1$H NMR** (400 MHz, $CD_3OD$) δ 3.54 (t, *J* = 6.7 Hz, 2H), 2.11 (t, *J* = 6.7 Hz, 2H), 1.57 – 1.48 (m, 2H), 1.48 – 1.25 (m, 18H), 1.17 (s, 9H). **$^{13}$C NMR** (100 MHz, $CD_3OD$) δ 89.7, 79.4, 63.0, 33.7, 31.9, 30.8, 30.74, 30.71, 30.69, 30.6, 30.23, 30.21, 29.8, 28.3, 27.0, 19.3 (*16 out of 18 carbon signals observed due to chemical shift equivalence*). **HRMS** (APCI) *m/z* calculated for $C_{18}H_{35}O^+$ $[M + H]^+$: 267.26824, found 267.26781.

***12-Phenyldodec-11-yn-1-ol* (11f).** Synthesis was carried out according to *General Procedure B for THP Deprotection* using compound **9f** (5.10 g, 14.9 mmol, 1.00 eq). Purified via column chromatography eluting along a gradient of 0-10% EtOAc in hexanes to yield a colorless oil (2.65 g, 10.2 mmol, 69% yield). **TLC** (20% EtOAc in hexanes) $R_f$ = 0.23. **$^1$H NMR** (600 MHz, $CD_3OD$) δ 7.36 – 7.31 (m, 2H), 7.30 – 7.23 (m, 3H), 3.53 (t, *J* = 6.7 Hz, 2H), 2.39 (t, *J* = 7.0 Hz, 2H), 1.63 – 1.56 (m, 2H), 1.55 – 1.44 (m, 4H), 1.40 – 1.28 (m, 10H). **$^{13}$C NMR** (151 MHz, $CD_3OD$) δ 132.4, 129.3, 128.5, 125.6, 90.9, 81.6, 63.0, 33.7, 30.7, 30.61, 30.58, 30.2, 30.0, 29.9, 27.0, 20.0 (*16 out of 18 carbon signals observed due to chemical shift equivalence*). **HRMS** (APCI) *m/z* calculated for $C_{18}H_{27}O^+$ $[M + H]^+$: 259.20564, found 259.20527.

***12-Cyclohexyldodec-11-yn-1-ol* (11h).** Synthesis was carried out according to *General Procedure B for THP Deprotection* using compound **9h** (10.0 g, 28.7 mmol, 1.00 eq). Purified via column chromatography eluting along a gradient of 0-10% EtOAc in hexanes to yield a colorless oil (5.63 g, 21.3 mmol, 74% yield). **TLC** (20% EtOAc in hexanes) $R_f$ = 0.30. **$^1$H NMR** (400 MHz, $CD_3OD$) δ 3.54 (t, *J* = 6.7 Hz, 2H), 2.37 – 2.27 (m, 1H), 2.13 (td, *J* = 6.7, 2.1 Hz, 2H), 1.80 – 1.65 (m, 4H), 1.58 – 1.25 (m, 22H). **$^{13}$C NMR** (151 MHz, $CD_3OD$) δ 85.3, 80.9, 63.0, 34.4, 33.7, 30.7, 30.64, 30.60, 30.3, 30.2, 29.8, 27.1, 27.0, 25.9, 19.4 (*15 out of 18 carbon signals observed due to chemical*

*shift equivalence*). **HRMS** (APCI) *m/z* calculated for $C_{18}H_{33}O^+$ $[M + H]^+$: 265.25259, found 265.25231.

***12-(4-Fluorophenyl)dodec-11-yn-1-ol* (11g).** 11-Dodecyn-1-ol[2] (**10**, 4.82 g, 26.4 mmol, 1.00 eq), 1-fluoro-4-iodobenzene (3.66 mL, 31.7 mmol, 1.20 eq), Ar-degassed triethylamine (11.1 mL, 79.3 mmol, 3.00 eq), and Ar-degassed anhydrous THF (66 mL, 0.4 M) were added to an oven-dried Schlenk flask equipped with a magnetic stir bar under an atmosphere of Ar. While stirring the mixture vigorously at room temperature, bis(triphenylphosphine)palladium(II) dichloride (928 mg, 1.32 mmol, 5 mol %) and copper (I) iodide (503 mg, 2.64 mmol, 10 mol %) were added sequentially. The reaction mixture was then heated to 70 $^{o}$C and stirred overnight. After confirming complete conversion of starting material by TLC, the reaction mixture was cooled to room temperature and filtered through a pad of Celite. Next, the Celite was washed with EtOAc, and the collected organic phase was washed with saturated aqueous $NH_4Cl$ (2x) and brine, dried with $Na_2SO_4$, and concentrated under reduced pressure. Finally, the resulting crude material was purified by column chromatography eluting along a gradient of 0-70% DCM in hexanes to yield a light brown solid (4.50 g, 16.3 mmol, 62% yield). MP = 45-47 $^{o}$C. **TLC** (20% EtOAc in hexanes) $R_f$ = 0.24. **$^1$H NMR** (400 MHz, $CD_3OD$) δ 7.39 – 7.33 (m, 2H), 7.06 – 6.99 (m, 2H), 3.53 (t, *J* = 6.6 Hz, 2H), 2.39 (t, *J* = 7.0 Hz, 2H), 1.63 – 1.42 (m, 6H), 1.41 – 1.28 (m, 10H). **$^{13}$C NMR** (151 MHz, $CD_3OD$) δ 163.4 (d, $J_{CF}$ = 246.7 Hz), 134.4 (d, $J_{CF}$ = 8.3 Hz), 121.8 (d, $J_{CF}$ = 3.5 Hz), 116.3 (d, $J_{CF}$ = 22.2 Hz), 90.7 (d, $J_{CF}$ = 1.1 Hz), 80.4, 63.0, 33.7, 30.7, 30.60, 30.58, 30.2, 30.0, 29.9, 27.0, 19.9 (*16 out of 18 carbon signals observed due to chemical shift equivalence*). **HRMS** (APCI) *m/z* calculated for $C_{18}H_{26}FO^+$ $[M + H]^+$: 277.19622, found 277.19561.

General Procedure for Alkyne Hydrogenation. Alkynol (1.00 eq) and either MeOH or EtOAc (0.1 M) were added to an oven-dried flask equipped with a magnetic stir bar under an atmosphere of

Ar. While stirring the solution vigorously at room temperature, palladium on carbon (10 wt %, 10 mol %) was added portion-wise. The reaction flask was then quickly degassed under vacuum and subsequently purged with Ar. After repeating the vacuum/purge cycle two more times, the heterogeneous mixture was placed under vacuum and then subjected to a balloon of hydrogen gas at room temperature for 3-16 h. Upon confirming complete conversion of starting material by TLC, the reaction mixture was filtered through a pad of Celite. Next, the Celite was washed with EtOAc, and the collected organic phase was concentrated under reduced pressure. Finally, the resulting crude material was then immediately purified by column chromatography to yield reduced long-chain alcohols.

***15,15-Dimethylhexadecan-1-ol* (12e).** Synthesis was carried out according to *General Procedure for Alkyne Hydrogenation* using compound **11e** (3.07 g, 11.5 mmol, 1.00 eq) and EtOAc (115 mL, 0.1 M). Purified via column chromatography eluting along a gradient of 0-10% EtOAc in hexanes to yield a colorless oil (1.71 g, 6.32 mmol, 55% yield). **TLC** (20% EtOAc in hexanes) $R_f$ = 0.35. **$^1$H NMR** (400 MHz, $CD_3OD$) δ 3.54 (t, *J* = 6.7 Hz, 2H), 1.58 – 1.48 (m, 2H), 1.41 – 1.13 (m, 24H), 0.88 (s, 9H). **$^{13}$C NMR** (100 MHz, $CD_3OD$) δ 63.0, 45.5, 33.7, 31.8, 31.1, 30.84, 30.80, 30.77, 30.7, 29.9, 27.0, 25.7 (*12 out of 18 carbon signals observed due to chemical shift equivalence*). **HRMS** (ESI) *m/z* calculated for $C_{18}H_{38}ONa^+$ $[M + Na]^+$: 293.28149, found 293.28136.

***12-Phenyldodecan-1-ol* (12f).** Synthesis was carried out according to *General Procedure for Alkyne Hydrogenation* using compound **11f** (2.65 g, 10.2 mmol, 1.00 eq) and MeOH (102 mL, 0.1 M). Purified via column chromatography eluting along a gradient of 0-10% EtOAc in hexanes to yield a white powder (2.18 g, 8.30 mmol, 81% yield). MP = 46-48 $^o$C. **TLC** (20% EtOAc in hexanes) $R_f$ = 0.28. **$^1$H NMR** (600 MHz, $CD_3OD$) δ 7.26 – 7.21 (m, 2H), 7.17 – 7.10 (m, 3H),

3.54 (t, $J$ = 6.7 Hz, 2H), 2.61 – 2.57 (m, 2H), 1.65 – 1.56 (m, 2H), 1.56 – 1.48 (m, 2H), 1.38 – 1.25 (m, 16H). **$^{13}$C NMR** (151 MHz, $CD_3OD$) δ 144.0, 129.4, 129.2, 126.6, 63.0, 36.9, 33.7, 32.8, 30.8, 30.72, 30.71, 30.61, 30.59, 30.3, 27.0 (*15 out of 18 carbon signals observed due to chemical shift equivalence*). **HRMS** (NSI) *m/z* calculated for $C_{18}H_{30}ONa^+$ $[M + Na]^+$: 285.21889, found 285.21830.

***12-(4-Fluorophenyl)dodecan-1-ol* (12g).** Synthesis was carried out according to *General Procedure for Alkyne Hydrogenation* using compound **11g** (2.26 g, 8.18 mmol, 1.00 eq) and EtOAc (82 mL, 0.1 M). Purified via column chromatography eluting along a gradient of 0-10% EtOAc in hexanes to yield a white solid (1.90 g, 6.78 mmol, 83% yield). MP = 40-42 $^o$C. **TLC** (20% EtOAc in hexanes) $R_f$ = 0.27. **$^1$H NMR** (400 MHz, $CD_3OD$) δ 7.19 – 7.12 (m, 2H), 7.00 – 6.92 (m, 2H), 3.53 (t, $J$ = 6.7 Hz, 2H), 2.61 – 2.55 (m, 2H), 1.64 – 1.47 (m, 4H), 1.40 – 1.23 (m, 16H). **$^{13}$C NMR** (151 MHz, $CD_3OD$) δ 162.6 (d, $J_{CF}$ = 241.6 Hz), 139.9 (d, $J_{CF}$ = 3.0 Hz), 130.9 (d, $J_{CF}$ = 7.7 Hz), 115.7 (d, $J_{CF}$ = 21.3 Hz), 63.0, 36.0, 33.7, 32.8, 30.8, 30.72, 30.70, 30.69, 30.61, 30.56, 30.2, 27.0 (*16 out of 18 carbon signals observed due to chemical shift equivalence*). **HRMS** (ESI) *m/z* calculated for $C_{18}H_{29}FONa^+$ $[M + Na]^+$: 303.20946, found 303.20924.

***12-Cyclohexyldodecan-1-ol* (12h).** Synthesis was carried out according to *General Procedure for Alkyne Hydrogenation* using compound **11h** (3.00 g, 11.3 mmol, 1.00 eq) and EtOAc (113 mL, 0.1 M). Purified via column chromatography eluting along a gradient of 0-10% EtOAc in hexanes to yield a white solid (1.88 g, 7.00 mmol, 62% yield). MP = 46-49 $^o$C. **TLC** (20% EtOAc in hexanes) $R_f$ = 0.34. **$^1$H NMR** (400 MHz, $CD_3OD$) δ 3.54 (t, $J$ = 6.7 Hz, 2H), 1.77 – 1.61 (m, 5H), 1.58 – 1.47 (m, 2H), 1.41 – 1.10 (m, 24H), 0.96 – 0.81 (m, 2H). **$^{13}$C NMR** (151 MHz, $CD_3OD$) δ 63.0, 39.0, 38.8, 34.7, 33.7, 31.1, 30.81, 30.79, 30.75, 30.6, 28.0, 27.9, 27.6, 27.0 (*14 out of 18*

*carbon signals observed due to chemical shift equivalence*). **HRMS** (NSI) *m/z* calculated for $C_{18}H_{36}ONa^+$ [M + Na]$^+$: 291.26584, found 291.26582.

*Synthesis of Long-Chain Alkyl Bromides*

General Procedure for Mitsunobu Reaction. 11-Bromo-1-undecanol (**17**, 1.00 eq), phenol derivative (1.00 eq), triphenylphosphine (1.00 eq), and anhydrous THF (0.5 M) were added to an oven-dried flask equipped with a magnetic stir bar under an atmosphere of Ar. The solution was then stirred vigorously to ensure dissolution of starting materials and cooled to 0 $^o$C. Next, diisopropyl azodicarboxylate (1.10 eq) was added dropwise, and the reaction mixture was slowly warmed to room temperature and stirred overnight. After confirming complete conversion of starting material by TLC, the reaction mixture was concentrated under reduced pressure and taken up in hexanes. The resulting heterogeneous mixture was then filtered through a pad of Celite. Subsequently, the Celite was washed with hexanes, and the collected filtrate was concentrated under reduced pressure. Lastly, the crude material was immediately purified by column chromatography to yield long-chain alkyl bromides.

***((11-Bromoundecyl)oxy)benzene*** **(19a).** Synthesis was carried out according to *General Procedure for Mitsunobu Reaction* using compound **17** (3.00 g, 11.9 mmol, 1.00 eq) and phenol (1.12 g, 11.9 mmol, 1.00 eq). Purified via column chromatography eluting along a gradient of 0-1% EtOAc in hexanes to yield a colorless oil (2.75 g, 8.40 mmol, 70% yield). **TLC** (2% EtOAc in hexanes) $R_f$ = 0.48. **$^1$H NMR** (400 MHz, $CDCl_3$) δ 7.31 – 7.24 (m, 2H), 6.96 – 6.87 (m, 3H), 3.95 (t, *J* = 6.6 Hz, 2H), 3.41 (t, *J* = 6.9 Hz, 2H), 1.90 – 1.73 (m, 4H), 1.51 – 1.24 (m, 14H). **$^{13}$C NMR** (151 MHz, $CDCl_3$) δ 159.3, 129.5, 120.6, 114.6, 68.0, 34.2, 33.0, 29.7, 29.60, 29.56, 29.52, 29.45, 28.9, 28.3, 26.2 (*15 out of 17 carbon signals observed due to chemical shift equivalence*). **HRMS** (APCI) *m/z* calculated for $C_{17}H_{28}BrO^+$ [M + H]$^+$: 327.13180, found 327.13134.

***1-((11-Bromoundecyl)oxy)-4-fluorobenzene* (19c).** Synthesis was carried out according to *General Procedure for Mitsunobu Reaction* using compound **17** (3.07 g, 12.2 mmol, 1.00 eq) and 4-fluorophenol (1.37 g, 12.2 mmol, 1.00 eq). Purified via column chromatography eluting along a gradient of 0-1% EtOAc in hexanes to yield a colorless oil (3.26 g, 9.44 mmol, 77% yield). **TLC** (2% EtOAc in hexanes) $R_f = 0.41$. **$^{1}$H NMR** (400 MHz, $CDCl_3$) δ 7.00 – 6.92 (m, 2H), 6.86 – 6.79 (m, 2H), 3.90 (t, $J$ = 6.6 Hz, 2H), 3.41 (t, $J$ = 6.8 Hz, 2H), 1.90 – 1.81 (m, 2H), 1.80 – 1.71 (m, 2H), 1.49 – 1.24 (m, 14H). **$^{13}$C NMR** (151 MHz, $CDCl_3$) δ 157.3 (d, $J_{CF}$ = 237.7 Hz), 155.4 (d, $J_{CF}$ = 1.9 Hz), 115.9 (d, $J_{CF}$ = 22.9 Hz), 115.6 (d, $J_{CF}$ = 7.9 Hz), 68.8, 34.2, 33.0, 29.7, 29.60, 29.56, 29.5, 29.4, 28.9, 28.3, 26.2 (*15 out of 17 carbon signals observed due to chemical shift equivalence*). **HRMS** (APCI) *m/z* calculated for $C_{17}H_{26}BrFO^{\bullet+}$ $[M]^{\bullet+}$: 344.11456, found 344.11417.

***((11-Bromoundecyl)oxy)cyclohexane* (19d).** Cyclohexanol (2.55 mL, 24.5 mmol, 2.00 eq), HMPA (4.26 mL, 24.5 mmol, 2.00 eq), and anhydrous THF (31 mL, 0.4 M) were added to an oven-dried Schlenk flask equipped with a magnetic stir bar under an atmosphere of Ar. The solution was cooled to -78 $^{o}$C, then *n*-BuLi (2.5 M in hexanes, 9.54 mL, 23.9 mmol, 1.95 eq) was added dropwise. The reaction mixture was then slowly warmed to 0 $^{o}$C and stirred vigorously for 1 h. After returning the reaction mixture to -78 $^{o}$C, 11-bromoundecyl trifluoromethanesulfonate[3] (**18**, 4.69 g, 12.2 mmol, 1.00 eq) dissolved in anhydrous THF was added dropwise. Next, the reaction mixture was slowly warmed to 0 $^{o}$C and stirred vigorously for 5 h. Upon confirming complete conversion of starting material by TLC, the reaction mixture was slowly quenched with water. Subsequently, the aqueous layer was extracted with EtOAc (3x), and the collected organic phase was washed with water and brine, dried with $MgSO_4$, and concentrated under reduced pressure. Finally, the resulting crude material was purified by column chromatography eluting

along a gradient of 0-10% DCM in hexanes to yield a colorless oil (3.00 g, 9.00 mmol, 74% yield). **TLC** (2% EtOAc in hexanes) $R_f$ = 0.25. **$^1$H NMR** (600 MHz, $CDCl_3$) δ 3.42 (t, *J* = 6.8 Hz, 2H), 3.40 (t, *J* = 6.9 Hz, 2H), 3.23 – 3.14 (m, 1H), 1.94 – 1.81 (m, 4H), 1.78 – 1.68 (m, 2H), 1.59 – 1.49 (m, 3H), 1.46 – 1.37 (m, 2H), 1.37 – 1.14 (m, 17H). **$^{13}$C NMR** (151 MHz, $CDCl_3$) δ 77.6, 68.1, 34.2, 33.0, 32.6, 30.4, 29.7, 29.63, 29.62, 29.56, 28.9, 28.3, 26.4, 26.0, 24.5 (*15 out of 17 carbon signals observed due to chemical shift equivalence*). **HRMS** (APCI) *m/z* calculated for $C_{17}H_{33}BrO^{\bullet+}$ $[M]^{\bullet+}$: 332.17093, found 332.17071.

*Synthesis of Long-Chain Thioether-Containing Alcohols*

General Procedure A. Alkyl bromide (1.00 eq), cesium carbonate (2.00 eq), and Ar-degassed anhydrous DMF (0.2 M) were added to an oven-dried flask equipped with a magnetic stir bar under an atmosphere of Ar. While stirring the suspension vigorously at room temperature, thiol (2.00 eq) was added dropwise. The reaction mixture was then stirred at room temperature for 1-16 h. After confirming complete conversion of starting material by TLC, the reaction mixture was diluted with EtOAc (0.02 M). The collected organic phase was then washed with saturated aqueous $NH_4Cl$ (3x), dried with either $Na_2SO_4$ or $MgSO_4$, and concentrated under reduced pressure. Finally, the resulting crude material was purified by column chromatography to yield long-chain thioether-containing alcohols.

General Procedure B. Thiol (2.00 eq) and Ar-degassed anhydrous DMF (0.2 M) were added to an oven-dried flask equipped with a magnetic stir bar under an atmosphere of Ar. While stirring the solution vigorously at room temperature, 1,8-diazabicyclo[5.4.0]undec-7-ene (2.00 eq) was added dropwise. The mixture was stirred for 20 min, then alkyl halide (1.00 eq) dissolved in Ar-degassed anhydrous DMF was added dropwise at room temperature. The following reaction mixture was subsequently heated to 60-65 $^{o}$C and stirred for 1.5-4 h. After confirming complete conversion of

starting material by TLC, the reaction mixture was cooled to room temperature and diluted with EtOAc (0.02 M). The collected organic phase was then washed with saturated aqueous $NH_4Cl$ (3x), dried with either $Na_2SO_4$ or $MgSO_4$, and concentrated under reduced pressure. Lastly, the resulting crude material was either washed with cold hexanes or purified by column chromatography to yield the long-chain thioether-containing alcohol.

General Procedure C. Alcohol (1.00 eq), triethylamine (1.50 eq), and anhydrous DCM (0.4 M) were added to an oven-dried flask equipped with a magnetic stir bar under an atmosphere of Ar. After cooling the solution to 0 $^oC$, methanesulfonyl chloride (1.50 eq) was added dropwise. The following reaction mixture was stirred at 0 $^oC$ for 15 min, then allowed to warm to room temperature for 30 min. Upon confirming complete conversion of starting material by TLC, the reaction mixture was diluted with DCM. Next, the collected organic phase was washed with saturated aqueous $NaHCO_3$ and brine, dried with either $Na_2SO_4$ or $MgSO_4$, and concentrated under reduced pressure. The resulting crude material was then taken on immediately without further purification. 3-Mercaptopropanol (2.00 eq) and Ar-degassed anhydrous DMF (0.2 M) were added to a separate oven-dried flask equipped with a magnetic stir bar under an atmosphere of Ar. While stirring the solution vigorously at room temperature, 1,8-diazabicyclo[5.4.0]undec-7-ene (2.00 eq) was added dropwise. The reaction mixture was stirred for 20 min, then crude long-chain methanesulfonate (1.00 eq) dissolved in Ar-degassed anhydrous DMF was added dropwise at room temperature. Subsequently, the following reaction mixture was heated to 60-65 $^oC$ and stirred for 1.5-5 h. After confirming complete conversion of starting material by TLC, the reaction mixture was cooled to room temperature and diluted with EtOAc (0.02 M). The collected organic phase was then washed with saturated aqueous $NH_4Cl$ (3x), dried with either $Na_2SO_4$ or $MgSO_4$, and concentrated under reduced pressure. Finally, the resulting crude material was either washed with

cold hexanes or purified by column chromatography to yield the long-chain thioether-containing alcohol.

***(Heptadecylthio)ethan-1-ol* (7b).** Synthesis was carried out according to *General Procedure A for Long-Chain Thioether-Containing Alcohols* using 1-bromoheptadecane (2.50 g, 7.83 mmol, 1.00 eq) and 2-mercaptoethanol (1.10 mL, 15.7 mmol, 2.00 eq). Purified via column chromatography eluting along a gradient of 0-10% EtOAc in hexanes to yield a white powder (2.29 g, 7.23 mmol, 92% yield). MP = 60-62 $^{o}$C. **TLC** (20% EtOAc in hexanes) $R_f$ = 0.36. **$^{1}$H NMR** (400 MHz, $CDCl_3$) δ 3.71 (q, *J* = 6.1 Hz, 2H), 2.73 (t, *J* = 5.9 Hz, 2H), 2.54 – 2.48 (m, 2H), 2.17 (t, *J* = 6.2 Hz, 1H), 1.63 – 1.53 (m, 2H), 1.44 – 1.18 (m, 28H), 0.88 (app t, *J* = 6.9 Hz, 3H). **$^{13}$C NMR** (151 MHz, $CDCl_3$) δ 60.3, 35.5, 32.1, 31.8, 29.92, 29.85, 29.82, 29.81, 29.80, 29.75, 29.7, 29.5, 29.4, 29.0, 22.8, 14.3 (*16 out of 19 carbon signals observed due to chemical shift equivalence*). **HRMS** (APCI) *m/z* calculated for $C_{19}H_{41}OS^+$ $[M + H]^+$: 317.28726, found 317.28742.

***6-(Tridecylthio)hexan-1-ol* (7c).** Synthesis was carried out according to *General Procedure B for Long-Chain Thioether-Containing Alcohols* using 1-bromotridecane (2.00 g, 7.60 mmol, 1.00 eq) and 6-mercaptohexanol (2.08 mL, 15.2 mmol, 2.00 eq). Purified via column chromatography eluting along a gradient of 0-15% EtOAc in hexanes to yield a white powder (2.19 g, 6.93 mmol, 91% yield). MP = 51-53 $^{o}$C. **TLC** (20% EtOAc in hexanes) $R_f$ = 0.28. **$^{1}$H NMR** (500 MHz, $CDCl_3$) δ 3.65 (td, *J* = 6.5, 5.3 Hz, 2H), 2.53 – 2.47 (m, 4H), 1.64 – 1.53 (m, 6H), 1.46 – 1.33 (m, 6H), 1.33 – 1.22 (m, 18H), 1.20 (t, *J* = 5.3 Hz, 1H), 0.88 (app t, *J* = 7.0 Hz, 3H). **$^{13}$C NMR** (151 MHz, $CDCl_3$) δ 63.1, 32.8, 32.4, 32.2, 32.1, 29.9, 29.83, 29.80, 29.79, 29.78, 29.76, 29.7, 29.5, 29.4, 29.1, 28.8, 25.5, 22.8, 14.3. **HRMS** (APCI) *m/z* calculated for $C_{19}H_{41}OS^+$ $[M + H]^+$: 317.28726, found 317.28708.

***9-(Decylthio)nonan-1-ol*** **(7d).** Synthesis was carried out according to *General Procedure B for Long-Chain Thioether-Containing Alcohols* using 9-bromo-1-nonanol (2.00 g, 8.96 mmol, 1.00 eq) and 1-decanethiol (3.70 mL, 17.9 mmol, 2.00 eq). After extraction, crude material was washed with cold hexanes to yield a shiny white powder (2.15 g, 6.79 mmol, 76% yield). MP = 51-53 $^{o}$C. **TLC** (20% EtOAc in hexanes) $R_f$ = 0.30. **$^{1}$H NMR** (600 MHz, $CD_3OD$) δ 3.54 (t, *J* = 6.7 Hz, 2H), 2.52 – 2.47 (m, 4H), 1.61 – 1.49 (m, 6H), 1.44 – 1.24 (m, 24H), 0.90 (app t, *J* = 7.0 Hz, 3H). **$^{13}$C NMR** (151 MHz, $CD_3OD$) δ 63.0, 33.7, 33.1, 32.9, 30.80, 30.79, 30.69, 30.68, 30.66, 30.6, 30.5, 30.34, 30.31, 29.88, 29.87, 26.9, 23.7, 14.4 (*18 out of 19 carbon signals observed due to chemical shift equivalence*). **HRMS** (APCI) *m/z* calculated for $C_{19}H_{41}OS^+$ $[M + H]^+$: 317.28726, found 317.28731.

***12-(Heptylthio)dodecan-1-ol*** **(7e).** Synthesis was carried out according to *General Procedure B for Long-Chain Thioether-Containing Alcohols* using 12-bromo-1-dodecanol (2.25 g, 8.48 mmol, 1.00 eq) and 1-heptanethiol (2.66 mL, 17.0 mmol, 2.00 eq). Purified via column chromatography eluting along a gradient of 0-10% EtOAc in hexanes to yield a white powder (2.36 g, 7.46 mmol, 88% yield). MP = 50-53 $^{o}$C. **TLC** (20% EtOAc in hexanes) $R_f$ = 0.31. **$^{1}$H NMR** (500 MHz, $CDCl_3$) δ 3.64 (td, *J* = 6.6, 5.5 Hz, 2H), 2.53 – 2.46 (m, 4H), 1.61 – 1.52 (m, 6H), 1.41 – 1.23 (m, 24H), 1.21 (t, *J* = 5.5 Hz, 1H), 0.88 (app t, *J* = 7.0 Hz, 3H). **$^{13}$C NMR** (151 MHz, $CDCl_3$) δ 63.3, 33.0, 32.4, 31.9, 29.90, 29.89, 29.74, 29.72, 29.71, 29.67, 29.6, 29.4, 29.11, 29.08, 25.9, 22.8, 14.2 (*17 out of 19 carbon signals observed due to chemical shift equivalence*). **HRMS** (APCI) *m/z* calculated for $C_{19}H_{41}OS^+$ $[M + H]^+$: 317.28726, found 317.28699.

***15-(Butylthio)pentadecan-1-ol*** **(7f).** Synthesis was carried out according to *General Procedure B for Long-Chain Thioether-Containing Alcohols* using 15-bromo-1-pentadecanol[4] (2.50 g, 8.14 mmol, 1.00 eq) and 1-butanethiol (1.74 mL, 16.3 mmol, 2.00 eq). Purified via column

chromatography eluting along a gradient of 0-15% EtOAc in hexanes to yield a white powder (2.31 g, 7.30 mmol, 90% yield). MP = 53-55 $^{o}$C. **TLC** (20% EtOAc in hexanes) $R_f$ = 0.30. **$^{1}$H NMR** (500 MHz, $CDCl_3$) δ 3.64 (td, *J* = 6.6, 5.5 Hz, 2H), 2.53 – 2.47 (m, 4H), 1.61 – 1.52 (m, 6H), 1.45 – 1.23 (m, 24H), 1.20 (t, *J* = 5.5 Hz, 1H), 0.91 (app t, *J* = 7.3 Hz, 3H). **$^{13}$C NMR** (151 MHz, $CDCl_3$) δ 63.3, 33.0, 32.4, 32.01, 31.99, 29.9, 29.79, 29.76, 29.75, 29.74, 29.68, 29.6, 29.4, 29.1, 25.9, 22.2, 13.9 (*17 out of 19 carbon signals observed due to chemical shift equivalence*). **HRMS** (APCI) *m/z* calculated for $C_{19}H_{41}OS^+$ [M + H]$^+$: 317.28726, found 317.28678.

***3-(Dodecylthio)propan-1-ol* (7g).** Synthesis was carried out according to *General Procedure B for Long-Chain Thioether-Containing Alcohols* using 1-iodododecane (2.50 g, 8.44 mmol, 1.00 eq) and 3-mercaptopropanol (1.46 mL, 16.9 mmol, 2.00 eq). Purified via column chromatography eluting along a gradient of 0-15% EtOAc in hexanes to yield a white powder (1.92 g, 7.38 mmol, 87% yield). MP = 37-40 $^{o}$C. **TLC** (20% EtOAc in hexanes) $R_f$ = 0.26. **$^{1}$H NMR** (400 MHz, $CDCl_3$) δ 3.77 (q, *J* = 5.9 Hz, 2H), 2.64 (t, *J* = 7.0 Hz, 2H), 2.56 – 2.49 (m, 2H), 1.85 (tt, *J* = 7.0, 6.0 Hz, 2H), 1.65 (t, *J* = 5.5 Hz, 1H), 1.63 – 1.53 (m, 2H), 1.42 – 1.19 (m, 18H), 0.88 (app t, *J* = 6.9 Hz, 3H). **$^{13}$C NMR** (151 MHz, $CDCl_3$) δ 62.2, 32.3, 32.06, 32.05, 29.80, 29.78, 29.75, 29.7, 29.5, 29.4, 29.09, 29.07, 22.8, 14.3 (*14 out of 15 carbon signals observed due to chemical shift equivalence*). **HRMS** (APCI) *m/z* calculated for $C_{15}H_{33}OS^+$ [M + H]$^+$: 261.22466, found 261.22446.

***3-(Tetradecylthio)propan-1-ol* (7h).** Synthesis was carried out according to *General Procedure B for Long-Chain Thioether-Containing Alcohols* using 1-bromotetradecane (2.50 g, 9.02 mmol, 1.00 eq) and 3-mercaptopropanol (1.56 mL, 18.0 mmol, 2.00 eq). Purified via column chromatography eluting along a gradient of 0-15% EtOAc in hexanes to yield a white powder (2.57 g, 8.90 mmol, >95% yield). MP = 46-49 $^{o}$C. **TLC** (20% EtOAc in hexanes) $R_f$ = 0.27. **$^{1}$H NMR** (400 MHz, $CDCl_3$) δ 3.77 (q, *J* = 5.9 Hz, 2H), 2.64 (t, *J* = 7.0 Hz, 2H), 2.56 – 2.49 (m, 2H),

1.85 (tt, $J$ = 7.0, 6.0 Hz, 2H), 1.64 (t, $J$ = 5.5 Hz, 1H), 1.62 – 1.53 (m, 2H), 1.42 – 1.19 (m, 22H), 0.88 (app t, $J$ = 6.9 Hz, 3H). **$^{13}$C NMR** (151 MHz, $CDCl_3$) δ 62.2, 32.3, 32.07, 32.05, 29.83, 29.82, 29.80, 29.75, 29.7, 29.5, 29.4, 29.09, 29.07, 22.8, 14.3 (*15 out of 17 carbon signals observed due to chemical shift equivalence*). **HRMS** (APCI) *m/z* calculated for $C_{17}H_{37}OS^+$ [M + H]$^+$: 289.25596, found 289.25553.

***3-(Octadecylthio)propan-1-ol* (7i).** Synthesis was carried out according to *General Procedure B for Long-Chain Thioether-Containing Alcohols* using 1-bromooctadecane (2.50 g, 7.50 mmol, 1.00 eq) and 3-mercaptopropanol (1.30 mL, 15.0 mmol, 2.00 eq). Purified via column chromatography eluting along a gradient of 0-15% EtOAc in hexanes to yield a white powder (2.34 g, 6.78 mmol, 90% yield). MP = 60-62 $^o$C. **TLC** (20% EtOAc in hexanes) $R_f$ = 0.32. **$^1$H NMR** (400 MHz, $CDCl_3$) δ 3.77 (q, $J$ = 5.9 Hz, 2H), 2.64 (t, $J$ = 7.0 Hz, 2H), 2.56 – 2.49 (m, 2H), 1.86 (tt, $J$ = 7.0, 6.0 Hz, 2H), 1.62 (t, $J$ = 5.5 Hz, 1H), 1.63 – 1.54 (m, 2H), 1.42 – 1.17 (m, 30H), 0.88 (app t, $J$ = 6.9 Hz, 3H). **$^{13}$C NMR** (151 MHz, $CDCl_3$) δ 62.2, 32.3, 32.08, 32.05, 29.85, 29.83, 29.81, 29.76, 29.75, 29.7, 29.5, 29.4, 29.09, 29.07, 22.8, 14.3 (*16 out of 21 carbon signals observed due to chemical shift equivalence*). **HRMS** (NSI) *m/z* calculated for $C_{21}H_{45}OS^+$ [M + H]$^+$: 345.31856, found 345.31797.

***3-(Icosylthio)propan-1-ol* (7j).** Synthesis was carried out according to *General Procedure B for Long-Chain Thioether-Containing Alcohols* using 1-bromoicosane (2.50 g, 6.92 mmol, 1.00 eq) and 3-mercaptopropanol (1.19 mL, 13.8 mmol, 2.00 eq). Purified via column chromatography eluting along a gradient of 0-10% EtOAc in hexanes to yield a white powder (2.12 g, 5.70 mmol, 82% yield). MP = 65-67 $^o$C. **TLC** (20% EtOAc in hexanes) $R_f$ = 0.32. **$^1$H NMR** (400 MHz, $CDCl_3$) δ 3.77 (q, $J$ = 5.9 Hz, 2H), 2.64 (t, $J$ = 7.0 Hz, 2H), 2.56 – 2.49 (m, 2H), 1.86 (tt, $J$ = 7.0, 6.0 Hz, 2H), 1.62 (t, $J$ = 5.5 Hz, 1H), 1.63 – 1.54 (m, 2H), 1.42 – 1.18 (m, 34H), 0.88 (app t, $J$ = 6.9 Hz,

3H). $^{13}$C NMR (151 MHz, $CDCl_3$) δ 62.2, 32.3, 32.08, 32.05, 29.9, 29.83, 29.81, 29.76, 29.76, 29.7, 29.5, 29.4, 29.10, 29.08, 22.9, 14.3 (*16 out of 23 carbon signals observed due to chemical shift equivalence*). **HRMS** (APCI) *m/z* calculated for $C_{23}H_{49}OS^+$ $[M + H]^+$: 373.34986, found 373.34964.

***3-((16-(Trimethylsilyl)hexadec-15-yn-1-yl)thio)propan-1-ol* (13a).** Synthesis was carried out according to *General Procedure C for Long-Chain Thioether-Containing Alcohols* using compound **11a** (2.53 g, 8.14 mmol, 1.00 eq). Purified via column chromatography eluting along a gradient of 0-10% EtOAc in hexanes to yield a colorless oil (2.67 g, 6.93 mmol, 85% yield over two steps). **TLC** (20% EtOAc in hexanes) $R_f$ = 0.30. **$^1$H NMR** (400 MHz, $CDCl_3$) δ 3.77 (q, *J* = 5.9 Hz, 2H), 2.63 (t, *J* = 7.0 Hz, 2H), 2.56 – 2.49 (m, 2H), 2.21 (t, *J* = 7.2 Hz, 2H), 1.85 (tt, *J* = 7.0, 6.0 Hz, 2H), 1.64 (t, *J* = 5.5 Hz, 1H), 1.62 – 1.46 (m, 4H), 1.42 – 1.20 (m, 20H), 0.14 (s, 9H). **$^{13}$C NMR** (151 MHz, $CDCl_3$) δ 108.0, 84.4, 62.2, 32.3, 32.1, 29.79, 29.75, 29.74, 29.68, 29.6, 29.4, 29.2, 29.09, 29.08, 29.0, 28.8, 20.0, 0.3 (*18 out of 22 carbon signals observed due to chemical shift equivalence*). **HRMS** (APCI) *m/z* calculated for $C_{22}H_{45}OSSi^+$ $[M + H]^+$: 385.29549, found 385.29507.

***3-(Hexadec-15-yn-1-ylthio)propan-1-ol* (13b).** Compound **13a** (2.21 g, 5.75 mmol, 1.00 eq) and anhydrous THF (38 mL, 0.15 M) were added to an oven-dried flask equipped with a magnetic stir bar under an atmosphere of Ar. While stirring the solution vigorously at room temperature, tetrabutylammonium fluoride (1 M in THF, 11.5 mL, 11.5 mmol, 2.00 eq) was added dropwise. The reaction mixture was then stirred at room temperature for 45 min. After confirming complete conversion of starting material by TLC, the reaction mixture was diluted with EtOAc. The collected organic phase was subsequently washed with saturated aqueous $NH_4Cl$ (2x), dried with $Na_2SO_4$, and concentrated under reduced pressure. Finally, the resulting crude material was

purified by column chromatography eluting along a gradient of 0-20% EtOAc in hexanes to yield a white powder (1.56 g, 5.00 mmol, 87% yield). MP = 51-54 ºC. **TLC** (20% EtOAc in hexanes) $R_f$ = 0.28. **$^1$H NMR** (400 MHz, $CDCl_3$) δ 3.77 (q, *J* = 5.9 Hz, 2H), 2.64 (t, *J* = 7.0 Hz, 2H), 2.56 – 2.49 (m, 2H), 2.18 (td, *J* = 7.1, 2.6 Hz, 2H), 1.94 (t, *J* = 2.6 Hz, 1H), 1.85 (tt, *J* = 7.0, 6.0 Hz, 2H), 1.63 (t, *J* = 5.5 Hz, 1H), 1.63 – 1.47 (m, 4H), 1.44 – 1.19 (m, 20H). **$^{13}$C NMR** (151 MHz, $CDCl_3$) δ 85.0, 68.2, 62.2, 32.3, 32.1, 29.8, 29.74, 29.67, 29.65, 29.4, 29.3, 29.08, 29.07, 28.9, 28.7, 18.6 (*16 out of 19 carbon signals observed due to chemical shift equivalence*). **HRMS** (APCI) *m/z* calculated for $C_{19}H_{37}OS^+$ $[M + H]^+$: 313.25596, found 313.25559.

***3-((14-(Trimethylsilyl)tetradec-13-yn-1-yl)thio)propan-1-ol* (13c).** Synthesis was carried out according to *General Procedure C for Long-Chain Thioether-Containing Alcohols* using compound **11c** (2.06 g, 7.29 mmol, 1.00 eq). Purified via column chromatography eluting along a gradient of 0-10% EtOAc in hexanes to yield a colorless oil (2.22 g, 6.22 mmol, 85% yield over two steps). **TLC** (20% EtOAc in hexanes) $R_f$ = 0.27. **$^1$H NMR** (400 MHz, $CDCl_3$) δ 3.77 (q, *J* = 5.9 Hz, 2H), 2.64 (t, *J* = 7.0 Hz, 2H), 2.56 – 2.49 (m, 2H), 2.21 (t, *J* = 7.2 Hz, 2H), 1.85 (tt, *J* = 7.1, 6.1 Hz, 2H), 1.64 (t, *J* = 5.5 Hz, 1H), 1.62 – 1.46 (m, 4H), 1.42 – 1.21 (m, 16H), 0.14 (s, 9H). **$^{13}$C NMR** (151 MHz, $CDCl_3$) δ 107.9, 84.4, 62.2, 32.3, 32.1, 29.74, 29.72, 29.70, 29.67, 29.6, 29.4, 29.2, 29.08, 29.07, 28.9, 28.8, 20.0, 0.3 (*18 out of 20 carbon signals observed due to chemical shift equivalence*). **HRMS** (APCI) *m/z* calculated for $C_{20}H_{41}OSSi^+$ $[M + H]^+$: 357.26419, found 357.26398.

***3-((13-(Triisopropylsilyl)tridec-12-yn-1-yl)thio)propan-1-ol* (13d).** Synthesis was carried out according to *General Procedure C for Long-Chain Thioether-Containing Alcohols* using compound **11d** (3.47 g, 9.84 mmol, 1.00 eq). Purified via column chromatography eluting along a gradient of 0-10% EtOAc in hexanes to yield a colorless oil (2.87 g, 6.72 mmol, 68% yield over

two steps). **TLC** (20% EtOAc in hexanes) $R_f$ = 0.32. **$^1$H NMR** (600 MHz, $CD_3OD$) δ 3.63 (t, *J* = 6.3 Hz, 2H), 2.58 (app t, *J* = 7.3 Hz, 2H), 2.51 (app t, *J* = 7.3 Hz, 2H), 2.27 (t, *J* = 6.5 Hz, 2H), 1.81 – 1.75 (m, 2H), 1.61 – 1.55 (m, 2H), 1.55 – 1.44 (m, 4H), 1.43 – 1.36 (m, 2H), 1.36 – 1.28 (m, 10H), 1.10 – 0.96 (m, 21H). **$^{13}$C NMR** (151 MHz, $CD_3OD$) δ 110.7, 80.8, 61.6, 33.6, 32.9, 30.8, 30.7, 30.6, 30.4, 30.1, 29.9, 29.8, 29.6, 29.3, 20.3, 19.1, 12.5 (*17 out of 25 carbon signals observed due to chemical shift equivalence*). **HRMS** (APCI) *m/z* calculated for $C_{25}H_{51}OSSi^+$ $[M + H]^+$: 427.34244, found 427.34240.

***3-((15,15-Dimethylhexadec-13-yn-1-yl)thio)propan-1-ol*** **(13e).** Synthesis was carried out according to *General Procedure C for Long-Chain Thioether-Containing Alcohols* using compound **11e** (2.42 g, 9.08 mmol, 1.00 eq). Purified via column chromatography eluting along a gradient of 0-10% EtOAc in hexanes to yield a colorless oil (2.19 g, 6.43 mmol, 71% yield over two steps). **TLC** (20% EtOAc in hexanes) $R_f$ = 0.26. **$^1$H NMR** (400 MHz, $CD_3OD$) δ 3.63 (t, *J* = 6.3 Hz, 2H), 2.61 – 2.55 (m, 2H), 2.54 – 2.48 (m, 2H), 2.11 (t, *J* = 6.7 Hz, 2H), 1.82 – 1.73 (m, 2H), 1.63 – 1.52 (m, 2H), 1.49 – 1.27 (m, 18H), 1.17 (s, 9H). **$^{13}$C NMR** (151 MHz, $CD_3OD$) δ 89.7, 79.5, 61.6, 33.6, 32.9, 31.9, 30.8, 30.69, 30.65, 30.4, 30.23, 30.18, 29.9, 29.7, 29.3, 28.3, 19.3 (*17 out of 21 carbon signals observed due to chemical shift equivalence*). **HRMS** (APCI) *m/z* calculated for $C_{21}H_{41}OS^+$ $[M + H]^+$: 341.28726, found 341.28680.

***3-((15,15-Dimethylhexadecyl)thio)propan-1-ol*** **(14e).** Synthesis was carried out according to *General Procedure C for Long-Chain Thioether-Containing Alcohols* using compound **12e** (1.71 g, 6.32 mmol, 1.00 eq). Purified via column chromatography eluting along a gradient of 0-10% EtOAc in hexanes to yield a white powder (1.72 g, 4.99 mmol, 79% yield over two steps). MP = 33-36 $^o$C. **TLC** (20% EtOAc in hexanes) $R_f$ = 0.29. **$^1$H NMR** (400 MHz, $CD_3OD$) δ 3.63 (t, *J* = 6.3 Hz, 2H), 2.61 – 2.55 (m, 2H), 2.54 – 2.48 (m, 2H), 1.83 – 1.73 (m, 2H), 1.63 – 1.53 (m,

2H), 1.45 – 1.14 (m, 24H), 0.88 (s, 9H). **$^{13}$C NMR** (151 MHz, $CD_3OD$) δ 61.6, 45.5, 33.6, 32.9, 31.8, 31.1, 30.81, 30.78, 30.77, 30.75, 30.71, 30.68, 30.4, 29.92, 29.85, 29.3, 25.7 (*17 out of 21 carbon signals observed due to chemical shift equivalence*). **HRMS** (APCI) *m/z* calculated for $C_{21}H_{45}OS^+$ [M + H]$^+$: 345.31856, found 345.31805.

***3-((12-Phenyldodec-11-yn-1-yl)thio)propan-1-ol*** **(13f).** Synthesis was carried out according to *General Procedure C for Long-Chain Thioether-Containing Alcohols* using compound **11f** (2.61 g, 10.1 mmol, 1.00 eq). Purified via column chromatography eluting along a gradient of 0-10% EtOAc in hexanes to yield a white crystalline solid (3.09 g, 9.31 mmol, 92% yield over two steps). MP = 30-32 $^o$C. **TLC** (25% EtOAc in hexanes) $R_f$ = 0.25. **$^1$H NMR** (600 MHz, $CD_3OD$) δ 7.36 – 7.31 (m, 2H), 7.30 – 7.24 (m, 3H), 3.63 (t, *J* = 6.3 Hz, 2H), 2.59 – 2.55 (m, 2H), 2.53 – 2.48 (m, 2H), 2.40 (t, *J* = 7.0 Hz, 2H), 1.81 – 1.74 (m, 2H), 1.63 – 1.54 (m, 4H), 1.52 – 1.44 (m, 2H), 1.43 – 1.28 (m, 10H). **$^{13}$C NMR** (151 MHz, $CD_3OD$) δ 132.4, 129.3, 128.5, 125.6, 90.9, 81.6, 61.6, 33.6, 32.9, 30.8, 30.59, 30.57, 30.3, 30.2, 29.93, 29.91, 29.88, 29.3, 20.0 (*19 out of 21 carbon signals observed due to chemical shift equivalence*). **HRMS** (APCI) *m/z* calculated for $C_{21}H_{33}OS^+$ [M + H]$^+$: 333.22466, found 333.22375.

***3-((12-Phenyldodecyl)thio)propan-1-ol*** **(14f).** Synthesis was carried out according to *General Procedure C for Long-Chain Thioether-Containing Alcohols* using compound **12f** (2.09 g, 7.95 mmol, 1.00 eq). Purified via column chromatography eluting along a gradient of 0-10% EtOAc in hexanes to yield a white powder (2.37 g, 7.06 mmol, 89% yield over two steps). MP = 41-44 $^o$C. **TLC** (25% EtOAc in hexanes) $R_f$ = 0.29. **$^1$H NMR** (600 MHz, $CD_3OD$) δ 7.26 – 7.21 (m, 2H), 7.17 – 7.11 (m, 3H), 3.63 (t, *J* = 6.3 Hz, 2H), 2.62 – 2.55 (m, 4H), 2.53 – 2.49 (m, 2H), 1.81 – 1.74 (m, 2H), 1.65 – 1.54 (m, 4H), 1.43 – 1.36 (m, 2H), 1.36 – 1.25 (m, 14H). **$^{13}$C NMR** (151 MHz, $CD_3OD$) δ 144.0, 129.4, 129.2, 126.6, 61.6, 36.9, 33.6, 32.9, 32.8, 30.8, 30.69, 30.67, 30.66, 30.6,

30.34, 30.31, 29.9, 29.3 (*18 out of 21 carbon signals observed due to chemical shift equivalence*). **HRMS** (APCI) *m/z* calculated for $C_{21}H_{37}OS^+$ $[M + H]^+$: 337.25596, found 337.25563.

***3-((11-Phenoxyundecyl)thio)propan-1-ol* (22a).** Synthesis was carried out according to *General Procedure A for Long-Chain Thioether-Containing Alcohols* using compound **19a** (2.75 g, 8.40 mmol, 1.00 eq) and 3-mercaptopropanol (1.45 mL, 16.8 mmol, 2.00 eq). Purified via column chromatography eluting along a gradient of 0-15% EtOAc in hexanes to yield a white powder (2.54 g, 7.51 mmol, 89% yield). MP = 49-51 $^oC$. **TLC** (25% EtOAc in hexanes) $R_f$ = 0.26. **$^1$H NMR** (400 MHz, $CD_3OD$) δ 7.28 – 7.20 (m, 2H), 6.92 – 6.85 (m, 3H), 3.95 (t, *J* = 6.4 Hz, 2H), 3.63 (t, *J* = 6.3 Hz, 2H), 2.61 – 2.54 (m, 2H), 2.54 – 2.48 (m, 2H), 1.82 – 1.71 (m, 4H), 1.63 – 1.53 (m, 2H), 1.52 – 1.27 (m, 14H). **$^{13}$C NMR** (151 MHz, $CD_3OD$) δ 160.6, 130.4, 121.5, 115.5, 68.9, 61.6, 33.6, 32.9, 30.8, 30.7, 30.63, 30.62, 30.5, 30.4, 30.3, 29.9, 29.3, 27.2 (*18 out of 20 carbon signals observed due to chemical shift equivalence*). **HRMS** (APCI) *m/z* calculated for $C_{20}H_{35}O_2S^+$ $[M + H]^+$: 339.23523, found 339.23517.

***11-(Phenylthio)undecan-1-ol* (20).** Synthesis was carried out according to *General Procedure A for Long-Chain Thioether-Containing Alcohols* using compound **17** (3.00 g, 11.9 mmol, 1.00 eq) and thiophenol (2.44 mL, 23.9 mmol, 2.00 eq). Purified via column chromatography eluting along a gradient of 0-15% EtOAc in hexanes to yield a white powder (3.19 g, 11.4 mmol, 95% yield). MP = 68-71 $^oC$. **TLC** (25% EtOAc in hexanes) $R_f$ = 0.27. **$^1$H NMR** (400 MHz, $CD_3OD$) δ 7.34 – 7.24 (m, 4H), 7.18 – 7.12 (m, 1H), 3.53 (t, *J* = 6.7 Hz, 2H), 2.95 – 2.88 (m, 2H), 1.66 – 1.57 (m, 2H), 1.56 – 1.48 (m, 2H), 1.47 – 1.24 (m, 14H). **$^{13}$C NMR** (126 MHz, $CD_3OD$) δ 138.3, 130.1, 129.9, 126.7, 63.0, 34.3, 33.7, 30.7, 30.61, 30.59, 30.3, 30.2, 29.7, 26.9 (*14 out of 17 carbon signals observed due to chemical shift equivalence*). **HRMS** (APCI) *m/z* calculated for $C_{17}H_{29}OS^+$ $[M + H]^+$: 281.19336, found 281.19300.

***3-((11-(Phenylthio)undecyl)thio)propan-1-ol* (22b).** Synthesis was carried out according to *General Procedure C for Long-Chain Thioether-Containing Alcohols* using compound **20** (1.00 g, 3.57 mmol 1.00 eq). After extraction, crude material was washed with cold hexanes to yield a white powder (858 mg, 2.42 mmol, 68% yield over two steps). MP = 64-66 $^oC$. **TLC** (25% EtOAc in hexanes) $R_f$ = 0.23. **$^1$H NMR** (400 MHz, $CD_3OD$) δ 7.34 – 7.24 (m, 4H), 7.18 – 7.12 (m, 1H), 3.63 (t, *J* = 6.3 Hz, 2H), 2.95 – 2.88 (m, 2H), 2.61 – 2.54 (m, 2H), 2.54 – 2.47 (m, 2H), 1.83 – 1.72 (m, 2H), 1.67 – 1.51 (m, 4H), 1.49 – 1.22 (m, 14H). **$^{13}$C NMR** (126 MHz, $CD_3OD$) δ 138.3, 130.1, 129.9, 126.7, 61.6, 34.3, 33.6, 32.9, 30.8, 30.62, 30.57, 30.55, 30.32, 30.27, 30.2, 29.9, 29.7, 29.3 (*18 out of 20 carbon signals observed due to chemical shift equivalence*). **HRMS** (APCI) *m/z* calculated for $C_{20}H_{35}OS_2^+$ $[M + H]^+$: 355.21238, found 355.21193.

***3-((12-(4-Fluorophenyl)dodec-11-yn-1-yl)thio)propan-1-ol* (13g).** Synthesis was carried out according to *General Procedure C for Long-Chain Thioether-Containing Alcohols* using compound **11g** (1.79 g, 6.48 mmol, 1.00 eq). Purified via column chromatography eluting along a gradient of 0-15% EtOAc in hexanes to yield a white powder (1.95 g, 5.56 mmol, 86% yield over two steps). MP = 41-44 $^oC$. **TLC** (25% EtOAc in hexanes) $R_f$ = 0.28. **$^1$H NMR** (400 MHz, $CD_3OD$) δ 7.40 – 7.32 (m, 2H), 7.06 – 6.99 (m, 2H), 3.63 (t, *J* = 6.3 Hz, 2H), 2.60 – 2.54 (m, 2H), 2.54 – 2.47 (m, 2H), 2.39 (t, *J* = 7.0 Hz, 2H), 1.82 – 1.73 (m, 2H), 1.64 – 1.52 (m, 4H), 1.52 – 1.28 (m, 12H). **$^{13}$C NMR** (151 MHz, $CD_3OD$) δ 163.4 (d, $J_{CF}$ = 246.6 Hz), 134.4 (d, $J_{CF}$ = 8.3 Hz), 121.8 (d, $J_{CF}$ = 3.7 Hz), 116.3 (d, $J_{CF}$ = 22.2 Hz), 90.7 (d, $J_{CF}$ = 1.6 Hz), 80.5, 61.6, 33.6, 32.9, 30.8, 30.59, 30.56, 30.3, 30.2, 29.93, 29.90, 29.8, 29.3, 19.9 (*19 out of 21 carbon signals observed due to chemical shift equivalence*). **HRMS** (APCI) *m/z* calculated for $C_{21}H_{32}FOS^+$ $[M + H]^+$: 351.21524, found 351.21518.

***3-((12-(4-Fluorophenyl)dodecyl)thio)propan-1-ol* (14g).** Synthesis was carried out according to *General Procedure C for Long-Chain Thioether-Containing Alcohols* using compound **12g** (1.90 g, 6.78 mmol, 1.00 eq). Purified via column chromatography eluting along a gradient of 0-15% EtOAc in hexanes to yield a white powder (2.05 g, 5.78 mmol, 85% yield over two steps). MP = 40-42 $^oC$. **TLC** (25% EtOAc in hexanes) $R_f$ = 0.32. **$^1$H NMR** (400 MHz, $CD_3OD$) δ 7.19 – 7.13 (m, 2H), 7.00 – 6.92 (m, 2H), 3.63 (t, *J* = 6.3 Hz, 2H), 2.62 – 2.54 (m, 4H), 2.54 – 2.48 (m, 2H), 1.82 – 1.73 (m, 2H), 1.64 – 1.52 (m, 4H), 1.44 – 1.23 (m, 16H). **$^{13}$C NMR** (151 MHz, $CD_3OD$) δ 162.6 (d, $J_{CF}$ = 241.6 Hz), 139.9 (d, $J_{CF}$ = 3.0 Hz), 130.9 (d, $J_{CF}$ = 7.7 Hz), 115.7 (d, $J_{CF}$ = 21.4 Hz), 61.6, 36.0, 33.6, 32.9, 32.8, 30.8, 30.68, 30.67, 30.65, 30.5, 30.3, 30.2, 29.9, 29.3 (*18 out of 21 carbon signals observed due to chemical shift equivalence*). **HRMS** (APCI) *m/z* calculated for $C_{21}H_{36}FOS^+$ $[M + H]^+$: 355.24654, found 355.24669.

***3-((11-(4-Fluorophenoxy)undecyl)thio)propan-1-ol* (22c).** Synthesis was carried out according to *General Procedure A for Long-Chain Thioether-Containing Alcohols* using compound **19c** (3.00 g, 8.69 mmol, 1.00 eq) and 3-mercaptopropanol (1.50 mL, 17.4 mmol, 2.00 eq). Purified via column chromatography eluting along a gradient of 0-15% EtOAc in hexanes to yield a white powder (2.93 g, 8.22 mmol, 95% yield). MP = 51-53 $^oC$. **TLC** (25% EtOAc in hexanes) $R_f$ = 0.26. **$^1$H NMR** (600 MHz, $CD_3OD$) δ 7.00 – 6.94 (m, 2H), 6.90 – 6.84 (m, 2H), 3.92 (t, *J* = 6.5 Hz, 2H), 3.63 (t, *J* = 6.3 Hz, 2H), 2.57 (app t, *J* = 7.3 Hz, 2H), 2.51 (app t, *J* = 7.3 Hz, 2H), 1.81 – 1.71 (m, 4H), 1.61 – 1.54 (m, 2H), 1.50 – 1.43 (m, 2H), 1.43 – 1.27 (m, 12H). **$^{13}$C NMR** (151 MHz, $CD_3OD$) δ 158.5 (d, $J_{CF}$ = 236.1 Hz), 156.9 (d, $J_{CF}$ = 2.2 Hz), 116.61 (d, $J_{CF}$ = 8.3 Hz), 116.56 (d, $J_{CF}$ = 23.2 Hz), 69.6, 61.6, 33.6, 32.9, 30.8, 30.7, 30.63, 30.61, 30.5, 30.4, 30.3, 29.9, 29.3, 27.1 (*18 out of 20 carbon signals observed due to chemical shift equivalence*). **HRMS** (APCI) *m/z* calculated for $C_{20}H_{34}FO_2S^+$ $[M + H]^+$: 357.22581, found 357.22523.

***3-((12-Cyclohexyldodec-11-yn-1-yl)thio)propan-1-ol* (13h).** Synthesis was carried out according to *General Procedure C for Long-Chain Thioether-Containing Alcohols* using compound **11h** (2.98 g, 11.3 mmol, 1.00 eq). Purified via column chromatography eluting along a gradient of 0-15% EtOAc in hexanes to yield a colorless oil (3.43 g, 10.1 mmol, 90% yield over two steps). **TLC** (20% EtOAc in hexanes) $R_f$ = 0.26. **$^1$H NMR** (400 MHz, $CD_3OD$) δ 3.63 (t, *J* = 6.3 Hz, 2H), 2.61 – 2.55 (m, 2H), 2.54 – 2.48 (m, 2H), 2.38 – 2.26 (m, 1H), 2.13 (td, *J* = 6.7, 2.2 Hz, 2H), 1.83 – 1.65 (m, 6H), 1.63 – 1.53 (m, 2H), 1.53 – 1.25 (m, 20H). **$^{13}$C NMR** (151 MHz, $CD_3OD$) δ 85.3, 80.9, 61.6, 34.4, 33.6, 32.9, 30.8, 30.60, 30.58, 30.33, 30.30, 30.26, 30.2, 29.9, 29.8, 29.3, 27.1, 25.9, 19.4 (*19 out of 21 carbon signals observed due to chemical shift equivalence*). **HRMS** (APCI) *m/z* calculated for $C_{21}H_{39}OS^+$ $[M + H]^+$: 339.27161, found 339.27173.

***3-((12-Cyclohexyldodecyl)thio)propan-1-ol* (14h).** Synthesis was carried out according to *General Procedure C for Long-Chain Thioether-Containing Alcohols* using compound **12h** (1.88 g, 7.00 mmol, 1.00 eq). Purified via column chromatography eluting along a gradient of 0-15% EtOAc in hexanes to yield a white powder (2.02 g, 5.90 mmol, 84% yield over two steps). MP = 39-41 $^o$C. **TLC** (20% EtOAc in hexanes) $R_f$ = 0.27. **$^1$H NMR** (400 MHz, $CD_3OD$) δ 3.63 (t, *J* = 6.3 Hz, 2H), 2.61 – 2.55 (m, 2H), 2.54 – 2.48 (m, 2H), 1.82 – 1.62 (m, 7H), 1.62 – 1.53 (m, 2H), 1.48 – 1.10 (m, 24H), 0.96 – 0.80 (m, 2H). **$^{13}$C NMR** (151 MHz, $CD_3OD$) δ 61.6, 39.0, 38.8, 34.7, 33.6, 32.9, 31.1, 30.77, 30.75, 30.74, 30.70, 30.67, 30.4, 29.9, 29.3, 28.0, 27.9, 27.6 (*18 out of 21 carbon signals observed due to chemical shift equivalence*). **HRMS** (APCI) *m/z* calculated for $C_{21}H_{43}OS^+$ $[M + H]^+$: 343.30291, found 343.30304.

***3-((11-(Cyclohexyloxy)undecyl)thio)propan-1-ol* (22d).** Synthesis was carried out according to *General Procedure A for Long-Chain Thioether-Containing Alcohols* using compound **19d** (3.00 g, 9.00 mmol, 1.00 eq) and 3-mercaptopropanol (1.55 mL, 18.0 mmol, 2.00 eq). Purified via

column chromatography eluting along a gradient of 0-10% EtOAc in hexanes to yield a colorless oil (2.77 g, 8.04 mmol, 89% yield). **TLC** (25% EtOAc in hexanes) $R_f = 0.32$. **$^1$H NMR** (400 MHz, $CD_3OD$) δ 3.63 (t, *J* = 6.3 Hz, 2H), 3.46 (t, *J* = 6.6 Hz, 2H), 3.29 – 3.21 (m, 1H), 2.61 – 2.55 (m, 2H), 2.54 – 2.48 (m, 2H), 1.97 – 1.85 (m, 2H), 1.82 – 1.68 (m, 4H), 1.63 – 1.49 (m, 5H), 1.45 – 1.16 (m, 19H). **$^{13}$C NMR** (151 MHz, $CD_3OD$) δ 78.9, 69.0, 61.6, 33.6, 33.4, 32.9, 31.2, 30.8, 30.7, 30.63, 30.62, 30.55, 30.3, 29.9, 29.3, 27.3, 27.0, 25.2 (*18 out of 20 carbon signals observed due to chemical shift equivalence*). **HRMS** (APCI) *m/z* calculated for $C_{20}H_{41}O_2S^+$ $[M + H]^+$: 345.28218, found 345.28183.

***Methyl 16-((3-hydroxypropyl)thio)hexadecanoate* (22e).** Synthesis was carried out according to *General Procedure B for Long-Chain Thioether-Containing Alcohols* using 16-bromohexa-decanoate (**21**, 4.75 g, 13.6 mmol, 1.00 eq) and 3-mercaptopropanol (2.35 mL, 27.2 mmol, 2.00 eq). Purified via column chromatography eluting along a gradient of 0-40% EtOAc in hexanes to yield a white powder (4.29 g, 11.9 mmol, 88% yield). MP = 56-59 $^{o}$C. **TLC** (30% EtOAc in hexanes) $R_f = 0.31$. **$^1$H NMR** (400 MHz, $CDCl_3$) δ 3.76 (t, *J* = 6.0 Hz, 2H), 3.66 (s, 3H), 2.63 (t, *J* = 7.0 Hz, 2H), 2.56 – 2.48 (m, 2H), 2.30 (app t, *J* = 7.6 Hz, 2H), 1.85 (tt, *J* = 7.0, 6.0 Hz, 2H), 1.71 – 1.53 (m, 5H), 1.43 – 1.19 (m, 22H). **$^{13}$C NMR** (151 MHz, $CDCl_3$) δ 174.5, 62.2, 51.6, 34.3, 32.3, 32.1, 29.8, 29.74, 29.73, 29.67, 29.6, 29.40, 29.39, 29.3, 29.08, 29.07, 25.1 (*17 out of 20 carbon signals observed due to chemical shift equivalence*). **HRMS** (NSI) *m/z* calculated for $C_{20}H_{41}O_3S^+$ $[M + H]^+$: 361.27709, found 361.27765.

*Synthesis of Thioether-Lipid Prodrugs of Tenofovir*

<u>General Procedure for DCC Coupling</u>. Anhydrous tenofovir (200 mg, 0.696 mmol, 1.00 eq), thioether-containing alcohol (1.00 eq), DCC (287 mg, 1.39 mmol, 2.00 eq), and Ar-degassed anhydrous DMF or NMP (0.3 M) were added to an oven-dried Biotage microwave reaction vial

(2-5 mL) equipped with a magnetic stir bar under an atmosphere of Ar. While stirring the heterogeneous mixture vigorously at room temperature, triethylamine (194 μL, 1.39 mmol, 2.00 eq) was added. The reaction mixture was then stirred at room temperature for 10 min or until complete dissolution of starting material was observed. DMAP (8.50 mg, 0.0696 mmol, 10 mol %) was subsequently added, and the reaction mixture was stirred at 100-105 $^{o}$C overnight. After confirming product formation by LC-MS, the reaction mixture was cooled to room temperature, quenched with $H_2O$ (300 μL), and immediately purified by column chromatography to yield tenofovir prodrugs.

***Ammonium 2-(heptadecylthio)ethyl (R)-(((1-(6-amino-9H-purin-9-yl)propan-2-yl)oxy)methyl) phosphonate*** **(2b).** Synthesis was carried out according to *General Procedure for DCC Coupling* using compound **7b** (220 mg, 0.696 mmol, 1.00 eq). After quenching with $H_2O$, the reaction mixture was purified via normal-phase column chromatography eluting along a gradient of 0-80% 80:20:3 DCM:MeOH:$NH_4OH$ in DCM. The product fractions were then collected and concentrated under reduced pressure. The obtained residue was subsequently taken up in a solution of DCM (5 mL) and methanolic ammonia (7 N, 5 mL), and the mixture was stirred at room temperature overnight. After the mixture was concentrated under reduced pressure, the resulting material was further purified by reversed-phase column chromatography eluting along a gradient of 0-90% MeOH in $H_2O$. The product fractions were once more collected and concentrated under reduced pressure. Finally, the acquired solid was washed with acetone and dried under high vacuum to yield a white powder (231 mg, 0.384 mmol, 55% yield). MP = Decomp. 145-150 $^{o}$C. **TLC** (80:20:3 DCM:MeOH:$NH_4OH$) $R_f$ = 0.33. **$^1$H NMR** (600 MHz, $CD_3OD$) δ 8.32 (s, 1H), 8.20 (s, 1H), 4.38 (dd, *J* = 14.5, 3.2 Hz, 1H), 4.23 (dd, *J* = 14.4, 6.7 Hz, 1H), 3.95 – 3.84 (m, 3H), 3.72 (dd, *J* = 12.8, 9.4 Hz, 1H), 3.49 (dd, *J* = 12.8, 10.1 Hz, 1H), 2.68 – 2.57 (m, 2H), 2.48 (app t,

*J* = 7.3 Hz, 2H), 1.54 – 1.46 (m, 2H), 1.37 – 1.22 (m, 28H), 1.16 (d, *J* = 6.2 Hz, 3H), 0.90 (app t, *J* = 7.0 Hz, 3H). **$^{13}$C NMR** (151 MHz, $CD_3OD$) δ 157.2, 153.5, 151.0, 144.2, 119.6, 77.0 (d, $J_{CP}$ = 12.9 Hz), 65.6 (d, $J_{CP}$ = 160.3 Hz), 65.5 (d, $J_{CP}$ = 5.9 Hz), 33.5 (d, $J_{CP}$ = 6.3 Hz), 33.2, 33.1, 31.0, 30.79, 30.77, 30.75, 30.72, 30.69, 30.5, 30.4, 29.9, 23.7, 16.8, 14.4 (*23 out of 28 carbon signals observed due to chemical shift equivalence*). **$^{31}$P NMR** (243 MHz, $CD_3OD$) δ 15.3. **HRMS** (NSI) *m/z* calculated for $C_{28}H_{53}N_5O_4PS^+$ $[M + H]^+$: 586.35504, found 586.35406. **LC-MS** (ESI, C8, 0.5 mL/min) 65-95% MeCN in $H_2O$, 6 min, rt = 1.779 min, *m/z* = 586.3 $[M + H]^+$; 50-95% MeCN in $H_2O$, 6 min, rt = 4.379 min, *m/z* = 586.3 $[M + H]^+$.

***Ammonium 6-(tridecylthio)hexyl (R)-(((1-(6-amino-9H-purin-9-yl)propan-2-yl)oxy)methyl) phosphonate*** **(2c).** Synthesis was carried out according to *General Procedure for DCC Coupling* using compound **7c** (220 mg, 0.696 mmol, 1.00 eq). After quenching with $H_2O$, the reaction mixture was purified via normal-phase column chromatography eluting along a gradient of 0-80% 80:20:3 DCM:MeOH:$NH_4OH$ in DCM. The product fractions were then collected and concentrated under reduced pressure. The obtained residue was subsequently taken up in a solution of MeOH (5 mL) and aqueous ammonia (28-30% $NH_3$ in $H_2O$, 5 mL). The mixture was stirred at room temperature overnight and then immediately purified by reversed-phase column chromatography eluting along a gradient of 0-85% MeOH in $H_2O$. The product fractions were once more collected and concentrated under reduced pressure. Finally, the acquired solid was washed with acetone and dried under high vacuum to yield a white powder (258 mg, 0.428 mmol, 62% yield). MP = Decomp. 152-156 $^oC$. **TLC** (80:20:3 DCM:MeOH:$NH_4OH$) $R_f$ = 0.33. **$^1$H NMR** (600 MHz, $CD_3OD$) δ 8.31 (s, 1H), 8.20 (s, 1H), 4.38 (dd, *J* = 14.4, 3.1 Hz, 1H), 4.23 (dd, *J* = 14.4, 6.8 Hz, 1H), 3.90 (pd, *J* = 6.3, 3.1 Hz, 1H), 3.79 – 3.67 (m, 3H), 3.46 (dd, *J* = 12.7, 10.1 Hz, 1H), 2.50 – 2.43 (m, 4H), 1.58 – 1.45 (m, 6H), 1.41 – 1.23 (m, 24H), 1.17 (d, *J* = 6.2 Hz, 3H),

0.90 (app t, $J$ = 7.0 Hz, 3H). **$^{13}$C NMR** (151 MHz, $CD_3OD$) δ 157.1, 153.3, 150.9, 144.3, 119.6, 76.9 (d, $J_{CP}$ = 12.8 Hz), 65.8 (d, $J_{CP}$ = 6.0 Hz), 65.5 (d, $J_{CP}$ = 159.8 Hz), 33.1, 32.9, 32.8, 32.0 (d, $J_{CP}$ = 6.2 Hz), 30.80, 30.78, 30.75, 30.70, 30.67, 30.5, 30.3, 29.9, 29.6, 26.5, 23.7, 16.8, 14.4 (*25 out of 28 carbon signals observed due to chemical shift equivalence*). **$^{31}$P NMR** (243 MHz, $CD_3OD$) δ 15.4. **HRMS** (APCI) *m/z* calculated for $C_{28}H_{51}N_5O_4PS^-$ $[M - H]^-$: 584.34049, found 584.34084. **LC-MS** (ESI, C8, 0.5 mL/min) 65-95% MeCN in $H_2O$, 6 min, rt = 1.445 min, *m/z* = 586.3 $[M + H]^+$; 50-95% MeCN in $H_2O$, 6 min, rt = 3.944 min, *m/z* = 586.3 $[M + H]^+$.

***Ammonium 9-(decylthio)nonyl (R)-(((1-(6-amino-9H-purin-9-yl)propan-2-yl)oxy)methyl) phosphonate* (2d).** Synthesis was carried out according to *General Procedure for DCC Coupling* using compound **7d** (220 mg, 0.696 mmol, 1.00 eq). After quenching with $H_2O$, the reaction mixture was purified via normal-phase column chromatography eluting along a gradient of 0-85% 80:20:3 DCM:MeOH:$NH_4OH$ in DCM. The product fractions were then collected and concentrated under reduced pressure. The obtained residue was subsequently taken up in a solution of DCM (5 mL) and methanolic ammonia (7 N, 5 mL), and the mixture was stirred at room temperature for 30 min. After the mixture was concentrated under reduced pressure, the resulting material was further purified by reversed-phase column chromatography eluting along a gradient of 0-85% MeOH in $H_2O$. The product fractions were once more collected and concentrated under reduced pressure. Finally, the acquired solid was washed with acetone and dried under high vacuum to yield a white powder (122 mg, 0.208 mmol, 30% yield). MP = Decomp. 177-183 $^{o}$C. **TLC** (80:20:3 DCM:MeOH:$NH_4OH$) $R_f$ = 0.32. **$^1$H NMR** (600 MHz, $CD_3OD$) δ 8.33 (s, 1H), 8.21 (s, 1H), 4.39 (dd, $J$ = 14.5, 3.1 Hz, 1H), 4.24 (dd, $J$ = 14.5, 6.7 Hz, 1H), 3.91 (pd, $J$ = 6.2, 3.0 Hz, 1H), 3.80 – 3.68 (m, 3H), 3.47 (dd, $J$ = 12.8, 10.0 Hz, 1H), 2.51 – 2.45 (m, 4H), 1.60 – 1.44 (m, 6H), 1.42 – 1.22 (m, 24H), 1.16 (d, $J$ = 6.2 Hz, 3H), 0.90 (app t, $J$ = 7.0 Hz, 3H). **$^{13}$C NMR**

(126 MHz, $CD_3OD$) δ 156.7, 152.8, 150.9, 144.5, 119.5, 76.9 (d, $J_{CP}$ = 12.8 Hz), 65.9 (d, $J_{CP}$ = 5.9 Hz), 65.5 (d, $J_{CP}$ = 159.7 Hz), 33.1, 32.9, 32.1 (d, $J_{CP}$ = 6.3 Hz), 30.82, 30.79, 30.7, 30.6, 30.44, 30.40, 30.33, 30.30, 29.90, 29.87, 26.9, 23.7, 16.8, 14.4 (*25 out of 28 carbon signals observed due to chemical shift equivalence*). **$^{31}$P NMR** (243 MHz, $CD_3OD$) δ 15.3. **HRMS** (APCI) *m/z* calculated for $C_{28}H_{51}N_5O_4PS^-$ [M - H]$^-$: 584.34049, found 584.34077. **LC-MS** (ESI, C8, 0.5 mL/min) 65-95% MeCN in $H_2O$, 6 min, rt = 1.266 min, *m/z* = 586.3 [M + H]$^+$; 45-95% MeCN in $H_2O$, 6 min, rt = 4.384 min, *m/z* = 586.3 [M + H]$^+$.

***Ammonium 12-(heptylthio)dodecyl (R)-(((1-(6-amino-9H-purin-9-yl)propan-2-yl)oxy)methyl) phosphonate* (2e).** Synthesis was carried out according to *General Procedure for DCC Coupling* using compound **7e** (220 mg, 0.696 mmol, 1.00 eq). After quenching with $H_2O$, the reaction mixture was purified via normal-phase column chromatography eluting along a gradient of 0-80% 80:20:3 DCM:MeOH:$NH_4OH$ in DCM. The product fractions were then collected and concentrated under reduced pressure. The obtained residue was subsequently taken up in a solution of DCM (5 mL) and methanolic ammonia (7 N, 5 mL), and the mixture was stirred at room temperature overnight. After the mixture was concentrated under reduced pressure, the resulting material was further purified by reversed-phase column chromatography eluting along a gradient of 0-90% MeOH in $H_2O$. The product fractions were once more collected and concentrated under reduced pressure. Finally, the acquired solid was washed with acetone and dried under high vacuum to yield a white powder (291 mg, 0.482 mmol, 69% yield). MP = Decomp. 156-162 $^{o}$C. **TLC** (80:20:3 DCM:MeOH:$NH_4OH$) $R_f$ = 0.32. **$^1$H NMR** (600 MHz, $CD_3OD$) δ 8.31 (s, 1H), 8.20 (s, 1H), 4.37 (dd, *J* = 14.4, 3.1 Hz, 1H), 4.23 (dd, *J* = 14.4, 6.7 Hz, 1H), 3.89 (pd, *J* = 6.3, 3.1 Hz, 1H), 3.79 – 3.67 (m, 3H), 3.46 (dd, *J* = 12.7, 10.0 Hz, 1H), 2.51 – 2.46 (m, 4H), 1.60 – 1.43 (m, 6H), 1.42 – 1.35 (m, 4H), 1.35 – 1.21 (m, 20H), 1.16 (d, *J* = 6.2 Hz, 3H), 0.90 (app t, *J* = 7.0

Hz, 3H). **$^{13}$C NMR** (151 MHz, $CD_3OD$) δ 157.2, 153.5, 150.9, 144.2, 119.6, 76.9 (d, $J_{CP}$ = 12.7 Hz), 65.9 (d, $J_{CP}$ = 5.8 Hz), 65.6 (d, $J_{CP}$ = 159.8 Hz), 33.0, 32.9, 32.1 (d, $J_{CP}$ = 6.4 Hz), 30.81, 30.80, 30.73, 30.70, 30.67, 30.5, 30.3, 30.0, 29.88, 29.86, 26.9, 23.7, 16.8, 14.4 (*25 out of 28 carbon signals observed due to chemical shift equivalence*). **$^{31}$P NMR** (243 MHz, $CD_3OD$) δ 15.2. **HRMS** (NSI) *m/z* calculated for $C_{28}H_{53}N_5O_4PS^+$ [M + H]$^+$: 586.35504, found 586.35420. **LC-MS** (ESI, C8, 0.5 mL/min) 60-95% MeCN in $H_2O$, 6 min, rt = 1.610 min, *m/z* = 586.3 [M + H]$^+$; 45-95% MeCN in $H_2O$, 6 min, rt = 4.298 min, *m/z* = 586.3 [M + H]$^+$.

***Ammonium 15-(butylthio)pentadecyl (R)-(((1-(6-amino-9H-purin-9-yl)propan-2-yl)oxy) methyl)phosphonate* (2f).** Synthesis was carried out according to *General Procedure for DCC Coupling* using compound **7f** (220 mg, 0.696 mmol, 1.00 eq). After quenching with $H_2O$, the reaction mixture was purified via normal-phase column chromatography eluting along a gradient of 0-80% 80:20:3 DCM:MeOH:$NH_4OH$ in DCM. The product fractions were then collected and concentrated under reduced pressure. The obtained residue was subsequently taken up in a solution of DCM (5 mL) and methanolic ammonia (7 N, 5 mL), and the mixture was stirred at room temperature overnight. After the mixture was concentrated under reduced pressure, the resulting material was further purified by reversed-phase column chromatography eluting along a gradient of 0-80% MeOH in $H_2O$. The product fractions were once more collected and concentrated under reduced pressure. Finally, the acquired solid was washed with acetone and dried under high vacuum to yield a white powder (211 mg, 0.350 mmol, 50% yield). MP = Decomp. 142-147 $^o$C. **TLC** (80:20:3 DCM:MeOH:$NH_4OH$) $R_f$ = 0.32. **$^1$H NMR** (600 MHz, $CD_3OD$) δ 8.31 (s, 1H), 8.20 (s, 1H), 4.37 (dd, *J* = 14.4, 3.1 Hz, 1H), 4.23 (dd, *J* = 14.4, 6.7 Hz, 1H), 3.89 (pd, *J* = 6.3, 3.0 Hz, 1H), 3.79 – 3.66 (m, 3H), 3.46 (dd, *J* = 12.7, 10.0 Hz, 1H), 2.53 – 2.46 (m, 4H), 1.60 – 1.45 (m, 6H), 1.45 – 1.35 (m, 4H), 1.34 – 1.20 (m, 20H), 1.16 (d, *J* = 6.2 Hz, 3H), 0.92 (app t, *J* = 7.4

Hz, 3H). **$^{13}$C NMR** (151 MHz, $CD_3OD$) δ 157.2, 153.5, 150.9, 144.2, 119.6, 76.9 (d, $J_{CP}$ = 12.8 Hz), 65.9 (d, $J_{CP}$ = 5.8 Hz), 65.6 (d, $J_{CP}$ = 160.1 Hz), 33.0, 32.9, 32.6, 32.1 (d, $J_{CP}$ = 6.3 Hz), 30.79, 30.77, 30.75, 30.74, 30.73, 30.70, 30.65, 30.5, 30.3, 29.9, 26.9, 23.0, 16.8, 14.0 (*26 out of 28 carbon signals observed due to chemical shift equivalence*). **$^{31}$P NMR** (243 MHz, $CD_3OD$) δ 15.2. **HRMS** (NSI) *m/z* calculated for $C_{28}H_{53}N_5O_4PS^+$ [M + H]$^+$: 586.35504, found 586.35455. **LC-MS** (ESI, C8, 0.5 mL/min) 60-95% MeCN in $H_2O$, 6 min, rt = 1.663 min, *m/z* = 586.3 [M + H]$^+$; 45-95% MeCN in $H_2O$, 6 min, rt = 4.369 min, *m/z* = 586.3 [M + H]$^+$.

***Ammonium 3-(dodecylthio)propyl (R)-(((1-(6-amino-9H-purin-9-yl)propan-2-yl)oxy)methyl) phosphonate* (2g).** Synthesis was carried out according to *General Procedure for DCC Coupling* using compound **7g** (181 mg, 0.696 mmol, 1.00 eq). After quenching with $H_2O$, the reaction mixture was purified via normal-phase column chromatography eluting along a gradient of 0-80% 80:20:3 DCM:MeOH:$NH_4OH$ in DCM. The product fractions were then collected and concentrated under reduced pressure. The obtained residue was subsequently taken up in a solution of MeOH (5 mL) and aqueous ammonia (28-30% $NH_3$ in $H_2O$, 5 mL). The mixture was stirred at room temperature for 20 min and then immediately purified by reversed-phase column chromatography eluting along a gradient of 0-85% MeOH in $H_2O$. The product fractions were once more collected and concentrated under reduced pressure. Finally, the acquired solid was washed with acetone and dried under high vacuum to yield a white powder (232 mg, 0.425 mmol, 61% yield). MP = Decomp. 177-183 $^oC$. **TLC** (80:20:3 DCM:MeOH:$NH_4OH$) $R_f$ = 0.31. **$^1$H NMR** (600 MHz, $CD_3OD$) δ 8.33 (s, 1H), 8.21 (s, 1H), 4.39 (dd, *J* = 14.4, 3.2 Hz, 1H), 4.24 (dd, *J* = 14.4, 6.8 Hz, 1H), 3.95 – 3.80 (m, 3H), 3.72 (dd, *J* = 12.8, 9.4 Hz, 1H), 3.48 (dd, *J* = 12.8, 10.0 Hz, 1H), 2.51 (t, *J* = 7.3 Hz, 2H), 2.44 (app t, *J* = 7.3 Hz, 2H), 1.82 – 1.71 (m, 2H), 1.55 – 1.48 (m, 2H), 1.38 – 1.23 (m, 18H), 1.17 (d, *J* = 6.2 Hz, 3H), 0.90 (app t, *J* = 7.0 Hz, 3H). **$^{13}$C NMR**

(151 MHz, $CD_3OD$) δ 156.6, 152.5, 150.8, 144.5, 119.6, 76.9 (d, $J_{CP}$ = 12.9 Hz), 65.4 (d, $J_{CP}$ = 160.0 Hz), 64.6 (d, $J_{CP}$ = 5.6 Hz), 33.1, 32.8, 32.2 (d, $J_{CP}$ = 6.2 Hz), 30.77, 30.75, 30.73, 30.69, 30.5, 30.4, 29.9, 29.2, 23.7, 16.8, 14.4 (*22 out of 24 carbon signals observed due to chemical shift equivalence*). **$^{31}$P NMR** (243 MHz, $CD_3OD$) δ 15.4. **HRMS** (NSI) *m/z* calculated for $C_{24}H_{45}N_5O_4PS^+$ $[M + H]^+$: 530.29244, found 530.29162. **LC-MS** (ESI, C8, 0.5 mL/min) 50-95% MeCN in $H_2O$, 6 min, rt = 1.531 min, *m/z* = 530.3 $[M + H]^+$; 30-95% MeCN in $H_2O$, 6 min, rt = 4.627 min, *m/z* = 530.3 $[M + H]^+$.

***Ammonium 3-(tetradecylthio)propyl (R)-(((1-(6-amino-9H-purin-9-yl)propan-2-yl)oxy)methyl) phosphonate* (2h).** Synthesis was carried out according to *General Procedure for DCC Coupling* using compound **7h** (201 mg, 0.696 mmol, 1.00 eq). After quenching with $H_2O$, the reaction mixture was purified via normal-phase column chromatography eluting along a gradient of 0-80% 80:20:3 DCM:MeOH:$NH_4OH$ in DCM. The product fractions were then collected and concentrated under reduced pressure. The obtained residue was subsequently taken up in a solution of MeOH (5 mL) and aqueous ammonia (28-30% $NH_3$ in $H_2O$, 5 mL). The mixture was stirred at room temperature for 20 min and then immediately purified by reversed-phase column chromatography eluting along a gradient of 0-85% MeOH in $H_2O$. The product fractions were once more collected and concentrated under reduced pressure. Finally, the acquired solid was washed with acetone and dried under high vacuum to yield a white powder (216 mg, 0.377 mmol, 54% yield). MP = Decomp. 168-174 $^oC$. **TLC** (80:20:3 DCM:MeOH:$NH_4OH$) $R_f$ = 0.32. **$^1$H NMR** (600 MHz, $CD_3OD$) δ 8.33 (s, 1H), 8.21 (s, 1H), 4.39 (dd, *J* = 14.4, 3.1 Hz, 1H), 4.24 (dd, *J* = 14.4, 6.8 Hz, 1H), 3.95 – 3.80 (m, 3H), 3.73 (dd, *J* = 12.8, 9.4 Hz, 1H), 3.49 (dd, *J* = 12.8, 10.0 Hz, 1H), 2.51 (t, *J* = 7.2 Hz, 2H), 2.44 (app t, *J* = 7.3 Hz, 2H), 1.82 – 1.70 (m, 2H), 1.55 – 1.48 (m, 2H), 1.39 – 1.22 (m, 22H), 1.17 (d, *J* = 6.2 Hz, 3H), 0.90 (app t, *J* = 7.0 Hz, 3H). **$^{13}$C NMR**

(151 MHz, $CD_3OD$) δ 156.5, 152.4, 150.8, 144.5, 119.6, 76.9 (d, $J_{CP}$ = 12.9 Hz), 65.4 (d, $J_{CP}$ = 160.1 Hz), 64.6 (d, $J_{CP}$ = 5.6 Hz), 33.1, 32.8, 32.2 (d, $J_{CP}$ = 6.2 Hz), 30.79, 30.78, 30.76, 30.72, 30.69, 30.5, 30.4, 29.9, 29.2, 23.7, 16.8, 14.4 (*23 out of 26 carbon signals observed due to chemical shift equivalence*). **$^{31}$P NMR** (243 MHz, $CD_3OD$) δ 15.5. **HRMS** (NSI) *m/z* calculated for $C_{26}H_{49}N_5O_4PS^+$ $[M + H]^+$: 558.32374, found 558.32311. **LC-MS** (ESI, C8, 0.5 mL/min) 60-95% MeCN in $H_2O$, 6 min, rt = 1.360 min, *m/z* = 558.3 $[M + H]^+$; 40-95% MeCN in $H_2O$, 6 min, rt = 4.560 min, *m/z* = 558.3 $[M + H]^+$.

***Ammonium 3-(octadecylthio)propyl (R)-(((1-(6-amino-9H-purin-9-yl)propan-2-yl)oxy)methyl) phosphonate*** **(2i).** Synthesis was carried out according to *General Procedure for DCC Coupling* using compound **7i** (240 mg, 0.696 mmol, 1.00 eq). After quenching with $H_2O$, the reaction mixture was purified via normal-phase column chromatography eluting along a gradient of 0-80% 80:20:3 DCM:MeOH:$NH_4OH$ in DCM. The product fractions were then collected and concentrated under reduced pressure. The obtained residue was subsequently taken up in a solution of MeOH (5 mL) and aqueous ammonia (28-30% $NH_3$ in $H_2O$, 5 mL). The mixture was stirred at room temperature overnight and then immediately purified by reversed-phase column chromatography eluting along a gradient of 0-85% MeOH in $H_2O$. The product fractions were once more collected and concentrated under reduced pressure. Finally, the acquired solid was washed with acetone and dried under high vacuum to yield a white powder (242 mg, 0.384 mmol, 55% yield). MP = Decomp. 172-177 $^{o}$C. **TLC** (80:20:3 DCM:MeOH:$NH_4OH$) $R_f$ = 0.35. **$^1$H NMR** (600 MHz, $CD_3OD$) δ 8.33 (s, 1H), 8.21 (s, 1H), 4.39 (dd, *J* = 14.4, 3.2 Hz, 1H), 4.24 (dd, *J* = 14.4, 6.7 Hz, 1H), 3.94 – 3.80 (m, 3H), 3.72 (dd, *J* = 12.8, 9.5 Hz, 1H), 3.48 (dd, *J* = 12.8, 10.0 Hz, 1H), 2.51 (t, *J* = 7.3 Hz, 2H), 2.47 – 2.42 (m, 2H), 1.82 – 1.70 (m, 2H), 1.56 – 1.48 (m, 2H), 1.41 – 1.22 (m, 30H), 1.16 (d, *J* = 6.3 Hz, 3H), 0.89 (app t, *J* = 7.0 Hz, 3H). **$^{13}$C NMR** (151 MHz,

$CD_3OD$) δ 157.2, 153.5, 150.9, 144.2, 119.6, 76.9 (d, $J_{CP}$ = 13.0 Hz), 65.5 (d, $J_{CP}$ = 160.0 Hz), 64.6 (d, $J_{CP}$ = 5.6 Hz), 33.1, 32.8, 32.3 (d, $J_{CP}$ = 6.3 Hz), 30.79, 30.77, 30.75, 30.72, 30.69, 30.5, 30.4, 29.9, 29.2, 23.7, 16.8, 14.4 (*23 out of 30 carbon signals observed due to chemical shift equivalence*). **$^{31}$P NMR** (243 MHz, $CD_3OD$) δ 15.4. **HRMS** (NSI) *m/z* calculated for $C_{30}H_{57}N_5O_4PS^+$ [M + H]$^+$: 614.38634, found 614.38621. **LC-MS** (ESI, C8, 0.5 mL/min) 80-95% MeCN in $H_2O$, 6 min, rt = 1.529 min, *m/z* = 614.4 [M + H]$^+$; 60-95% MeCN in $H_2O$, 6 min, rt = 3.988 min, *m/z* = 614.4 [M + H]$^+$.

***Ammonium 3-(icosylthio)propyl (R)-(((1-(6-amino-9H-purin-9-yl)propan-2-yl)oxy)methyl) phosphonate* (2j).** Synthesis was carried out according to *General Procedure for DCC Coupling* using compound **7j** (260 mg, 0.696 mmol, 1.00 eq). After quenching with $H_2O$, the reaction mixture was purified via normal-phase column chromatography eluting along a gradient of 0-80% 80:20:3 DCM:MeOH:$NH_4OH$ in DCM. The product fractions were then collected and concentrated under reduced pressure. The obtained residue was subsequently taken up in a solution of MeOH (5 mL) and aqueous ammonia (28-30% $NH_3$ in $H_2O$, 5 mL). The mixture was stirred at room temperature overnight and then immediately purified by reversed-phase column chromatography eluting along a gradient of 0-90% MeOH in $H_2O$. The product fractions were once more collected and concentrated under reduced pressure. Finally, the acquired solid was washed with acetone and dried under high vacuum to yield a white powder (235 mg, 0.357 mmol, 51% yield). MP = Decomp. 157-162 $^o$C. **TLC** (80:20:3 DCM:MeOH:$NH_4OH$) $R_f$ = 0.36. **$^1$H NMR** (600 MHz, $CD_3OD$) δ 8.32 (s, 1H), 8.20 (s, 1H), 4.38 (dd, *J* = 14.4, 3.2 Hz, 1H), 4.23 (dd, *J* = 14.5, 6.7 Hz, 1H), 3.93 – 3.80 (m, 3H), 3.71 (dd, *J* = 12.7, 9.4 Hz, 1H), 3.47 (dd, *J* = 12.7, 10.0 Hz, 1H), 2.51 (t, *J* = 7.3 Hz, 2H), 2.47 – 2.41 (m, 2H), 1.82 – 1.70 (m, 2H), 1.55 – 1.48 (m, 2H), 1.38 – 1.24 (m, 34H), 1.16 (d, *J* = 6.3 Hz, 3H), 0.90 (app t, *J* = 7.0 Hz, 3H). **$^{13}$C NMR** (151 MHz,

$CD_3OD$) δ 157.2, 153.5, 151.0, 144.2, 119.6, 76.9 (d, $J_{CP}$ = 13.0 Hz), 65.5 (d, $J_{CP}$ = 159.9 Hz), 64.6 (d, $J_{CP}$ = 5.7 Hz), 33.1, 32.8, 32.3 (d, $J_{CP}$ = 6.4 Hz), 30.79, 30.76, 30.72, 30.70, 30.5, 30.4, 29.9, 29.2, 23.7, 16.8, 14.4 (*22 out of 32 carbon signals observed due to chemical shift equivalence*). **$^{31}$P NMR** (243 MHz, $CD_3OD$) δ 15.3. **HRMS** (NSI) *m/z* calculated for $C_{32}H_{61}N_5O_4PS^+$ [M + H]$^+$: 642.41764, found 642.41774. **LC-MS** (ESI, C8, 0.5 mL/min) 85-95% MeCN in $H_2O$, 6 min, rt = 1.629 min, *m/z* = 642.3 [M + H]$^+$; 65-95% MeCN in $H_2O$, 6 min, rt = 4.358 min, *m/z* = 642.4 [M + H]$^+$.

***Ammonium 3-(hexadec-15-yn-1-ylthio)propyl (R)-(((1-(6-amino-9H-purin-9-yl)propan-2-yl) oxy)methyl)phosphonate* (15b).** Synthesis was carried out according to *General Procedure for DCC Coupling* using compound **13b** (218 mg, 0.696 mmol, 1.00 eq). After quenching with $H_2O$, the reaction mixture was purified via normal-phase column chromatography eluting along a gradient of 0-80% 80:20:3 DCM:MeOH:$NH_4OH$ in DCM. The product fractions were then collected and concentrated under reduced pressure. The obtained residue was subsequently taken up in a solution of MeOH (5 mL) and aqueous ammonia (28-30% $NH_3$ in $H_2O$, 5 mL). The mixture was stirred at room temperature for 10 min and then immediately purified by reversed-phase column chromatography eluting along a gradient of 0-85% MeOH in $H_2O$. The product fractions were once more collected and concentrated under reduced pressure. Finally, the acquired solid was washed with acetone and dried under high vacuum to yield a waxy white solid (206 mg, 0.343 mmol, 49% yield). MP = Decomp. 152-158 $^o$C. **TLC** (80:20:3 DCM:MeOH:$NH_4OH$) $R_f$ = 0.33. **$^1$H NMR** (600 MHz, $CD_3OD$) δ 8.33 (s, 1H), 8.22 (s, 1H), 4.39 (dd, *J* = 14.4, 3.2 Hz, 1H), 4.24 (dd, *J* = 14.5, 6.7 Hz, 1H), 3.95 – 3.81 (m, 3H), 3.73 (dd, *J* = 12.8, 9.4 Hz, 1H), 3.49 (dd, *J* = 12.8, 10.0 Hz, 1H), 2.51 (t, *J* = 7.3 Hz, 2H), 2.47 – 2.42 (m, 2H), 2.18 – 2.13 (m, 3H), 1.82 – 1.71 (m, 2H), 1.55 – 1.46 (m, 4H), 1.43 – 1.25 (m, 20H), 1.17 (d, *J* = 6.3 Hz, 3H). **$^{13}$C NMR** (151 MHz,

$CD_3OD$) δ 157.1, 153.3, 150.9, 144.3, 119.6, 85.1, 76.9 (d, $J_{CP}$ = 13.1 Hz), 69.3, 65.5 (d, $J_{CP}$ = 160.0 Hz), 64.6 (d, $J_{CP}$ = 5.7 Hz), 32.8, 32.2 (d, $J_{CP}$ = 6.3 Hz), 30.8, 30.74, 30.70, 30.68, 30.65, 30.4, 30.2, 29.9, 29.8, 29.7, 29.2, 19.0, 16.8 (*25 out of 28 carbon signals observed due to chemical shift equivalence*). **$^{31}$P NMR** (243 MHz, $CD_3OD$) δ 15.4. **HRMS** (NSI) *m/z* calculated for $C_{28}H_{49}N_5O_4PS^+$ $[M + H]^+$: 582.32374, found 582.32318. **LC-MS** (ESI, C8, 0.5 mL/min) 55-95% MeCN in $H_2O$, 6 min, rt = 1.303 min, *m/z* = 582.3 $[M + H]^+$; 35-95% MeCN in $H_2O$, 6 min, rt = 4.564 min, *m/z* = 582.3 $[M + H]^+$.

***Ammonium 3-((14-(trimethylsilyl)tetradec-13-yn-1-yl)thio)propyl (R)-(((1-(6-amino-9H-purin-9-yl)propan-2-yl)oxy)methyl)phosphonate* (15c).** Synthesis was carried out according to *General Procedure for DCC Coupling* using compound **13c** (248 mg, 0.696 mmol, 1.00 eq). After quenching with $H_2O$, the reaction mixture was purified via normal-phase column chromatography eluting along a gradient of 0-80% MeOH in DCM. The product fractions were then collected and concentrated under reduced pressure. The obtained residue was subsequently taken up in a solution of MeOH (8 mL) and $H_2O$ (2 mL), and diammonium phosphate (460 mg, 3.48 mmol, 5.00 eq) was added. The heterogeneous mixture was stirred at room temperature overnight and then immediately purified by reversed-phase column chromatography eluting along a gradient of 0-85% MeOH in $H_2O$. The product fractions were once more collected and concentrated under reduced pressure. Finally, the acquired solid was washed with acetone and dried under high vacuum to yield a white powder (226 mg, 0.361 mmol, 52% yield). MP = Decomp. 157-162 °C. **TLC** (80:20:3 DCM:MeOH:$NH_4OH$) $R_f$ = 0.35. **$^1$H NMR** (600 MHz, $CD_3OD$) δ 8.34 (s, 1H), 8.22 (s, 1H), 4.39 (dd, *J* = 14.4, 3.1 Hz, 1H), 4.24 (dd, *J* = 14.5, 6.8 Hz, 1H), 3.94 – 3.80 (m, 3H), 3.73 (dd, *J* = 12.8, 9.4 Hz, 1H), 3.48 (dd, *J* = 12.7, 10.0 Hz, 1H), 2.50 (t, *J* = 7.3 Hz, 2H), 2.46 – 2.42 (m, 2H), 2.20 (t, *J* = 7.0 Hz, 2H), 1.81 – 1.70 (m, 2H), 1.55 – 1.45 (m, 4H), 1.43 – 1.24 (m, 16H), 1.17 (d,

*J* = 6.2 Hz, 3H), 0.11 (s, 9H). **$^{13}$C NMR** (151 MHz, $CD_3OD$) δ 156.5, 152.4, 150.8, 144.6, 119.5, 108.7, 84.8, 76.9 (d, $J_{CP}$ = 12.8 Hz), 65.4 (d, $J_{CP}$ = 160.3 Hz), 64.6 (d, $J_{CP}$ = 5.8 Hz), 32.8, 32.2 (d, $J_{CP}$ = 6.4 Hz), 30.8, 30.70, 30.70, 30.65, 30.6, 30.4, 30.1, 29.9, 29.8, 29.7, 29.2, 20.4, 16.8, 0.3 (*26 out of 29 carbon signals observed due to chemical shift equivalence*). **$^{31}$P NMR** (243 MHz, $CD_3OD$) δ 15.5. **HRMS** (APCI) *m/z* calculated for $C_{29}H_{51}N_5O_4PSSi^-$ [M - H]$^-$: 624.31741, found 624.31779. **LC-MS** (ESI, C8, 0.5 mL/min) 60-95% MeCN in $H_2O$, 6 min, rt = 1.698 min, *m/z* = 626.3 [M + H]$^+$; 45-95% MeCN in $H_2O$, 6 min, rt = 4.410 min, *m/z* = 626.3 [M + H]$^+$.

***Ammonium 3-((13-(triisopropylsilyl)tridec-12-yn-1-yl)thio)propyl (R)-(((1-(6-amino-9H-purin-9-yl) propan-2-yl)oxy)methyl)phosphonate* (15d).** Synthesis was carried out according to *General Procedure for DCC Coupling* using compound **13d** (297 mg, 0.696 mmol, 1.00 eq). After quenching with $H_2O$, the reaction mixture was purified via normal-phase column chromatography eluting along a gradient of 0-80% 80:20:3 DCM:MeOH:$NH_4OH$ in DCM. The product fractions were then collected and concentrated under reduced pressure. The obtained residue was subsequently taken up in a solution of MeOH (5 mL) and aqueous ammonia (28-30% $NH_3$ in $H_2O$, 5 mL). The mixture was stirred at room temperature for 20 min and then immediately purified by reversed-phase column chromatography eluting along a gradient of 0-85% MeOH in $H_2O$. The product fractions were once more collected and concentrated under reduced pressure. Finally, the acquired solid was washed with acetone and dried under high vacuum to yield a white powder (175 mg, 0.246 mmol, 35% yield). MP = Decomp. 163-169 $^oC$. **TLC** (80:20:3 DCM:MeOH:$NH_4OH$) $R_f$ = 0.37. **$^1$H NMR** (600 MHz, $CD_3OD$) δ 8.33 (s, 1H), 8.22 (s, 1H), 4.39 (dd, *J* = 14.4, 3.2 Hz, 1H), 4.24 (dd, *J* = 14.4, 6.7 Hz, 1H), 3.95 – 3.81 (m, 3H), 3.73 (dd, *J* = 12.8, 9.4 Hz, 1H), 3.49 (dd, *J* = 12.8, 10.0 Hz, 1H), 2.51 (t, *J* = 7.3 Hz, 2H), 2.45 (app t, *J* = 7.3 Hz, 2H), 2.26 (t, *J* = 6.6 Hz, 2H), 1.82 – 1.71 (m, 2H), 1.56 – 1.42 (m, 6H), 1.39 – 1.23 (m, 12H), 1.17 (d,

$J$ = 6.3 Hz, 3H), 1.10 – 0.95 (m, 21H). **$^{13}$C NMR** (151 MHz, $CD_3OD$) δ 156.5, 152.3, 150.8, 144.6, 119.5, 110.7, 80.8, 76.9 (d, $J_{CP}$ = 12.8 Hz), 65.4 (d, $J_{CP}$ = 160.1 Hz), 64.6 (d, $J_{CP}$ = 5.8 Hz), 32.9, 32.2 (d, $J_{CP}$ = 6.3 Hz), 30.8, 30.68, 30.67, 30.6, 30.4, 30.1, 29.9, 29.8, 29.6, 29.2, 20.3, 19.1, 16.8, 12.5 (*26 out of 34 carbon signals observed due to chemical shift equivalence*). **$^{31}$P NMR** (243 MHz, $CD_3OD$) δ 15.4. **HRMS** (ESI) *m/z* calculated for $C_{34}H_{63}N_5O_4PSSi^+$ $[M + H]^+$: 696.41022, found 696.41097. **LC-MS** (ESI, C8, 0.5 mL/min) 80-95% MeCN in $H_2O$, 6 min, rt = 1.824 min, *m/z* = 696.3 $[M + H]^+$; 60-95% MeCN in $H_2O$, 6 min, rt = 4.542 min, *m/z* = 696.3 $[M + H]^+$.

***Ammonium 3-((15,15-dimethylhexadec-13-yn-1-yl)thio)propyl (R)-(((1-(6-amino-9H-purin-9-yl)propan-2-yl)oxy)methyl)phosphonate* (15e).** Synthesis was carried out according to *General Procedure for DCC Coupling* using compound **13e** (237 mg, 0.696 mmol, 1.00 eq). After quenching with $H_2O$, the reaction mixture was purified via normal-phase column chromatography eluting along a gradient of 0-80% 80:20:3 DCM:MeOH:$NH_4OH$ in DCM. The product fractions were then collected and concentrated under reduced pressure. The obtained residue was subsequently taken up in a solution of methanolic ammonia (7 N, 5 mL) and $H_2O$ (5 mL). The mixture was stirred at room temperature for 20 min and then immediately purified by reversed-phase column chromatography eluting along a gradient of 0-75% MeOH in $H_2O$. The product fractions were once more collected and concentrated under reduced pressure. Finally, the acquired solid was washed with acetone and dried under high vacuum to yield a white powder (195 mg, 0.311 mmol, 45% yield). MP = Decomp. 152-158 $^{o}$C. **TLC** (80:20:3 DCM:MeOH:$NH_4OH$) $R_f$ = 0.34. **$^1$H NMR** (600 MHz, $CD_3OD$) δ 8.34 (s, 1H), 8.22 (s, 1H), 4.40 (dd, $J$ = 14.4, 3.2 Hz, 1H), 4.24 (dd, $J$ = 14.4, 6.8 Hz, 1H), 3.96 – 3.81 (m, 3H), 3.73 (dd, $J$ = 12.8, 9.4 Hz, 1H), 3.49 (dd, $J$ = 12.7, 9.9 Hz, 1H), 2.51 (t, $J$ = 7.3 Hz, 2H), 2.45 (app t, $J$ = 7.3 Hz, 2H), 2.10 (t, $J$ = 6.8 Hz, 2H), 1.83 – 1.71 (m, 2H), 1.56 – 1.48 (m, 2H), 1.47 – 1.24 (m, 18H), 1.17 (d,

$J$ = 6.2 Hz, 3H), 1.17 (s, 9H). **$^{13}$C NMR** (151 MHz, $CD_3OD$) δ 156.2, 151.9, 150.8, 144.7, 119.5, 89.7, 79.5, 76.8 (d, $J_{CP}$ = 12.7 Hz), 65.4 (d, $J_{CP}$ = 160.3 Hz), 64.6 (d, $J_{CP}$ = 5.5 Hz), 32.8, 32.2 (d, $J_{CP}$ = 6.6 Hz), 31.9, 30.8, 30.70, 30.66, 30.4, 30.23, 30.18, 29.9, 29.8, 29.2, 28.3, 19.3, 16.8 (*25 out of 30 carbon signals observed due to chemical shift equivalence*). **$^{31}$P NMR** (243 MHz, $CD_3OD$) δ 15.5. **HRMS** (ESI) *m/z* calculated for $C_{30}H_{53}N_5O_4PS^+$ [M + H]$^+$: 610.35504, found 610.35558. **LC-MS** (ESI, C8, 0.5 mL/min) 60-95% MeCN in $H_2O$, 6 min, rt = 1.573 min, *m/z* = 610.3 [M + H]$^+$; 40-95% MeCN in $H_2O$, 6 min, rt = 4.834 min, *m/z* = 610.3 [M + H]$^+$.

***Ammonium 3-((15,15-dimethylhexadecyl)thio)propyl (R)-(((1-(6-amino-9H-purin-9-yl)propan-2-yl)oxy)methyl)phosphonate* (16e).** Synthesis was carried out according to *General Procedure for DCC Coupling* using compound **14e** (240 mg, 0.696 mmol, 1.00 eq). After quenching with $H_2O$, the reaction mixture was purified via normal-phase column chromatography eluting along a gradient of 0-80% 80:20:3 DCM:MeOH:$NH_4OH$ in DCM. The product fractions were then collected and concentrated under reduced pressure. The obtained residue was subsequently taken up in a solution of methanolic ammonia (7 N, 5 mL) and $H_2O$ (5 mL). The mixture was stirred at room temperature for 20 min and then immediately purified by reversed-phase column chromatography eluting along a gradient of 0-80% MeOH in $H_2O$. The product fractions were once more collected and concentrated under reduced pressure. Finally, the acquired solid was washed with acetone and dried under high vacuum to yield a white powder (202 mg, 0.319 mmol, 46% yield). MP = Decomp. 166-172 °C. **TLC** (80:20:3 DCM:MeOH:$NH_4OH$) $R_f$ = 0.34. **$^1$H NMR** (600 MHz, $CD_3OD$) δ 8.33 (s, 1H), 8.21 (s, 1H), 4.39 (dd, $J$ = 14.4, 3.0 Hz, 1H), 4.24 (dd, $J$ = 14.4, 6.7 Hz, 1H), 3.95 – 3.80 (m, 3H), 3.72 (dd, $J$ = 12.7, 9.5 Hz, 1H), 3.49 (dd, $J$ = 12.7, 10.1 Hz, 1H), 2.51 (t, $J$ = 7.3 Hz, 2H), 2.44 (app t, $J$ = 7.3 Hz, 2H), 1.82 – 1.71 (m, 2H), 1.56 – 1.48 (m, 2H), 1.38 – 1.23 (m, 22H), 1.20 – 1.14 (m, 5H), 0.87 (s, 9H). **$^{13}$C NMR** (151 MHz, $CD_3OD$)

δ 156.5, 152.4, 150.8, 144.6, 119.6, 76.9 (d, $J_{CP}$ = 13.3 Hz), 65.4 (d, $J_{CP}$ = 160.3 Hz), 64.6 (d, $J_{CP}$ = 5.5 Hz), 45.4, 32.8, 32.2 (d, $J_{CP}$ = 6.3 Hz), 31.8, 31.0, 30.80, 30.77, 30.75, 30.72, 30.70, 30.4, 29.93, 29.85, 29.2, 25.7, 16.8 (*24 out of 30 carbon signals observed due to chemical shift equivalence*). **$^{31}$P NMR** (243 MHz, $CD_3OD$) δ 15.4. **HRMS** (ESI) *m/z* calculated for $C_{30}H_{57}N_5O_4PS^+$ $[M + H]^+$: 614.38634, found 614.38703. **LC-MS** (ESI, C8, 0.5 mL/min) 75-95% MeCN in $H_2O$, 6 min, rt = 1.471 min, *m/z* = 614.4 $[M + H]^+$; 55-95% MeCN in $H_2O$, 6 min, rt = 4.353 min, *m/z* = 614.4 $[M + H]^+$.

***Ammonium 3-((12-phenyldodec-11-yn-1-yl)thio)propyl (R)-(((1-(6-amino-9H-purin-9-yl) propan-2-yl)oxy)methyl)phosphonate* (15f).** Synthesis was carried out according to *General Procedure for DCC Coupling* using compound **13f** (232 mg, 0.696 mmol, 1.00 eq). After quenching with $H_2O$, the reaction mixture was purified via normal-phase column chromatography eluting along a gradient of 0-80% 80:20:3 DCM:MeOH:$NH_4OH$ in DCM. The product fractions were then collected and concentrated under reduced pressure. The obtained residue was subsequently taken up in a solution of MeOH (5 mL) and aqueous ammonia (28-30% $NH_3$ in $H_2O$, 5 mL). The mixture was stirred at room temperature for 10 min and then immediately purified by reversed-phase column chromatography eluting along a gradient of 0-85% MeOH in $H_2O$. The product fractions were once more collected and concentrated under reduced pressure. Finally, the acquired solid was washed with acetone and dried under high vacuum to yield a white powder (235 mg, 0.379 mmol, 54% yield). MP = Decomp. 160-165 $^o$C. **TLC** (80:20:3 DCM:MeOH:$NH_4OH$) $R_f$ = 0.33. **$^1$H NMR** (600 MHz, $CD_3OD$) δ 8.32 (s, 1H), 8.21 (s, 1H), 7.35 – 7.31 (m, 2H), 7.29 – 7.23 (m, 3H), 4.38 (dd, *J* = 14.4, 3.2 Hz, 1H), 4.23 (dd, *J* = 14.5, 6.7 Hz, 1H), 3.94 – 3.80 (m, 3H), 3.72 (dd, *J* = 12.8, 9.4 Hz, 1H), 3.48 (dd, *J* = 12.7, 10.0 Hz, 1H), 2.50 (t, *J* = 7.3 Hz, 2H), 2.46 – 2.41 (m, 2H), 2.39 (t, *J* = 7.0 Hz, 2H), 1.82 – 1.70 (m, 2H), 1.62 – 1.55

(m, 2H), 1.55 – 1.43 (m, 4H), 1.39 – 1.25 (m, 10H), 1.16 (d, $J$ = 6.2 Hz, 3H). **$^{13}$C NMR** (151 MHz, $CD_3OD$) δ 156.7, 152.8, 150.9, 144.4, 132.4, 129.3, 128.5, 125.6, 119.6, 90.9, 81.6, 76.9 (d, $J_{CP}$ = 13.0 Hz), 65.4 (d, $J_{CP}$ = 159.9 Hz), 64.6 (d, $J_{CP}$ = 5.7 Hz), 32.8, 32.2 (d, $J_{CP}$ = 6.4 Hz), 30.8, 30.60, 30.57, 30.3, 30.2, 29.93, 29.92, 29.88, 29.2, 20.0, 16.8 (*27 out of 30 carbon signals observed due to chemical shift equivalence*). **$^{31}$P NMR** (243 MHz, $CD_3OD$) δ 15.4. **HRMS** (NSI) *m/z* calculated for $C_{30}H_{45}N_5O_4PS^+$ [M + H]$^+$: 602.29244, found 602.29177. **LC-MS** (ESI, C8, 0.5 mL/min) 50-95% MeCN in $H_2O$, 6 min, rt = 1.645 min, *m/z* = 602.3 [M + H]$^+$; 30-95% MeCN in $H_2O$, 6 min, rt = 4.698 min, *m/z* = 602.3 [M + H]$^+$.

***Ammonium 3-((12-phenyldodecyl)thio)propyl (R)-(((1-(6-amino-9H-purin-9-yl)propan-2-yl) oxy)methyl)phosphonate* (16f).** Synthesis was carried out according to *General Procedure for DCC Coupling* using compound **14f** (234 mg, 0.696 mmol, 1.00 eq). After quenching with $H_2O$, the reaction mixture was purified via normal-phase column chromatography eluting along a gradient of 0-80% 80:20:3 DCM:MeOH:$NH_4OH$ in DCM. The product fractions were then collected and concentrated under reduced pressure. The obtained residue was subsequently taken up in a solution of MeOH (5 mL) and aqueous ammonia (28-30% $NH_3$ in $H_2O$, 5 mL). The mixture was stirred at room temperature for 10 min and then immediately purified by reversed-phase column chromatography eluting along a gradient of 0-85% MeOH in $H_2O$. The product fractions were once more collected and concentrated under reduced pressure. Finally, the acquired solid was washed with acetone and dried under high vacuum to yield a white powder (263 mg, 0.423 mmol, 61% yield). MP = Decomp. 168-173 $^oC$. **TLC** (80:20:3 DCM:MeOH:$NH_4OH$) $R_f$ = 0.33. **$^1$H NMR** (600 MHz, $CD_3OD$) δ 8.32 (s, 1H), 8.21 (s, 1H), 7.25 – 7.20 (m, 2H), 7.17 – 7.10 (m, 3H), 4.38 (dd, $J$ = 14.4, 3.2 Hz, 1H), 4.23 (dd, $J$ = 14.4, 6.7 Hz, 1H), 3.94 – 3.80 (m, 3H), 3.72 (dd, $J$ = 12.8, 9.4 Hz, 1H), 3.48 (dd, $J$ = 12.8, 10.0 Hz, 1H), 2.61 – 2.56 (m, 2H), 2.51 (t, $J$ = 7.3 Hz, 2H), 2.46

– 2.42 (m, 2H), 1.82 – 1.70 (m, 2H), 1.64 – 1.55 (m, 2H), 1.55 – 1.47 (m, 2H), 1.38 – 1.23 (m, 16H), 1.16 (d, *J* = 6.2 Hz, 3H). **$^{13}$C NMR** (151 MHz, $CD_3OD$) δ 156.7, 152.7, 150.9, 144.5, 144.0, 129.4, 129.2, 126.6, 119.6, 76.9 (d, $J_{CP}$ = 12.8 Hz), 65.4 (d, $J_{CP}$ = 159.9 Hz), 64.6 (d, $J_{CP}$ = 5.6 Hz), 36.9, 32.84, 32.76, 32.2 (d, $J_{CP}$ = 6.7 Hz), 30.8, 30.7, 30.6, 30.4, 30.3, 29.9, 29.2, 16.8 (*24 out of 30 carbon signals observed due to chemical shift equivalence*). **$^{31}$P NMR** (243 MHz, $CD_3OD$) δ 15.4. **HRMS** (NSI) *m/z* calculated for $C_{30}H_{49}N_5O_4PS^+$ $[M + H]^+$: 606.32374, found 606.32300. **LC-MS** (ESI, C8, 0.5 mL/min) 55-95% MeCN in $H_2O$, 6 min, rt = 1.692 min, *m/z* = 606.3 $[M + H]^+$; 40-95% MeCN in $H_2O$, 6 min, rt = 4.410 min, *m/z* = 606.3 $[M + H]^+$.

***Ammonium 3-((11-phenoxyundecyl)thio)propyl (R)-(((1-(6-amino-9H-purin-9-yl)propan-2-yl) oxy)methyl)phosphonate*** **(23a).** Synthesis was carried out according to *General Procedure for DCC Coupling* using compound **22a** (236 mg, 0.696 mmol, 1.00 eq). After quenching with $H_2O$, the reaction mixture was purified via normal-phase column chromatography eluting along a gradient of 0-80% 80:20:3 DCM:MeOH:$NH_4OH$ in DCM. The product fractions were then collected and concentrated under reduced pressure. The obtained residue was subsequently taken up in a solution of DCM (5 mL) and methanolic ammonia (7 N, 5 mL), and the mixture was stirred at room temperature overnight. After the mixture was concentrated under reduced pressure, the resulting material was further purified by reversed-phase column chromatography eluting along a gradient of 0-80% MeOH in $H_2O$. The product fractions were once more collected and concentrated under reduced pressure. Finally, the acquired solid was washed with acetone and dried under high vacuum to yield a white powder (309 mg, 0.494 mmol, 71% yield). MP = Decomp. 152-158 $^{o}$C. **TLC** (80:20:3 DCM:MeOH:$NH_4OH$) $R_f$ = 0.34. **$^{1}$H NMR** (600 MHz, $CD_3OD$) δ 8.31 (s, 1H), 8.20 (s, 1H), 7.26 – 7.20 (m, 2H), 6.90 – 6.86 (m, 3H), 4.37 (dd, *J* = 14.4, 3.2 Hz, 1H), 4.23 (dd, *J* = 14.4, 6.7 Hz, 1H), 3.95 (t, *J* = 6.4 Hz, 2H), 3.93 – 3.79 (m, 3H), 3.71

(dd, $J$ = 12.8, 9.4 Hz, 1H), 3.47 (dd, $J$ = 12.7, 10.0 Hz, 1H), 2.50 (t, $J$ = 7.3 Hz, 2H), 2.44 (app t, $J$ = 7.3 Hz, 2H), 1.81 – 1.70 (m, 4H), 1.55 – 1.43 (m, 4H), 1.39 – 1.24 (m, 12H), 1.16 (d, $J$ = 6.2 Hz, 3H). **$^{13}$C NMR** (151 MHz, $CD_3OD$) δ 160.6, 157.2, 153.4, 150.9, 144.2, 130.4, 121.5, 119.6, 115.5, 76.9 (d, $J_{CP}$ = 13.0 Hz), 68.9, 65.5 (d, $J_{CP}$ = 159.9 Hz), 64.6 (d, $J_{CP}$ = 5.7 Hz), 32.8, 32.2 (d, $J_{CP}$ = 6.3 Hz), 30.8, 30.67, 30.65, 30.6, 30.5, 30.43, 30.35, 29.9, 29.2, 27.2, 16.8 (*26 out of 29 carbon signals observed due to chemical shift equivalence*). **$^{31}$P NMR** (243 MHz, $CD_3OD$) δ 15.3. **HRMS** (NSI) *m/z* calculated for $C_{29}H_{47}N_5O_5PS^+$ $[M + H]^+$: 608.30300, found 608.30222. **LC-MS** (ESI, C8, 0.5 mL/min) 50-95% MeCN in $H_2O$, 6 min, rt = 1.411 min, *m/z* = 608.3 $[M + H]^+$; 35-95% MeCN in $H_2O$, 6 min, rt = 4.183 min, *m/z* = 608.3 $[M + H]^+$.

***Ammonium 3-((11-(phenylthio)undecyl)thio)propyl (R)-(((1-(6-amino-9H-purin-9-yl)propan-2-yl)oxy)methyl)phosphonate* (23b).** Synthesis was carried out according to *General Procedure for DCC Coupling* using compound **22b** (247 mg, 0.696 mmol, 1.00 eq). After quenching with $H_2O$, the reaction mixture was purified via normal-phase column chromatography eluting along a gradient of 0-75% 80:20:3 DCM:MeOH:$NH_4OH$ in DCM. The product fractions were then collected and concentrated under reduced pressure. The obtained residue was subsequently taken up in methanolic ammonia (7 N, 5 mL). The mixture was stirred at room temperature for 30 min and then immediately purified by reversed-phase column chromatography eluting along a gradient of 0-75% MeOH in $H_2O$. The product fractions were once more collected and concentrated under reduced pressure. Finally, the acquired solid was washed with acetone and dried under high vacuum to yield a white powder (219 mg, 0.342 mmol, 49% yield). MP = Decomp. 162-168 $^oC$. **TLC** (80:20:3 DCM:MeOH:$NH_4OH$) $R_f$ = 0.34. **$^1$H NMR** (400 MHz, $CD_3OD$) δ 8.33 (s, 1H), 8.21 (s, 1H), 7.34 – 7.22 (m, 4H), 7.19 – 7.10 (m, 1H), 4.39 (dd, $J$ = 14.4, 3.2 Hz, 1H), 4.24 (dd, $J$ = 14.4, 6.7 Hz, 1H), 3.95 – 3.79 (m, 3H), 3.72 (dd, $J$ = 12.7, 9.4 Hz, 1H), 3.48 (dd, $J$ = 12.8, 10.0

Hz, 1H), 2.91 (app t, $J$ = 7.2 Hz, 2H), 2.51 (t, $J$ = 7.3 Hz, 2H), 2.44 (app t, $J$ = 7.3 Hz, 2H), 1.84 – 1.68 (m, 2H), 1.66 – 1.56 (m, 2H), 1.56 – 1.47 (m, 2H), 1.47 – 1.22 (m, 14H), 1.16 (d, $J$ = 6.3 Hz, 3H). **$^{13}$C NMR** (151 MHz, $CD_3OD$) δ 156.0, 151.7, 150.8, 144.8, 138.3, 130.1, 129.9, 126.8, 119.5, 76.8 (d, $J_{CP}$ = 12.7 Hz), 65.4 (d, $J_{CP}$ = 160.9 Hz), 64.6 (d, $J_{CP}$ = 5.5 Hz), 34.3, 32.8, 32.2 (d, $J_{CP}$ = 6.1 Hz), 30.8, 30.62, 30.57, 30.55, 30.32, 30.27, 30.2, 29.9, 29.7, 29.2, 16.8 (*26 out of 29 carbon signals observed due to chemical shift equivalence*). **$^{31}$P NMR** (162 MHz, $CD_3OD$) δ 15.4. **HRMS** (APCI) *m/z* calculated for $C_{29}H_{45}N_5O_4PS_2^-$ [M - H]$^-$: 622.26561, found 622.26573. **LC-MS** (ESI, C8, 0.5 mL/min) 50-95% MeCN in $H_2O$, 6 min, rt = 1.850 min, *m/z* = 624.3 [M + H]$^+$; 35-95% MeCN in $H_2O$, 6 min, rt = 4.443 min, *m/z* = 624.3 [M + H]$^+$.

***Ammonium 3-((12-(4-fluorophenyl)dodec-11-yn-1-yl)thio)propyl (R)-(((1-(6-amino-9H-purin-9-yl)propan-2-yl)oxy)methyl)phosphonate* (15g).** Synthesis was carried out according to *General Procedure for DCC Coupling* using compound **13g** (244 mg, 0.696 mmol, 1.00 eq). After quenching with $H_2O$, the reaction mixture was purified via normal-phase column chromatography eluting along a gradient of 0-80% 80:20:3 DCM:MeOH:$NH_4OH$ in DCM. The product fractions were then collected and concentrated under reduced pressure. The obtained residue was subsequently taken up in a solution of MeOH (8 mL) and $H_2O$ (2 mL), and diammonium phosphate (460 mg, 3.48 mmol, 5.00 eq) was added. The heterogeneous mixture was stirred at room temperature overnight and then immediately purified by reversed-phase column chromatography eluting along a gradient of 0-80% MeOH in $H_2O$. The product fractions were once more collected and concentrated under reduced pressure. Finally, the acquired solid was washed with acetone and dried under high vacuum to yield a white powder (214 mg, 0.345 mmol, 50% yield). MP = Decomp. 130-135 $^oC$. **TLC** (80:20:3 DCM:MeOH:$NH_4OH$) $R_f$ = 0.34. **$^1$H NMR** (600 MHz, $CD_3OD$) δ 8.33 (s, 1H), 8.22 (s, 1H), 7.38 – 7.33 (m, 2H), 7.05 – 6.99 (m, 2H), 4.39 (dd, $J$ = 14.4,

3.2 Hz, 1H), 4.24 (dd, $J$ = 14.4, 6.7 Hz, 1H), 3.95 – 3.81 (m, 3H), 3.73 (dd, $J$ = 12.8, 9.4 Hz, 1H), 3.49 (dd, $J$ = 12.8, 9.9 Hz, 1H), 2.51 (t, $J$ = 7.3 Hz, 2H), 2.44 (app t, $J$ = 7.3 Hz, 2H), 2.38 (t, $J$ = 7.1 Hz, 2H), 1.82 – 1.71 (m, 2H), 1.61 – 1.55 (m, 2H), 1.54 – 1.48 (m, 2H), 1.48 – 1.42 (m, 2H), 1.39 – 1.24 (m, 10H), 1.17 (d, $J$ = 6.2 Hz, 3H). **$^{13}$C NMR** (151 MHz, $CD_3OD$) δ 163.4 (d, $J_{CF}$ = 246.6 Hz), 156.3, 152.0, 150.8, 144.7, 134.4 (d, $J_{CF}$ = 8.3 Hz), 121.8 (d, $J_{CF}$ = 3.3 Hz), 119.5, 116.3 (d, $J_{CF}$ = 22.7 Hz), 90.7 (d, $J_{CF}$ = 1.7 Hz), 80.4, 76.8 (d, $J_{CP}$ = 12.7 Hz), 65.4 (d, $J_{CP}$ = 159.8 Hz), 64.6 (d, $J_{CP}$ = 5.5 Hz), 32.8, 32.2 (d, $J_{CP}$ = 6.1 Hz), 30.8, 30.61, 30.57, 30.3, 30.2, 29.94, 29.91, 29.8, 29.2, 19.9, 16.8 (*27 out of 30 carbon signals observed due to chemical shift equivalence*). **$^{31}$P NMR** (243 MHz, $CD_3OD$) δ 15.5. **HRMS** (ESI) *m/z* calculated for $C_{30}H_{42}N_5O_4FPS^-$ [M - H]$^-$: 618.26846, found 618.26805. **LC-MS** (ESI, C8, 0.5 mL/min) 50-95% MeCN in $H_2O$, 6 min, rt = 1.796 min, *m/z* = 620.3 [M + H]$^+$; 35-95% MeCN in $H_2O$, 6 min, rt = 4.398 min, *m/z* = 620.3 [M + H]$^+$.

***Ammonium 3-((12-(4-fluorophenyl)dodecyl)thio)propyl (R)-(((1-(6-amino-9H-purin-9-yl) propan-2-yl)oxy)methyl)phosphonate* (16g).** Synthesis was carried out according to *General Procedure for DCC Coupling* using compound **14g** (247 mg, 0.696 mmol, 1.00 eq). After quenching with $H_2O$, the reaction mixture was purified via normal-phase column chromatography eluting along a gradient of 0-80% 80:20:3 DCM:MeOH:$NH_4OH$ in DCM. The product fractions were then collected and concentrated under reduced pressure. The obtained residue was subsequently taken up in a solution of MeOH (8 mL) and $H_2O$ (2 mL), and diammonium phosphate (460 mg, 3.48 mmol, 5.00 eq) was added. The heterogeneous mixture was stirred at room temperature overnight and then immediately purified by reversed-phase column chromatography eluting along a gradient of 0-80% MeOH in $H_2O$. The product fractions were once more collected and concentrated under reduced pressure. Finally, the acquired solid was washed with acetone and

dried under high vacuum to yield a white powder (221 mg, 0.354 mmol, 51% yield). MP = Decomp. 161-166 $^{o}$C. **TLC** (80:20:3 DCM:MeOH:$NH_4OH$) $R_f$ = 0.33. **$^{1}$H NMR** (600 MHz, $CD_3OD$) δ 8.33 (s, 1H), 8.22 (s, 1H), 7.18 – 7.12 (m, 2H), 6.99 – 6.92 (m, 2H), 4.39 (dd, *J* = 14.4, 3.2 Hz, 1H), 4.24 (dd, *J* = 14.4, 6.7 Hz, 1H), 3.95 – 3.81 (m, 3H), 3.73 (dd, *J* = 12.8, 9.4 Hz, 1H), 3.49 (dd, *J* = 12.8, 9.9 Hz, 1H), 2.60 – 2.55 (m, 2H), 2.51 (t, *J* = 7.3 Hz, 2H), 2.44 (app t, *J* = 7.3 Hz, 2H), 1.82 – 1.71 (m, 2H), 1.62 – 1.55 (m, 2H), 1.55 – 1.48 (m, 2H), 1.38 – 1.22 (m, 16H), 1.16 (d, *J* = 6.2 Hz, 3H). **$^{13}$C NMR** (151 MHz, $CD_3OD$) δ 162.6 (d, $J_{CF}$ = 241.6 Hz), 156.4, 152.2, 150.8, 144.6, 139.9 (d, $J_{CF}$ = 3.3 Hz), 130.9 (d, $J_{CF}$ = 7.7 Hz), 119.5, 115.7 (d, $J_{CF}$ = 21.0 Hz), 76.8 (d, $J_{CP}$ = 12.7 Hz), 65.4 (d, $J_{CP}$ = 160.3 Hz), 64.6 (d, $J_{CP}$ = 6.1 Hz), 36.0, 32.83, 32.82, 32.2 (d, $J_{CP}$ = 6.3 Hz), 30.8, 30.69, 30.67, 30.5, 30.4, 30.2, 29.9, 29.2, 16.8 (*25 out of 30 carbon signals observed due to chemical shift equivalence*). **$^{31}$P NMR** (243 MHz, $CD_3OD$) δ 15.5. **HRMS** (ESI) *m/z* calculated for $C_{30}H_{46}N_5O_4FPS^-$ [M - H]$^-$: 622.29976, found 622.29968. **LC-MS** (ESI, C8, 0.5 mL/min) 55-95% MeCN in $H_2O$, 6 min, rt = 1.705 min, *m/z* = 624.3 [M + H]$^+$; 40-95% MeCN in $H_2O$, 6 min, rt = 4.423 min, *m/z* = 624.3 [M + H]$^+$.

***Ammonium 3-((11-(4-fluorophenoxy)undecyl)thio)propyl (R)-(((1-(6-amino-9H-purin-9-yl) propan-2-yl)oxy)methyl)phosphonate* (23c).** Synthesis was carried out according to *General Procedure for DCC Coupling* using compound **22c** (248 mg, 0.696 mmol, 1.00 eq). After quenching with $H_2O$, the reaction mixture was purified via normal-phase column chromatography eluting along a gradient of 0-80% 80:20:3 DCM:MeOH:$NH_4OH$ in DCM. The product fractions were then collected and concentrated under reduced pressure. The obtained residue was subsequently taken up in a solution of MeOH (5 mL) and aqueous ammonia (28-30% $NH_3$ in $H_2O$, 5 mL). The mixture was stirred at room temperature for 20 min and then immediately purified by reversed-phase column chromatography eluting along a gradient of 0-75% MeOH in $H_2O$. The

product fractions were once more collected and concentrated under reduced pressure. Finally, the acquired solid was washed with acetone and dried under high vacuum to yield a white powder (242 mg, 0.377 mmol, 54% yield). MP = Decomp. 156-162 °C. **TLC** (80:20:3 DCM:MeOH:$NH_4OH$) $R_f$ = 0.32. **$^1$H NMR** (600 MHz, $CD_3OD$) δ 8.33 (s, 1H), 8.21 (s, 1H), 6.99 – 6.94 (m, 2H), 6.89 – 6.84 (m, 2H), 4.39 (dd, *J* = 14.4, 3.2 Hz, 1H), 4.24 (dd, *J* = 14.4, 6.7 Hz, 1H), 3.95 – 3.81 (m, 5H), 3.73 (dd, *J* = 12.8, 9.4 Hz, 1H), 3.49 (dd, *J* = 12.8, 10.0 Hz, 1H), 2.51 (t, *J* = 7.3 Hz, 2H), 2.44 (app t, *J* = 7.3 Hz, 2H), 1.82 – 1.70 (m, 4H), 1.55 – 1.49 (m, 2H), 1.48 – 1.42 (m, 2H), 1.39 – 1.25 (m, 12H), 1.17 (d, *J* = 6.2 Hz, 3H). **$^{13}$C NMR** (151 MHz, $CD_3OD$) δ 158.5 (d, $J_{CF}$ = 236.6 Hz), 156.9 (d, $J_{CF}$ = 2.2 Hz), 156.4, 152.3, 150.8, 144.6, 119.5, 116.60 (d, $J_{CF}$ = 6.6 Hz), 116.55 (d, $J_{CF}$ = 23.2 Hz), 76.9 (d, $J_{CP}$ = 13.3 Hz), 69.6, 65.4 (d, $J_{CP}$ = 159.8 Hz), 64.6 (d, $J_{CP}$ = 5.5 Hz), 32.8, 32.2 (d, $J_{CP}$ = 6.3 Hz), 30.8, 30.7, 30.64, 30.62, 30.5, 30.4, 30.3, 29.9, 29.2, 27.1, 16.8 (*26 out of 29 carbon signals observed due to chemical shift equivalence*). **$^{31}$P NMR** (243 MHz, $CD_3OD$) δ 15.5. **HRMS** (ESI) *m/z* calculated for $C_{29}H_{46}N_5O_5FPS^+$ $[M + H]^+$: 626.29358, found 626.29399. **LC-MS** (ESI, C8, 0.5 mL/min) 50-95% MeCN in $H_2O$, 6 min, rt = 1.537 min, *m/z* = 626.3 $[M + H]^+$; 35-95% MeCN in $H_2O$, 6 min, rt = 4.275 min, *m/z* = 626.3 $[M + H]^+$.

***Ammonium 3-((12-cyclohexyldodec-11-yn-1-yl)thio)propyl (R)-(((1-(6-amino-9H-purin-9-yl) propan-2-yl)oxy)methyl)phosphonate* (15h).** Synthesis was carried out according to *General Procedure for DCC Coupling* using compound **13h** (236 mg, 0.696 mmol, 1.00 eq). After quenching with $H_2O$, the reaction mixture was purified via normal-phase column chromatography eluting along a gradient of 0-80% 80:20:3 DCM:MeOH:$NH_4OH$ in DCM. The product fractions were then collected and concentrated under reduced pressure. The obtained residue was subsequently taken up in a solution of MeOH (8 mL) and $H_2O$ (2 mL), and diammonium phosphate

(460 mg, 3.48 mmol, 5.00 eq) was added. The heterogeneous mixture was stirred at room temperature overnight and then immediately purified by reversed-phase column chromatography eluting along a gradient of 0-80% MeOH in $H_2O$. The product fractions were once more collected and concentrated under reduced pressure. Finally, the acquired solid was washed with acetone and dried under high vacuum to yield a white powder (205 mg, 0.337 mmol, 48% yield).MP = Decomp. 122-126 $^{o}$C. **TLC** (80:20:3 DCM:MeOH:$NH_4OH$) $R_f$ = 0.34. **$^1$H NMR** (600 MHz, $CD_3OD$) δ 8.34 (s, 1H), 8.23 (s, 1H), 4.40 (dd, *J* = 14.4, 3.2 Hz, 1H), 4.25 (dd, *J* = 14.4, 6.8 Hz, 1H), 3.96 – 3.82 (m, 3H), 3.73 (dd, *J* = 12.8, 9.4 Hz, 1H), 3.50 (dd, *J* = 12.8, 9.9 Hz, 1H), 2.52 (t, *J* = 7.3 Hz, 2H), 2.45 (app t, *J* = 7.3 Hz, 2H), 2.36 – 2.27 (m, 1H), 2.13 (td, *J* = 6.8, 2.2 Hz, 2H), 1.83 – 1.65 (m, 6H), 1.56 – 1.25 (m, 22H), 1.17 (d, *J* = 6.2 Hz, 3H). **$^{13}$C NMR** (151 MHz, $CD_3OD$) δ 156.0, 151.6, 150.8, 144.8, 119.5, 85.3, 80.9, 76.8 (d, $J_{CP}$ = 12.7 Hz), 65.4 (d, $J_{CP}$ = 160.3 Hz), 64.6 (d, $J_{CP}$ = 5.5 Hz), 34.4, 32.8, 32.2 (d, $J_{CP}$ = 6.3 Hz), 30.8, 30.61, 30.60, 30.4, 30.29, 30.26, 30.2, 29.9, 29.8, 29.2, 27.1, 25.9, 19.4, 16.8 (*27 out of 30 carbon signals observed due to chemical shift equivalence*). **$^{31}$P NMR** (243 MHz, $CD_3OD$) δ 15.5. **HRMS** (ESI) *m/z* calculated for $C_{30}H_{49}N_5O_4PS^-$ [M - H]$^-$: 606.32484, found 606.32492. **LC-MS** (ESI, C8, 0.5 mL/min) 55-95% MeCN in $H_2O$, 6 min, rt = 1.857 min, *m/z* = 608.3 [M + H]$^+$; 40-95% MeCN in $H_2O$, 6 min, rt = 4.488 min, *m/z* = 608.3 [M + H]$^+$.

***Ammonium 3-((12-cyclohexyldodecyl)thio)propyl (R)-(((1-(6-amino-9H-purin-9-yl)propan-2-yl)oxy)methyl)phosphonate* (16h).** Synthesis was carried out according to *General Procedure for DCC Coupling* using compound **14h** (239 mg, 0.696 mmol, 1.00 eq). After quenching with $H_2O$, the reaction mixture was purified via normal-phase column chromatography eluting along a gradient of 0-80% 80:20:3 DCM:MeOH:$NH_4OH$ in DCM. The product fractions were then collected and concentrated under reduced pressure. The obtained residue was subsequently taken

up in a solution of MeOH (8 mL) and $H_2O$ (2 mL), and diammonium phosphate (460 mg, 3.48 mmol, 5.00 eq) was added. The heterogeneous mixture was stirred at room temperature overnight and then immediately purified by reversed-phase column chromatography eluting along a gradient of 0-85% MeOH in $H_2O$. The product fractions were once more collected and concentrated under reduced pressure. Finally, the acquired solid was washed with acetone and dried under high vacuum to yield a white powder (201 mg, 0.329 mmol, 47% yield). MP = Decomp. 162-166 $^oC$. **TLC** (80:20:3 DCM:MeOH:$NH_4OH$) $R_f$ = 0.34. $^1$**H NMR** (600 MHz, $CD_3OD$) δ 8.32 (s, 1H), 8.20 (s, 1H), 4.38 (dd, *J* = 14.4, 3.1 Hz, 1H), 4.24 (dd, *J* = 14.4, 6.7 Hz, 1H), 3.94 – 3.80 (m, 3H), 3.72 (dd, *J* = 12.8, 9.5 Hz, 1H), 3.47 (dd, *J* = 12.7, 10.1 Hz, 1H), 2.51 (t, *J* = 7.3 Hz, 2H), 2.44 (app t, *J* = 7.3 Hz, 2H), 1.82 – 1.61 (m, 7H), 1.56 – 1.47 (m, 2H), 1.39 – 1.12 (m, 27H), 0.93 – 0.83 (m, 2H). $^{13}$**C NMR** (151 MHz, $CD_3OD$) δ 157.0, 153.2, 150.9, 144.3, 119.6, 76.9 (d, $J_{CP}$ = 12.9 Hz), 65.5 (d, $J_{CP}$ = 159.8 Hz), 64.6 (d, $J_{CP}$ = 5.7 Hz), 39.0, 38.7, 34.6, 32.8, 32.2 (d, $J_{CP}$ = 6.4 Hz), 31.1, 30.77, 30.75, 30.74, 30.70, 30.68, 30.4, 29.9, 29.2, 28.0, 27.9, 27.6, 16.8 (*26 out of 30 carbon signals observed due to chemical shift equivalence*). $^{31}$**P NMR** (243 MHz, $CD_3OD$) δ 15.4. **HRMS** (ESI) *m/z* calculated for $C_{30}H_{53}N_5O_4PS^-$ [M - H]$^-$: 610.35614, found 610.35639. **LC-MS** (ESI, C8, 0.5 mL/min) 70-95% MeCN in $H_2O$, 6 min, rt = 1.639 min, *m/z* = 612.4 $[M + H]^+$; 50-95% MeCN in $H_2O$, 6 min, rt = 4.781 min, *m/z* = 612.4 $[M + H]^+$.

***Ammonium 3-((11-(cyclohexyloxy)undecyl)thio)propyl (R)-(((1-(6-amino-9H-purin-9-yl) propan-2-yl)oxy)methyl)phosphonate* (23d).** Synthesis was carried out according to *General Procedure for DCC Coupling* using compound **22d** (240 mg, 0.696 mmol, 1.00 eq). After quenching with $H_2O$, the reaction mixture was purified via normal-phase column chromatography eluting along a gradient of 0-80% 80:20:3 DCM:MeOH:$NH_4OH$ in DCM. The product fractions were then collected and concentrated under reduced pressure. The obtained residue was

subsequently taken up in a solution of MeOH (5 mL) and aqueous ammonia (28-30% $NH_3$ in $H_2O$, 5 mL). The mixture was stirred at room temperature for 20 min and then immediately purified by reversed-phase column chromatography eluting along a gradient of 0-75% MeOH in $H_2O$. The product fractions were once more collected and concentrated under reduced pressure. Finally, the acquired solid was washed with acetone and dried under high vacuum to yield a white powder (205 mg, 0.325 mmol, 47% yield). MP = Decomp. 154-159 $^oC$. **TLC** (80:20:3 DCM:MeOH:$NH_4OH$) $R_f$ = 0.33. **$^1$H NMR** (600 MHz, $CD_3OD$) δ 8.33 (s, 1H), 8.22 (s, 1H), 4.39 (dd, *J* = 14.4, 3.2 Hz, 1H), 4.24 (dd, *J* = 14.4, 6.7 Hz, 1H), 3.95 – 3.81 (m, 3H), 3.73 (dd, *J* = 12.8, 9.4 Hz, 1H), 3.49 (dd, *J* = 12.8, 9.9 Hz, 1H), 3.46 (t, *J* = 6.6 Hz, 2H), 3.28 – 3.21 (m, 1H), 2.51 (t, *J* = 7.3 Hz, 2H), 2.44 (app t, *J* = 7.3 Hz, 2H), 1.94 – 1.86 (m, 2H), 1.82 – 1.69 (m, 4H), 1.58 – 1.48 (m, 5H), 1.38 – 1.18 (m, 19H), 1.17 (d, *J* = 6.2 Hz, 3H). **$^{13}$C NMR** (151 MHz, $CD_3OD$) δ 156.4, 152.3, 150.8, 144.6, 119.5, 78.9, 76.9 (d, $J_{CP}$ = 12.7 Hz), 69.0, 65.4 (d, $J_{CP}$ = 159.8 Hz), 64.6 (d, $J_{CP}$ = 5.7 Hz), 33.4, 32.8, 32.2 (d, $J_{CP}$ = 6.3 Hz), 31.2, 30.8, 30.70, 30.66, 30.65, 30.6, 30.4, 29.9, 29.2, 27.3, 27.0, 25.2, 16.8 (*26 out of 29 carbon signals observed due to chemical shift equivalence*). **$^{31}$P NMR** (243 MHz, $CD_3OD$) δ 15.5. **HRMS** (ESI) *m/z* calculated for $C_{29}H_{53}N_5O_5PS^+$ $[M + H]^+$: 614.34995, found 614.35060. **LC-MS** (ESI, C8, 0.5 mL/min) 50-95% MeCN in $H_2O$, 6 min, rt = 1.833 min, *m/z* = 614.3 $[M + H]^+$; 35-95% MeCN in $H_2O$, 6 min, rt = 4.415 min, *m/z* = 614.3 $[M + H]^+$.

***Ammonium 3-((16-methoxy-16-oxohexadecyl)thio)propyl (R)-(((1-(6-amino-9H-purin-9-yl) propan-2-yl)oxy)methyl)phosphonate* (23e).** Synthesis was carried out according to *General Procedure for DCC Coupling* using compound **22e** (251 mg, 0.696 mmol, 1.00 eq). After quenching with $H_2O$, the reaction mixture was purified via normal-phase column chromatography eluting along a gradient of 0-70% 80:20:3 DCM:MeOH:$NH_4OH$ in DCM. The product fractions

were then collected and concentrated under reduced pressure. The obtained residue was subsequently taken up in methanolic ammonia (7 N, 10 mL). The mixture was stirred at room temperature for 20 min and then immediately purified by reversed-phase column chromatography eluting along a gradient of 0-85% MeOH in $H_2O$. The product fractions were once more collected and concentrated under reduced pressure. Finally, the acquired solid was washed with acetone and dried under high vacuum to yield a white powder (139 mg, 0.221 mmol, 32% yield). MP = Decomp. 170-175 $^oC$. **TLC** (80:20:3 DCM:MeOH:$NH_4OH$) $R_f$ = 0.33. **$^1$H NMR** (600 MHz, $CD_3OD$) δ 8.32 (s, 1H), 8.20 (s, 1H), 4.38 (dd, *J* = 14.4, 3.2 Hz, 1H), 4.24 (dd, *J* = 14.4, 6.7 Hz, 1H), 3.94 – 3.80 (m, 3H), 3.72 (dd, *J* = 12.8, 9.5 Hz, 1H), 3.65 (s, 3H), 3.47 (dd, *J* = 12.8, 10.0 Hz, 1H), 2.51 (t, *J* = 7.3 Hz, 2H), 2.44 (app t, *J* = 7.3 Hz, 2H), 2.31 (t, *J* = 7.4 Hz, 2H), 1.82 – 1.70 (m, 2H), 1.63 – 1.56 (m, 2H), 1.55 – 1.48 (m, 2H), 1.39 – 1.23 (m, 22H), 1.16 (d, *J* = 6.2 Hz, 3H). **$^{13}$C NMR** (151 MHz, $CD_3OD$) δ 176.1, 157.0, 153.2, 150.9, 144.3, 119.6, 76.9 (d, $J_{CP}$ = 12.8 Hz), 65.5 (d, $J_{CP}$ = 159.9 Hz), 64.6 (d, $J_{CP}$ = 5.7 Hz), 52.0, 34.8, 32.8, 32.2 (d, $J_{CP}$ = 6.3 Hz), 30.8, 30.74, 30.72, 30.71, 30.68, 30.6, 30.4, 30.2, 29.9, 29.2, 26.0, 16.8 (*25 out of 29 carbon signals observed due to chemical shift equivalence*). **$^{31}$P NMR** (243 MHz, $CD_3OD$) δ 15.3. **HRMS** (APCI) *m/z* calculated for $C_{29}H_{51}N_5O_6PS^-$ [M - H]$^-$: 628.33031, found 628.33149. **LC-MS** (ESI, C8, 0.5 mL/min) 55-95% MeCN in $H_2O$, 6 min, rt = 1.218 min, *m/z* = 630.3 [M + H]$^+$; 35-95% MeCN in $H_2O$, 6 min, rt = 4.512 min, *m/z* = 630.3 [M + H]$^+$.

## *In Vitro* Protocols and Data Analysis

*Instrumentation and LC-MS/MS Method Development*

LC-MS/MS analyses were conducted on an Agilent 1260 Infinity II HPLC equipped with an Agilent G6460 triple quadrupole mass spectrometer (Agilent Technologies, USA). Reverse-phase HPLC separation for each compound was achieved with an Agilent InfinityLab Poroshell EC-C8 (2.1 x 50 mm, 2.7 μm) column maintained at 40 $^oC$. The mobile phase used during analyses consisted of MeOH-$H_2O$ (0.1% FA) at a flow rate of 0.5 mL/min. Each method was developed in the presence of the internal standard (ISTD), $d_5$-7-ethoxycoumarin. Precursor and product ion detection was performed with Agilent Jet Stream electrospray positive ionization (ESI+) in multiple reaction monitoring (MRM) mode. All MRM transitions, fragmentor voltages, and collision energies for individual compounds are provided in **Table S1**. Other standard MS conditions were as follows: dwell time of 100 ms; gas flow of 10 L/min; nebulizer pressure of 45 psi; delta EMV of 200 V. Lastly, all acquired data was processed using Agilent 6460 Quantitative Analysis software.

**Table S1.** Scan parameters and corresponding transitions in MRM mode for tested compounds.

| Compound Name | Precursor Ion MS1 | Product Ion MS2 | Fragmentor Voltage (V) | Collision Energy (V) | Cell Accelerator (V) | Polarity |
|---|---|---|---|---|---|---|
| **2b** | 586.4 | 299.3<br>71.2<br>57.2 | 154 | 21<br>45<br>45 | 4 | Positive |
| **2c** | 586.4 | 299.3<br>176.1<br>117.1 | 240 | 29<br>65<br>41 | 4 | Positive |
| **2d** | 586.4 | 288.1<br>176.1 | 182 | 37<br>61 | 4 | Positive |
| **2e** | 586.4 | 288.1<br>270.0<br>176.1 | 240 | 37<br>45<br>65 | 4 | Positive |

| | | | | | | |
|---|---|---|---|---|---|---|
| **2f** | 586.4 | 288.1<br>270.1<br>176.1 | 240 | 37<br>45<br>61 | 4 | Positive |
| **2g** | 530.3 | 243.3<br>75.2 | 172 | 21<br>41 | 4 | Positive |
| **2h** | 558.3 | 271.3 | 192 | 25 | 4 | Positive |
| **2i** | 614.4 | 327.4 | 190 | 25 | 4 | Positive |
| **2j** | 642.4 | 355.3 | 202 | 29 | 4 | Positive |
| **15b** | 582.3 | 295.2 | 172 | 25 | 4 | Positive |
| **15c** | 626.3 | 339.2 | 132 | 21 | 4 | Positive |
| **15d** | 696.4 | 409.3<br>157.1<br>115.0 | 170 | 25<br>29<br>37 | 4 | Positive |
| **15e** | 610.4 | 323.3<br>109.1<br>95.1 | 160 | 25<br>33<br>41 | 4 | Positive |
| **16e** | 614.4 | 327.3<br>57.2 | 170 | 29<br>50 | 4 | Positive |
| **15f** | 602.3 | 315.2<br>129.0 | 172 | 21<br>41 | 4 | Positive |
| **16f** | 606.3 | 319.2 | 172 | 25 | 4 | Positive |
| **23a** | 608.3 | 321.2<br>107.1<br>69.2 | 172 | 25<br>45<br>49 | 4 | Positive |
| **23b** | 624.3 | 337.2 | 134 | 24 | 4 | Positive |
| **15g** | 620.3 | 333.1<br>147.0<br>109.0 | 150 | 21<br>41<br>50 | 4 | Positive |
| **16g** | 624.3 | 337.1<br>109.0<br>75.1 | 158 | 25<br>50<br>45 | 4 | Positive |
| **23c** | 626.3 | 339.2<br>97.1 | 170 | 25<br>41 | 4 | Positive |
| **15h** | 608.3 | 321.2<br>95.1<br>81.1 | 170 | 21<br>37<br>49 | 4 | Positive |

| | | | | | | |
|---|---|---|---|---|---|---|
| **16h** | 612.4 | 325.2<br>83.1 | 150 | 25<br>49 | 4 | Positive |
| **23d** | 614.4 | 327.3<br>245.2 | 170 | 25<br>33 | 4 | Positive |
| **23e** | 630.4 | 343.3 | 180 | 29 | 4 | Positive |
| **Diphenhydramine** | 256.2 | 167.1<br>152.0 | 78 | 8<br>44 | 4 | Positive |
| **Verapamil** | 455.1 | 303.2<br>165.1 | 126 | 24<br>28 | 4 | Positive |
| **Procaine** | 237.2 | 120.0<br>100.2 | 98 | 25<br>13 | 4 | Positive |
| ***d*$_5$-7-Ethoxycoumarin (ISTD)** | 196.1 | 164.0 | 116 | 17 | 4 | Positive |

*Metabolic Stability Assays*

<u>Liver Microsome Stability</u>

*<u>Liver Microsome Stability Assay Setup Example</u>*

**Test Compound:** 928.4 µL Potassium Phosphate Buffer (100 mM) + 55 µL HLM or MLM + 110 µL NADPH (10 mM) + 6.6 µL TC (500 µM).

**Positive Control:** 464.2 µL Potassium Phosphate Buffer (100 mM) + 27.5 µL HLM or MLM + 55 µL NADPH (10 mM) + 3.3 µL Verapamil or Diphenhydramine (500 µM).

**Negative Control:** 141.6 µL Potassium Phosphate Buffer (100 mM) + 7.5 µL HLM or MLM + 0.9 µL TC (500 µM).

**Quenching Mixture:** 100 µL MeOH with ISTD (2 µM d5-7-ethoxycoumarin).

**Final Volume After Quenching:** 200 µL (100 µL from reaction mixture + 100 µL quencher solution; ISTD final concentration of 1.0 µM).

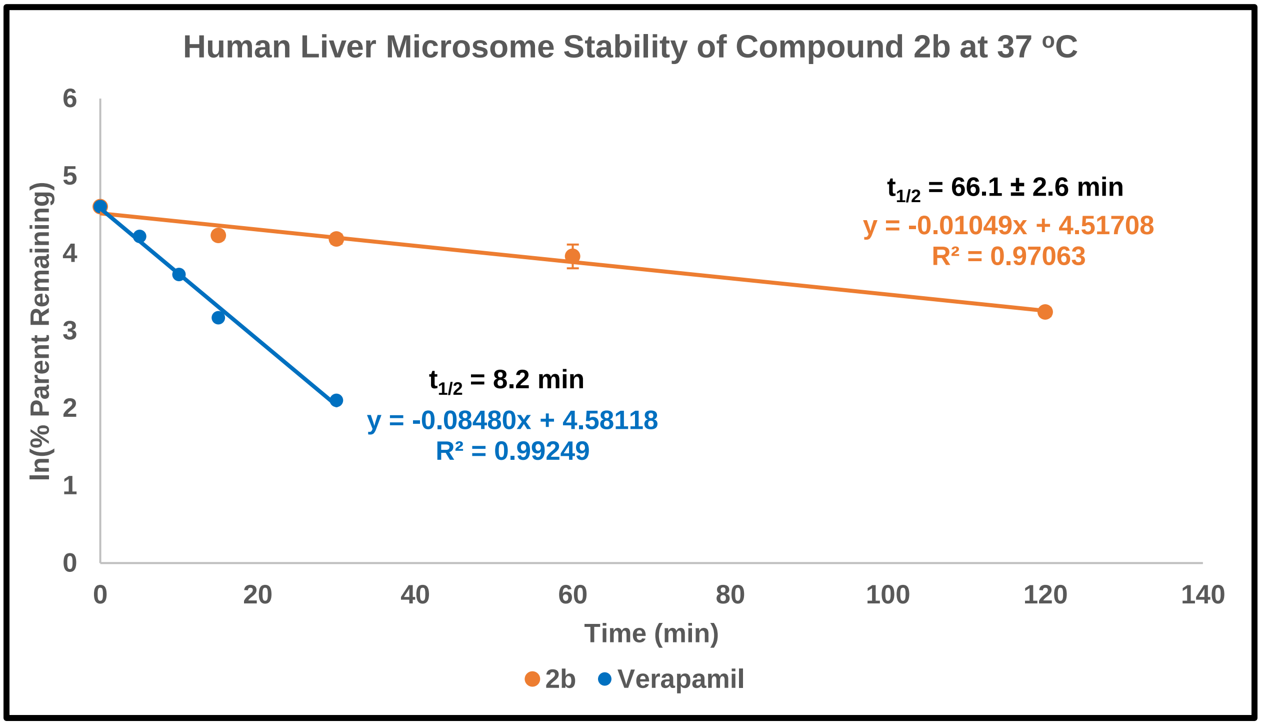


**Figure S1.** *In vitro* HLM stability of **2b** (5 points over 120 min) with verapamil serving as a positive control. A half-life of 66.1 ± 2.6 min was calculated for **2b**.

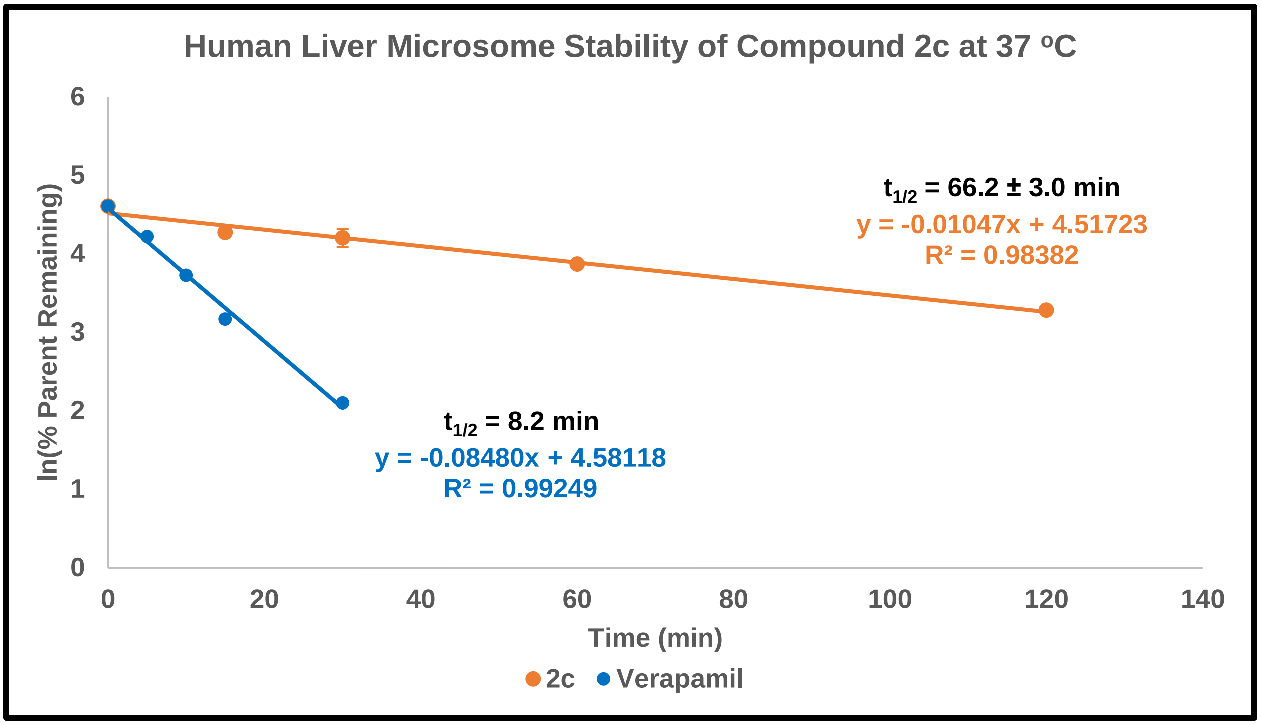


**Figure S2.** *In vitro* HLM stability of **2c** (5 points over 120 min) with verapamil serving as a positive control. A half-life of 66.2 ± 3.0 min was calculated for **2c**.

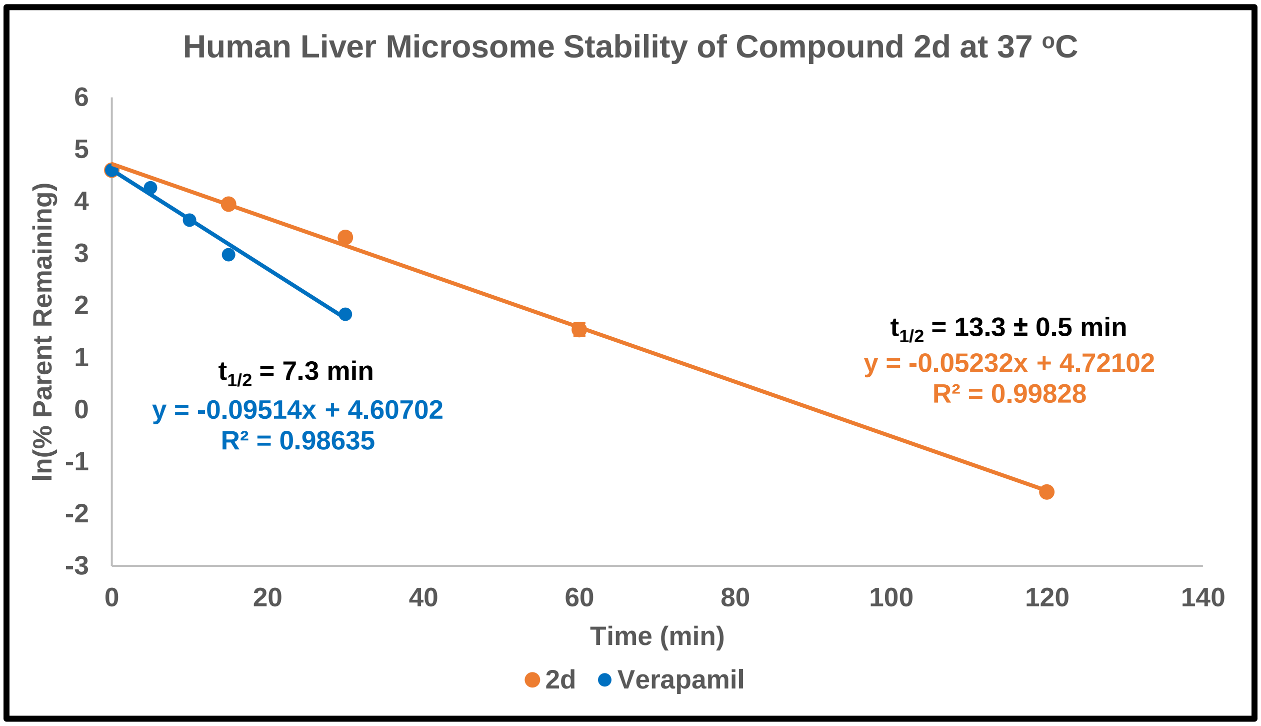


**Figure S3.** *In vitro* HLM stability of **2d** (5 points over 120 min) with verapamil serving as a positive control. A half-life of 13.3 ± 0.5 min was calculated for **2d**.

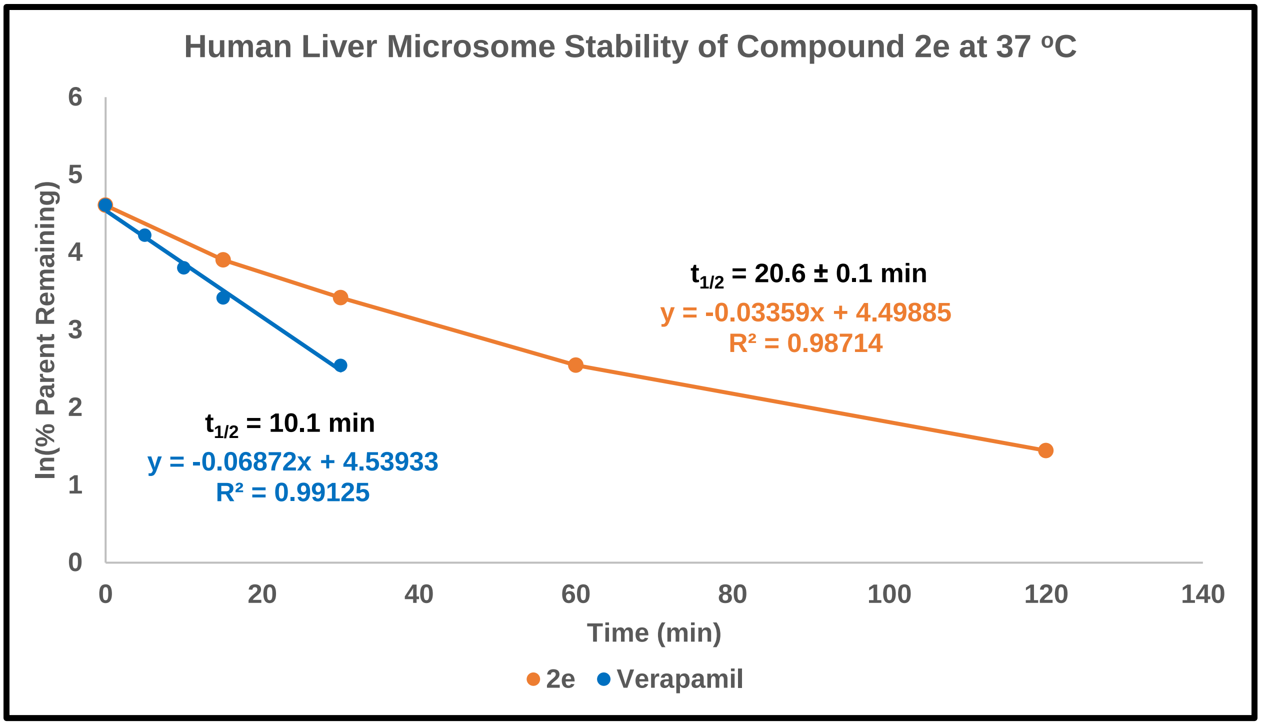


**Figure S4.** *In vitro* HLM stability of **2e** (5 points over 120 min) with verapamil serving as a positive control. A half-life of 20.6 ± 0.1 min was calculated for **2e**.

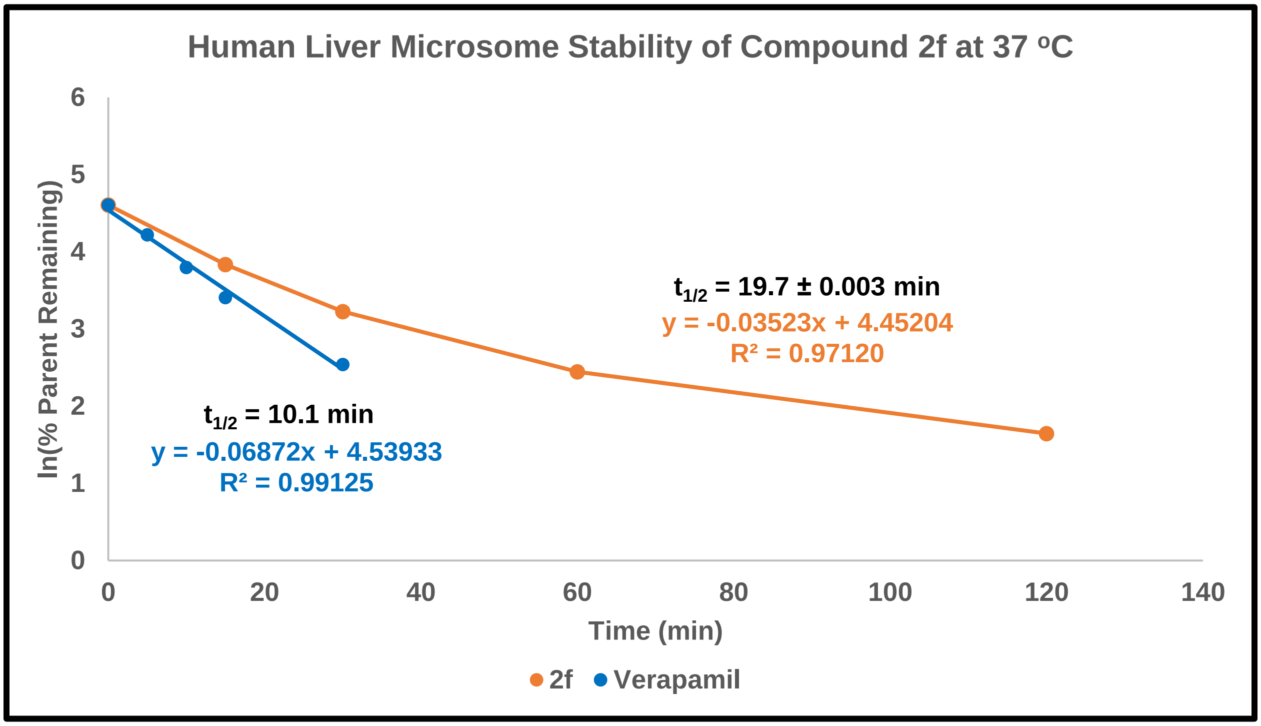


**Figure S5.** *In vitro* HLM stability of **2f** (5 points over 120 min) with verapamil serving as a positive control. A half-life of 19.7 ± 0.003 min was calculated for **2f**.

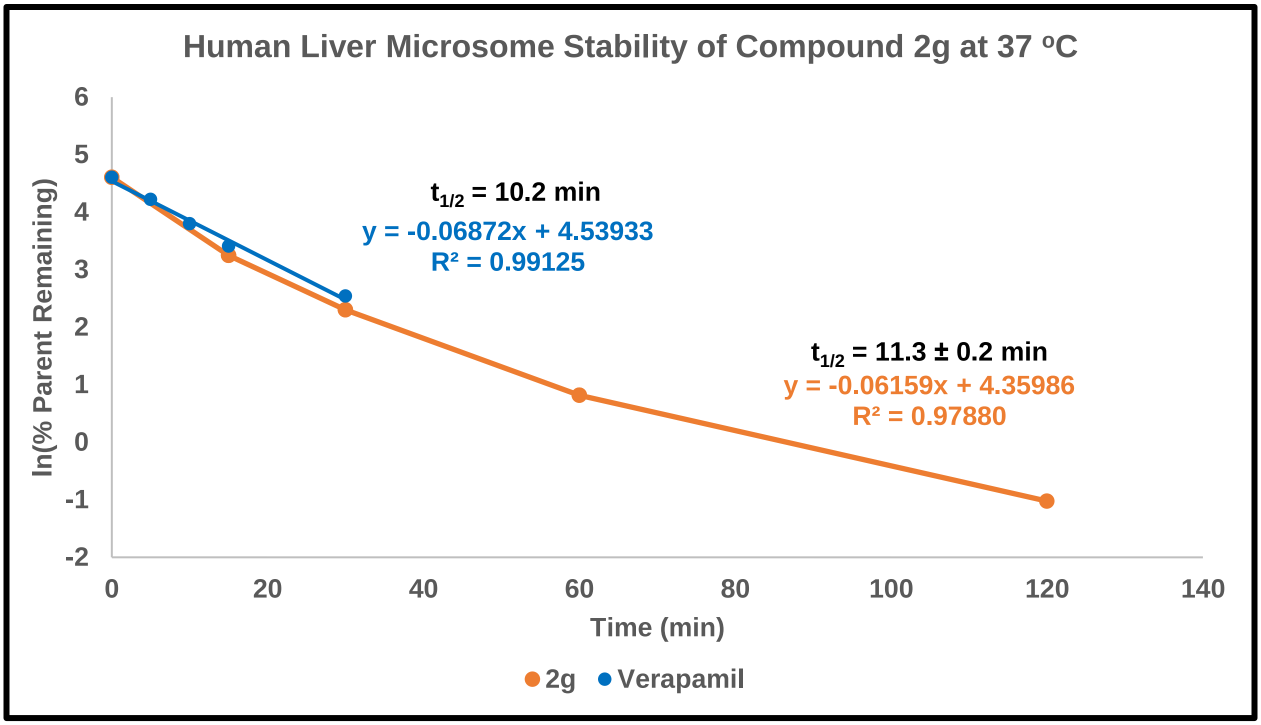


**Figure S6.** *In vitro* HLM stability of **2g** (5 points over 120 min) with verapamil serving as a positive control. A half-life of 11.3 ± 0.2 min was calculated for **2g**.

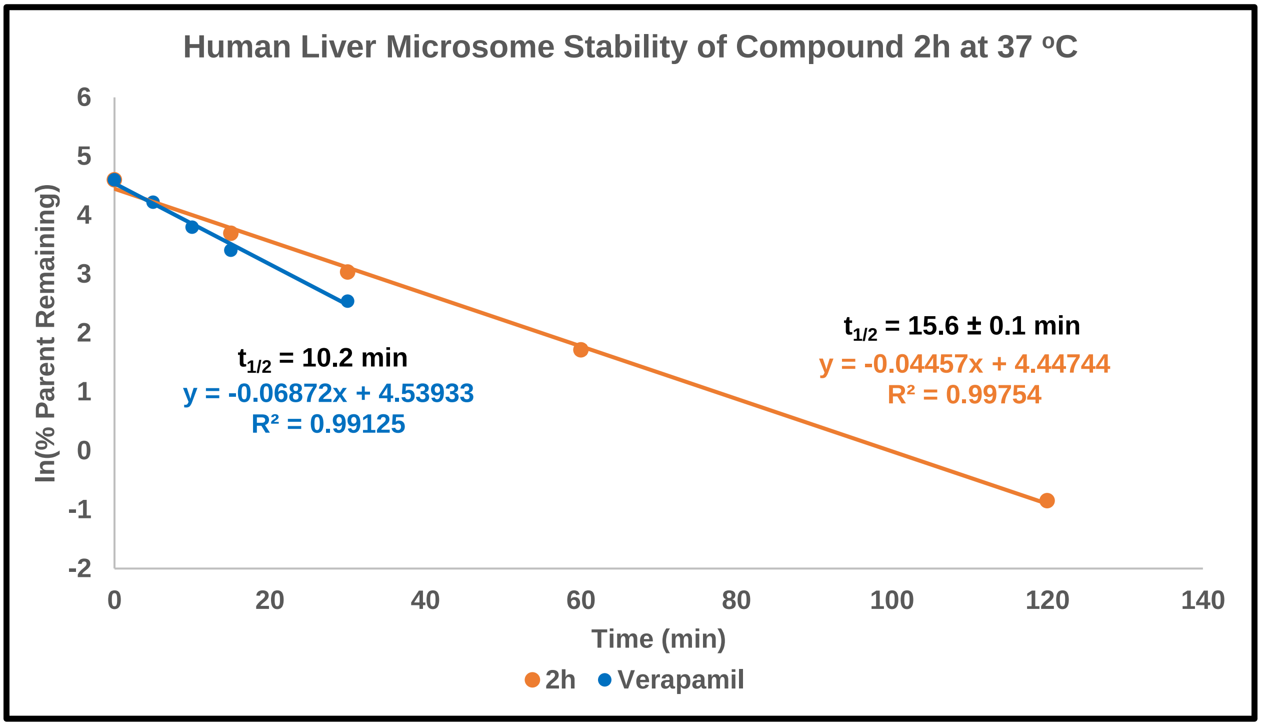


**Figure S7.** *In vitro* HLM stability of **2h** (5 points over 120 min) with verapamil serving as a positive control. A half-life of 15.6 ± 0.1 min was calculated for **2h**.

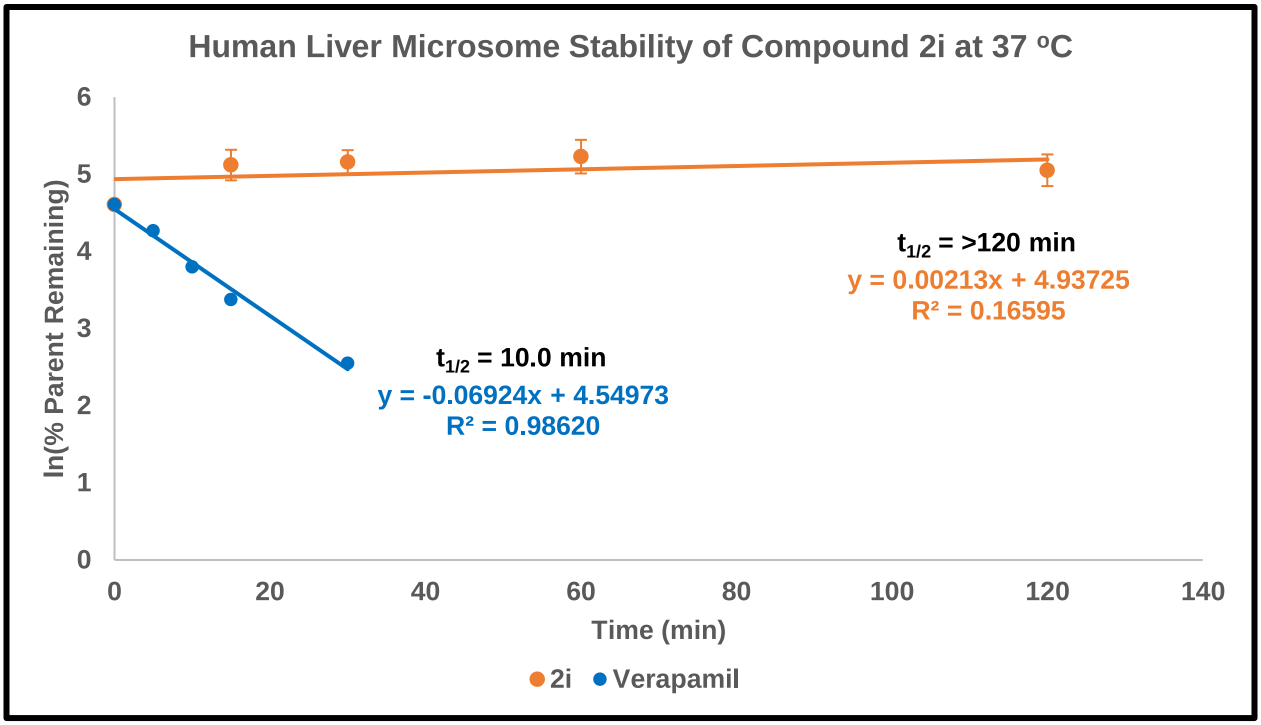


**Figure S8.** *In vitro* HLM stability of **2i** (5 points over 120 min) with verapamil serving as a positive control. A half-life greater than 120 min (>95% remaining) was calculated for **2i**.

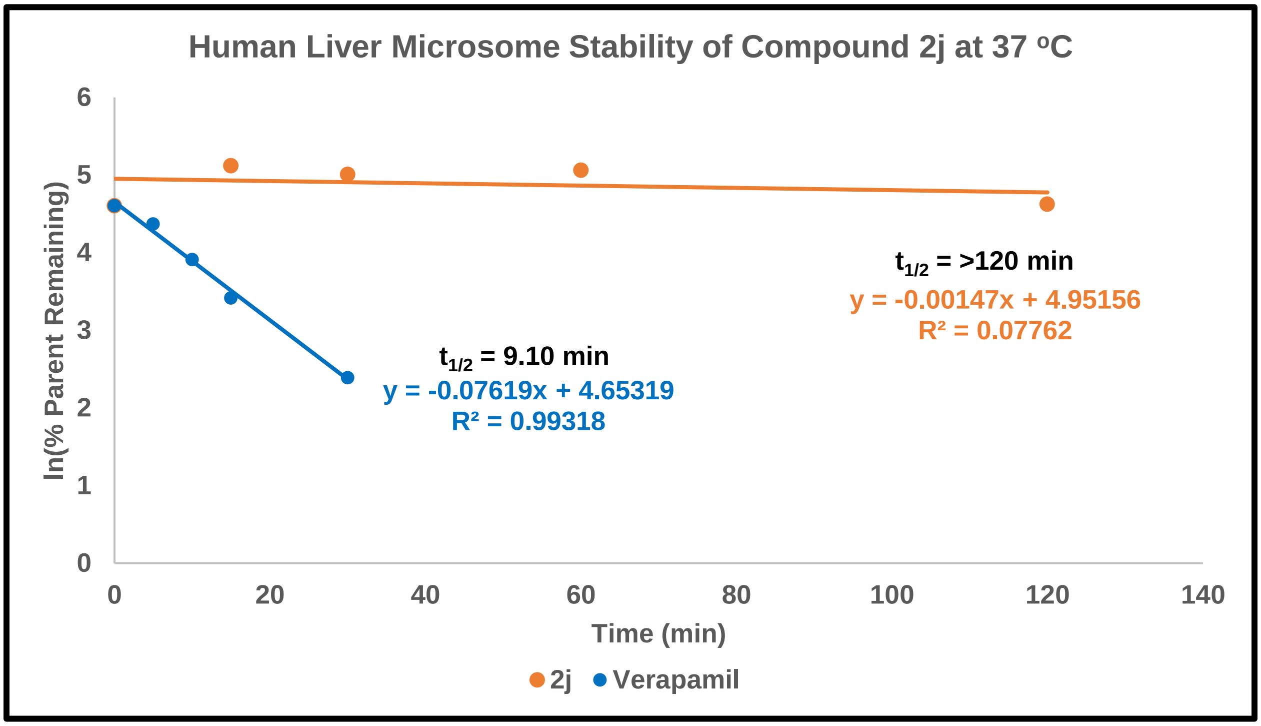


**Figure S9.** *In vitro* HLM stability of **2j** (5 points over 120 min) with verapamil serving as a positive control. A half-life greater than 120 min (>95% remaining) was calculated for **2j**.

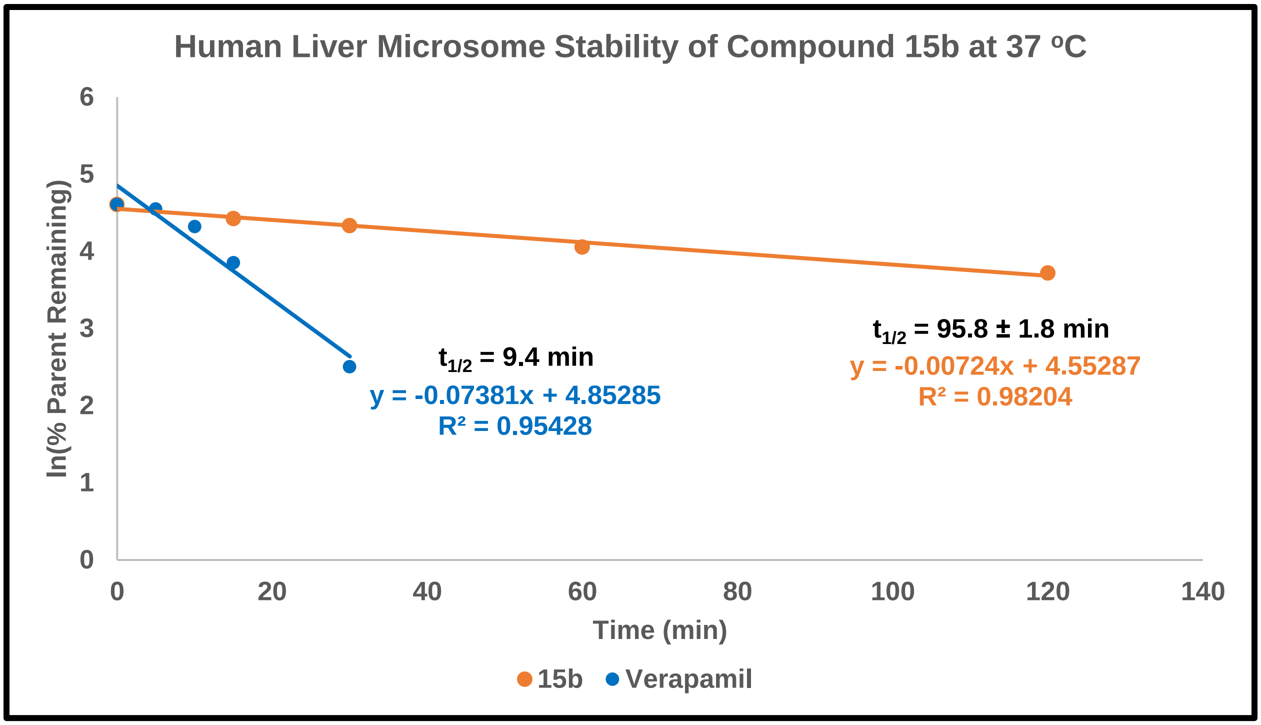


**Figure S10.** *In vitro* HLM stability of **15b** (5 points over 120 min) with verapamil serving as a positive control. A half-life of 95.8 ± 1.8 min was calculated for **15b**.

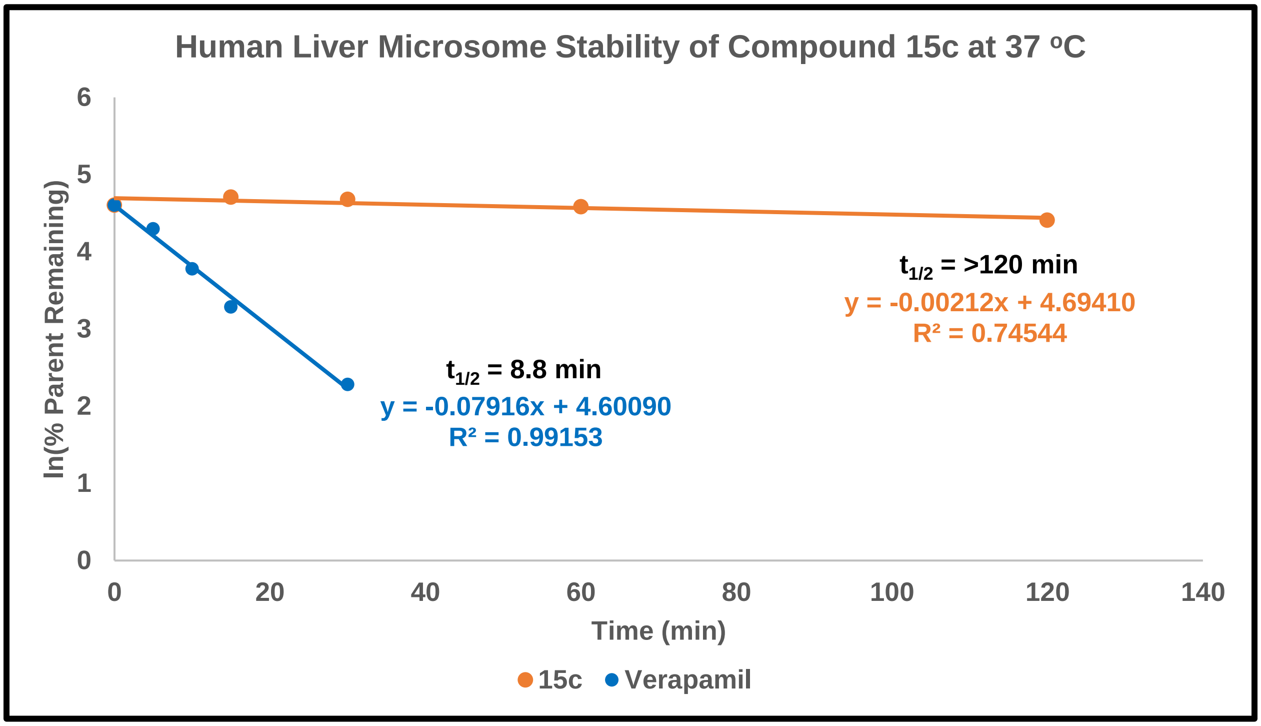


**Figure S11.** *In vitro* HLM stability of **15c** (5 points over 120 min) with verapamil serving as a positive control. A half-life greater than 120 min (82% remaining) was calculated for **15c**.

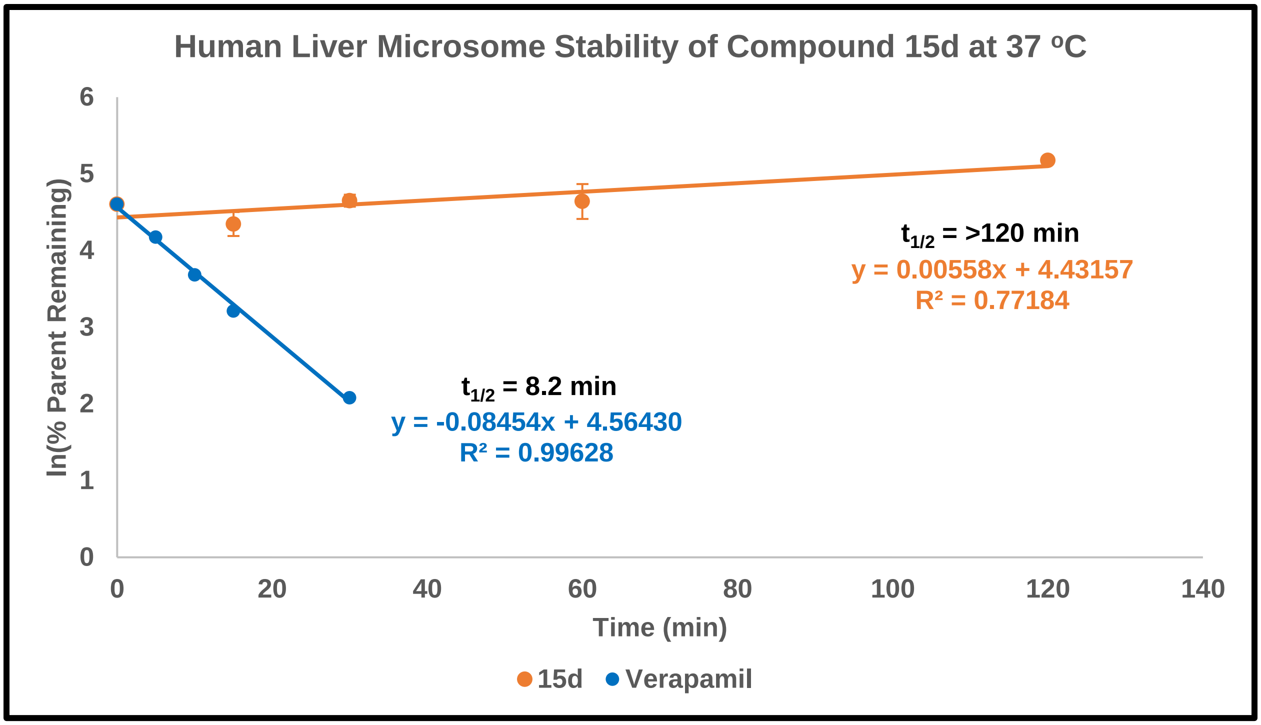


**Figure S12.** In vitro HLM stability of **15d** (5 points over 120 min) with verapamil serving as a positive control. A half-life greater than 120 min (>95% remaining) was calculated for **15d**.

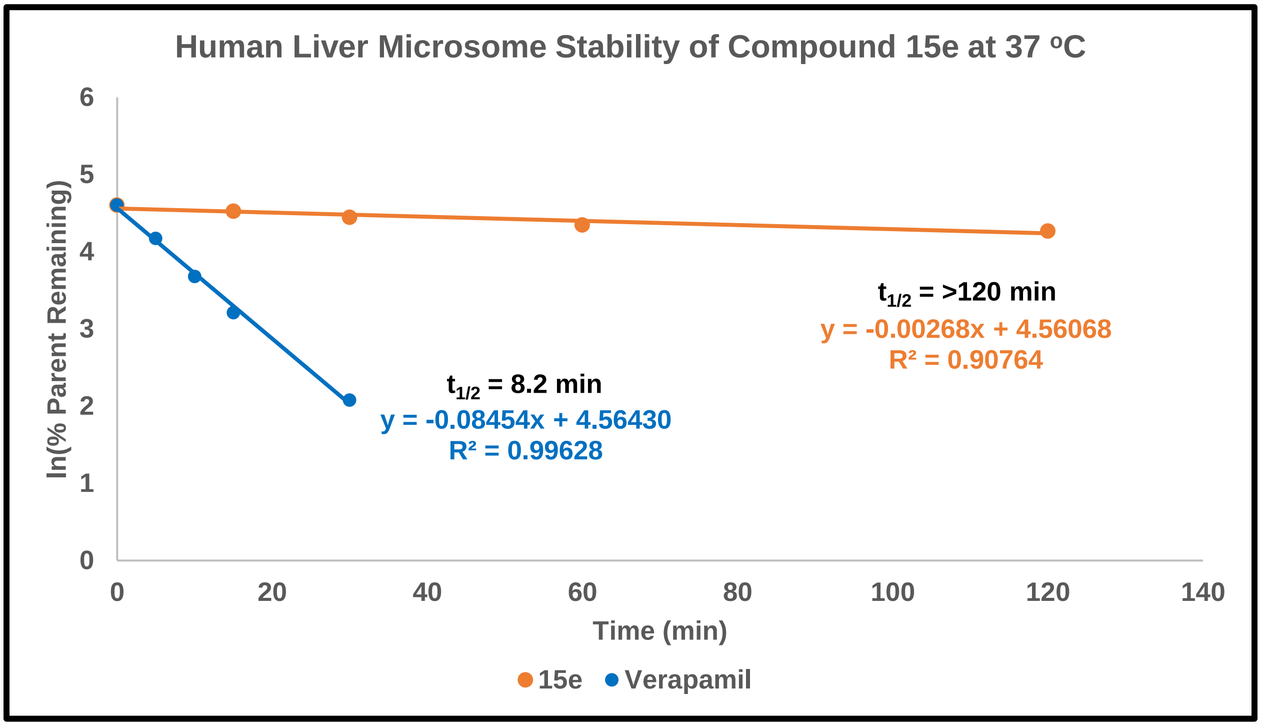


**Figure S13.** *In vitro* HLM stability of **15e** (5 points over 120 min) with verapamil serving as a positive control. A half-life greater than 120 min (71% remaining) was calculated for **15e**.

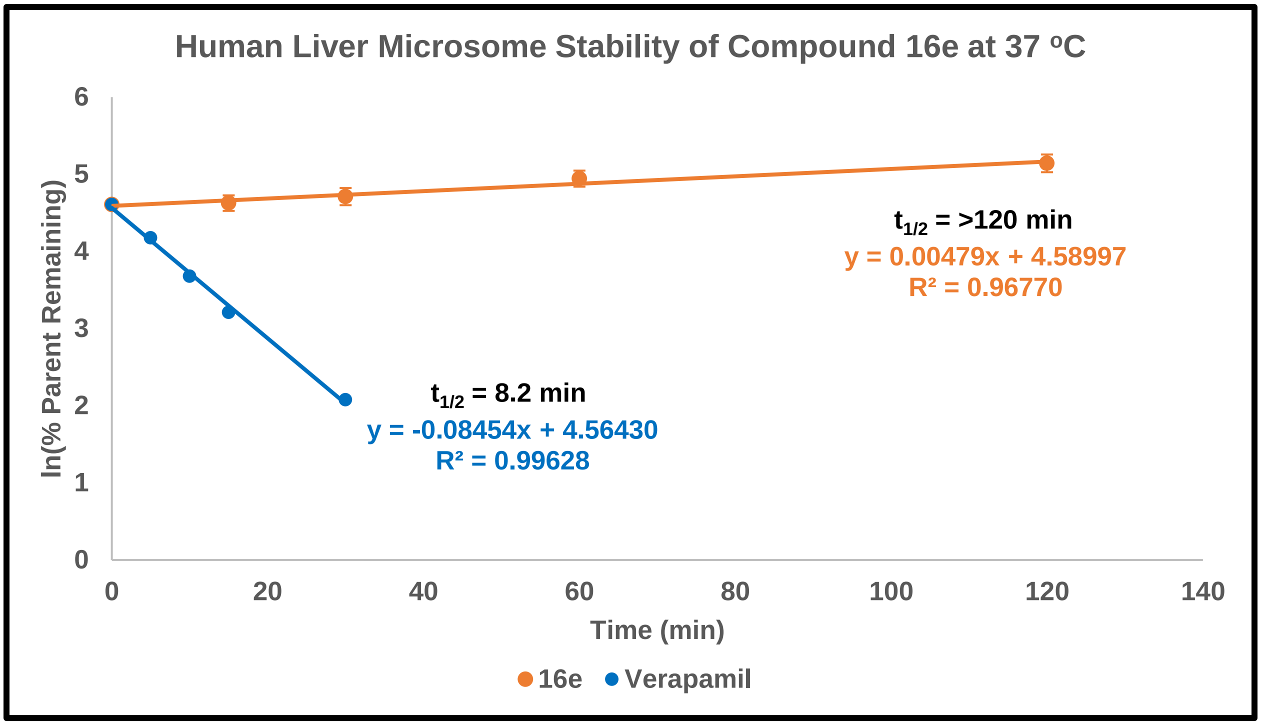


**Figure S14.** *In vitro* HLM stability of **16e** (5 points over 120 min) with verapamil serving as a positive control. A half-life greater than 120 min (>95% remaining) was calculated for **16e**.

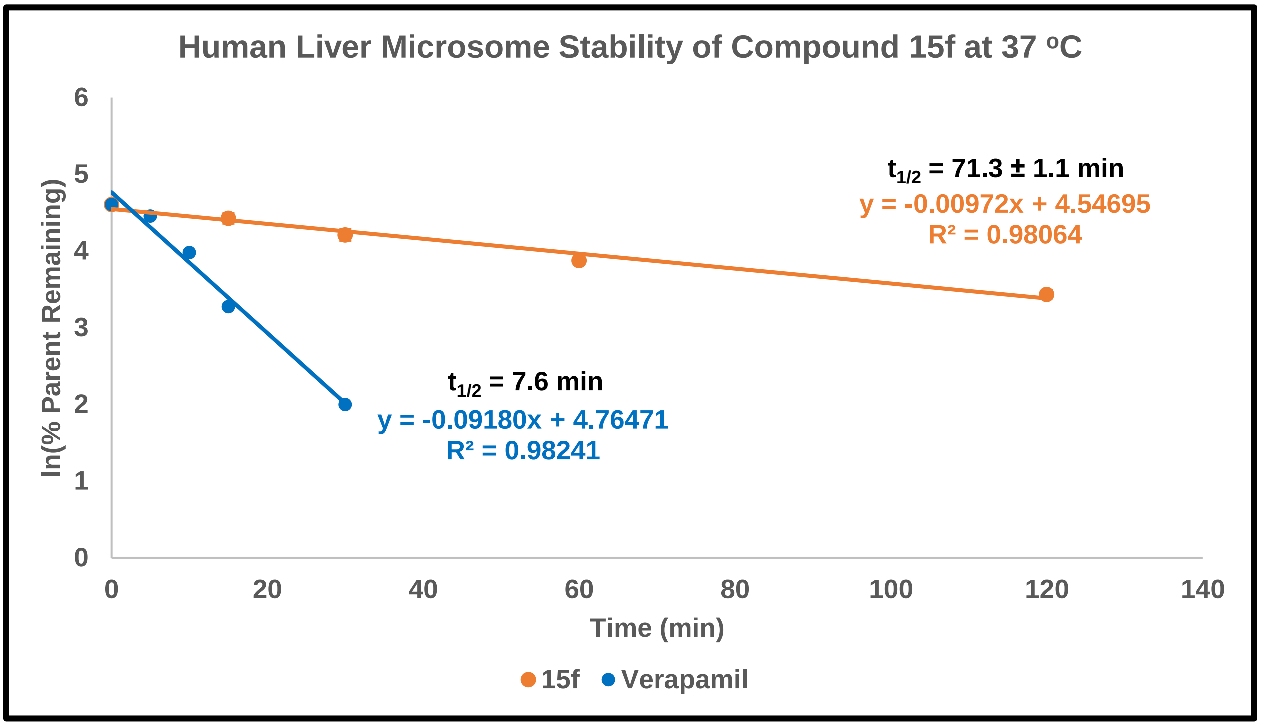


**Figure S15.** *In vitro* HLM stability of **15f** (5 points over 120 min) with verapamil serving as a positive control. A half-life of 71.3 ± 1.1 min was calculated for **15f**.

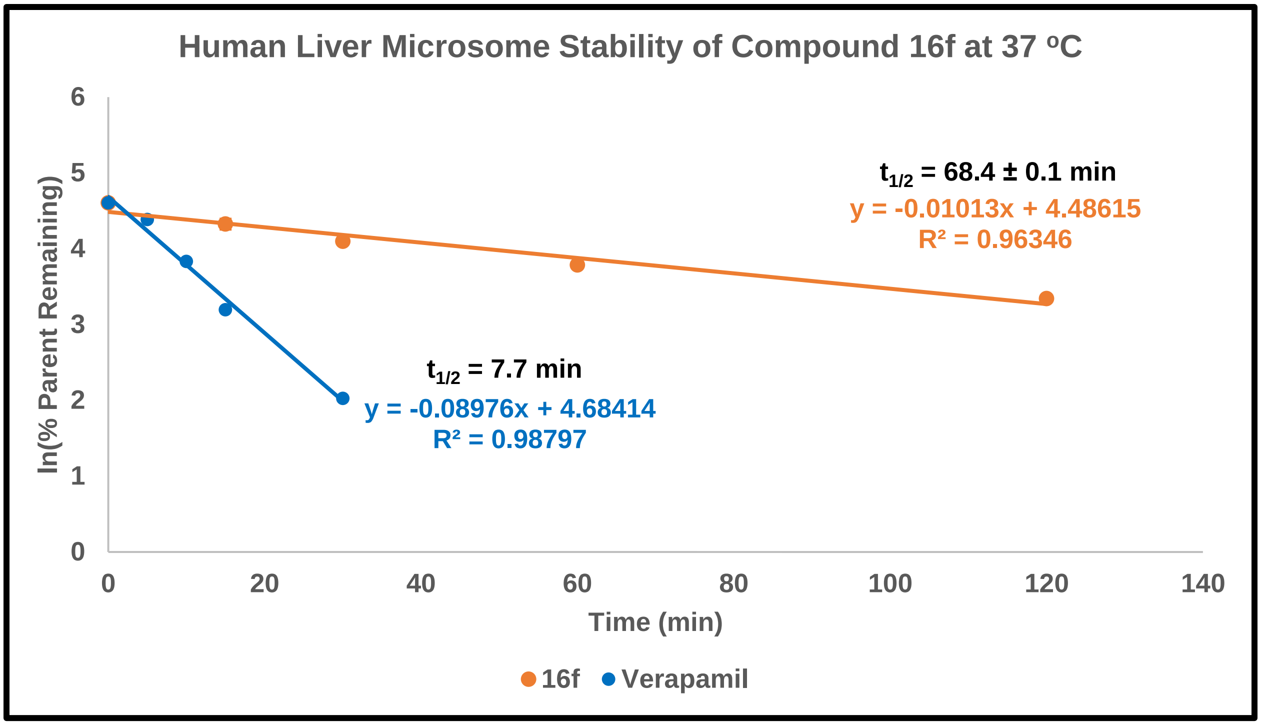


**Figure S16.** *In vitro* HLM stability of **16f** (5 points over 120 min) with verapamil serving as a positive control. A half-life of 68.4 ± 0.1 min was calculated for **16f**.

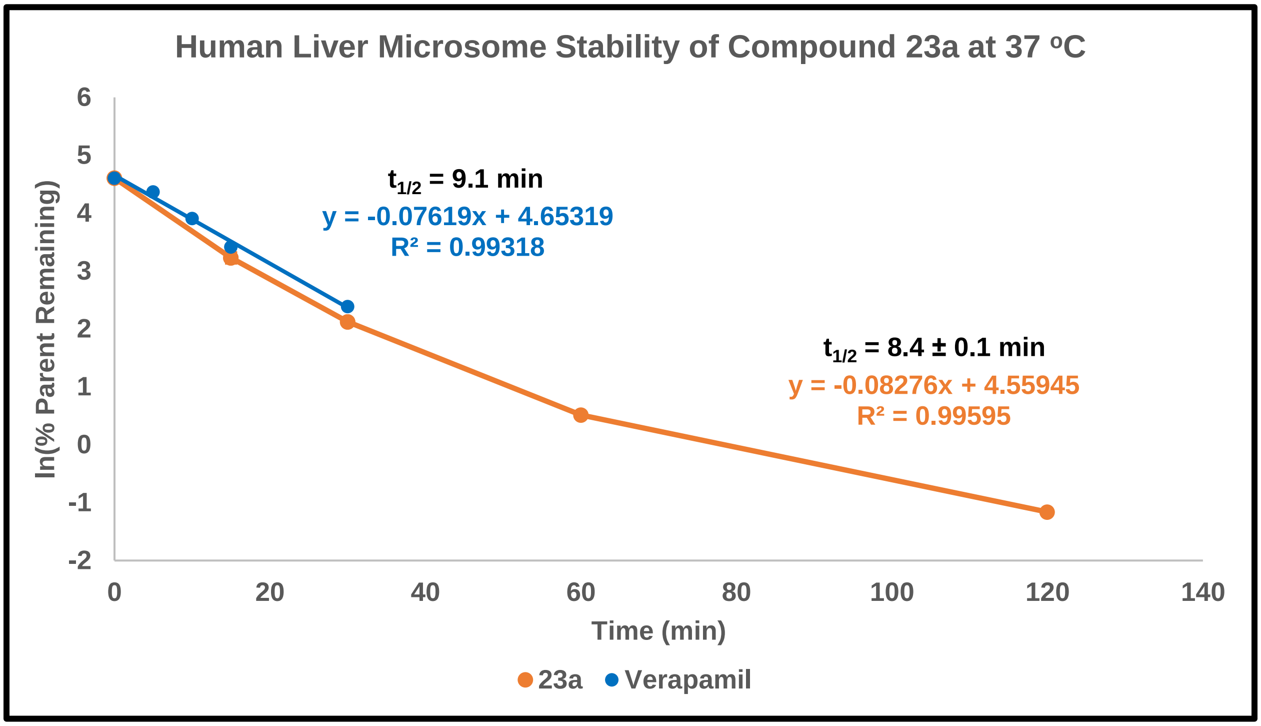


**Figure S17.** *In vitro* HLM stability of **23a** (5 points over 120 min) with verapamil serving as a positive control. A half-life of 8.4 ± 0.1 min was calculated for **23a**.

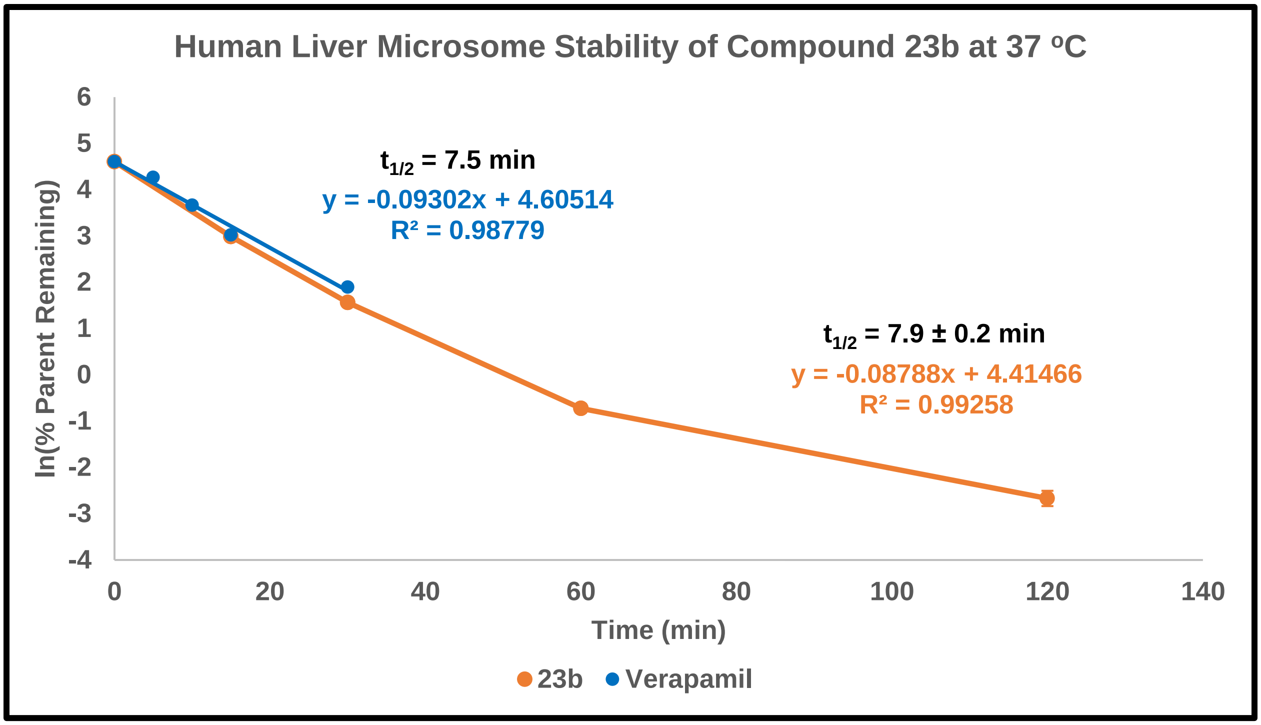


**Figure S18.** *In vitro* HLM stability of **23b** (5 points over 120 min) with verapamil serving as a positive control. A half-life of 7.9 ± 0.2 min was calculated for **23b**.

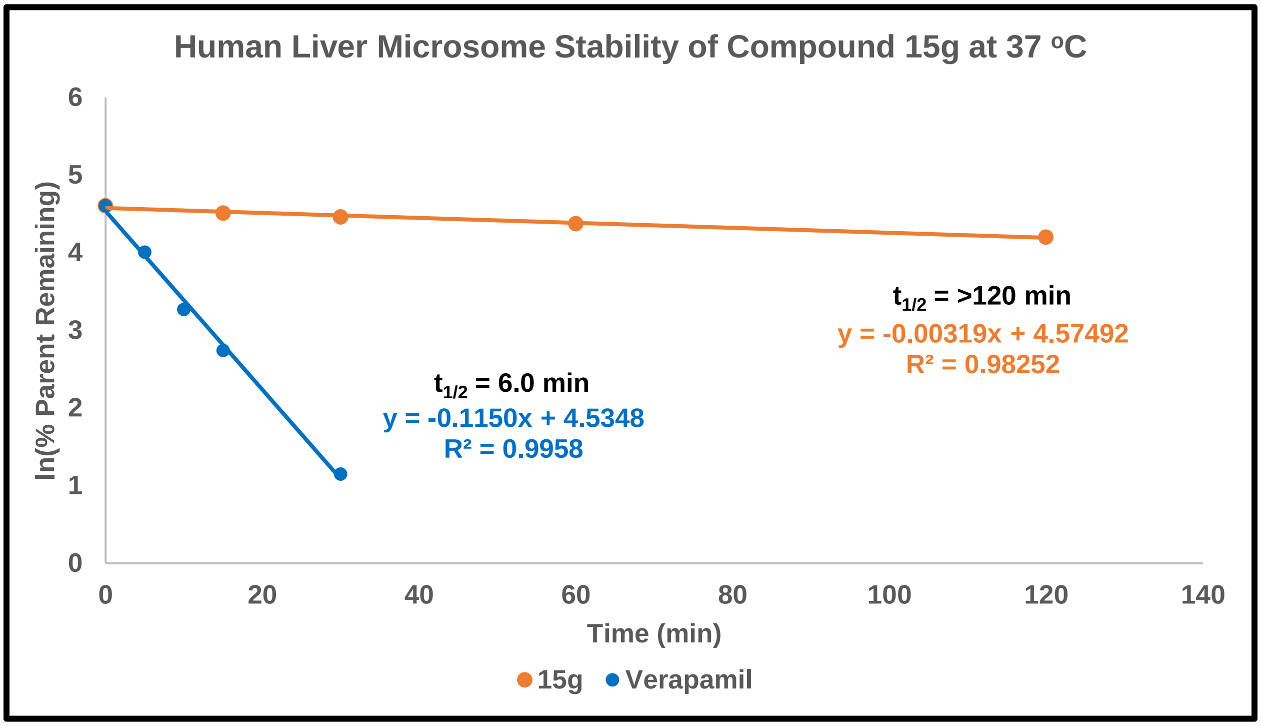


**Figure S19.** *In vitro* HLM stability of **15g** (5 points over 120 min) with verapamil serving as a positive control. A half-life greater than 120 min (67% remaining) was calculated for **15g**.

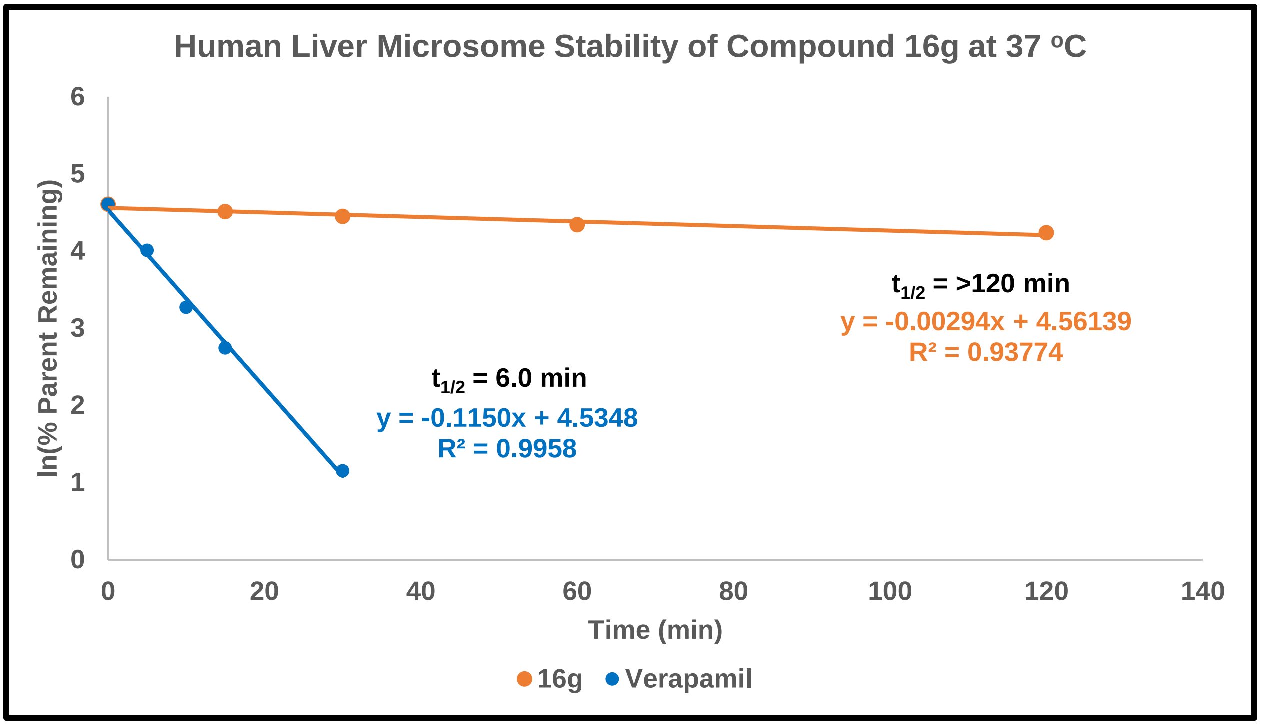


**Figure S20.** *In vitro* HLM stability of **16g** (5 points over 120 min) with verapamil serving as a positive control. A half-life greater than 120 min (69% remaining) was calculated for **16g**.

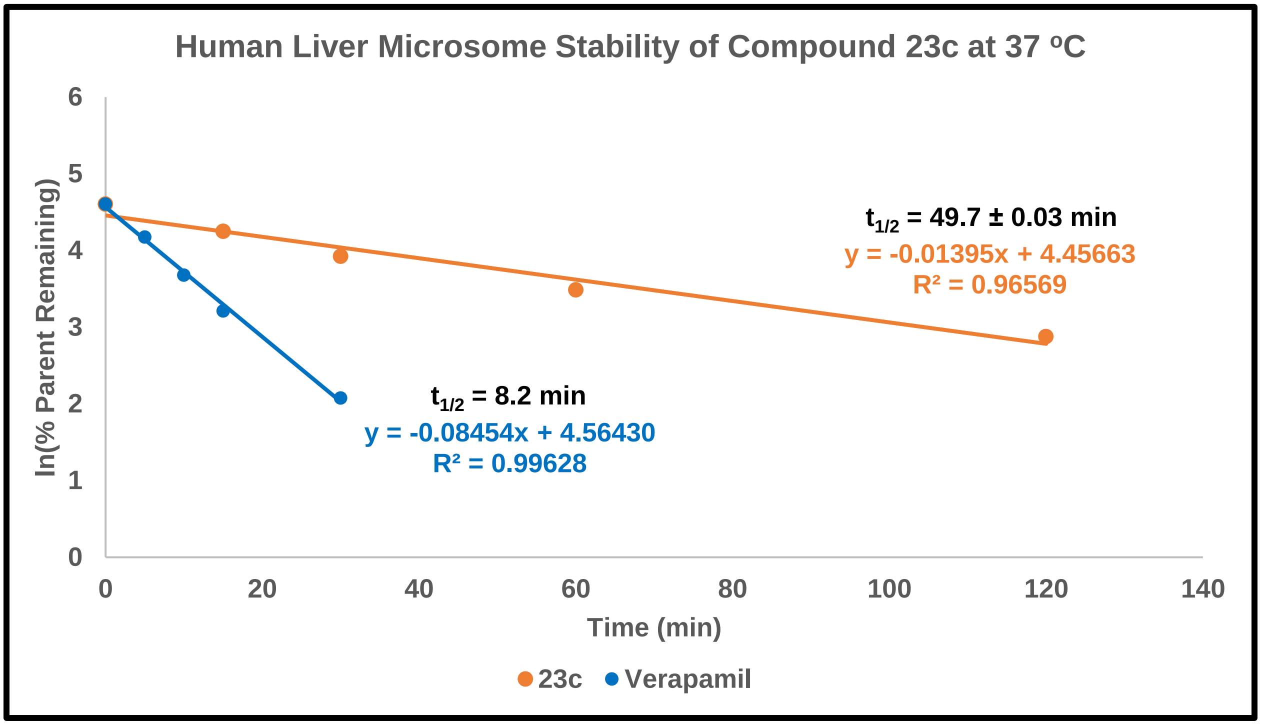


**Figure S21.** *In vitro* HLM stability of **23c** (5 points over 120 min) with verapamil serving as a positive control. A half-life of 49.7 ± 0.03 min was calculated for **23c**.

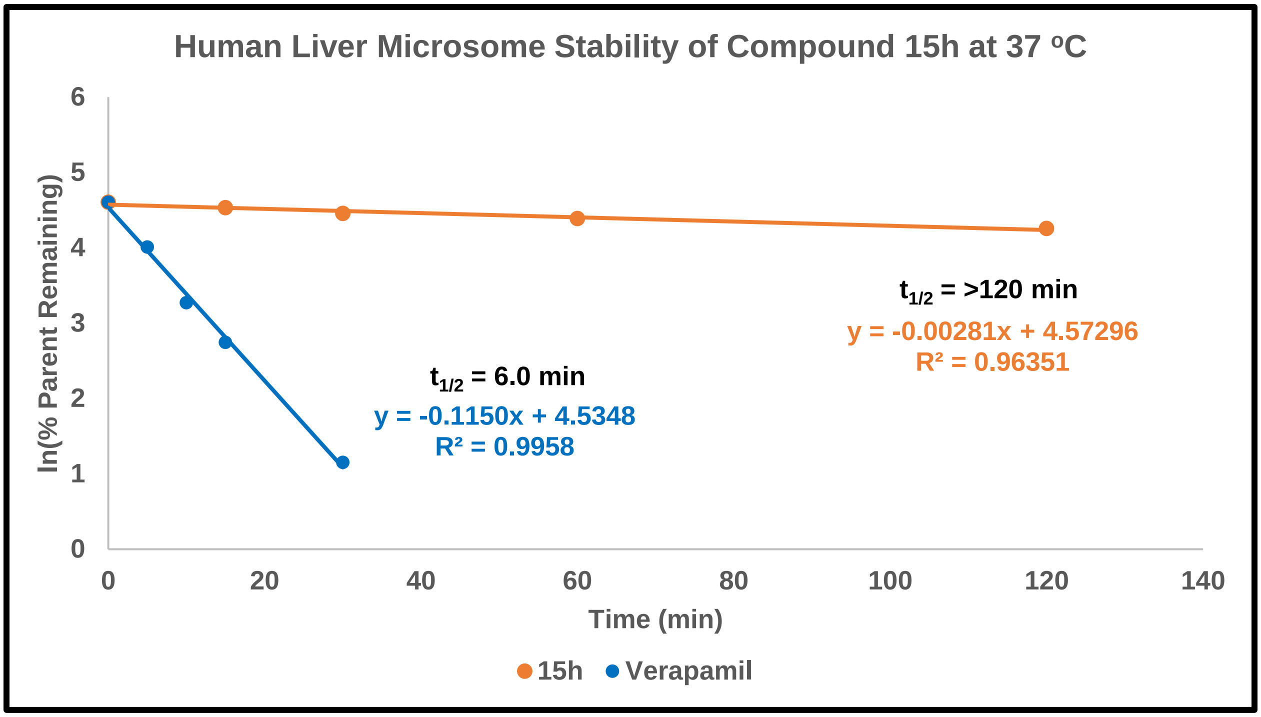


**Figure S22.** In vitro HLM stability of **15h** (5 points over 120 min) with verapamil serving as a positive control. A half-life greater than 120 min (70% remaining) was calculated for **15h**.

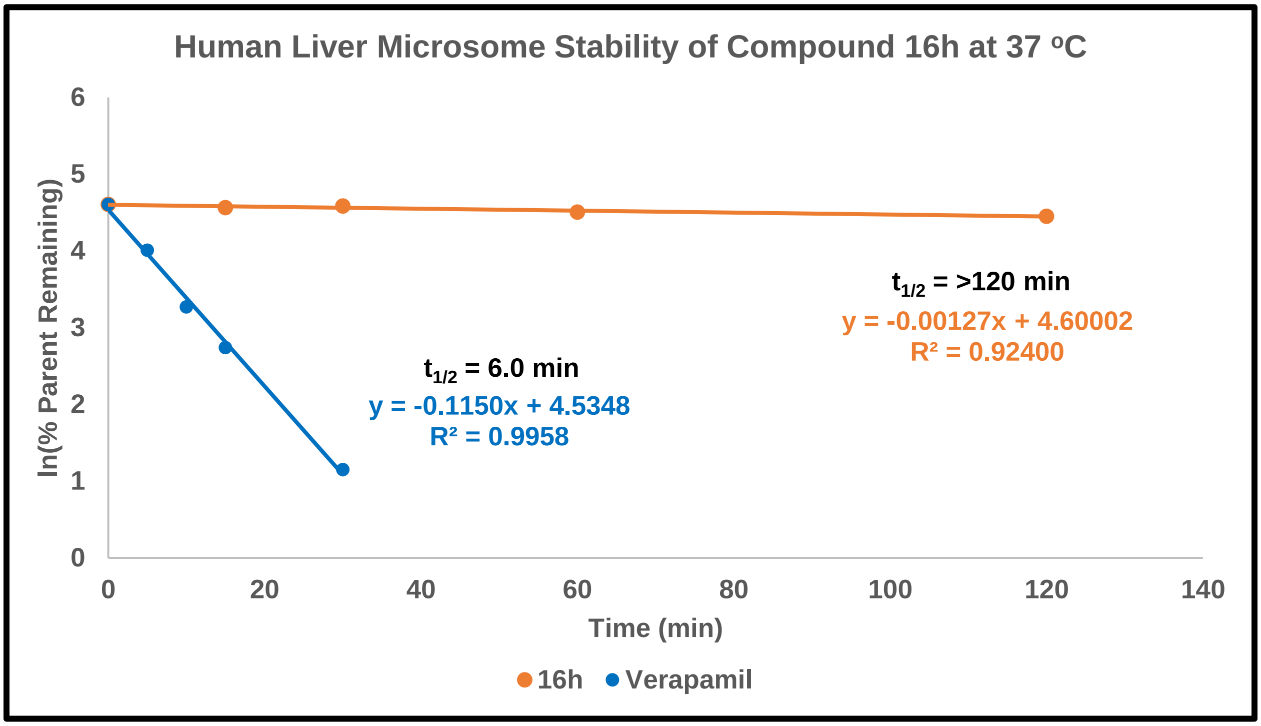


**Figure S23.** *In vitro* HLM stability of **16h** (5 points over 120 min) with verapamil serving as a positive control. A half-life greater than 120 min (85% remaining) was calculated for **16h**.

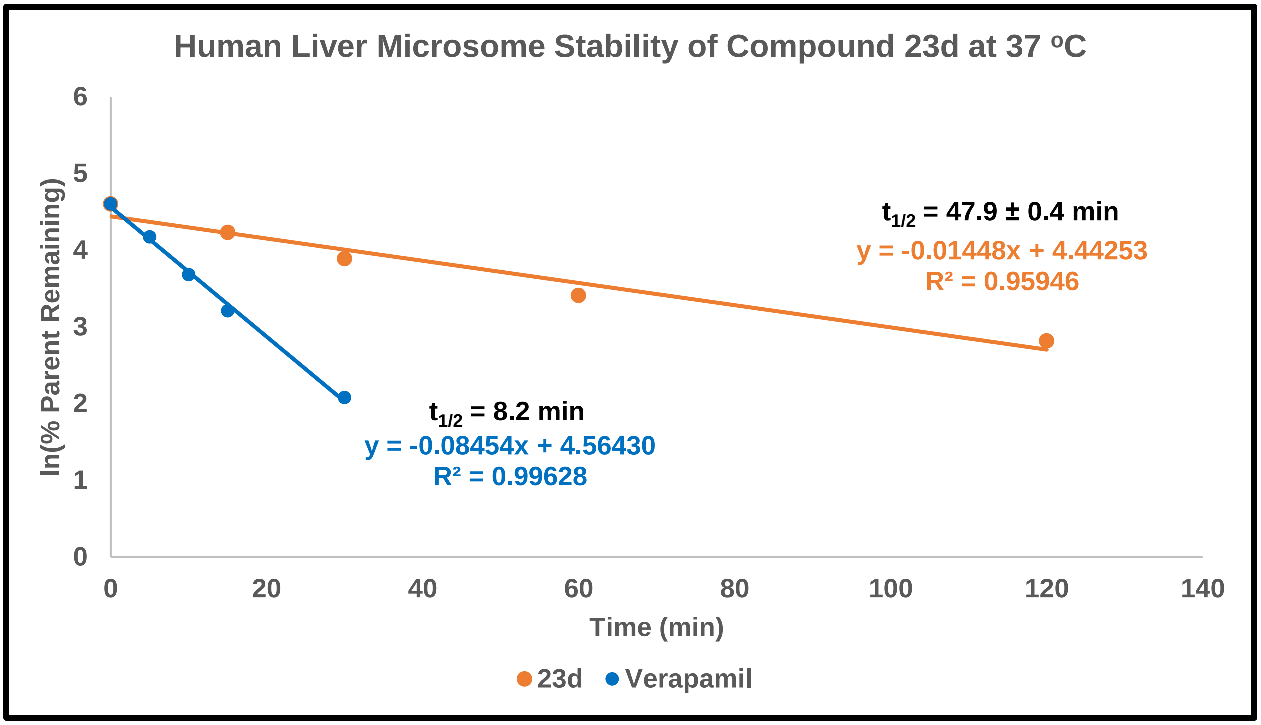


**Figure S24.** *In vitro* HLM stability of **23d** (5 points over 120 min) with verapamil serving as a positive control. A half-life of 47.9 ± 0.4 min was calculated for **23d**.

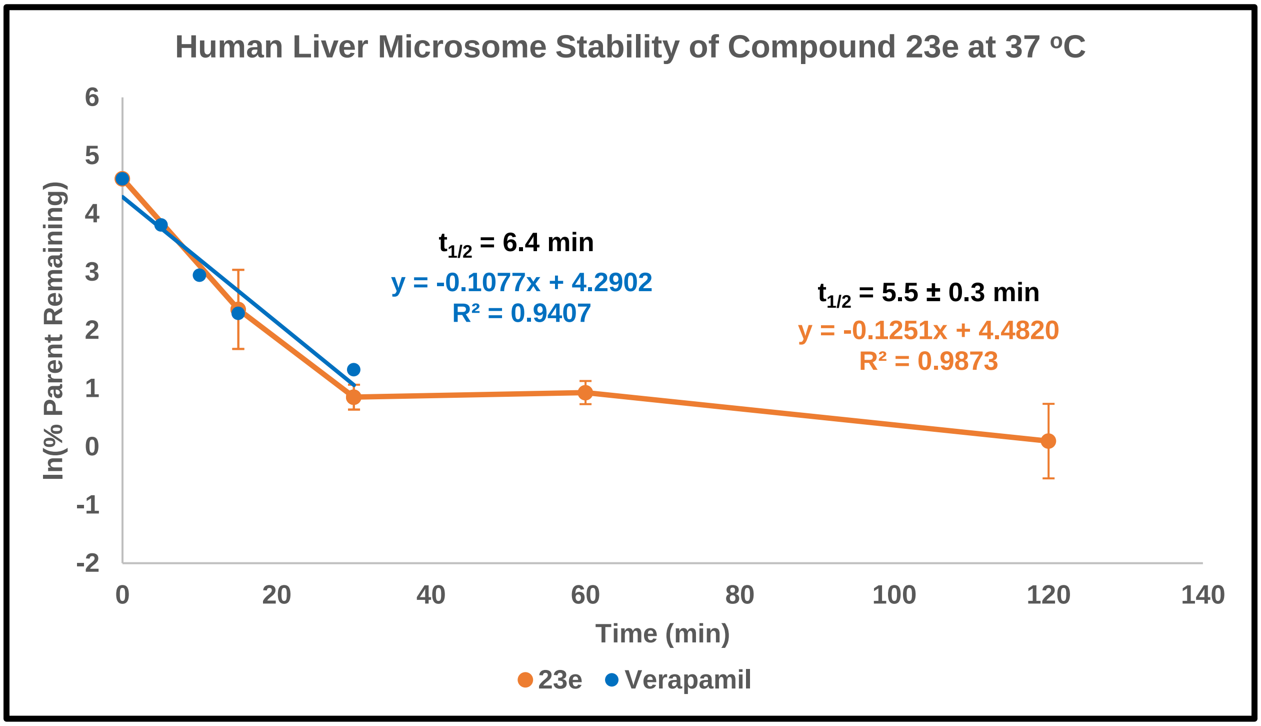


**Figure S25.** *In vitro* HLM stability of **23e** (5 points over 120 min) with verapamil serving as a positive control. A half-life of 5.5 ± 0.3 min was calculated for **23e**.

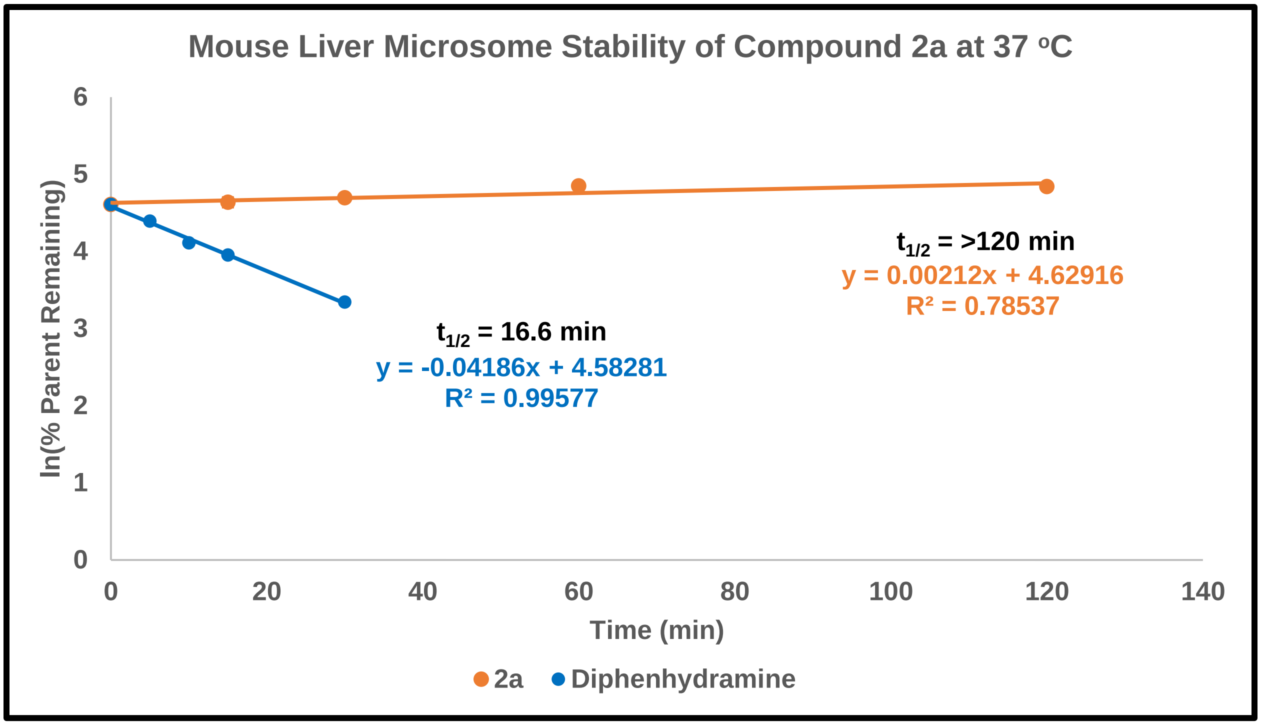


**Figure S26.** *In vitro* MLM stability of **2a** (5 points over 120 min) with diphenhydramine serving as a positive control. A half-life of greater than 120 min (>95% remaining) was calculated for **2a**.

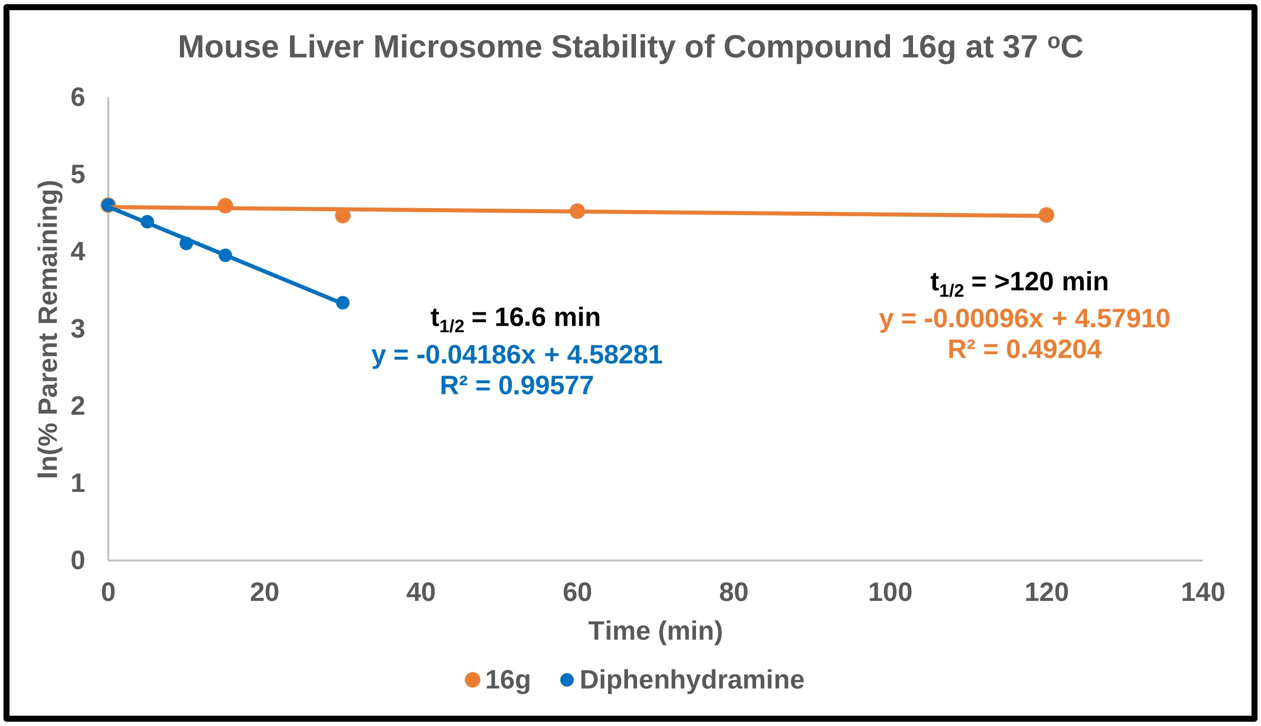


**Figure S27.** *In vitro* MLM stability of **16g** (5 points over 120 min) with diphenhydramine serving as a positive control. A half-life of greater than 120 min (88% remaining) was calculated for **16g**.

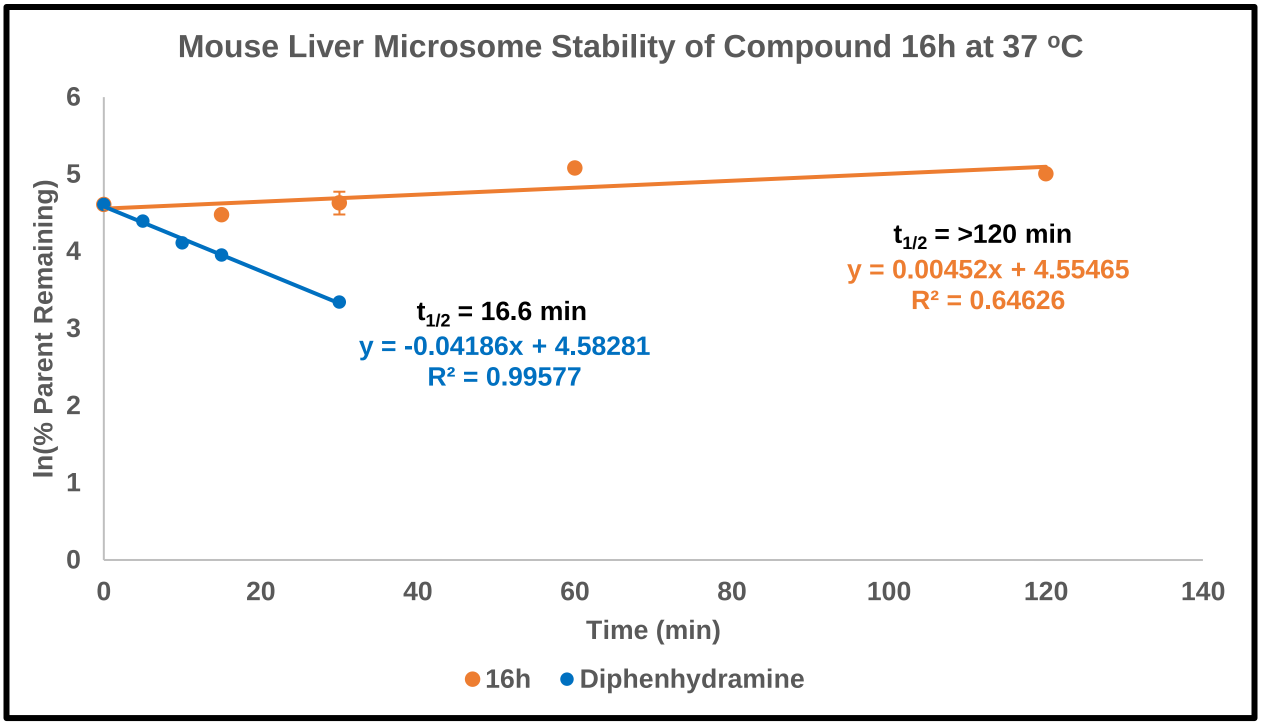


**Figure S28.** *In vitro* MLM stability of **16h** (5 points over 120 min) with diphenhydramine serving as a positive control. A half-life of greater than 120 min (>95% remaining) was calculated for **16h**.

Plasma Stability

*Plasma Stability Assay Setup Example*

**Test Compound:** 994 µL Human or Mouse Plasma + 6.0 µL TC (500 µM, Vial A).

994 µL Human or Mouse Plasma + 6.0 µL TC (500 µM, Vial B).

**Positive Control:** 994 µL Human or Mouse Plasma + 6.0 µL Procaine (500 µM).

**Negative Control:** 142 µL DPBS (1X, pH 7.1-7.3) + 0.9 µL TC (500 µM).

**Quenching Mixture:** 150 µL MeOH with ISTD (2 µM d5-7-ethoxycoumarin).

**Final Volume After Quenching:** 250 µL (100 µL from reaction mixture and 150 µL quencher solution; ISTD final concentration of 1.2 µM).

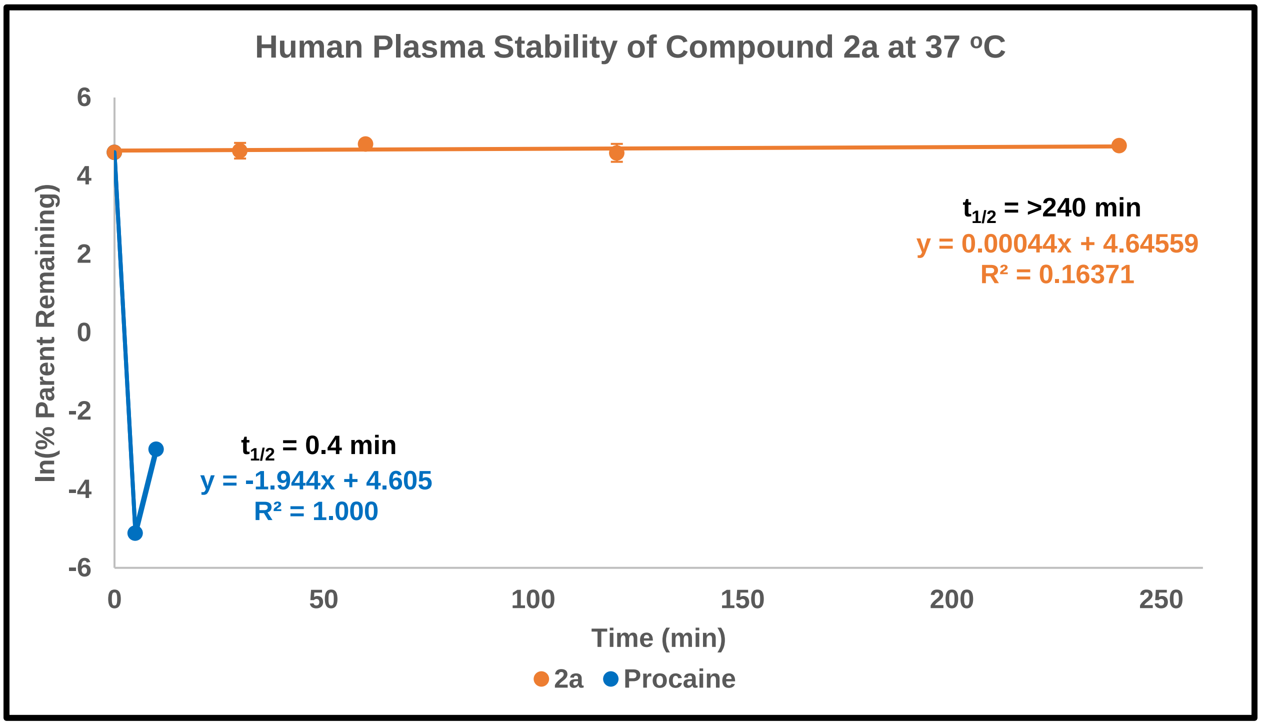


**Figure S29.** *In vitro* human plasma stability of **2a** (5 points over 240 min) with procaine serving as positive control. A half-life of greater than 240 min (>95% remaining) was calculated for **2a**.

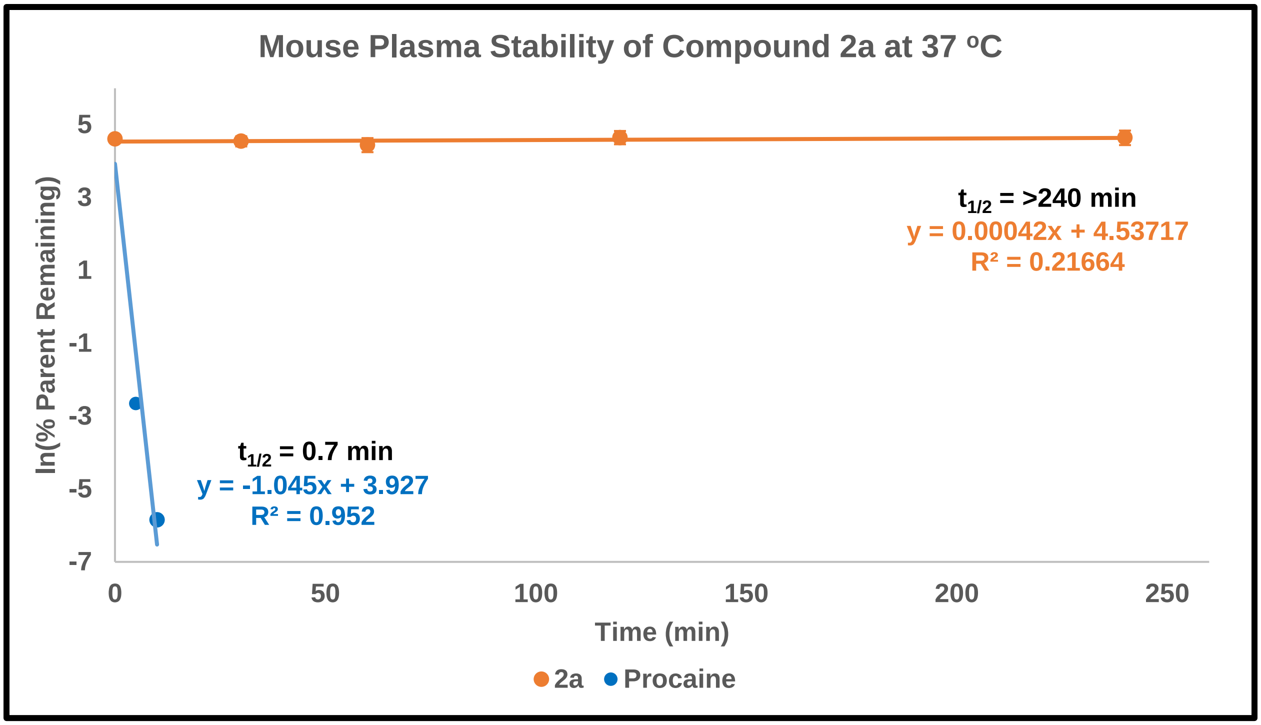


**Figure S30.** *In vitro* mouse plasma stability of **2a** (5 points over 240 min) with procaine serving as positive control. A half-life of greater than 240 min (>95% remaining) was calculated for **2a**.

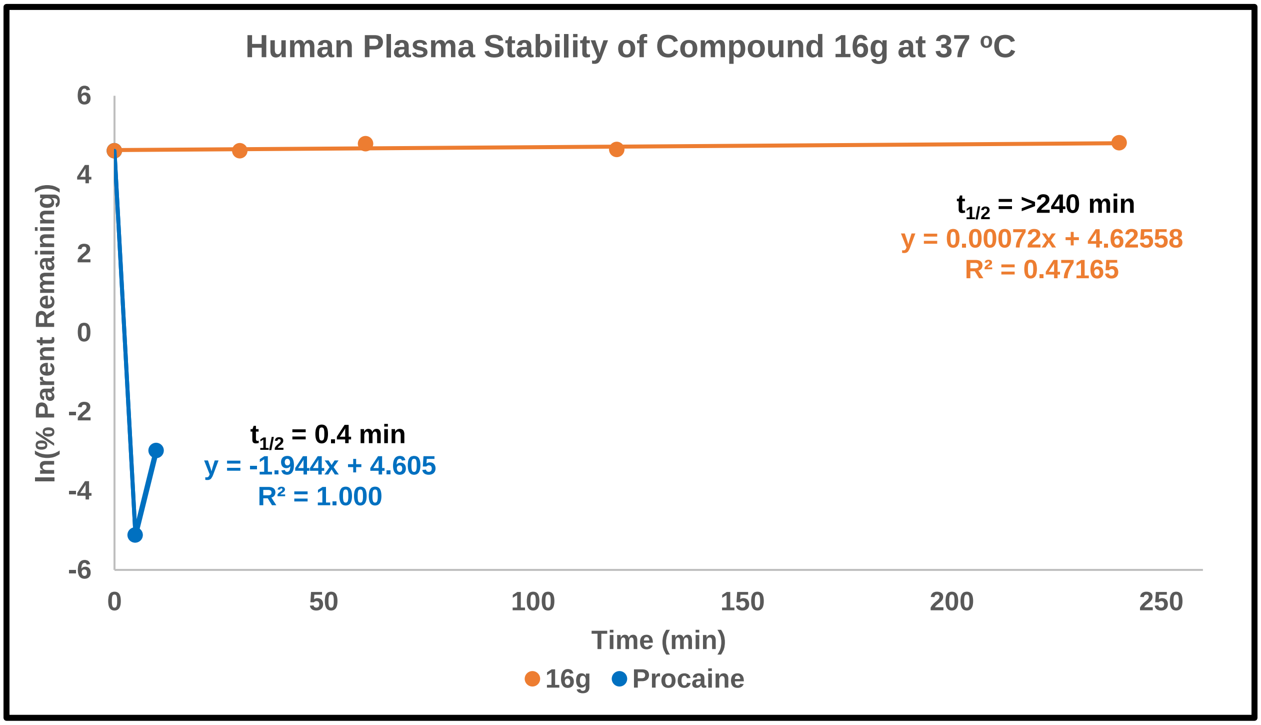


**Figure S31.** *In vitro* human plasma stability of **16g** (5 points over 240 min) with procaine serving as positive control. A half-life of greater than 240 min (>95% remaining) was calculated for **16g**.

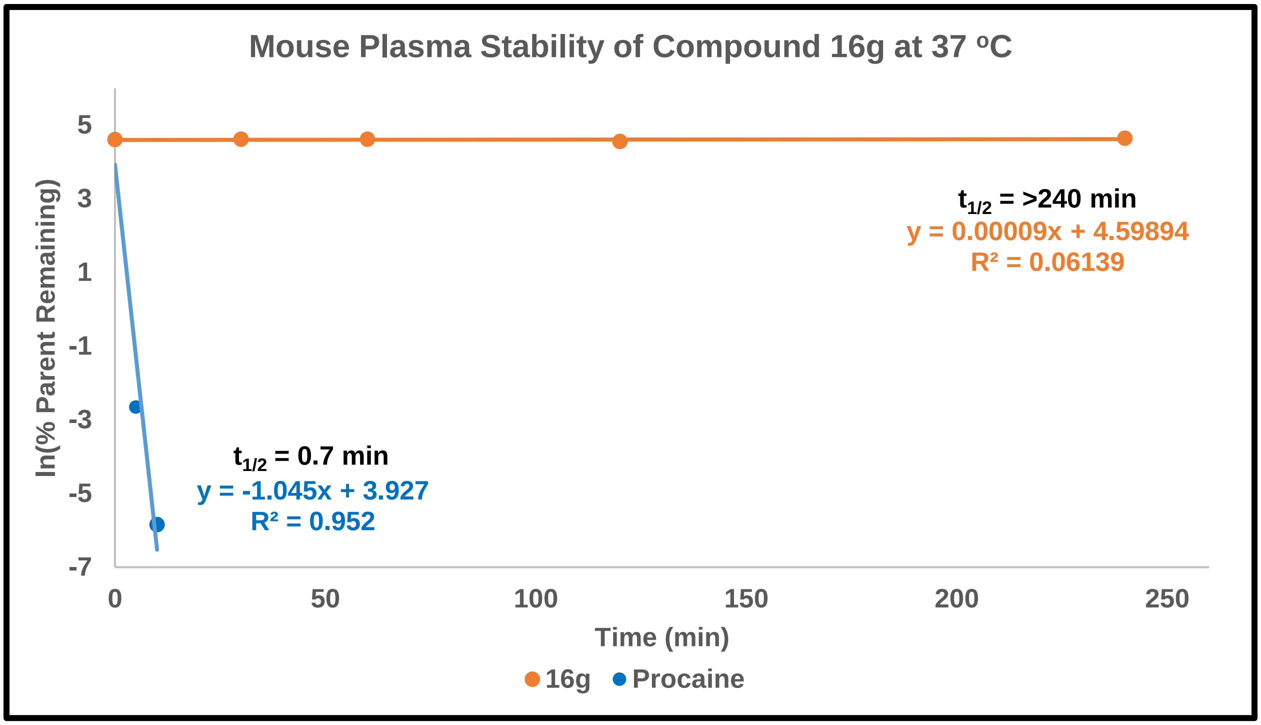


**Figure S32.** *In vitro* mouse plasma stability of **16g** (5 points over 240 min) with procaine serving as positive control. A half-life of greater than 240 min (>95% remaining) was calculated for **16g**.

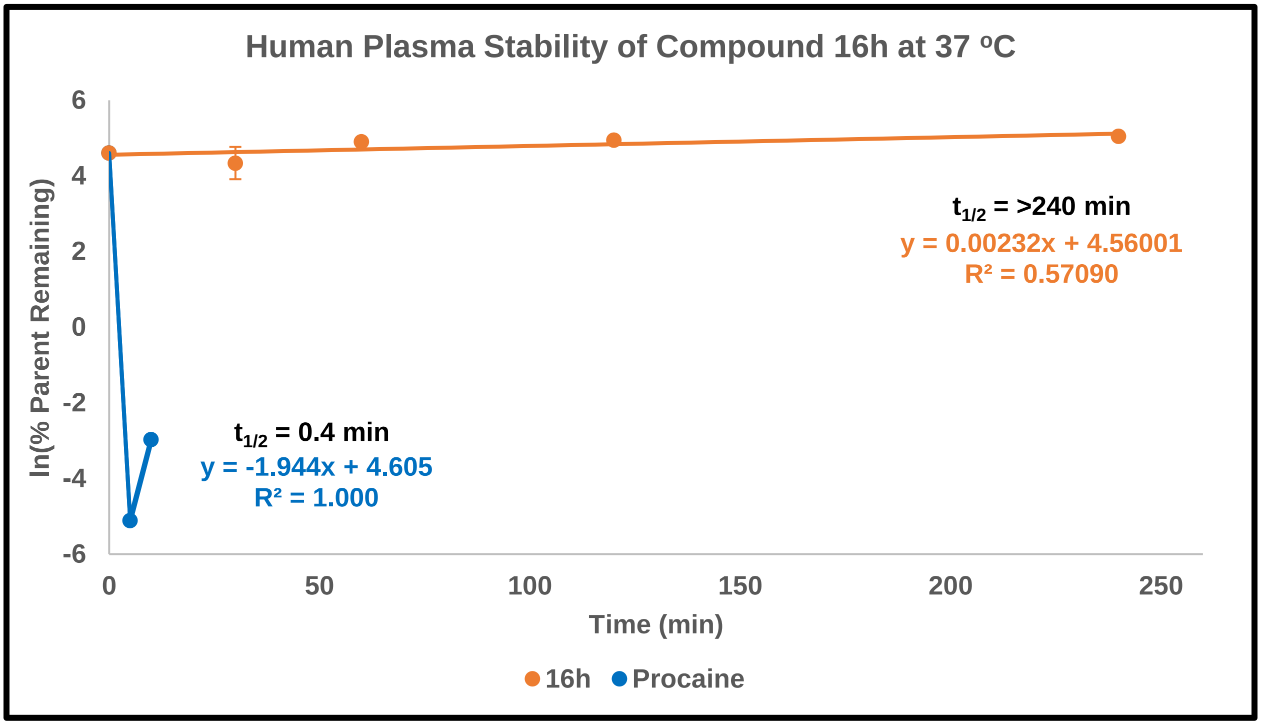


**Figure S33.** *In vitro* human plasma stability of **16h** (5 points over 240 min) with procaine serving as positive control. A half-life of greater than 240 min (>95% remaining) was calculated for **16h**.

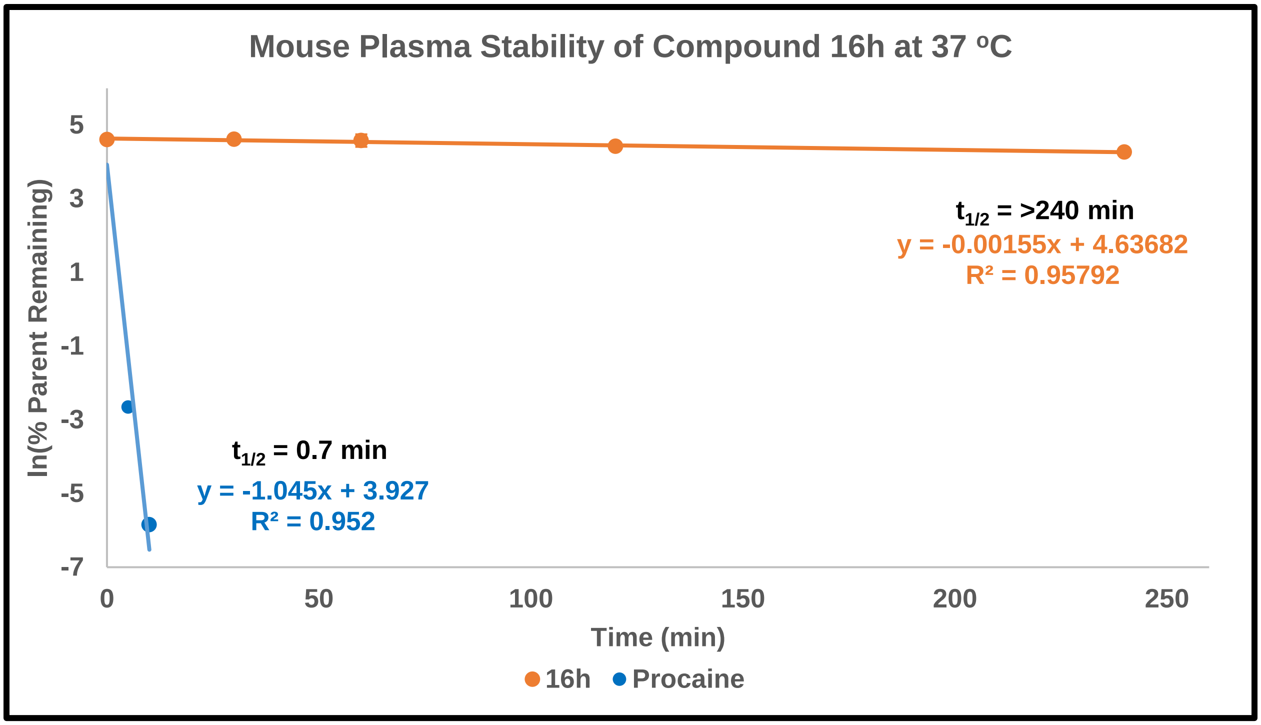


**Figure S34.** *In vitro* mouse plasma stability of **16h** (5 points over 240 min) with procaine serving as a positive control. A half-life of greater than 240 min (71% remaining) was calculated for **16h**.

*HepaRG™ Cellular Uptake and Stability*

Cellular Uptake

**Table S2.** Individual HepaRG™ cellular concentration-time data of TXL (20 µM).

| **Data Point** | **[TXL] in HepaRG™ Cells (ng/mL)** | | | | | | |
|---|---|---|---|---|---|---|---|
| | **Time (h)** | | | | | | |
| | **0** | **0.5** | **1** | **2** | **3** | **5** | **7** |
| **1** | 0 | 257.89 | 310.05 | 376.99 | 396.08 | 466.21 | 536.35 |
| **2** | 0 | 259.77 | 312.87 | 380.33 | 398.10 | 470.32 | 536.59 |
| **Mean** | 0 | 258.83 | 311.46 | 378.66 | 397.09 | 468.27 | 536.47 |
| **St. Dev.** | 0 | 1.33 | 1.99 | 2.36 | 1.42 | 2.90 | 0.17 |

**Table S3.** Individual HepaRG™ cellular concentration-time data of compound **1** (20 µM).

| **Data Point** | **[Compound 1] in HepaRG™ Cells (ng/mL)** | | | | | | |
|---|---|---|---|---|---|---|---|
| | **Time (h)** | | | | | | |
| | **0** | **0.5** | **1** | **2** | **3** | **5** | **7** |
| **1** | 0 | 141.49 | 181.19 | 319.51 | 338.16 | 321.49 | 415.80 |
| **2** | 0 | 141.36 | 179.49 | 320.15 | 336.70 | 321.63 | 418.46 |
| **Mean** | 0 | 141.43 | 180.34 | 319.83 | 337.43 | 321.56 | 417.13 |
| **St. Dev.** | 0 | 0.09 | 1.20 | 0.45 | 1.04 | 0.10 | 1.89 |

**Table S4.** Individual HepaRG™ cellular concentration-time data of compound **2a** (20 µM).

| **Data Point** | **[Compound 2a] in HepaRG™ Cells (ng/mL)** | | | | | | |
|---|---|---|---|---|---|---|---|
| | **Time (h)** | | | | | | |
| | **0** | **0.5** | **1** | **2** | **3** | **5** | **7** |
| **1** | 0 | 467.11 | 529.11 | 679.57 | 827.86 | 1010.68 | 995.34 |
| **2** | 0 | 467.44 | 531.23 | 674.30 | 828.08 | 998.59 | 993.37 |
| **Mean** | 0 | 467.27 | 530.17 | 676.9 | 827.97 | 1004.64 | 994.36 |
| **St. Dev.** | 0 | 0.23 | 1.50 | 3.73 | 0.16 | 8.55 | 1.39 |

**Table S5.** Individual HepaRG$^{TM}$ cellular concentration-time data of compound **15e** (20 µM).

| Data Point | [Compound 15e] in HepaRG$^{TM}$ Cells (ng/mL) | | | | | | |
|---|---|---|---|---|---|---|---|
| | Time (h) | | | | | | |
| | 0 | 0.5 | 1 | 2 | 3 | 4 | 6 |
| **1** | 0 | 182.51 | 210.27 | 233.72 | 245.85 | 290.14 | 343.99 |
| **2** | 0 | 157.01 | 213.40 | 232.33 | 238.11 | 273.64 | 299.92 |
| **Mean** | 0 | 169.76 | 211.84 | 233.02 | 241.98 | 281.89 | 321.96 |
| **St. Dev.** | 0 | 18.04 | 2.21 | 0.98 | 5.48 | 11.66 | 31.17 |

**Table S6.** Individual HepaRG$^{TM}$ cellular concentration-time data of compound **16e** (20 µM).

| Data Point | [Compound 16e] in HepaRG$^{TM}$ Cells (ng/mL) | | | | | | |
|---|---|---|---|---|---|---|---|
| | Time (h) | | | | | | |
| | 0 | 0.5 | 1 | 2 | 3 | 4 | 6 |
| **1** | 0 | 634.81 | 1060.73 | 818.73 | 1144.99 | 938.29 | 1287.80 |
| **2** | 0 | 615.62 | 907.50 | 843.56 | 1055.08 | 1062.08 | 1105.74 |
| **Mean** | 0 | 625.22 | 984.12 | 831.14[a] | 1100.03 | 1000.18 | 1196.77 |
| **St. Dev.** | 0 | 13.57 | 108.35 | 17.55 | 63.57 | 87.53 | 128.73 |

[a]Value excluded from data analysis.

**Table S7.** Individual HepaRG$^{TM}$ concentration-time data of compound **16g** (20 µM).

| Data Point | [Compound 16g] in HepaRG$^{TM}$ Cells (ng/mL) | | | | | | |
|---|---|---|---|---|---|---|---|
| | Time (h) | | | | | | |
| | 0 | 0.5 | 1 | 2 | 3 | 5 | 7 |
| **1** | 0 | 123.26 | 163.43 | 177.68 | 209.64 | 203.13 | 228.45 |
| **2** | 0 | 124.81 | 162.07 | 178.67 | 209.97 | 204.84 | 229.71 |
| **Mean** | 0 | 124.04 | 162.75 | 178.18 | 209.81 | 203.98 | 229.08 |
| **St. Dev.** | 0 | 1.09 | 0.96 | 0.70 | 0.23 | 1.21 | 0.89 |

**Table S8.** Individual HepaRG™ concentration-time data of compound **16h** (20 µM).

| Data Point | [Compound 16h] in HepaRG™ Cells (ng/mL) | | | | | | |
|---|---|---|---|---|---|---|---|
| | Time (h) | | | | | | |
| | **0** | **0.5** | **1** | **2** | **3** | **5** | **7** |
| **1** | 0 | 894.25 | 907.54 | 1176.17 | 1411.33 | 1536.47 | 1606.97 |
| **2** | 0 | 898.07 | 915.52 | 1180.07 | 1413.40 | 1530.28 | 1625.36 |
| **Mean** | 0 | 896.16 | 911.53 | 1178.12 | 1412.37 | 1533.37 | 1616.17 |
| **St. Dev.** | 0 | 2.70 | 5.65 | 2.76 | 1.47 | 4.37 | 13.00 |

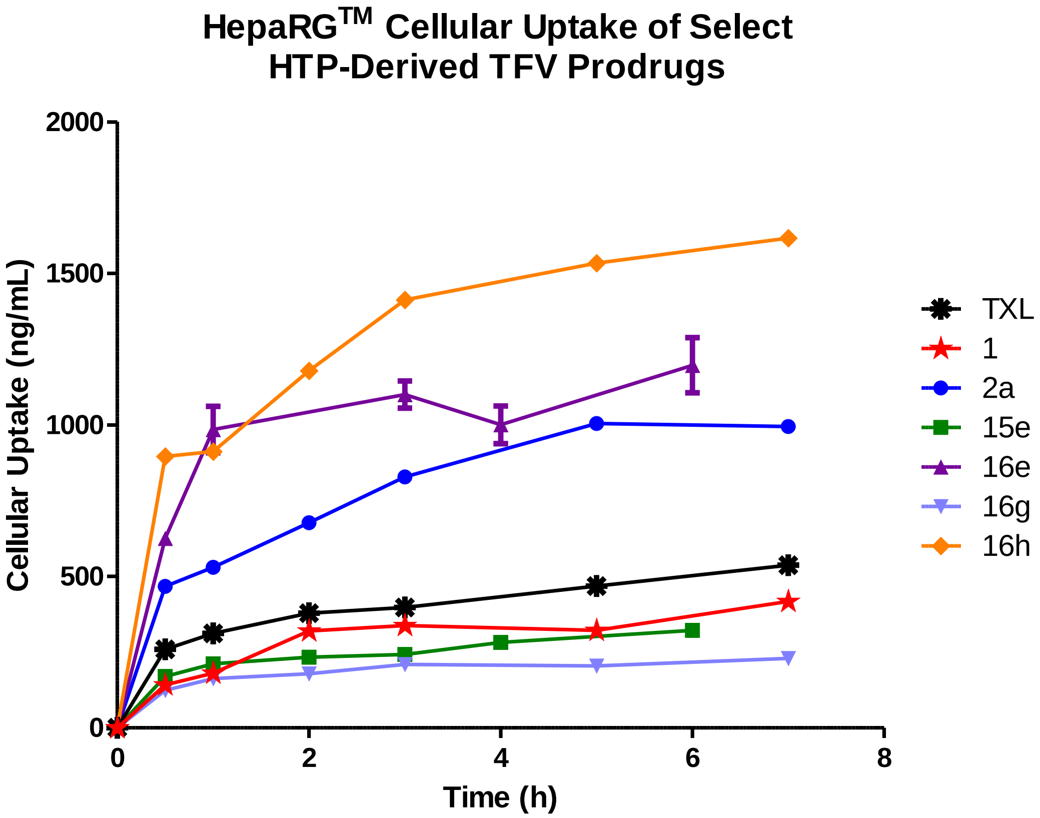


**Figure S35**. Summary of HepaRG™ cellular uptake results for HTS-derived prodrugs, alongside TXL and compound **1**.

Cellular Stability

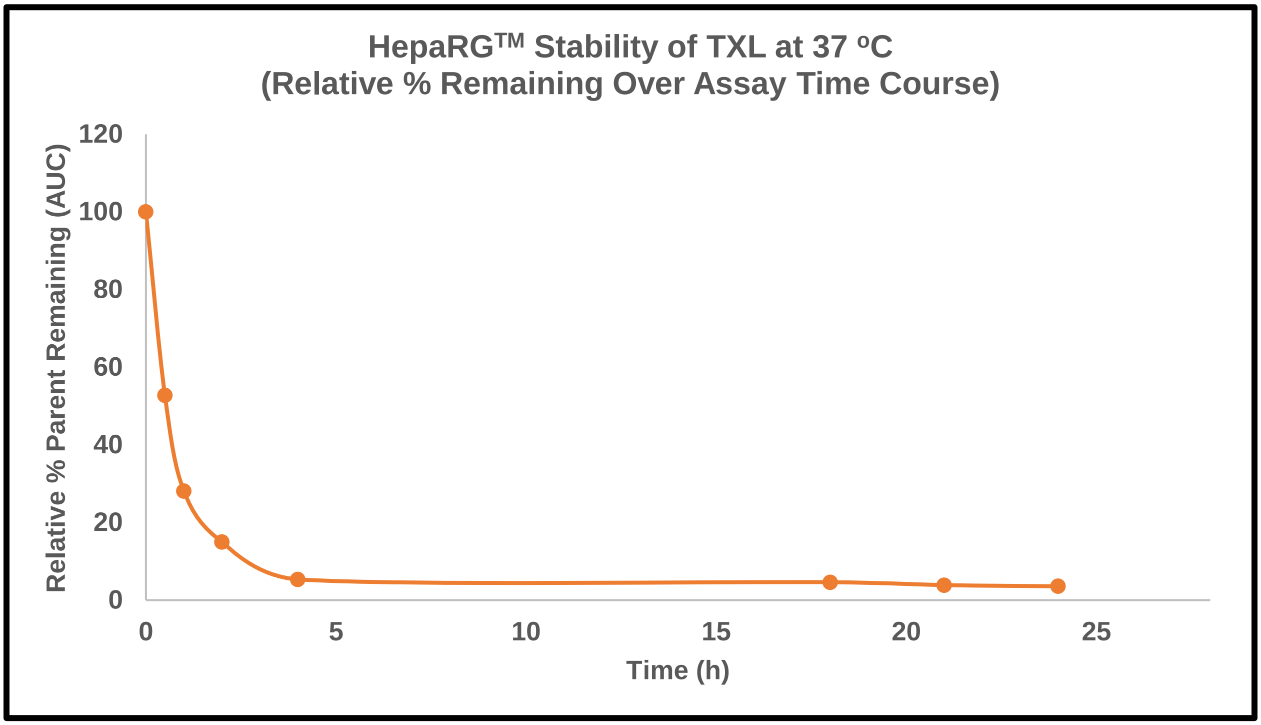


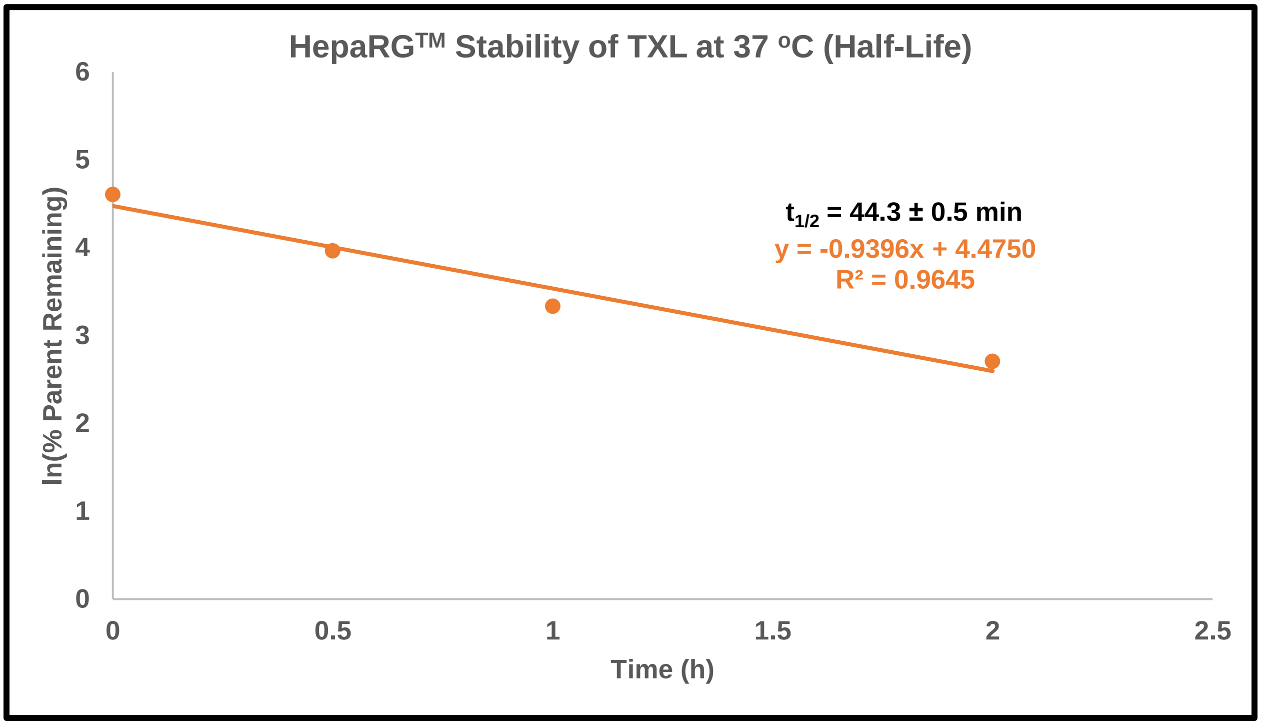


**Figure S36.** *In vitro* HepaRG™ cellular stability of TXL represented as relative % parent remaining (AUC) over assay time course (top). A half-life (4 points over 120 min) of 44.3 ± 0.5 min was calculated for TXL (bottom), resulting in an intrinsic clearance of 28.5 µL/min/million cells.

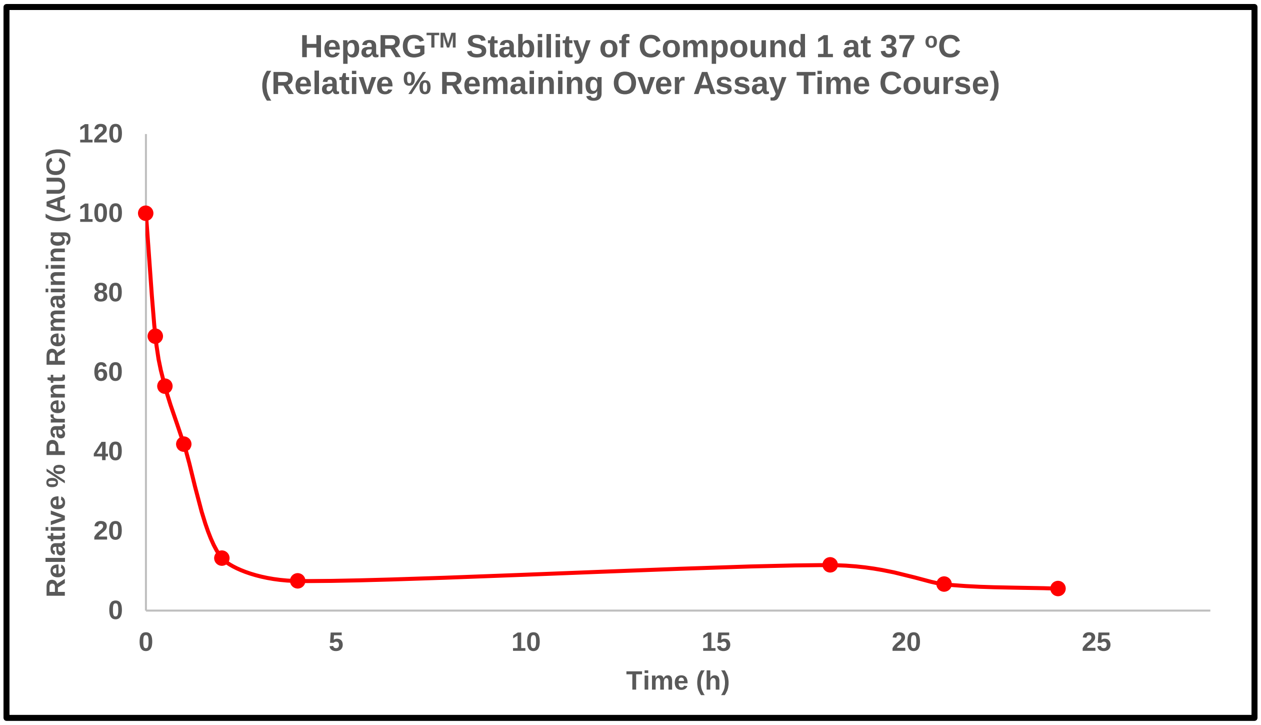


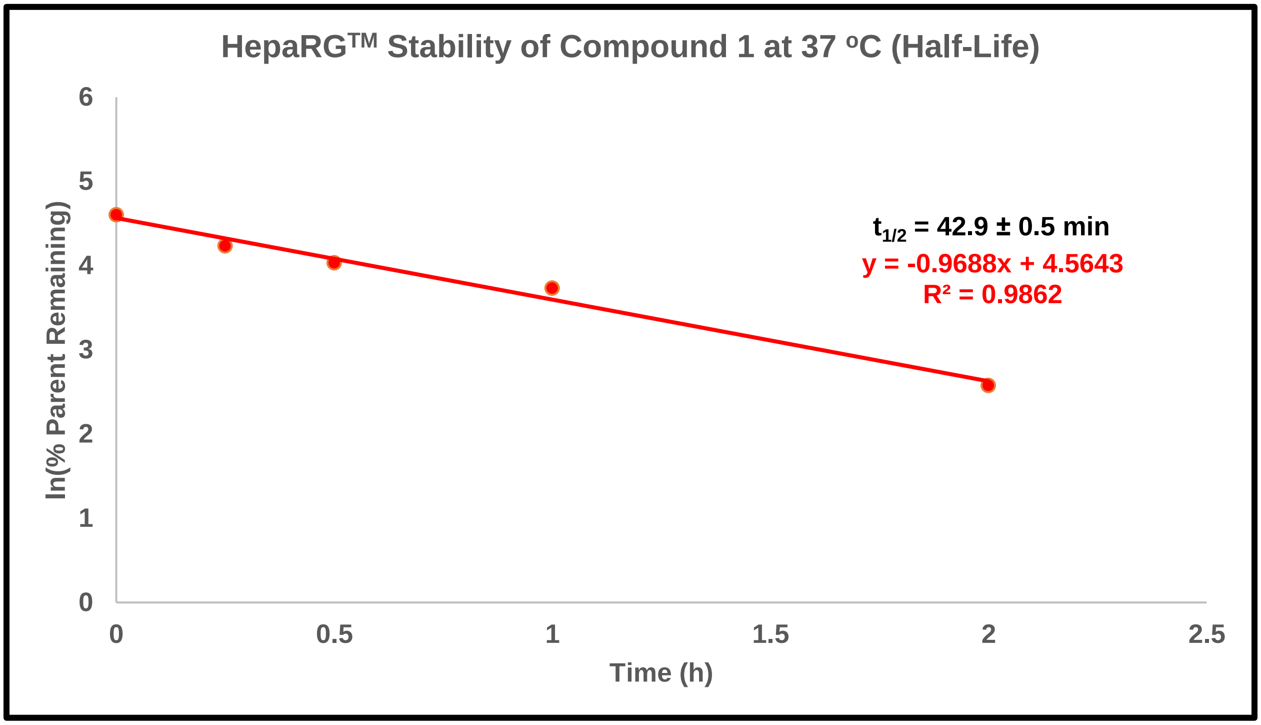


**Figure S37.** *In vitro* HepaRG™ cellular stability of compound **1** represented as relative % parent remaining (AUC) over assay time course (top). A half-life (5 points over 120 min) of 42.9 ± 0.5 min was calculated for **1** (bottom), resulting in an intrinsic clearance of 29.4 µL/min/million cells.

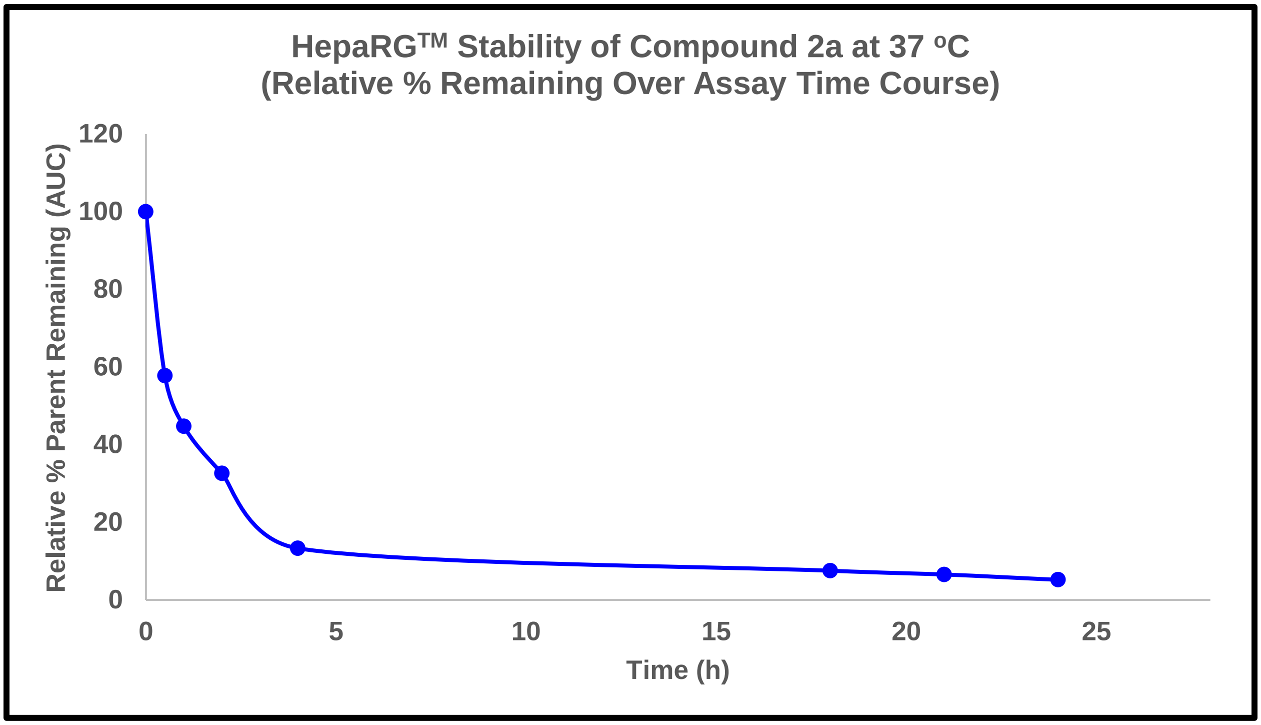


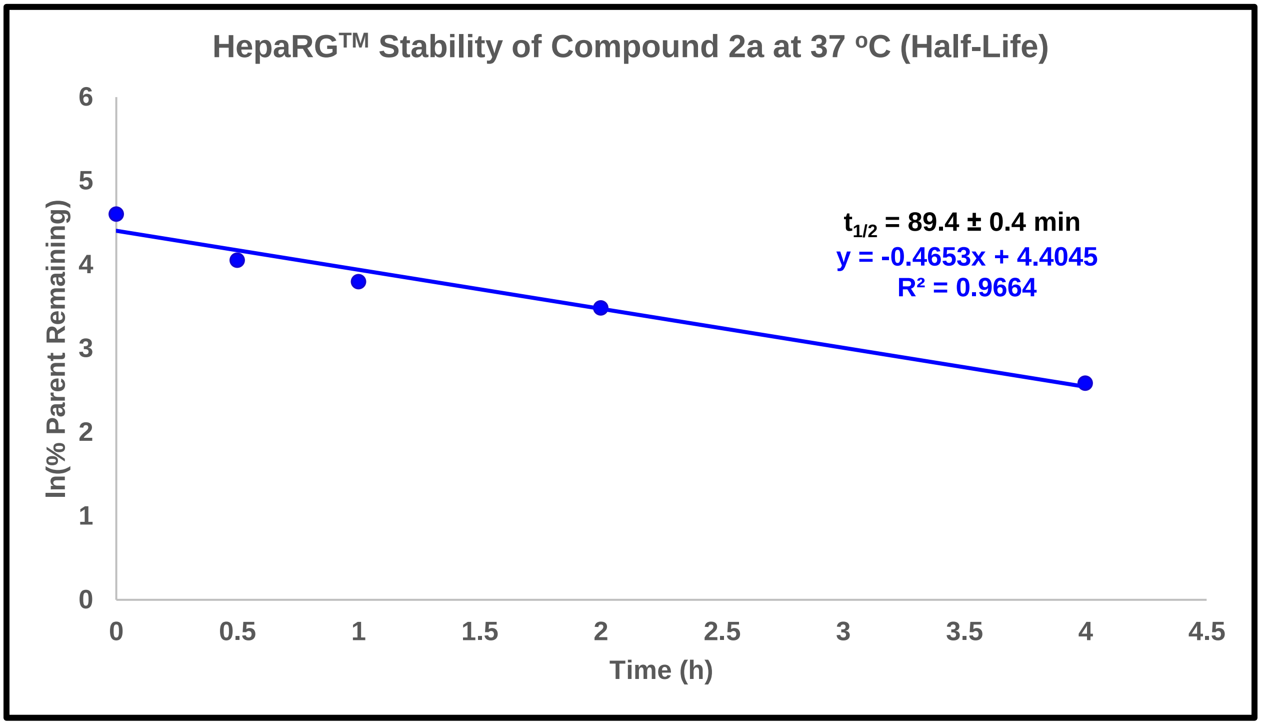


**Figure S38.** *In vitro* HepaRG™ cellular stability of compound **2a** represented as relative % parent remaining (AUC) over assay time course (top). A half-life (5 points over 240 min) of 89.4 ± 0.4 min was calculated for **2a** (bottom), resulting in an intrinsic clearance of 14.1 µL/min/million cells.

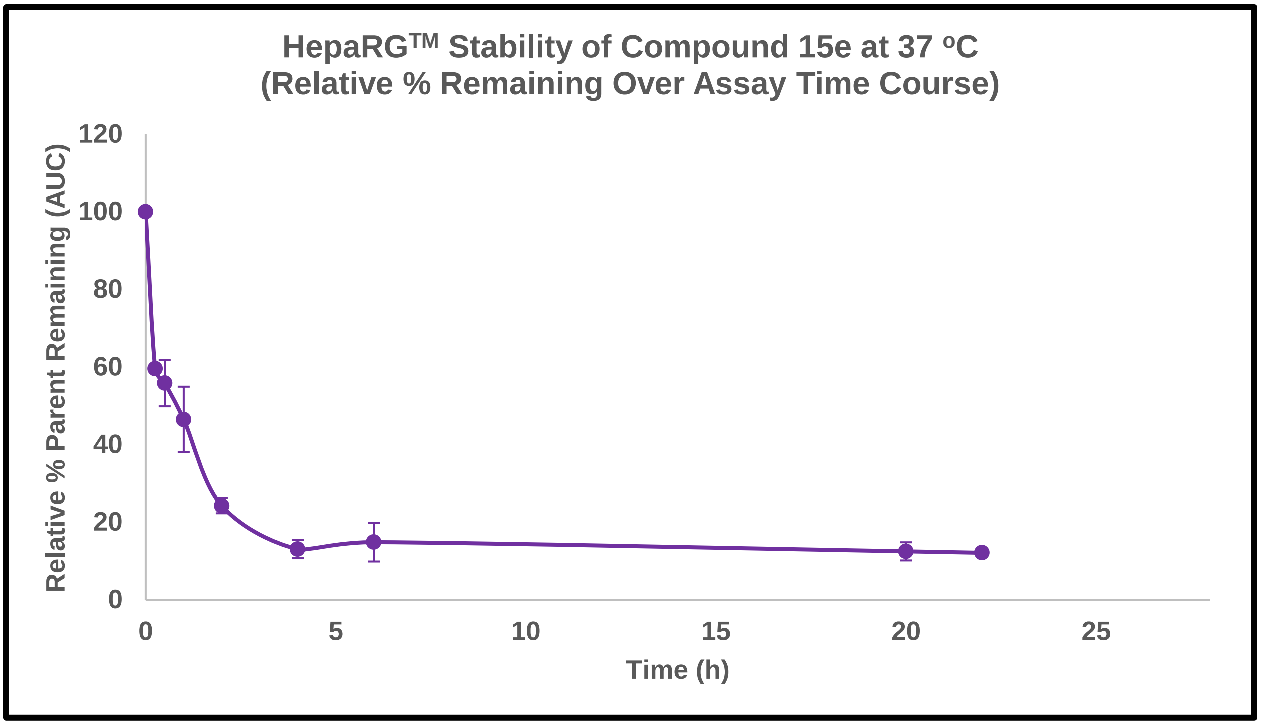


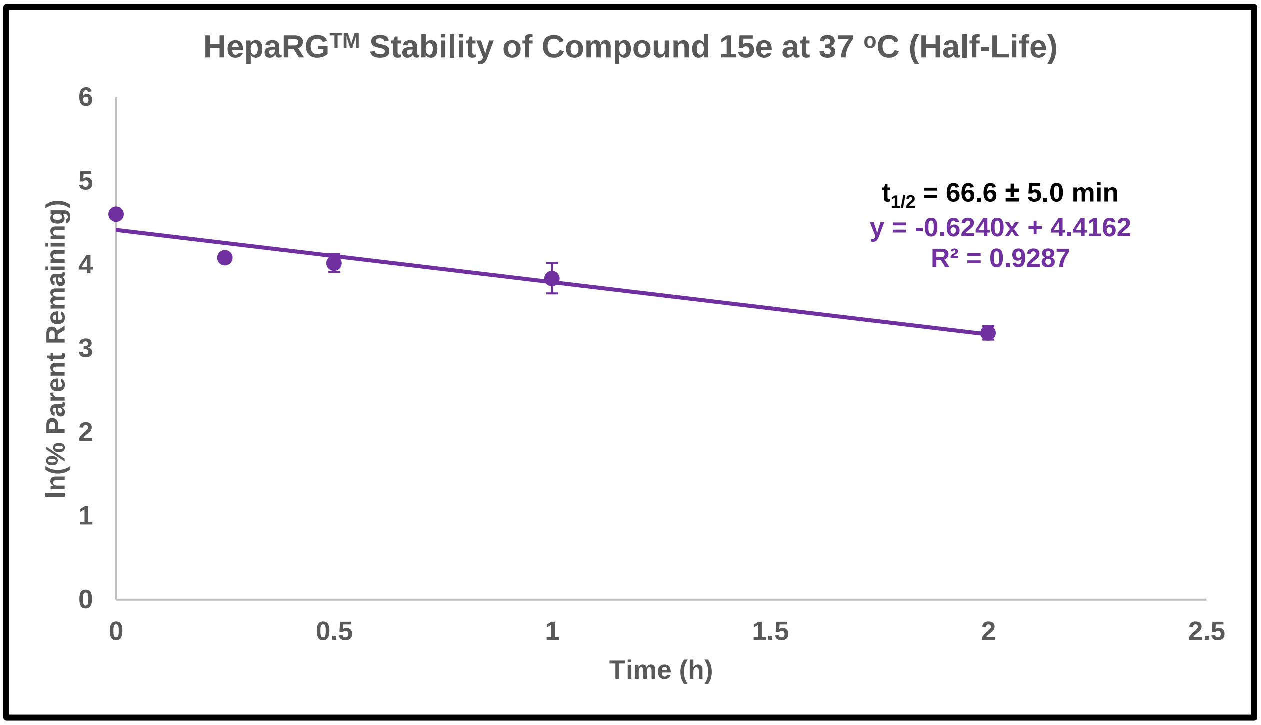


**Figure S39.** *In vitro* HepaRG$^{TM}$ cellular stability of compound **15e** represented as relative % parent remaining (AUC) over assay time course (top). A half-life (5 points over 120 min) of 66.6 ± 5.0 min was calculated for **15e** (bottom), resulting in an intrinsic clearance of 18.9 μL/min/million cells.

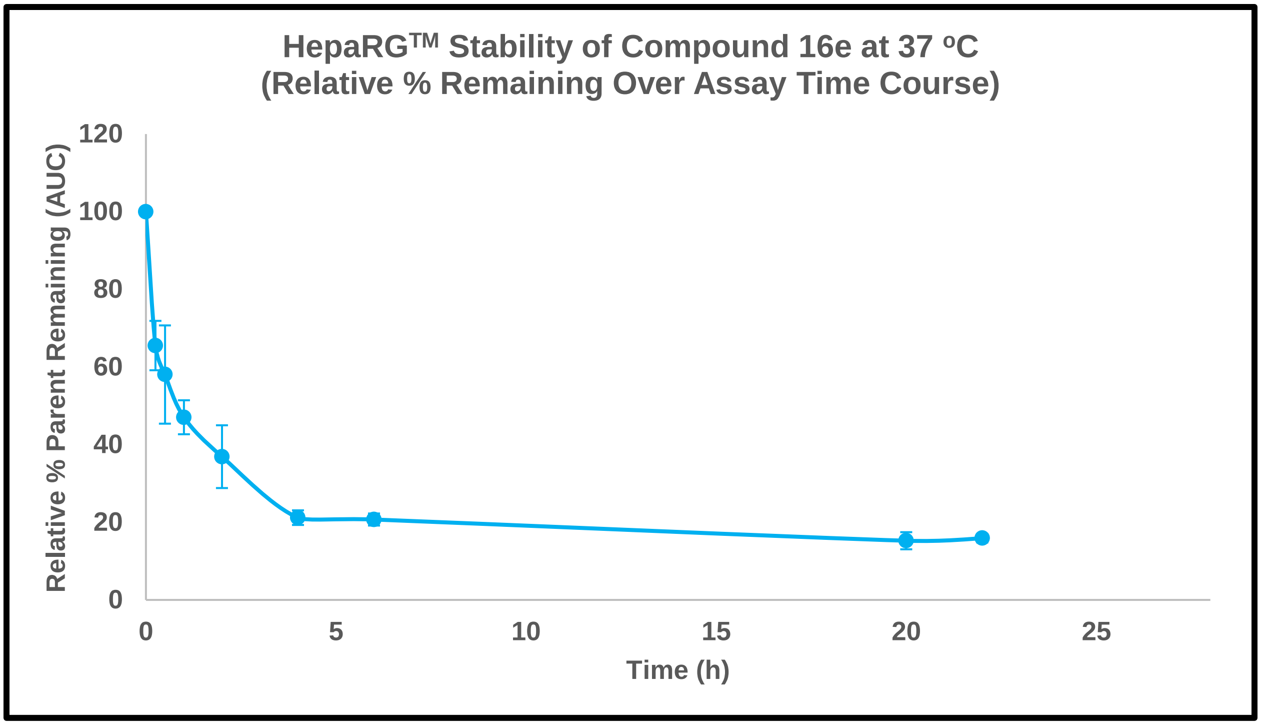


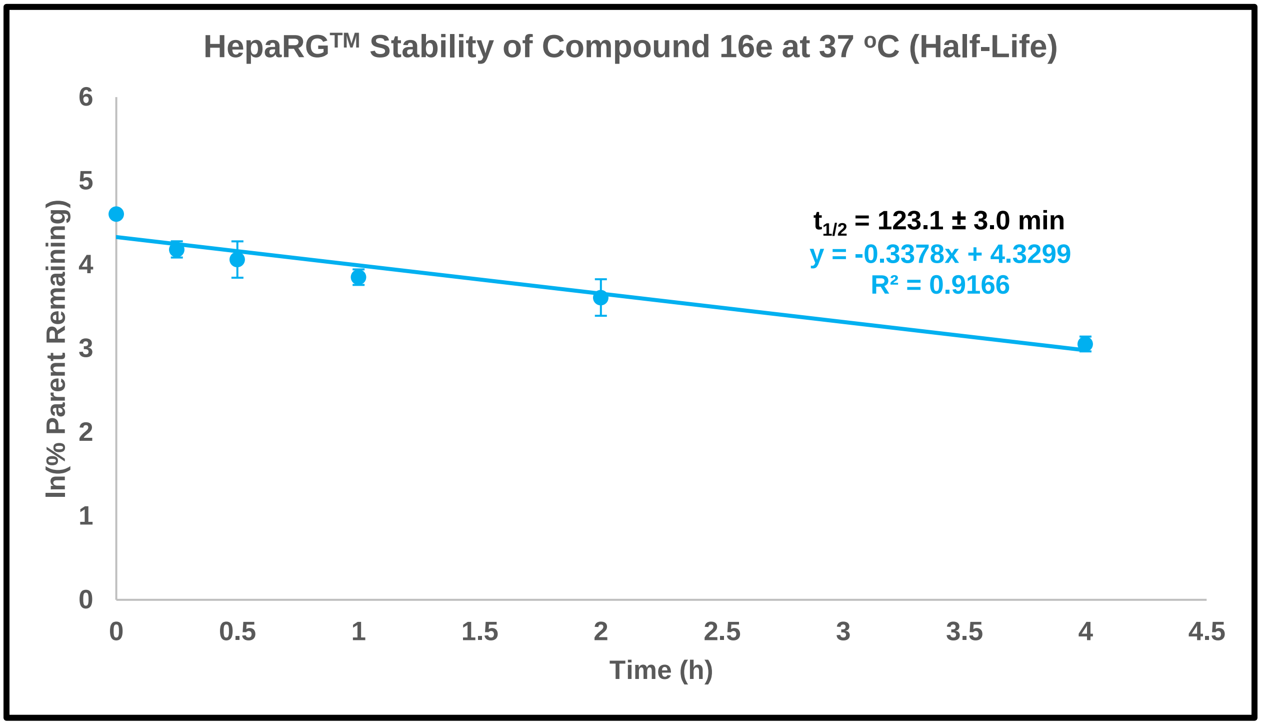


**Figure S40.** *In vitro* HepaRG$^{TM}$ cellular stability of compound **16e** represented as relative % parent remaining (AUC) over assay time course (top). A half-life (6 points over 240 min) of 123.1 ± 3.0 min was calculated for **16e** (bottom), resulting in an intrinsic clearance of 10.2 μL/min/million cells.

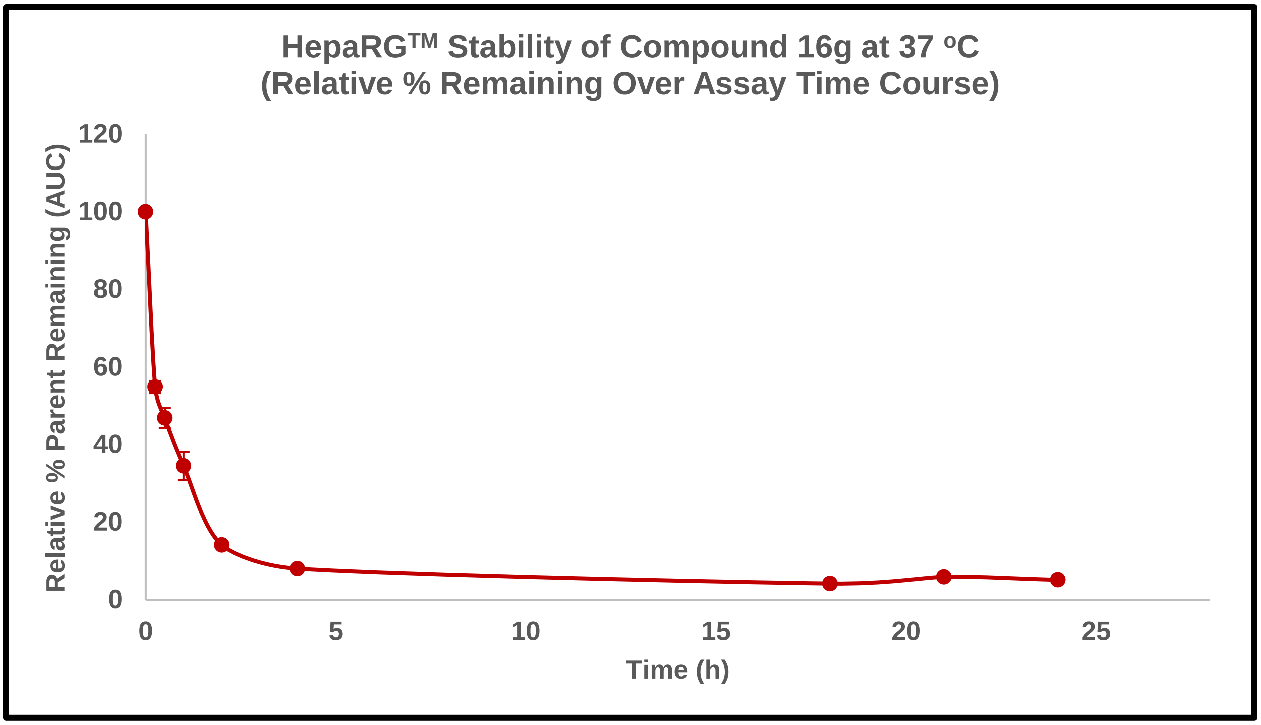


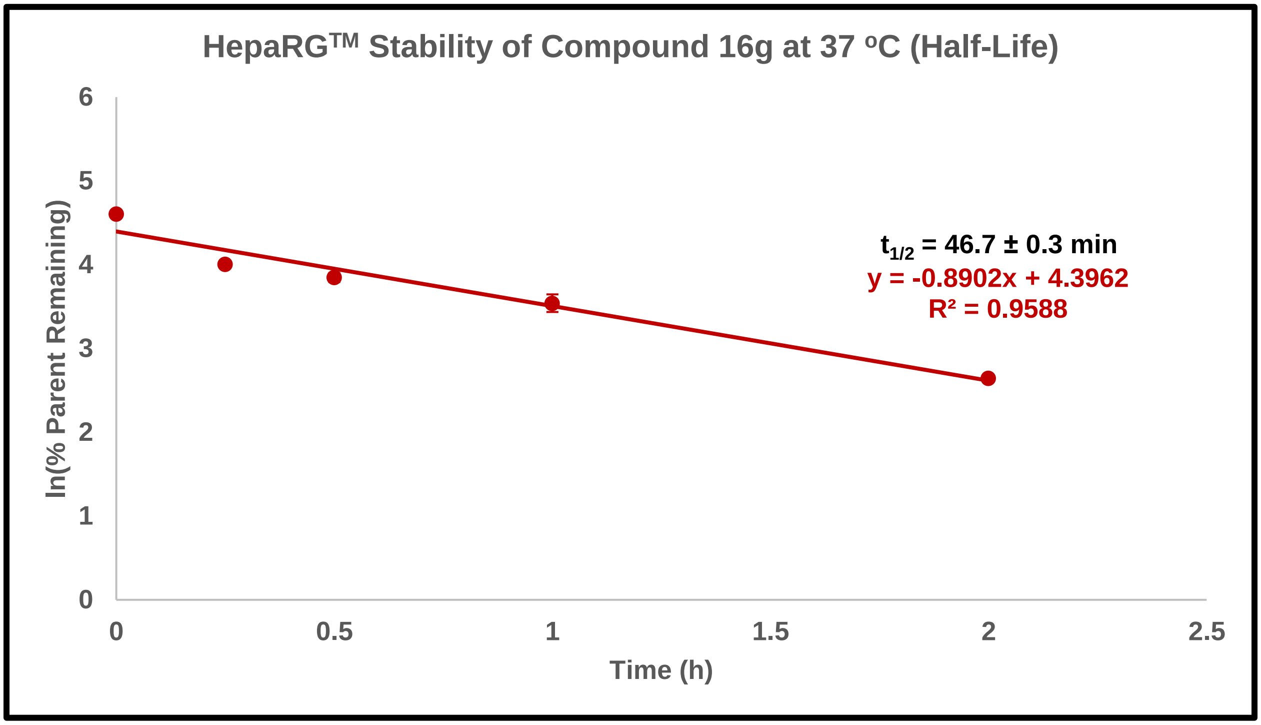


**Figure S41.** *In vitro* HepaRG$^{TM}$ cellular stability of compound **16g** represented as relative % parent remaining (AUC) over assay time course (top). A half-life (5 points over 120 min) of 46.7 ± 0.3 min was calculated for **16g** (bottom), resulting in an intrinsic clearance of 27.0 μL/min/million cells.

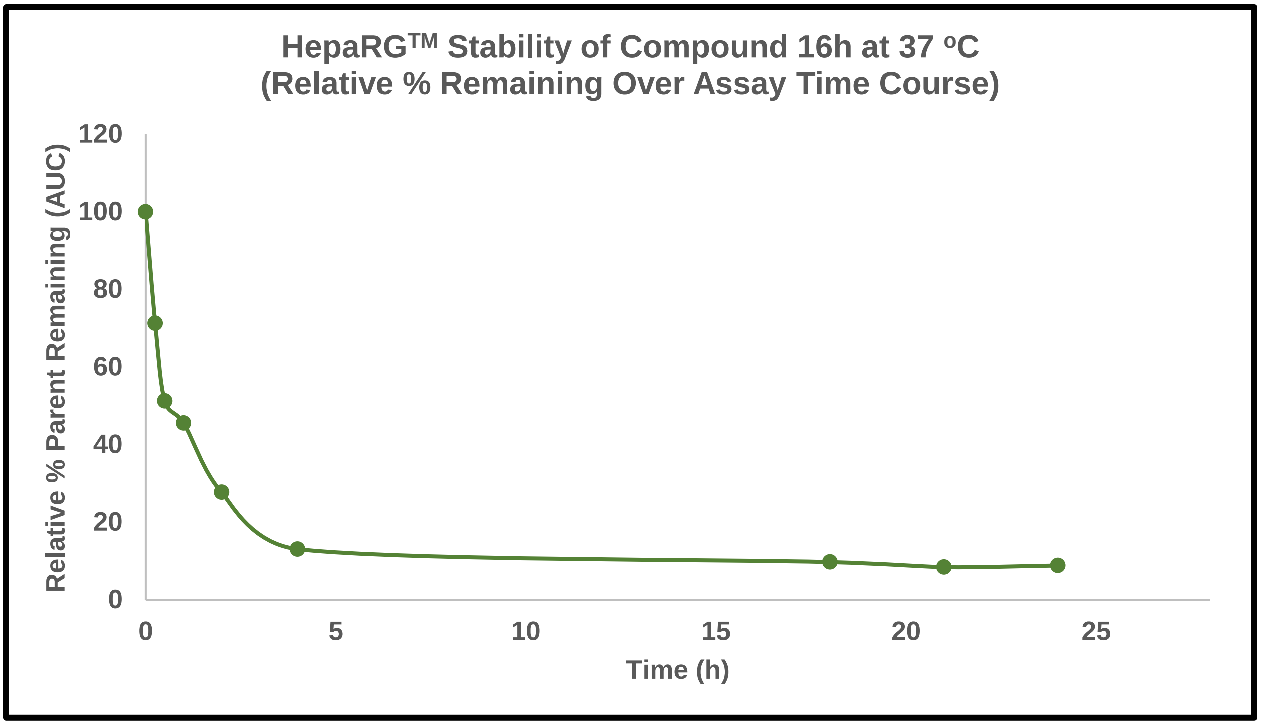


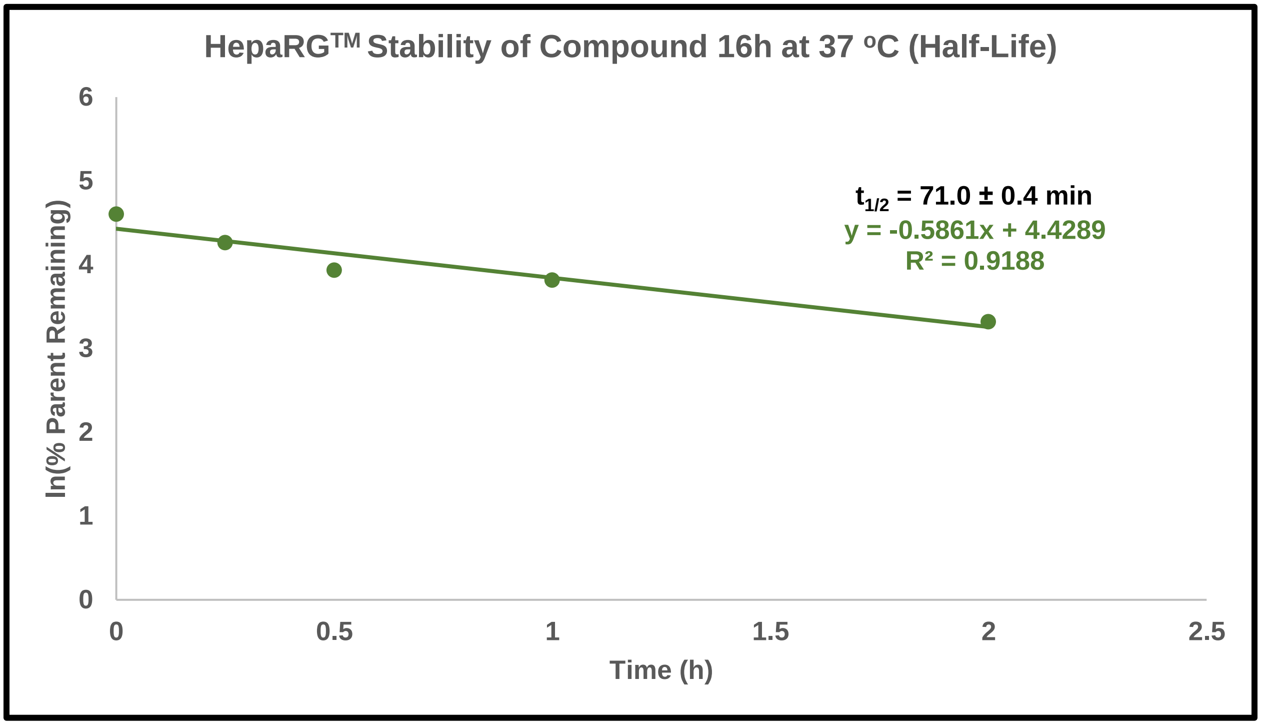


**Figure S42.** *In vitro* HepaRG$^{TM}$ cellular stability of compound **16h** represented as relative % parent remaining (AUC) over assay time course (top). A half-life (5 points over 120 min) of 71.0 ± 0.4 min was calculated for **16h** (bottom), resulting in an intrinsic clearance of 17.8 μL/min/million cells.

## *In Vivo* Mouse Pharmacokinetics Study (Conducted by Sai Life Sciences)

*Plasma Pharmacokinetics*

**Table S9.** Individual plasma concentration-time data of **2a** in male C57BL/6 mice following a single intravenous dose (3 mg/kg).

| **Data Point** | **[Compound 2a] in Plasma (ng/mL)** | | | | | | | |
|---|---|---|---|---|---|---|---|---|
| | **Time (h)** | | | | | | | |
| | **0.1** | **0.25** | **0.5** | **1** | **2** | **4** | **6** | **8** |
| **1** | 3159.09 | 334.77 | 155.38 | 50.40 | 11.19 | 3.04 | 1.43 | 1.36 |
| **2** | 1367.53 | 215.44 | 124.02 | 27.33 | 6.25 | 6.27 | 1.11 | 1.05 |
| **3** | 1657.15 | 410.14 | 121.70 | 93.53 | 8.59 | 1.94 | 7.69 | BLQ |
| **Mean** | 2061.26 | 320.12 | 133.70 | 57.09 | 8.68 | 3.75 | 3.41 | 1.21[a] |
| **St. Dev.** | 961.72 | 98.17 | 18.81 | 33.60 | 2.47 | 2.25 | 3.71 | 0.22 |

LLOQ = 0.58 ng/mL; BLQ = Below limit of quantitation; [a]Average of two values reported and considered for data analysis.

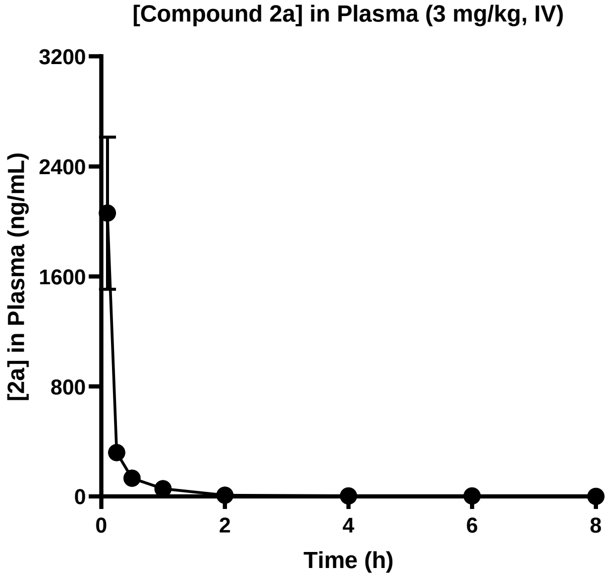


**Figure S43.** Mouse plasma pharmacokinetic profile for compound **2a**. Male C57BL/6 mice (n = 3 per time point) were administered a single intravenous dose (3 mg/kg) of TFV prodrug using 10% PEG-300, 10% Solutol HS-15, and 80% saline as a vehicle. Plasma levels of **2a** was quantified using LC-MS/MS. Data represent the mean concentration at each time point ± SEM. The figures are generated with GraphPad Prism v9.

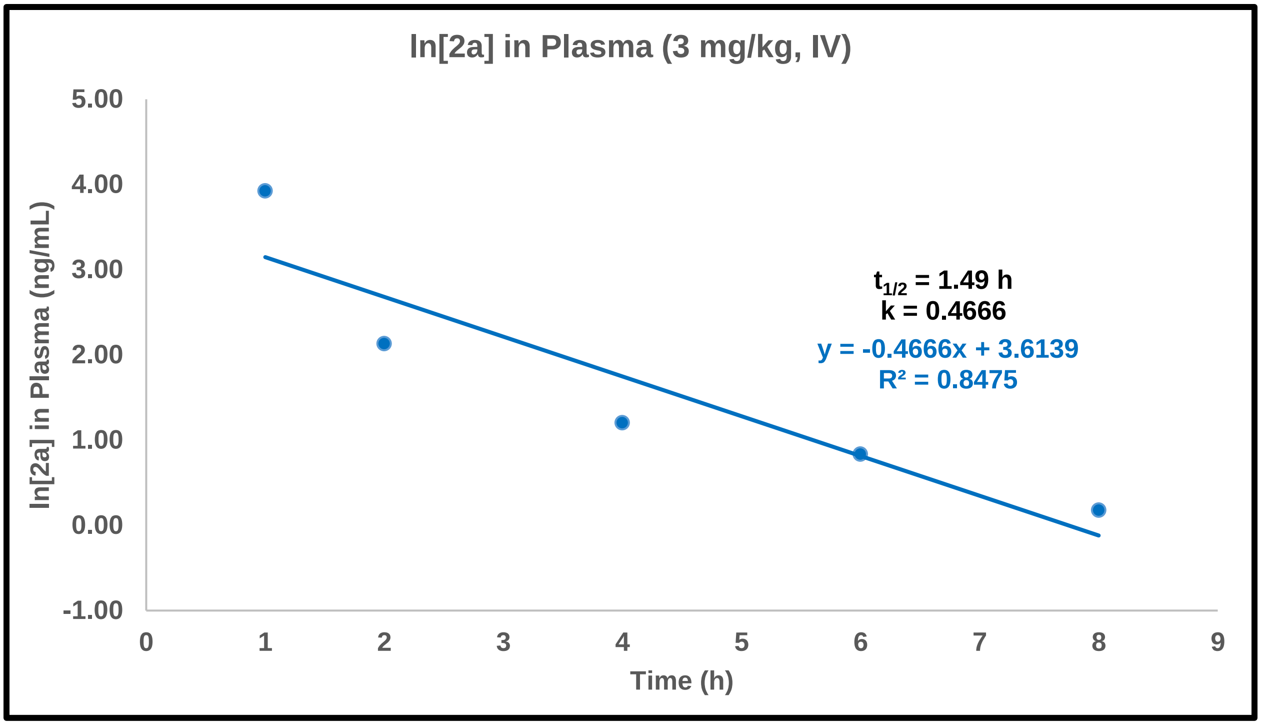


**Figure S44.** Plot of ln[**2a**] in mouse plasma (ng/mL) versus time after intravenous administration (3 mg/kg), which was used to calculate $t_{1/2}$.

**Table S10.** Individual plasma concentration-time data of **2a** in male C57BL/6 mice following a single oral dose (10 mg/kg).

| **Data Point** | **[Compound 2a] in Plasma (ng/mL)** | | | | | | | |
|---|---|---|---|---|---|---|---|---|
| | **Time (h)** | | | | | | | |
| | **0.25** | **0.5** | **1** | **2** | **4** | **8** | **12** | **24** |
| **1** | 40.47 | 58.69 | 70.75 | 48.92 | 8.68 | 9.33 | 9.73 | BLQ |
| **2** | 26.12 | 59.47 | 102.62 | 50.40 | 8.24 | 5.63 | 6.02 | BLQ |
| **3** | 22.08 | 37.20 | 46.60 | 44.61 | 8.10 | 6.33 | 7.06 | BLQ |
| **Mean** | 29.56 | 51.79 | 73.32 | 47.98 | 8.34 | 7.10 | 7.60 | NA |
| **St. Dev.** | 9.66 | 12.64 | 28.10 | 3.01 | 0.30 | 1.97 | 1.91 | NA |

LLOQ = 0.58 ng/mL; BLQ = Below limit of quantitation; NA = Not applicable.

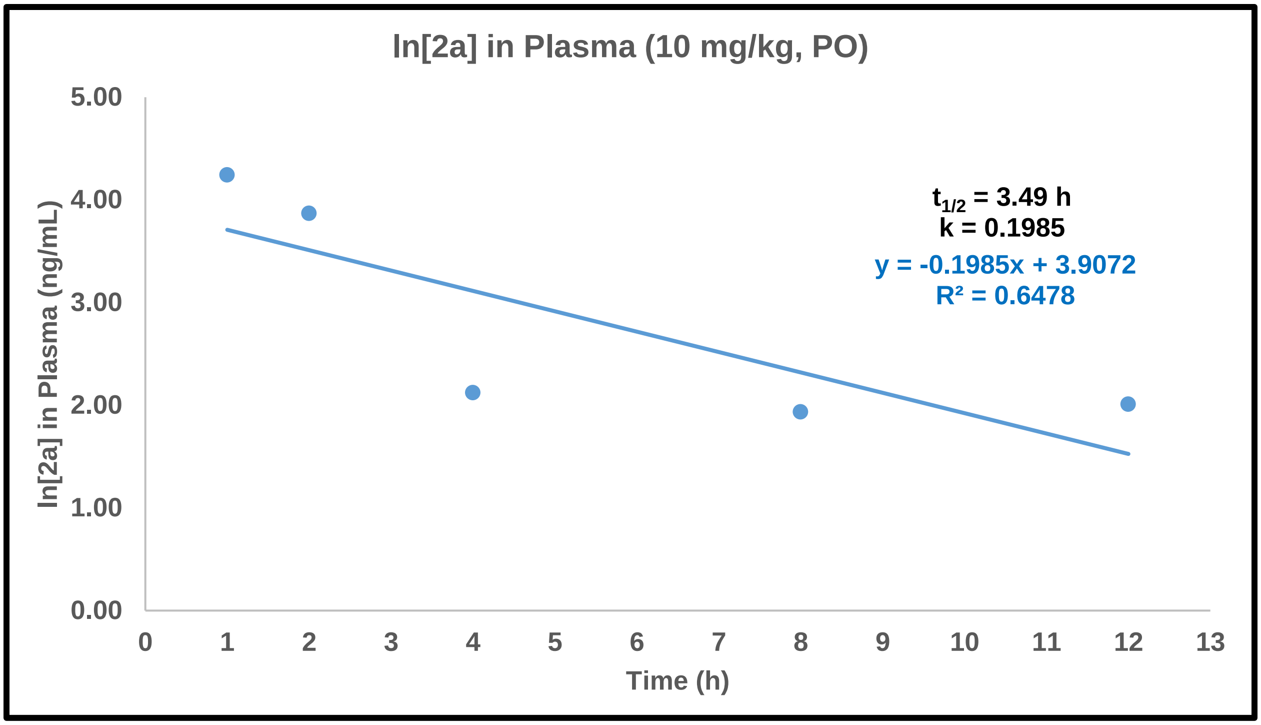


**Figure S45.** Plot of ln[**2a**] in mouse plasma (ng/mL) versus time after oral administration (10 mg/kg), which was used to calculate $t_{1/2}$.

**Table S11.** Individual plasma concentration-time data of TFV in male C57BL/6 mice following a single oral dose of compound **2a** (10 mg/kg).

| **Data Point** | **[TFV] in Plasma (ng/mL)** | | | | | | | |
|---|---|---|---|---|---|---|---|---|
| | **Time (h)** | | | | | | | |
| | **0.25** | **0.5** | **1** | **2** | **4** | **8** | **12** | **24** |
| **1** | BLQ | 5.28 | 12.72 | 23.61 | 38.64 | 23.70 | 35.20 | 9.34 |
| **2** | BLQ | BLQ | 11.39 | 34.94 | 41.07 | 26.64 | 36.56 | 15.41 |
| **3** | BLQ | 5.87 | 9.36 | 38.62 | 38.46 | 29.05 | 39.88 | 13.69 |
| **Mean** | NA | 5.58[a] | 11.16 | 32.39 | 39.39 | 26.46 | 37.21 | 12.81 |
| **St. Dev.** | NA | 0.42 | 1.69 | 7.82 | 1.46 | 2.68 | 2.41 | 3.13 |

LLOQ = 4.84 ng/mL; BLQ = Below limit of quantitation; NA = Not applicable. [a]Average of two values reported and considered for data analysis.

**Table S12.** Individual plasma concentration-time data of **16g** in male C57BL/6 mice following a single intravenous dose (3 mg/kg).

| **Data Point** | **[Compound 16g] in Plasma (ng/mL)** | | | | | | | |
|---|---|---|---|---|---|---|---|---|
| | **Time (h)** | | | | | | | |
| | **0.1** | **0.25** | **0.5** | **1** | **2** | **4** | **6** | **8** |
| **1** | 792.84 | 276.44 | 157.03 | 74.25 | 27.99 | 8.82 | 9.49 | 4.81 |
| **2** | 801.25 | 252.68 | 369.08 | 78.24 | 20.48 | 59.26[a] | 5.18 | 2.23 |
| **3** | 994.51 | 415.95 | 154.16 | 61.94 | 16.26 | 15.37 | 7.82 | 2.32 |
| **Mean** | 862.87 | 315.02 | 226.76 | 71.48 | 21.58 | 12.10[b] | 7.50 | 3.12 |
| **St. Dev.** | 114.08 | 88.21 | 123.26 | 8.50 | 5.94 | 4.63 | 2.17 | 1.46 |

LLOQ = 0.62 ng/mL; [a]Value excluded from data analysis. [b]Average of two values reported and considered for data analysis.

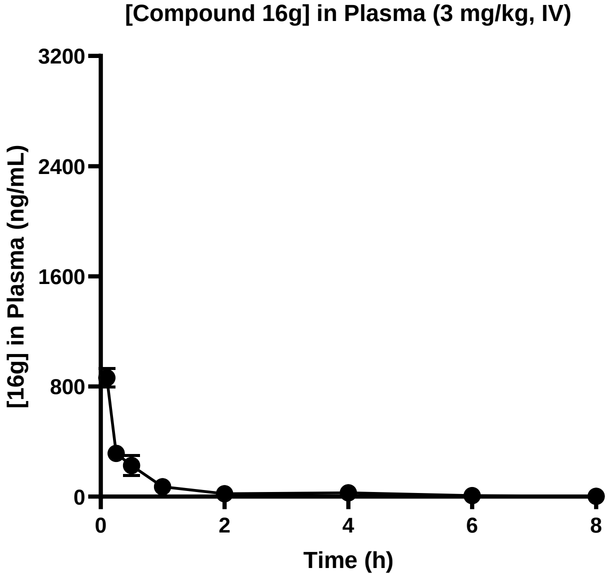


**Figure S46.** Mouse plasma pharmacokinetic profile for compound **16g**. Male C57BL/6 mice (n = 3 per time point) were administered a single intravenous dose (3 mg/kg) of TFV prodrug using 10% PEG-300, 10% Solutol HS-15, and 80% saline as a vehicle. Plasma levels of **16g** was quantified using LC-MS/MS. Data represent the mean concentration at each time point ± SEM. The figures are generated with GraphPad Prism v9.

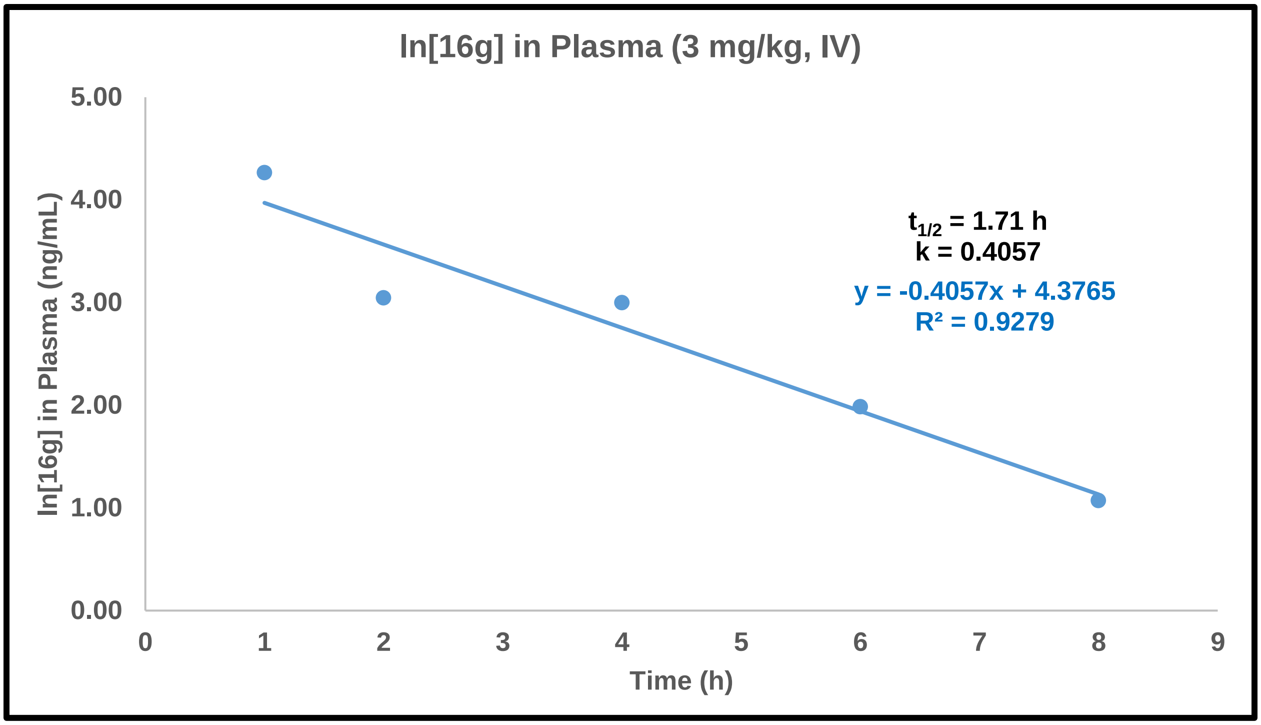


**Figure S47.** Plot of ln[**16g**] in mouse plasma (ng/mL) versus time after intravenous administration (3 mg/kg), which was used to calculate $t_{1/2}$.

**Table S13.** Individual plasma concentration-time data of **16g** in male C57BL/6 mice following a single oral dose (10 mg/kg).

| **Data Point** | **[Compound 16g] in Plasma (ng/mL)** | | | | | | | |
|---|---|---|---|---|---|---|---|---|
| | **Time (h)** | | | | | | | |
| | **0.25** | **0.5** | **1** | **2** | **4** | **8** | **12** | **24** |
| **1** | 30.30 | 46.46 | 59.82 | 43.33 | 19.03 | 6.71 | 11.94 | BLQ |
| **2** | 9.34 | 38.78 | 52.85 | 54.00 | 14.53 | 14.14 | 8.76 | BLQ |
| **3** | 11.24 | 43.91 | 63.04 | 70.24 | 14.39 | 12.91 | 10.73 | BLQ |
| **Mean** | 16.96 | 43.05 | 58.57 | 55.86 | 15.98 | 11.25 | 10.48 | NA |
| **St. Dev.** | 11.59 | 3.91 | 5.21 | 13.55 | 2.64 | 3.98 | 1.61 | NA |

LLOQ = 0.62 ng/mL; BLQ = Below limit of quantitation; NA = Not applicable.

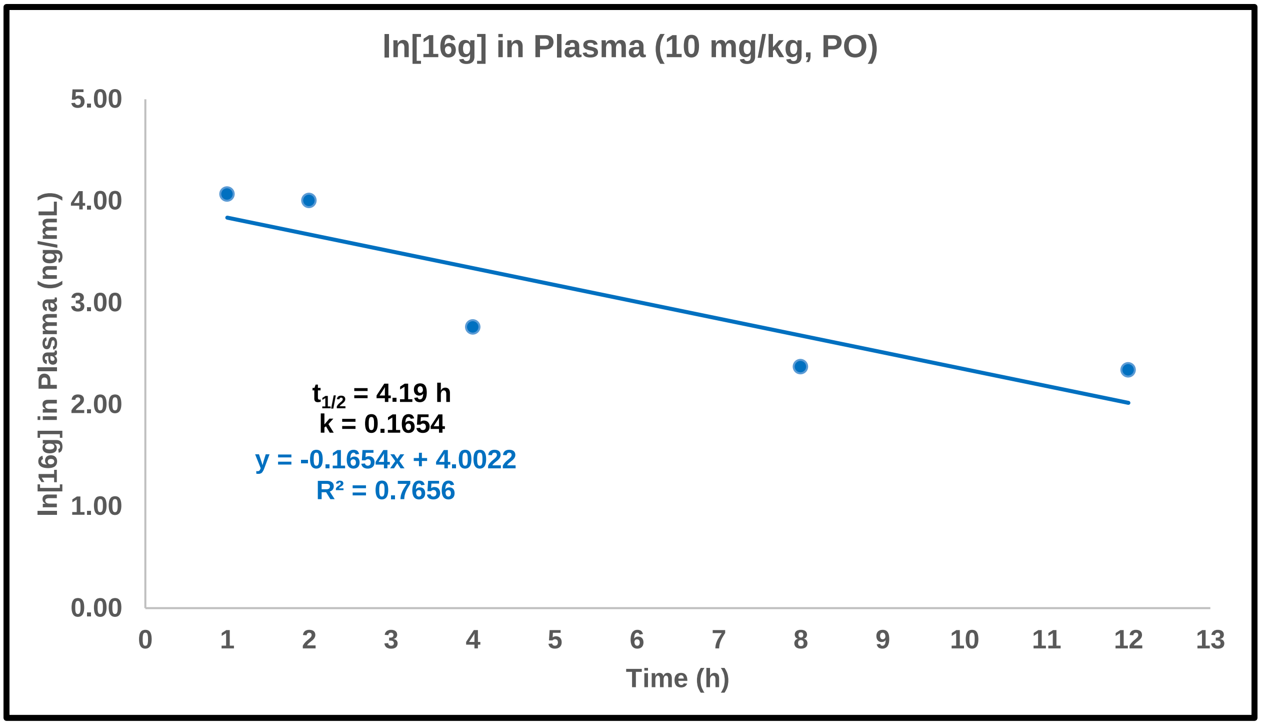


**Figure S48.** Plot of ln[**16g**] in mouse plasma (ng/mL) versus time after oral administration (10 mg/kg), which was used to calculate $t_{1/2}$.

**Table S14.** Individual plasma concentration-time data of TFV in male C57BL/6 mice following a single oral dose of compound **16g** (10 mg/kg).

| **Data Point** | **[TFV] in Plasma (ng/mL)** | | | | | | | |
|---|---|---|---|---|---|---|---|---|
| | **Time (h)** | | | | | | | |
| | **0.25** | **0.5** | **1** | **2** | **4** | **8** | **12** | **24** |
| **1** | BLQ | 7.39 | 19.94 | 14.61 | 25.07 | 24.69 | 32.92 | 18.41 |
| **2** | BLQ | BLQ | 13.02 | 30.28 | 46.98 | 28.83 | 29.34 | 13.36 |
| **3** | BLQ | BLQ | 15.58 | 30.22 | 70.09 | 27.87 | 27.54 | 14.73 |
| **Mean** | NA | 7.39 | 16.18 | 25.04 | 47.38 | 27.13 | 29.93 | 15.50 |
| **St. Dev.** | NA | NA | 3.50 | 9.03 | 22.51 | 2.17 | 2.74 | 2.61 |

LLOQ = 4.84 ng/mL; BLQ = Below limit of quantitation; NA = Not applicable.

**Table S15.** Individual plasma concentration-time data of **16h** in male C57BL/6 mice following a single intravenous dose (3 mg/kg).

| **Data Point** | **[Compound 16h] in Plasma (ng/mL)** | | | | | | | |
|---|---|---|---|---|---|---|---|---|
| | **Time (h)** | | | | | | | |
| | **0.1** | **0.25** | **0.5** | **1** | **2** | **4** | **6** | **8** |
| **1** | NS | 461.77 | 249.66 | 96.89 | 21.00 | 4.47 | 2.03 | 0.83 |
| **2** | 2436.72 | 823.44 | 693.54 | 165.45 | 14.81 | 13.60 | 5.89 | 1.69 |
| **3** | 2315.01 | 281.82 | 274.65 | 117.29 | 16.39 | 8.88 | 3.02 | 1.70 |
| **Mean** | 2375.87[a] | 522.34 | 405.95 | 126.54 | 17.40 | 8.98 | 3.65 | 1.41 |
| **St. Dev.** | 86.06 | 275.84 | 249.37 | 35.20 | 3.22 | 4.57 | 2.00 | 0.50 |

LLOQ = 0.60 ng/mL; NS = Sample lost during processing. [a]Average of two values reported and considered for data analysis.

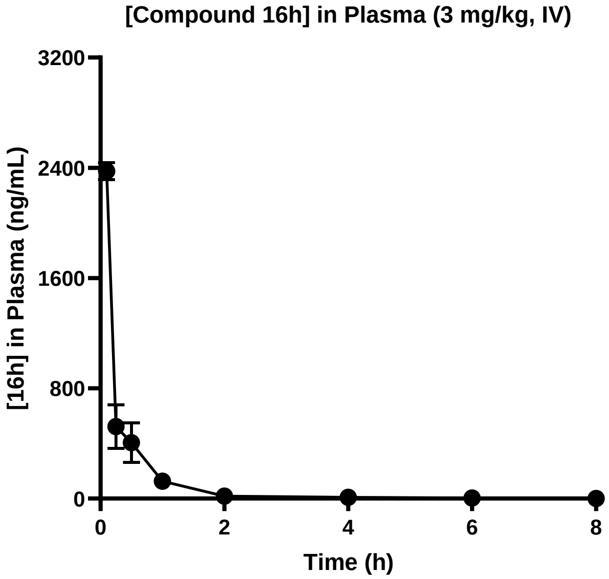


**Figure S49.** Mouse plasma pharmacokinetic profile for compound **16h**. Male C57BL/6 mice (n = 3 per time point) were administered a single intravenous dose (3 mg/kg) of TFV prodrug using 10% PEG-300, 10% Solutol HS-15, and 80% saline as a vehicle. Plasma levels of **16h** was quantified using LC-MS/MS. Data represent the mean concentration at each time point ± SEM. The figures are generated with GraphPad Prism v9.

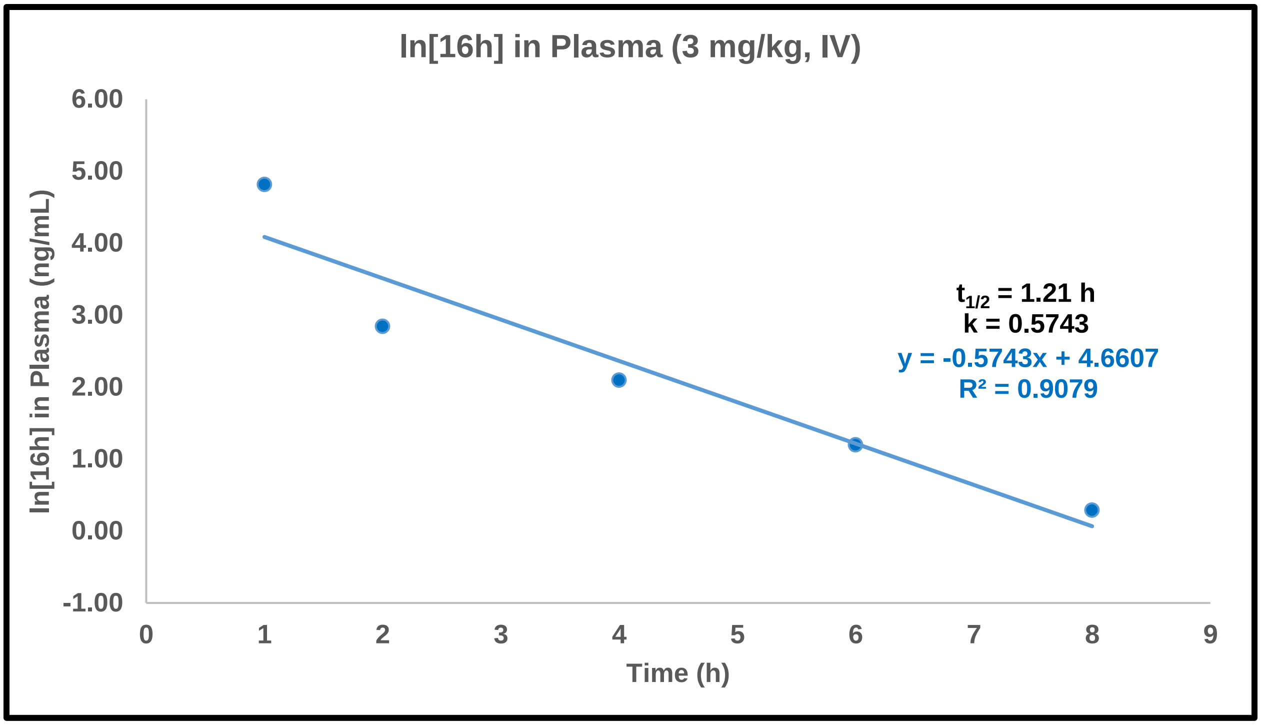


**Figure S50.** Plot of ln[**16h**] in mouse plasma (ng/mL) versus time after intravenous administration (3 mg/kg), which was used to calculate $t_{1/2}$.

**Table S16.** Individual plasma concentration-time data of **16h** in male C57BL/6 mice following a single oral dose (10 mg/kg).

| Data Point | [Compound 16h] in Plasma (ng/mL) | | | | | | | |
|---|---|---|---|---|---|---|---|---|
| | Time (h) | | | | | | | |
| | **0.25** | **0.5** | **1** | **2** | **4** | **8** | **12** | **24** |
| **1** | 46.00 | 96.07 | 161.71 | 205.84 | 69.88 | 44.26 | 47.52 | 0.80 |
| **2** | 32.71 | 52.13 | 104.62 | 201.74 | 54.28 | 25.65 | 47.63 | 0.87 |
| **3** | 67.99 | 89.05 | 153.77 | 177.53 | 53.46 | 38.39 | 19.34 | 0.93 |
| **Mean** | 48.90 | 79.08 | 140.03 | 195.04 | 59.21 | 36.10 | 38.16 | 0.87 |
| **St. Dev.** | 17.82 | 23.60 | 30.92 | 15.30 | 9.25 | 9.51 | 16.30 | 0.07 |

LLOQ = 0.60 ng/mL; BLQ = Below limit of quantitation; NA = Not applicable.

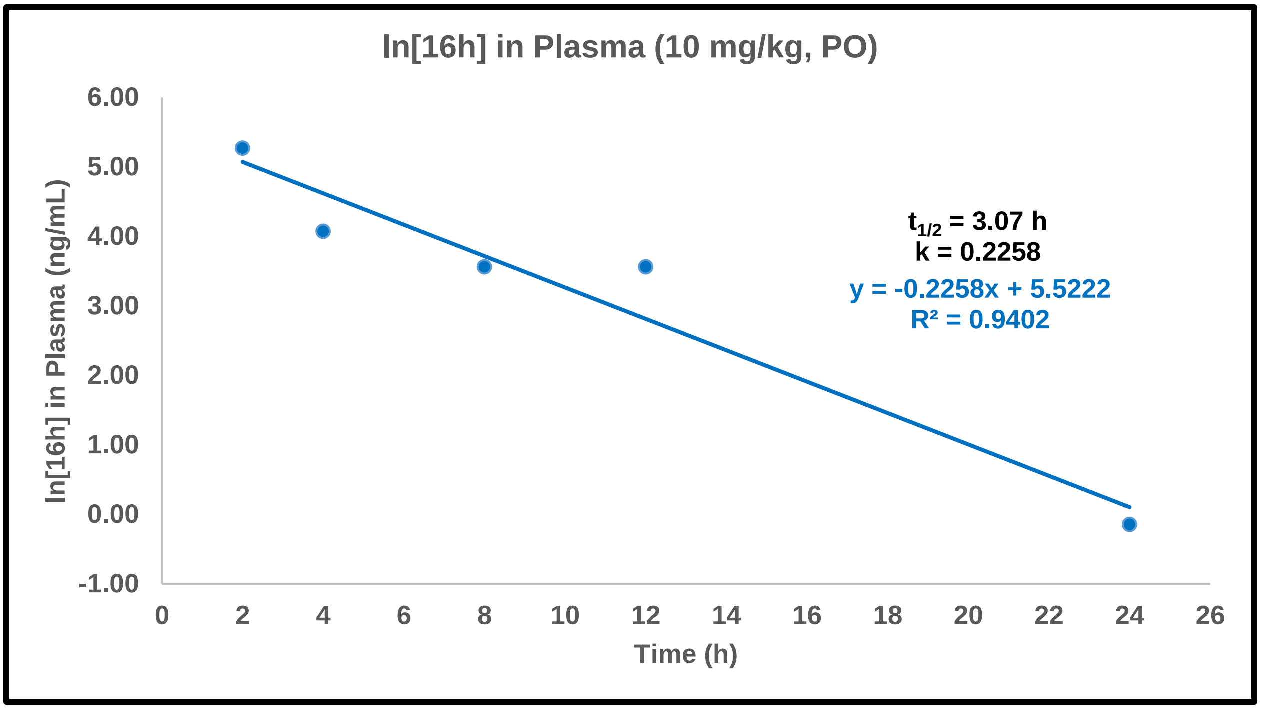


**Figure S51.** Plot of ln[**16h**] in mouse plasma (ng/mL) versus time after oral administration (10 mg/kg), which was used to calculate $t_{1/2}$.

**Table S17.** Individual plasma concentration-time data of TFV in male C57BL/6 mice following a single oral dose of compound **16h** (10 mg/kg).

| **Data Point** | **[TFV] in Plasma (ng/mL)** | | | | | | | |
|---|---|---|---|---|---|---|---|---|
| | **Time (h)** | | | | | | | |
| | **0.25** | **0.5** | **1** | **2** | **4** | **8** | **12** | **24** |
| **1** | BLQ | BLQ | 13.01 | 28.65 | 48.60 | 36.61 | 36.24 | 15.53 |
| **2** | BLQ | BLQ | 9.90 | 21.89 | 43.80 | 38.15 | 34.94 | 19.21 |
| **3** | BLQ | 5.36 | 11.46 | 31.77 | 50.87 | 38.73 | 41.65 | 12.15 |
| **Mean** | NA | 5.36 | 11.46 | 27.44 | 47.76 | 37.83 | 37.61 | 15.63 |
| **St. Dev.** | NA | NA | 1.56 | 5.05 | 3.61 | 1.10 | 3.56 | 3.53 |

LLOQ = 4.84 ng/mL; BLQ = Below limit of quantitation; NA = Not applicable.

**Table S18.** A summary of the mouse plasma PK results obtained after a 3 mg/kg intravenous dose of compounds **2a**, **16g**, and **16h**.

| Summary of Mouse Plasma PK Results (3 mg/kg, i.v.) | | | |
|---|---|---|---|
| **Parameter** | **2a** | **16g** | **16h** |
| Prodrug $T_{max}$ (h) | 0.10 | 0.10 | 0.10 |
| Prodrug $C_{max}$ (ng/mL) | **2060** | 863 | **2380** |
| Prodrug $AUC_{0\text{-}8h}$ (h*ng/mL) | 340 | 373 | **583** |
| Prodrug %F | 100 | 100 | 100 |
| k | 0.467 | 0.406 | 0.574 |
| $R^2$ | 0.85 | 0.93 | 0.91 |
| Prodrug $t_{1/2}$ (h) | 1.49 | **1.71** | 1.21 |
| Prodrug Cl (L/h/kg) | 8.82 | 8.05 | **5.15** |
| Prodrug Vd (L/kg) | **18.9** | **19.9** | 8.97 |

**Table S19.** A summary of the mouse plasma PK results obtained after a 10 mg/kg oral dose of compounds **2a**, **16g**, and **16h**.

| Summary of Mouse Plasma PK Results (10 mg/kg, p.o.) | | | | | |
|---|---|---|---|---|---|
| **Parameter** | **2a** | **16g** | **16h** | **TXL** | **1** |
| Prodrug $T_{max}$ (h) | 1.00 | 1.00 | 2.00 | 1.00 | 2.00 |
| Prodrug $C_{max}$ (ng/mL) | 73.3 | 58.6 | **195** | 125 | 125 |
| Prodrug $AUC_{0\text{-}24h}$ (h*ng/mL) | 219 | 260 | **1070** | 301 | **1280** |
| Prodrug %F | 19.3 | 20.9 | **54.9** | - | - |
| k | 0.199 | 0.165 | 0.226 | 0.237 | 0.080 |
| $R^2$ | 0.65 | 0.77 | 0.94 | 0.73 | 0.96 |
| Prodrug $t_{1/2}$ (h) | 3.49 | **4.19** | 3.07 | 2.92 | **8.66** |
| Prodrug Cl (L/h/kg) | 8.82 | 8.05 | **5.15** | - | - |
| Prodrug Vd (L/kg) | **44.4** | **48.7** | 22.8 | - | - |
| TFV $T_{max}$ (h) | 4.00 | 4.00 | 4.00 | 2.00 | 8.00 |
| TFV $C_{max}$ (ng/mL) | **39.4** | 47.4 | 47.8 | 19.6 | **22.1** |
| TFV $AUC_{0\text{-}24h}$ (h*ng/mL) | 657 | **635** | 740 | 132 | **337** |
| Prodrug $AUC_{0\text{-}24h}$ / TFV $AUC_{0\text{-}24h}$ | 0.333 | 0.409 | **1.44** | 2.29 | **3.80** |

*Liver Pharmacokinetics*

**Table S20.** Individual liver concentration-time data of **2a** in male C57BL/6 mice following a single oral dose (10 mg/kg).

| Data Point | [Compound 2a] in Liver (ng/mL) | | |
|---|---|---|---|
| | Time (h) | | |
| | 1 | 4 | 24 |
| 1 | 1385.45 | 251.10 | BLQ |
| 2 | 867.60 | 406.85 | BLQ |
| 3 | 1488.20 | 526.00 | 3.95 |
| Mean | 1247.08 | 394.65 | 3.95 |
| St. Dev. | 332.63 | 137.86 | NA |

LLOQ = 0.58 ng/mL; BLQ = Below limit of quantitation; NA = Not applicable.

**Table S21.** Individual liver concentration-time data of TFV in male C57BL/6 mice following a single oral dose of compound **2a** (10 mg/kg).

| Data Point | [TFV] in Liver (ng/mL) | | |
|---|---|---|---|
| | Time (h) | | |
| | 1 | 4 | 24 |
| 1 | 328.05 | 1355.10 | 1504.75 |
| 2 | 141.70 | 1459.75 | 836.75 |
| 3 | 297.15 | 1507.55 | 1296.45 |
| Mean | 255.63 | 1440.80 | 1212.65 |
| St. Dev. | 99.87 | 77.97 | 341.79 |

LLOQ = 4.84 ng/mL

**Table S22.** Individual liver concentration-time data of **16g** in male C57BL/6 mice following a single oral dose (10 mg/kg).

| Data Point | [Compound 16g] in Liver (ng/mL) | | |
|---|---|---|---|
| | Time (h) | | |
| | 1 | 4 | 24 |
| **1** | 3090.85 | 4025.35 | 43.60 |
| **2** | 9855.10 | 3219.80 | 14.55 |
| **3** | 3053.75 | 7194.65 | 16.10 |
| **Mean** | 5333.23 | 4813.27 | 24.75 |
| **St. Dev.** | 3916.10 | 2101.30 | 16.34 |

LLOQ = 0.62 ng/mL

**Table S23.** Individual liver concentration-time data of TFV in male C57BL/6 mice following a single oral dose of compound **16g** (10 mg/kg).

| Data Point | [TFV] in Liver (ng/mL) | | |
|---|---|---|---|
| | Time (h) | | |
| | 1 | 4 | 24 |
| **1** | 1208.60 | 3230.65 | 6202.85 |
| **2** | 1149.05 | 3170.55 | 2670.00 |
| **3** | 329.55 | 1780.45 | 3074.60 |
| **Mean** | 895.73 | 2727.22 | 3982.48 |
| **St. Dev.** | 491.23 | 820.47 | 1933.51 |

LLOQ = 4.84 ng/mL

**Table S24.** Individual liver concentration-time data of **16h** in male C57BL/6 mice following a single oral dose (10 mg/kg).

| Data Point | [Compound 16h] in Liver (ng/mL) | | |
|---|---|---|---|
| | Time (h) | | |
| | 1 | 4 | 24 |
| **1** | 1646.45 | 2303.75 | 30.05 |
| **2** | 1573.85 | 2593.00 | 11.40 |
| **3** | 1391.25 | 1550.40 | 55.75 |
| **Mean** | 1537.18 | 2149.05 | 32.40 |
| **St. Dev.** | 131.49 | 538.24 | 22.27 |

LLOQ = 1.19 ng/mL

**Table S25.** Individual liver concentration-time data of TFV in male C57BL/6 mice following a single oral dose of compound **16h** (10 mg/kg).

| Data Point | [TFV] in Liver (ng/mL) | | |
|---|---|---|---|
| | Time (h) | | |
| | 1 | 4 | 24 |
| **1** | 561.45 | 2758.55 | 5037.40 |
| **2** | 350.10 | 2476.95 | 3723.70 |
| **3** | 606.00 | 1752.00 | 2949.15 |
| **Mean** | 505.85 | 2329.17 | 3903.42 |
| **St. Dev.** | 136.71 | 519.29 | 1055.66 |

LLOQ = 4.84 ng/mL

**Table S26.** A summary of the mouse liver PK results obtained after a 10 mg/kg oral dose of compounds **2a**, **16g**, and **16h**.

| **Summary of Mouse Liver PK Results (10 mg/kg, p.o.)** | | | | | |
|---|---|---|---|---|---|
| **Parameter** | **2a** | **16g** | **16h** | **TXL** | **1** |
| Prodrug $T_{max}$ (h) | 1.00 | 1.00 | 4.00 | 1.00 | 2.00 |
| Prodrug $C_{max}$ (ng/mL) | 1250 | **5330** | **2150** | 2730 | **8710** |
| Prodrug $AUC_{0\text{-}24h}$ (h*ng/mL) | 6450 | **63600** | **27300** | 4970 | **109000** |
| TFV $T_{max}$ (h) | 4.00 | 24.0 | 24.0 | 17.3 | 24.0 |
| TFV $C_{max}$ (ng/mL) | **1440** | 3980 | 3900 | 1460 | **1120** |
| TFV $AUC_{0\text{-}24h}$ (h*ng/mL) | **29100** | 72500 | 66600 | 26100 | **22600** |
| Prodrug $AUC_{0\text{-}24h}$ / TFV $AUC_{0\text{-}24h}$ | 0.222 | 0.877 | 0.411 | 0.190 | **4.81** |

*Kidney Pharmacokinetics*

**Table S27.** Individual kidney concentration-time data of **2a** in male C57BL/6 mice following a single oral dose (10 mg/kg).

| **Data Point** | **[Compound 2a] in Kidney (ng/mL)** | | |
|---|---|---|---|
| | **Time (h)** | | |
| | **1** | **4** | **24** |
| **1** | 640.15 | 370.70 | 44.40 |
| **2** | 343.90 | 473.30 | 46.80 |
| **3** | 758.80 | 310.25 | 41.85 |
| **Mean** | 580.95 | 384.75 | 44.35 |
| **St. Dev.** | 213.69 | 82.43 | 2.48 |

LLOQ = 0.58 ng/mL

**Table S28.** Individual kidney concentration-time data of TFV in male C57BL/6 mice following a single oral dose of compound **2a** (10 mg/kg).

| **Data Point** | **[TFV] in Kidney (ng/mL)** | | |
|---|---|---|---|
| | **Time (h)** | | |
| | **1** | **4** | **24** |
| **1** | 109.60 | 607.50 | 771.60 |
| **2** | 124.95 | 688.15 | 804.40 |
| **3** | 59.40 | 668.25 | 507.85 |
| **Mean** | 97.98 | 654.63 | 694.62 |
| **St. Dev.** | 34.28 | 42.01 | 162.57 |

LLOQ = 9.67 ng/mL

**Table S29.** Individual kidney concentration-time data of **16g** in male C57BL/6 mice following a single oral dose (10 mg/kg).

| Data Point | [Compound 16g] in Kidney (ng/mL) | | |
|---|---|---|---|
| | Time (h) | | |
| | 1 | 4 | 24 |
| **1** | 318.10 | 579.95 | 27.00 |
| **2** | 264.60 | 705.05 | 24.30 |
| **3** | 348.35 | 521.75 | 25.00 |
| **Mean** | 310.35 | 602.25 | 25.43 |
| **St. Dev.** | 42.41 | 93.66 | 1.40 |

LLOQ = 0.62 ng/mL

**Table S30.** Individual kidney concentration-time data of TFV in male C57BL/6 mice following a single oral dose of compound **16g** (10 mg/kg).

| Data Point | [TFV] in Kidney (ng/mL) | | |
|---|---|---|---|
| | Time (h) | | |
| | 1 | 4 | 24 |
| **1** | 300.55 | 599.60 | 1821.60 |
| **2** | 179.85 | 735.25 | 1433.25 |
| **3** | 144.90 | 1076.70 | 1008.75 |
| **Mean** | 208.43 | 803.85 | 1421.20 |
| **St. Dev.** | 81.67 | 245.84 | 406.56 |

LLOQ = 9.67 ng/mL

**Table S31.** Individual kidney concentration-time data of **16h** in male C57BL/6 mice following a single oral dose (10 mg/kg).

| Data Point | [Compound 16h] in Kidney (ng/mL) | | |
|---|---|---|---|
| | Time (h) | | |
| | 1 | 4 | 24 |
| **1** | 233.65 | 820.65 | 123.60 |
| **2** | 240.40 | 926.85 | 47.70 |
| **3** | 362.25 | 636.55 | 196.30 |
| **Mean** | 278.77 | 794.68 | 122.53 |
| **St. Dev.** | 72.38 | 146.88 | 74.31 |

LLOQ = 2.97 ng/mL

**Table S32.** Individual kidney concentration-time data of TFV in male C57BL/6 mice following a single oral dose of compound **16h** (10 mg/kg).

| Data Point | [TFV] in Kidney (ng/mL) | | |
|---|---|---|---|
| | Time (h) | | |
| | 1 | 4 | 24 |
| **1** | 125.80 | 662.35 | 1341.55 |
| **2** | 89.20 | 536.70 | 1929.95 |
| **3** | 49.30 | 830.05 | 980.20 |
| **Mean** | 88.10 | 676.37 | 1417.23 |
| **St. Dev.** | 38.26 | 147.18 | 479.38 |

LLOQ = 9.67 ng/mL

**Table S33.** A summary of the mouse kidney PK results obtained after a 10 mg/kg oral dose of compounds **2a**, **16g**, and **16h**.

| **Summary of Mouse Kidney PK Results (10 mg/kg, p.o.)** | | | |
|---|---|---|---|
| **Parameter (p.o.)** | **2a** | **16g** | **16h** |
| **Prodrug $T_{max}$ (h)** | 1.00 | 4.00 | 4.00 |
| **Prodrug $C_{max}$ (ng/mL)** | 581 | 602 | **795** |
| **Prodrug $AUC_{0\text{-}24h}$ (h*ng/mL)** | 5740 | 7650 | **10800** |
| **TFV $T_{max}$ (h)** | 24.0 | 24.0 | 24.0 |
| **TFV $C_{max}$ (ng/mL)** | **695** | 1420 | 1420 |
| **TFV $AUC_{0\text{-}24h}$ (h*ng/mL)** | **14600** | 23800 | 22100 |
| **Prodrug $AUC_{0\text{-}24h}$ / TFV $AUC_{0\text{-}24h}$** | 0.393 | 0.322 | **0.488** |

*Brain Pharmacokinetics*

**Table S34.** Individual brain concentration-time data of **2a** in male C57BL/6 mice following a single oral dose (10 mg/kg).

| Data Point | [Compound 2a] in Brain (ng/mL) | | |
|---|---|---|---|
| | Time (h) | | |
| | 1 | 4 | 24 |
| **1** | 64.38 | 1.77 | BLQ |
| **2** | 45.63 | 2.61 | BLQ |
| **3** | 21.39 | BLQ | BLQ |
| **Mean** | 43.80 | 2.19[a] | NA |
| **St. Dev.** | 21.55 | 0.59 | NA |

LLOQ = 0.58 ng/mL; BLQ = Below limit of quantitation; NA = Not applicable. [a]Average of two values reported and considered for data analysis.

**Table S35.** Individual brain concentration-time data of TFV in male C57BL/6 mice following a single oral dose of compound **2a** (10 mg/kg).

| Data Point | [TFV] in Brain (ng/mL) | | |
|---|---|---|---|
| | Time (h) | | |
| | 1 | 4 | 24 |
| **1** | BLQ | BLQ | BLQ |
| **2** | BLQ | BLQ | BLQ |
| **3** | BLQ | BLQ | BLQ |
| **Mean** | NA | NA | NA |
| **St. Dev.** | NA | NA | NA |

LLOQ = 4.84 ng/mL; BLQ = Below limit of quantitation; NA = Not applicable.

**Table S36.** Individual brain concentration-time data of **16g** in male C57BL/6 mice following a single oral dose (10 mg/kg).

| Data Point | [Compound 16g] in Brain (ng/mL) | | |
|---|---|---|---|
| | Time (h) | | |
| | 1 | 4 | 24 |
| **1** | 3.99 | 6.57 | BLQ |
| **2** | 3.87 | 6.87 | BLQ |
| **3** | 1.86 | 3.78 | BLQ |
| **Mean** | 3.24 | 5.74 | NA |
| **St. Dev.** | 1.20 | 1.70 | NA |

LLOQ = 0.62 ng/mL; BLQ = Below limit of quantitation; NA = Not applicable.

**Table S37.** Individual brain concentration-time data of TFV in male C57BL/6 mice following a single oral dose of compound **16g** (10 mg/kg).

| Data Point | [TFV] in Brain (ng/mL) | | |
|---|---|---|---|
| | Time (h) | | |
| | 1 | 4 | 24 |
| **1** | BLQ | BLQ | BLQ |
| **2** | BLQ | BLQ | BLQ |
| **3** | BLQ | BLQ | BLQ |
| **Mean** | NA | NA | NA |
| **St. Dev.** | NA | NA | NA |

LLOQ = 4.84 ng/mL; BLQ = Below limit of quantitation; NA = Not applicable.

**Table S38.** Individual brain concentration-time data of **16h** in male C57BL/6 mice following a single oral dose (10 mg/kg).

| Data Point | [Compound 16h] in Brain (ng/mL) | | |
|---|---|---|---|
| | Time (h) | | |
| | 1 | 4 | 24 |
| **1** | 35.67 | 9.99 | BLQ |
| **2** | 7.80 | 6.78 | BLQ |
| **3** | 4.98 | BLQ | BLQ |
| **Mean** | 16.15 | 8.39[a] | NA |
| **St. Dev.** | 16.96 | 2.27 | NA |

LLOQ = 1.19 ng/mL; BLQ = Below limit of quantitation; NA = Not applicable. [a]Average of two values reported and considered for data analysis.

**Table S39.** Individual brain concentration-time data of TFV in male C57BL/6 mice following a single oral dose of compound **16h** (10 mg/kg).

| Data Point | [TFV] in Brain (ng/mL) | | |
|---|---|---|---|
| | Time (h) | | |
| | 1 | 4 | 24 |
| **1** | BLQ | BLQ | BLQ |
| **2** | BLQ | BLQ | BLQ |
| **3** | BLQ | BLQ | BLQ |
| **Mean** | NA | NA | NA |
| **St. Dev.** | NA | NA | NA |

LLOQ = 9.67 ng/mL; BLQ = Below limit of quantitation; NA = Not applicable.

**Table S40.** A summary of the mouse brain PK results obtained after a 10 mg/kg oral dose of compounds **2a**, **16g**, and **16h**.

| Summary of Mouse Brain PK Results (10 mg/kg, p.o.) | | | |
|---|---|---|---|
| **Parameter (p.o.)** | **2a** | **16g** | **16h** |
| **Prodrug $T_{max}$ (h)** | 1.00 | 4.00 | 1.00 |
| **Prodrug $C_{max}$ (ng/mL)** | **43.8** | 5.74 | 16.2 |
| **Prodrug $AUC_{0\text{-}24h}$ (h*ng/mL)** | **69.0** | 13.5 | 36.8 |
| **TFV $T_{max}$ (h)** | - | - | - |
| **TFV $C_{max}$ (ng/mL)** | - | - | - |
| **TFV $AUC_{0\text{-}24h}$ (h*ng/mL)** | - | - | - |

## LC-MS Traces For HTS-Derived TFV Prodrugs

*Example of Acetylenic Terminal Group Degradation Over Time (Compound **15h**)*

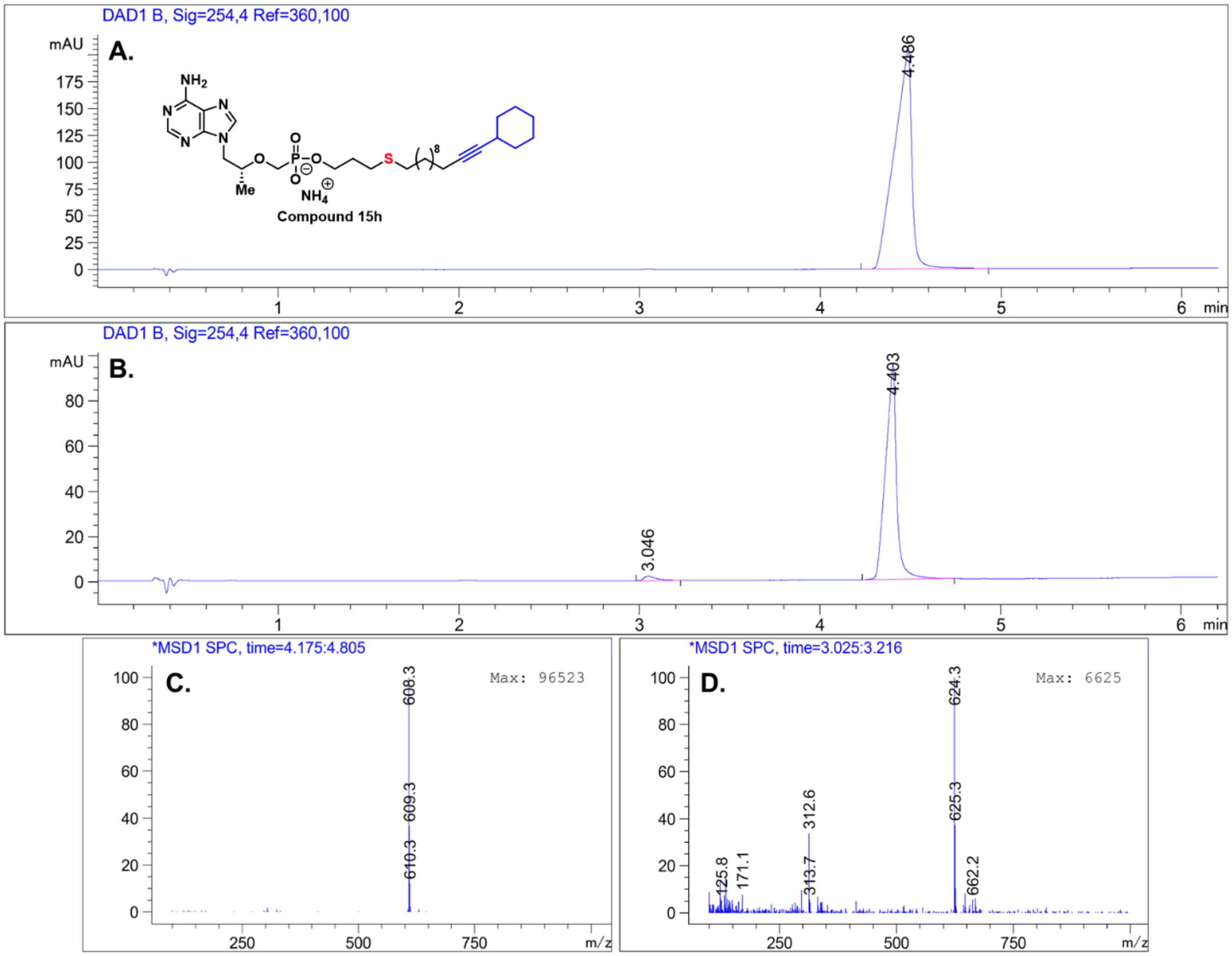


**E.** Signal 2: DAD1 B, Sig=254,4 Ref=360,100

| Peak # | RetTime [min] | Type | Width [min] | Area [mAU*s] | Height [mAU] | Area % |
|---|---|---|---|---|---|---|
| 1 | 3.046 | BB | 0.0622 | 9.25011 | 1.92191 | 2.2469 |
| 2 | 4.403 | BB | 0.0572 | 402.42627 | 95.35167 | 97.7531 |
| Totals : | | | | 411.67638 | 97.27358 | |

**Figure S52.** LC-MS traces of compound **15e** immediately after reversed-phase purification (**A**) and over several weeks of storage at -20 $^{o}$C (**B**). Compound **15e** has an $[M + H]^{+}$ *m/z* of 608.3 (**C**), while the impurity has an $[M + H]^{+}$ *m/z* of 624.3 (**D**) and accounts for 2.2% of total peak area % (**E**). Analysis was performed on an Agilent InfinityLab Poroshell 120 EC-C8 (2.1 mm x 50 mm, 2.7 µm) column at 40 $^{o}$C. Method: 40-95% $CH_3CN$ in $H_2O$ (0.1% FA) over 6 min (254 nm).

*LC-MS Traces For Compounds **2a**, **16g**, and **16h** Prior to In Vivo Analysis*

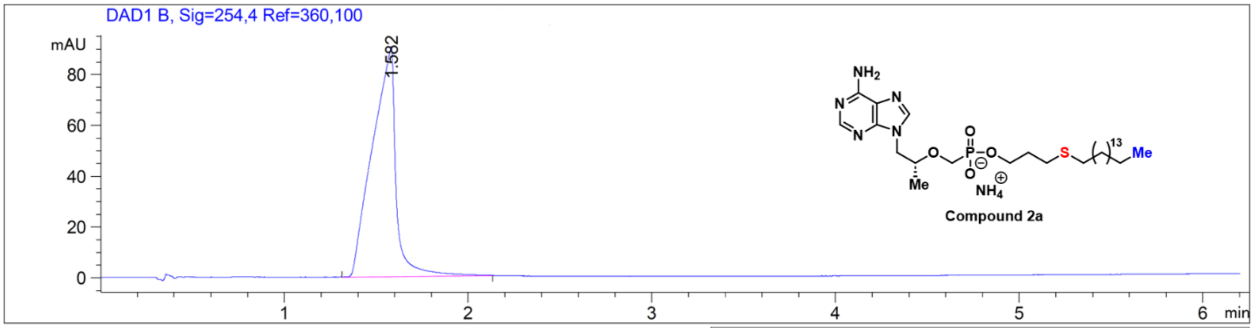


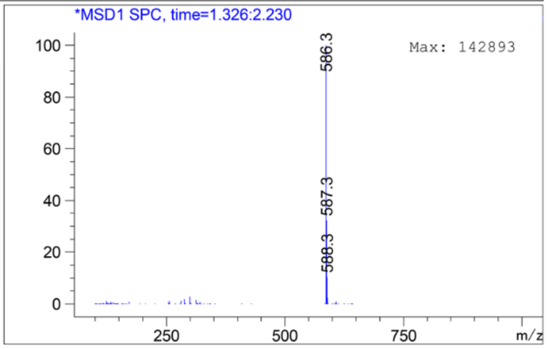


Signal 2: DAD1 B, Sig=254,4 Ref=360,100

| Peak # | RetTime [min] | Type | Width [min] | Area [mAU*s] | Height [mAU] | Area % |
|---|---|---|---|---|---|---|
| 1 | 1.582 | BB | 0.1114 | 809.40289 | 90.42661 | 100.0000 |
| Totals : | | | | 809.40289 | 90.42661 | |

**Figure S53.** LC-MS trace of compound **2a**. Analysis was performed on an Agilent InfinityLab Poroshell 120 EC-C8 (2.1 mm x 50 mm, 2.7 µm) column at 40 °C. Method: 65-95% $CH_3CN$ in $H_2O$ (0.1% FA) over 6 min (254 nm).

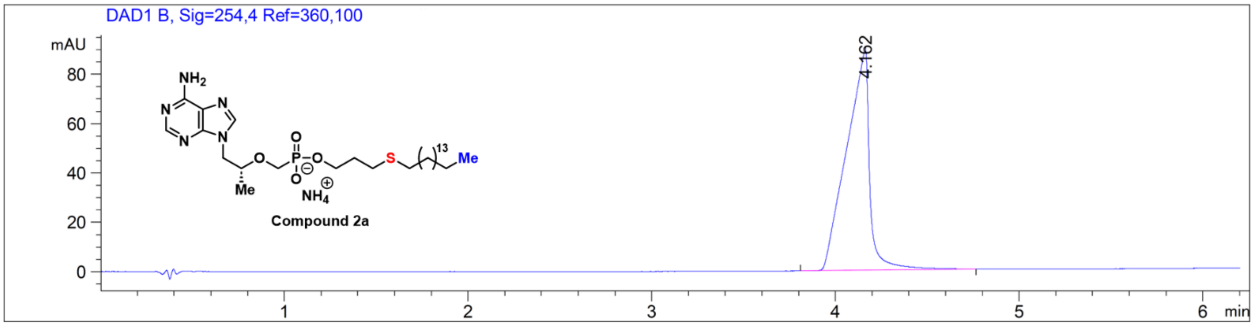


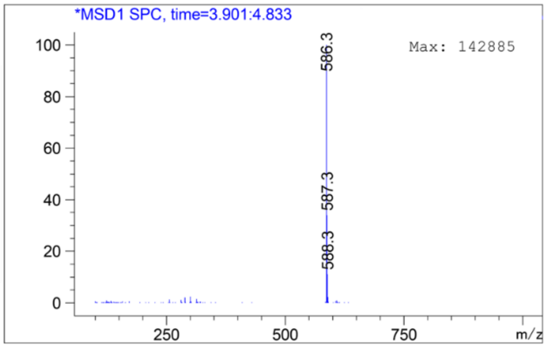


Signal 2: DAD1 B, Sig=254,4 Ref=360,100

| Peak # | RetTime [min] | Type | Width [min] | Area [mAU*s] | Height [mAU] | Area % |
|---|---|---|---|---|---|---|
| 1 | 4.162 | BB | 0.1101 | 787.50153 | 90.45956 | 100.0000 |
| Totals : | | | | 787.50153 | 90.45956 | |

**Figure S54.** LC-MS trace of compound **2a**. Analysis was performed on an Agilent InfinityLab Poroshell 120 EC-C8 (2.1 mm x 50 mm, 2.7 µm) column at 40 °C. Method: 50-95% $CH_3CN$ in $H_2O$ (0.1% FA) over 6 min (254 nm).

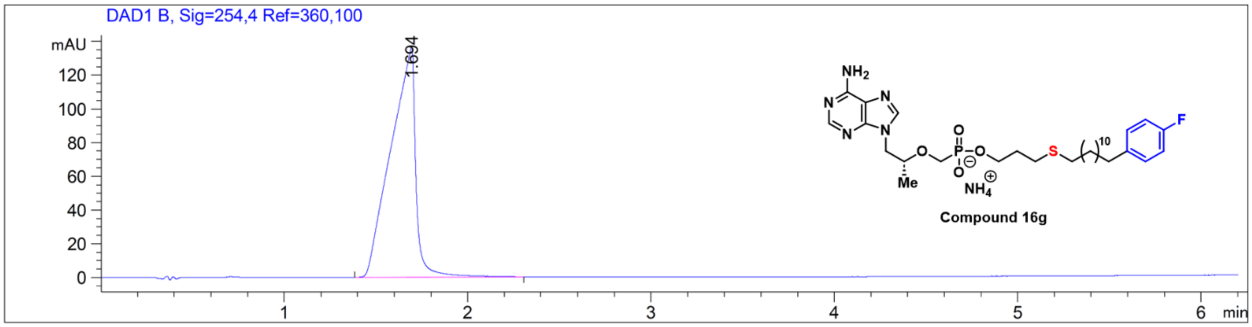


Signal 2: DAD1 B, Sig=254,4 Ref=360,100

| Peak # | RetTime [min] | Type | Width [min] | Area [mAU*s] | Height [mAU] | Area % |
|---|---|---|---|---|---|---|
| 1 | 1.694 | BB | 0.1178 | 1293.02222 | 136.74167 | 100.0000 |

Totals : 1293.02222 136.74167

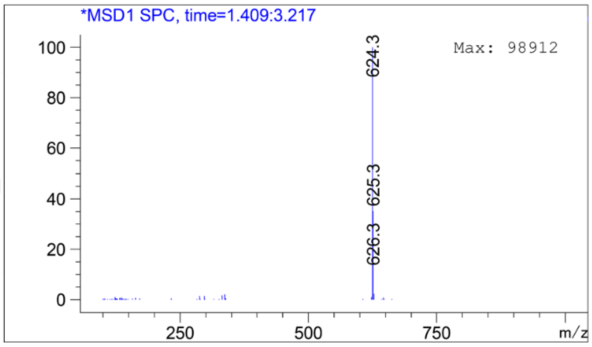


**Figure S55.** LC-MS trace of compound **16g**. Analysis was performed on an Agilent InfinityLab Poroshell 120 EC-C8 (2.1 mm x 50 mm, 2.7 µm) column at 40 °C. Method: 55-95% $CH_3CN$ in $H_2O$ (0.1% FA) over 6 min (254 nm).

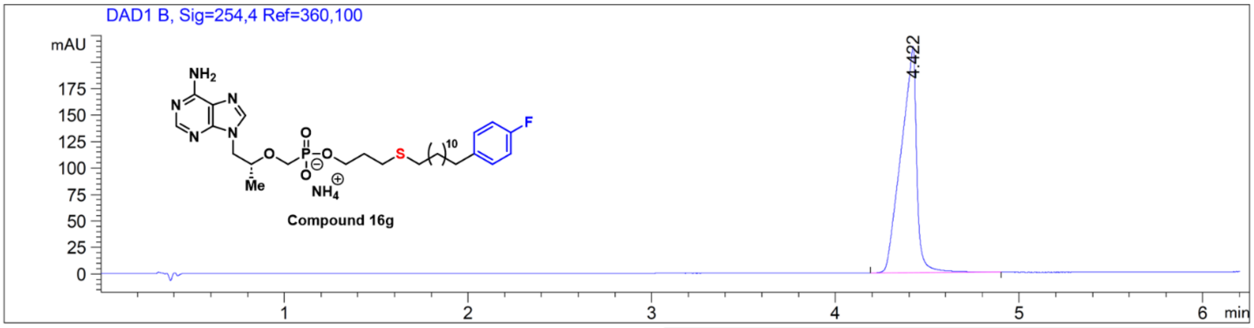


Signal 2: DAD1 B, Sig=254,4 Ref=360,100

| Peak # | RetTime [min] | Type | Width [min] | Area [mAU*s] | Height [mAU] | Area % |
|---|---|---|---|---|---|---|
| 1 | 4.422 | BB | 0.0769 | 1261.96826 | 213.56256 | 100.0000 |

Totals : 1261.96826 213.56256

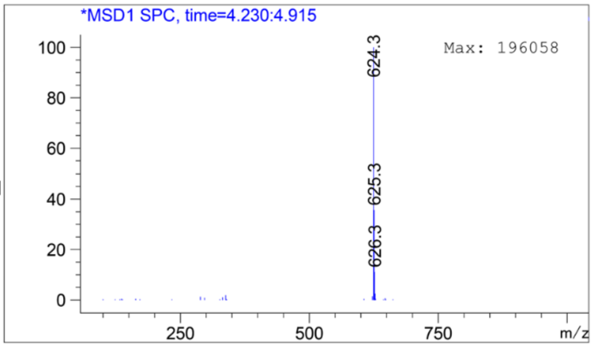


**Figure S56.** LC-MS trace of compound **16g**. Analysis was performed on an Agilent InfinityLab Poroshell 120 EC-C8 (2.1 mm x 50 mm, 2.7 µm) column at 40 °C. Method: 40-95% $CH_3CN$ in $H_2O$ (0.1% FA) over 6 min (254 nm).

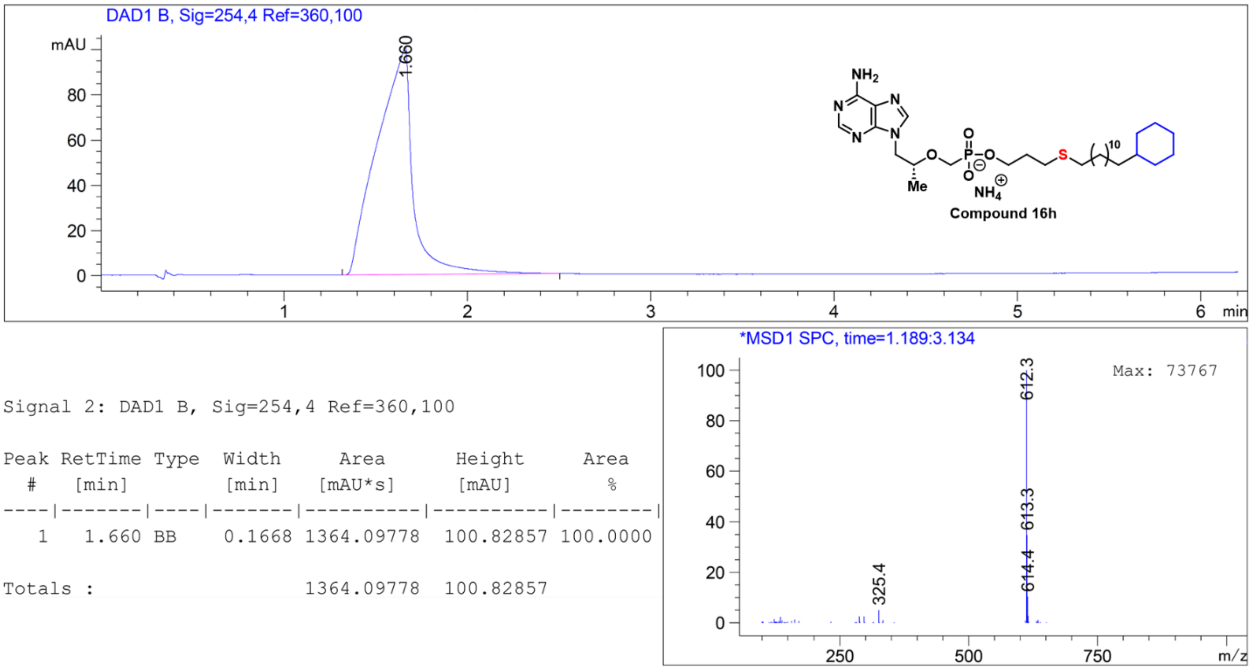


**Figure S57.** LC-MS trace of compound **16h**. Analysis was performed on an Agilent InfinityLab Poroshell 120 EC-C8 (2.1 mm x 50 mm, 2.7 µm) column at 40 $^{o}$C. Method: 70-95% $CH_3CN$ in $H_2O$ (0.1% FA) over 6 min (254 nm).

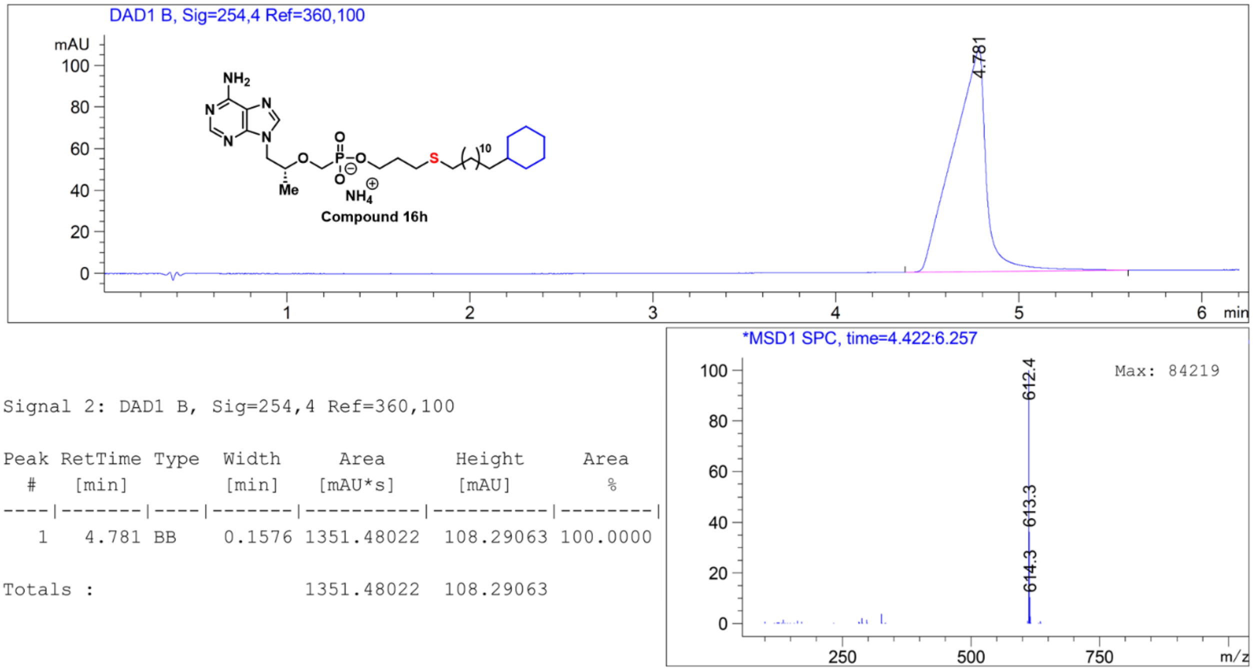


**Figure S58.** LC-MS trace of compound **16h**. Analysis was performed on an Agilent InfinityLab Poroshell 120 EC-C8 (2.1 mm x 50 mm, 2.7 µm) column at 40 $^{o}$C. Method: 50-95% $CH_3CN$ in $H_2O$ (0.1% FA) over 6 min (254 nm).

## Predicted Pharmacological Properties for Key Lipid Intermediates and TFV Prodrugs

**Table S41.** Summary of predicted pharmacological properties for key lipid intermediates and TFV prodrugs.[a]

| Entry | Formula | MW | Rotatable Bonds | HBA | HBD | clogP[b] | tPSA (Å$^2$) |
|---|---|---|---|---|---|---|---|
| **S1** | $C_{19}H_{40}O_2$ | 300.52 | 18 | 2 | 1 | 7.50 | 29.46 |
| **TXL** | $C_{28}H_{52}N_5O_5P$ | 569.72 (OH)<br>568.71 ($O^-$) | 25 | 8 | 2 (OH)<br>1 ($O^-$) | 6.69 | 144.42 (OH)<br>147.25 ($O^-$) |
| **S2** | $C_{19}H_{37}F_3O_2$ | 354.49 | 19 | 5 | 1 | 7.71 | 29.46 |
| **1** | $C_{28}H_{52}F_3N_5O_5P$ | 623.69 (OH)<br>622.68 ($O^-$) | 26 | 11 | 2 (OH)<br>1 ($O^-$) | 6.90 | 144.42 (OH)<br>147.25 ($O^-$) |
| **7a** | $C_{19}H_{40}OS$ | 316.59 | 18 | 1 | 1 | 8.30 | 45.53 |
| **2a** | $C_{28}H_{52}N_5O_4PS$ | 585.78 (OH)<br>584.77 ($O^-$) | 25 | 7 | 2 (OH)<br>1 ($O^-$) | 7.49 | 160.49 (OH)<br>163.32 ($O^-$) |
| **14g** | $C_{21}H_{35}FOS$ | 354.57 | 16 | 2 | 1 | 7.65 | 45.53 |
| **16g** | $C_{30}H_{47}FN_5O_4PS$ | 623.76 (OH)<br>622.75 ($O^-$) | 23 | 8 | 2 (OH)<br>1 ($O^-$) | 6.84 | 160.49 (OH)<br>163.32 ($O^-$) |
| **14h** | $C_{21}H_{42}OS$ | 342.62 | 16 | 1 | 1 | 8.88 | 45.53 |
| **16h** | $C_{30}H_{54}N_5O_4PS$ | 611.82 (OH)<br>610.81 ($O^-$) | 23 | 7 | 2 (OH)<br>1 ($O^-$) | 8.07 | 160.49 (OH)<br>163.32 ($O^-$) |

[a]ADME parameters were predicted using SwissADME web service from the Swiss Institute of Bioinformatics. Details and corresponding literature on methodologies and parameter validation can be found at http://www.swissadme.ch. [b]cLogP was calculated using XLOGP3 algorithm. MW = Molecular weight; HBA = Hydrogen-bond acceptors; HBD = Hydrogen-bond donors; tPSA = Topological polar surface area.

## References


1. For general synthesis and NMR profiles, see: (a) Grube, A.; Timm, C.; Köck, M. Synthesis and Mass Spectrometric Analysis of Cyclostellettamines H, I, K and L. *Eur. J. Org. Chem.* **2006**, 1285-1296. https://doi.org/10.1002/ejoc.200500208. (b) Drescher, S.; Meister, A.; Blume, A.; Karlsson, G.; Almgren, M.; Dobner, B. General Synthesis and Aggregation Behaviour of a Series of Single-Chain 1,ω-Bis(phosphocholines) *Chem. Eur. J.* **2007**, *13*, 5300-5307. https://doi.org/10.1002/chem.200601866. (c) Schmidt, G.; Timm, C.; Grube, A.; Volk, C. A.; Köck, M. Viscosalines $B_{1,2}$ and $E_{1,2}$: Challenging New 3-Alkyl Pyridinium Alkaloids from the Marine Sponge *Haliclona viscosa. Chem. Eur. J.* **2012**, *18*, 8180-8189. https://doi.org/10.1002/chem.201101362.

2. For synthesis and NMR profile, see: (a) Muñoz, L.; Rosell, G.; Guerrero, A. Penta-deuterated acid precursors in the pheromone biosynthesis of the Egyptian armyworm, *Spodoptera littoralis*. *J. Label Compd. Radiopharm.* **2009**, *52*, 493-498. https://doi.org/10.1002/jlcr.1666. (b) Liu, F.; Zhong, J.; Li, S.; Li, M.; Wu, L.; Wang, Q.; Mao, J.; Liu, S.; Zheng, B.; Wang, M.; Bian, Q. Total Syntheses of (*R*)-Strongylodiols C and D. *J. Nat. Prod.* **2016**, *79*, 244-247. https://doi.org/10.1021/acs.jnatprod.5b00713.

3. For synthesis and NMR profile, see: Dang, H.; Mailig, M.; Lalic, G. Mild Copper-Catalyzed Fluorination of Alkyl Triflates with Potassium Fluoride. *Angew. Chem. Int. Ed.* **2014**, *53*, 6473-6476. https://doi.org/10.1002/anie.201402238.

4. For synthesis and NMR profile, see: Shaikh, R. S.; Keul, P.; Schäfers, M.; Levkau, B.; Haufe, G. New fluorinated agonists for targeting the sphingosin-1-phosphate receptor 1 ($S1P_1$). *Bioorg. Med. Chem. Lett.* **2015**, *25*, 5048-5051. https://doi.org/10.1016/j.bmcl.2015.10.026.
